# Supplementary material for: Synthesis of Bio-Inspired 1,3-Diarylpropene Derivatives via Heck Cross-Coupling and Cytotoxic Evaluation on Breast Cancer Cells
Source: Molecules. 2022 Aug 23;27(17):5373. doi: 10.3390/molecules27175373 (PMC9457622; doi:10.3390/molecules27175373)
Supplement: Supplementary file 1 [file molecules-27-05373-s001.zip › molecules-1813924-supplementary.pdf]

SUPPORTING INFORMATION

Synthesis of Bio-inspired 1,3-Diarylpropene Derivatives via Heck Cross-Coupling and Cytotoxic Evaluation on Breast cancer cells.

Aik Sian Tan, Jaymeer Singh, Nurul Syafiqah Rezali, Musthahimah Muhamad, Nik Nur Syazni Nik Mohamed Kamal, Yvan Six, and Mohamad Nurul Azmi *

Table of Contents

1. General Information 5

2. Preparation of Amide Precursors 3a−3j 6

3. Synthesis of substituted *N*-(2-cinnamylphenyl) amide compounds 11

4. NMR Spectra 31

Figure S1: 500 MHz ^{1}H- and 126 MHz ^{13}C-NMR spectra of compound 3a in $CDCl_3$. 31

Figure S2: 500 MHz ^{1}H- and 126 MHz ^{13}C-NMR spectra of compound 3b in $CDCl_3$. 32

Figure S3: 500 MHz ^{1}H- and 126 MHz ^{13}C-NMR spectra of compound 3c in $CDCl_3$. 33

Figure S4: 500 MHz ^{1}H- and 126 MHz ^{13}C-NMR spectra of compound 3d in $CDCl_3$. 34

Figure S5: 500 MHz ^{1}H- and 126 MHz ^{13}C-NMR spectra of compound 3e in $CDCl_3$. 35

Figure S6: 500 MHz ^{1}H- and 126 MHz ^{13}C-NMR spectra of compound 3f in $CDCl_3$. 36

Figure S7: 500 MHz ^{1}H- and 126 MHz ^{13}C-NMR spectra of compound 3g in $CDCl_3$. 37

Figure S8: 500 MHz ^{1}H- and 126 MHz ^{13}C-NMR spectra of compound 3h in $CDCl_3$. 38

Figure S9: 500 MHz ^{1}H- and 126 MHz ^{13}C-NMR spectra of compound 3i in $CDCl_3$. 39

Figure S10: 500 MHz ^{1}H- and 126 MHz ^{13}C-NMR spectra of compound 3j in $CDCl_3$. 40

Figure S11: 500 MHz ^{1}H- and 126 MHz ^{13}C-NMR spectra of compound 6a,7a in $CDCl_3$. 41

Figure S12: 500 MHz ^{1}H- and 126 MHz ^{13}C-NMR spectra of compound 7a in $CDCl_3$. 42

Figure S13: 500 MHz ^{1}H- and 126 MHz ^{13}C-NMR spectra of compound 6b,7b in $CDCl_3$. 43

Figure S14: 500 MHz ^{1}H- and 126 MHz ^{13}C-NMR spectra of compound 6c,7c in $CDCl_3$. 44

Figure S15: 500 MHz ^{1}H- and 126 MHz ^{13}C-NMR spectra of compound 7c in $CDCl_3$. 45

Figure S16: 500 MHz ^{1}H- and 126 MHz ^{13}C-NMR spectra of compound 6d,7d in $CDCl_3$. 46

Figure S17: 500 MHz ^{1}H- and 126 MHz ^{13}C-NMR spectra of compound 6e,7e in $CDCl_3$. 47

Figure S18: 500 MHz ^{1}H- and 126 MHz ^{13}C-NMR spectra of compound 6f,7f in $CDCl_3$. 48

Figure S19: 500 MHz ^{1}H- and 126 MHz ^{13}C-NMR spectra of compound 7f in $CDCl_3$. 49

Figure S20: 500 MHz ^{1}H- and 126 MHz ^{13}C-NMR spectra of compound 6g,7g in $CDCl_3$. 50
Figure S21: 500 MHz ^{1}H- and 126 MHz ^{13}C-NMR spectra of compound 6h,7h in $CDCl_3$. 51
Figure S22: 500 MHz ^{1}H- and 126 MHz ^{13}C-NMR spectra of compound 7h in $CDCl_3$. 52
Figure S23: 500 MHz ^{1}H- and 126 MHz ^{13}C-NMR spectra of compound 6i,7i in $CDCl_3$. 53
Figure S24: 500 MHz ^{1}H- and 126 MHz ^{13}C-NMR spectra of compound 6i in $CDCl_3$. 54
Figure S25: 500 MHz ^{1}H- and 126 MHz ^{13}C-NMR spectra of compound 7i in $CDCl_3$. 55
Figure S26: 500 MHz ^{1}H- and 126 MHz ^{13}C-NMR spectra of compound 6j,7j in $CDCl_3$. 56
Figure S27: 500 MHz ^{1}H- and 126 MHz ^{13}C-NMR spectra of compound 8a,9a in $CDCl_3$. 57
Figure S28: 500 MHz ^{1}H- and 126 MHz ^{13}C-NMR spectra of compound 9a in $CDCl_3$. 58
Figure S29: 500 MHz ^{1}H- and 126 MHz ^{13}C-NMR spectra of compound 8b,9b in $CDCl_3$. 59
Figure S30: 500 MHz ^{1}H- and 126 MHz ^{13}C-NMR spectra of compound 9b in $CDCl_3$. 60
Figure S31: 500 MHz ^{1}H- and 126 MHz ^{13}C-NMR spectra of compound 8c,9c in $CDCl_3$. 61
Figure S32: 500 MHz ^{1}H- and 126 MHz ^{13}C-NMR spectra of compound 8d,9d in $CDCl_3$. 62
Figure S33: 500 MHz ^{1}H- and 126 MHz ^{13}C-NMR spectra of compound 8e,9e in $CDCl_3$. 63
Figure S34: 500 MHz ^{1}H- and 126 MHz ^{13}C-NMR spectra of compound 8f,9f in $CDCl_3$. 64
Figure S35: 500 MHz ^{1}H- and 126 MHz ^{13}C-NMR spectra of compound 8g,9g in $CDCl_3$. 65
Figure S36: 500 MHz ^{1}H- and 126 MHz ^{13}C-NMR spectra of compound 8h,9h in $CDCl_3$. 66
Figure S37: 500 MHz ^{1}H- and 126 MHz ^{13}C-NMR spectra of compound 9h in $CDCl_3$. 67
Figure S38: 500 MHz ^{1}H- and 126 MHz ^{13}C-NMR spectra of compound 8i,9i in $CDCl_3$. 68
Figure S39: 500 MHz ^{1}H- and 126 MHz ^{13}C-NMR spectra of compound 8j,9j in $CDCl_3$. 69
5. ATR-IR Spectra 70
Figure S40: ATR-FTIR spectrum of compound 3a. 70
Figure S41: ATR-FTIR spectrum of compound 3b. 70
Figure S42: ATR-FTIR spectrum of compound 3c. 71
Figure S43: ATR-FTIR spectrum of compound 3d. 71
Figure S44: ATR-FTIR spectrum of compound 3e. 72
Figure S45: ATR-FTIR spectrum of compound 3f. 72
Figure S46: ATR-FTIR spectrum of compound 3g. 73
Figure S47: ATR-FTIR spectrum of compound 3h. 73
Figure S48: ATR-FTIR spectrum of compound 3i. 74
Figure S49: ATR-FTIR spectrum of compound 3j. 74
Figure S50: ATR-FTIR spectrum of compound 6a,7a. 75
Figure S51: ATR-FTIR spectrum of compound 6b,7b. 76
Figure S52: ATR-FTIR spectrum of compound 6c,7c. 77
Figure S53: ATR-FTIR spectrum of compound 6d,7d. 78
Figure S54: ATR-FTIR spectrum of compound 6e,7e. 79

Figure S55: ATR-FTIR spectrum of compound 6f,7f. 80
Figure S56: ATR-FTIR spectrum of compound 6g,7g. 81
Figure S57: ATR-FTIR spectrum of compound 6h,7h. 82
Figure S58: ATR-FTIR spectrum of compound 6i,7i. 83
Figure S59: ATR-FTIR spectrum of compound 6j,7j. 84
Figure S60: ATR-FTIR spectrum of compound 8a,9a. 85
Figure S61: ATR-FTIR spectrum of compound 8b,9b. 86
Figure S62: ATR-FTIR spectrum of compound 8c,9c. 87
Figure S63: ATR-FTIR spectrum of compound 8d,9d. 88
Figure S64: ATR-FTIR spectrum of compound 8e,9e. 89
Figure S65: ATR-FTIR spectrum of compound 8f,9f. 90
Figure S66: ATR-FTIR spectrum of compound 8g,9g. 91
Figure S67: ATR-FTIR spectrum of compound 8h,9h. 92
Figure S68: ATR-FTIR spectrum of compound 8i,9i. 93
Figure S69: ATR-FTIR spectrum of compound 8j,9j. 94
6. HR-MS Spectra 95
Figure S70: HR-MS spectrum of compound 3d. 95
Figure S71: HR-MS spectrum of compound 3f. 96
Figure S72: HR-MS spectrum of compound 6a,7a. 97
Figure S73: HR-MS spectrum of compound 6b,7b. 98
Figure S74: HR-MS spectrum of compound 6c,7c. 99
Figure S75: HR-MS spectrum of compound 6d,7d. 100
Figure S76: HR-MS spectrum of compound 6e,7e. 101
Figure S77: HR-MS spectrum of compound 6f,7f. 102
Figure S78: HR-MS spectrum of compound 6g,7g. 103
Figure S79: HR-MS spectrum of compound 6h,7h. 104
Figure S80: HR-MS spectrum of compound 6i,7i. 105
Figure S81: HR-MS spectrum of compound 6j,7j. 106
Figure S82: HR-MS spectrum of compound 8a,9a. 107
Figure S83: HR-MS spectrum of compound 8b,9b. 108
Figure S84: HR-MS spectrum of compound 8c,9c. 109
Figure S85: HR-MS spectrum of compound 8d,9d. 110
Figure S86: HR-MS spectrum of compound 8e,9e. 111
Figure S87: HR-MS spectrum of compound 8f,9f. 112
Figure S88: HR-MS spectrum of compound 8g,9g. 113
Figure S89: HR-MS spectrum of compound 8h,9h. 114

Figure S90: HR-MS spectrum of compound 8i,9i. 115
Figure S91: HR-MS spectrum of compound 8j,9j. 116
7. Molecular modelling 117

1. General Information

All spectral data were procured using the following instruments: Fourier-transform Infrared (FTIR) spectra were obtained using a PerkinElmer 2000 FTIR Spectrum spectrometer at wavenumbers ranging from 4000 to 600 cm^{-1}. Nuclear magnetic resonance (NMR) spectra were recorded on a Bruker AVN 500 MHz spectrometer (Bruker Bioscience, Billerica, MA, USA). Data were analysed with the Top Spin 3.6.1 software package. Chemical shifts are given in ppm and are internally calibrated using the residual $CHCl_3$ solvent peak in $CDCl_3$ (^1H, δ 7.26), the $CDCl_3$ solvent signal (^{13}C, δ 77.0) or the tetramethylsilane (TMS) signal (^1H or ^{13}C, δ 0.00 ppm). Coupling constants are given in Hz and are rounded to the closest multiple of 0.5. Mass spectra were recorded with a Waters Xevo QTOF MS spectrometer. Melting points were measured with a calibrated Stuart SMP10 digital melting point apparatus (Stuart Scientific Bibby Sterilin Ltd., UK) by an open capillary method.

Unless otherwise noted, all chemicals and materials were purchased from commercial suppliers and used without further purification. Tetrahydrofuran (THF) (QRëC, Grade AR) was freshly distilled from sodium benzophenone ketyl. Dimethylformamide (DMF) (QRëC, Grade AR) was dried over 4Å molecular sieves (Sigma-Aldrich, St. Louis, MO, USA) prior to use. Column chromatography was performed using Merck silica gel (0.040–0.063 mm). Thin Layer Chromatography (TLC) was done with silica gel-coated aluminium sheets (silica gel 60 F_{254}).

2. Preparation of Amide Precursors 3a−3j

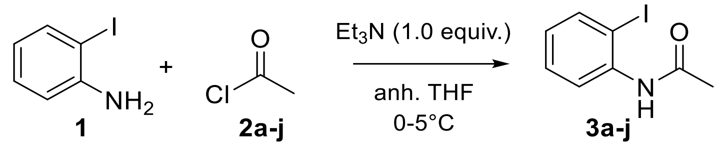

General Procedure A.[1] A solution of 2-iodoaniline **1** (1.00 equiv.) and Et_3N (1.00 equiv.) in dry THF (1.6-2.0 mL per mmol of **1**) was cooled to 0–5 °C. The requisite acyl chloride **2** (1.00 equiv.) was then added dropwise, with stirring. The cold bath was removed and the mixture was stirred vigorously overnight at room temperature. The white precipitate of Et_3N.HCl was filtered off and rinsed with THF (3 × 5 ml). All the organic fractions were combined and concentrated under reduced pressure to afford the crude amide product **3**, which was then purified by recrystallisation from *n*-hexane/chloroform.

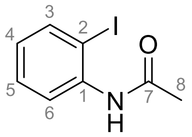

3a

Following general procedure A with acetyl chloride **2a** (2.56 mL, 36.0 mmol), *N*-(2-iodophenyl)acetamide **3a** was obtained as a white solid (7.09 g, 91%).

N-(2-Iodophenyl)acetamide **3a**. *Mp* 110-111 °C; IR (neat) υ: 3271 (w, NH), 3028 (w), 1658 (m, C=O), 1525 (s), 1290 (s), 748 (s) cm^{-1}; ^{1}H NMR ($CDCl_3$, 500 MHz) *δ:* 8.21 (d, *J* = 7.7 Hz, H-6, 1H), 7.78 (d, *J* = 7.8 Hz, H-3, 1H), 7.41 (br s, NH, 1H), 7.35 (t, *J* = 7.7 Hz, H-5, 1H), 6.85 (t, *J* = 7.4 Hz, H-4, 1H), 2.24 (s, H-8, 3H); ^{13}C NMR ($CDCl_3$, 125.8 MHz) *δ:* 168.2 (C-7), 138.8 (C-3), 138.2 (C-1), 129.3 (C-5), 126.0 (C-4), 122.0 (C-6), 89.9 (C-2), 24.8 (C-8). Analytical data is in accordance with literature data.[1,2]

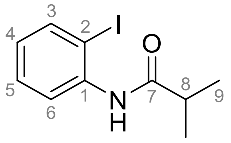

3b

Following general procedure A with isobutyryl chloride **2b** (3.77 mL, 36.0 mmol), *N*-(2-iodophenyl)isobutyramide **3b** was obtained as a white solid (7.83 g, 90%).

[1] Procedure adapted from: Ladziata, U., Koposov, A. Y., Lo, K. Y., Willging, J., Nemykin, V. N., and Zhdankin, V. V. Synthesis, structure, and chemoselective reactivity of *N*-(2-iodylphenyl)acylamides: Hypervalent iodine reagents bearing a pseudo-six-membered ring scaffold. *Angew. Chem. Int. Ed.*, **2005**, 44, 7127-7131 (supporting information).

[2] Azmi, M. N., Din, M. F. M., Kee, C. H., Suhaimi, M., Ping, A. K., Ahmad, K., Nafiah, M. A., Thomas, N. F., Mohamad, K. and Hoong, L. K. Design, synthesis and cytotoxic evaluation of *o*-carboxamido stilbene analogues. *Int. J. Mol. Sci.*, **2013**, 14, 23369-23389.

N-(2-Iodophenyl)isobutyramide **3b**. *Mp* 115-117 °C; IR (neat) υ: 3261 (w, NH), 2966 (w), 2870 (w), 1658 (m, C=O), 1525 (s), 1433 (m), 1357 (w), 1281 (m), 1161 (w), 746 (s) cm^{-1}; ^{1}H NMR ($CDCl_3$, 500 MHz) δ: 8.26 (d, J = 8.1 Hz, H-6, 1H), 7.78 (d, J = 8.0 Hz, H-3, 1H), 7.52 (br s, NH, 1H), 7.34 (t, J = 7.8 Hz, H-5, 1H), 6.84 (t, J = 7.6 Hz, H-4, 1H), 2.61 (sept, J = 6.9 Hz, H-8, 1H), 1.31 (d, J = 6.9 Hz, H-9, 6H); ^{13}C NMR ($CDCl_3$, 125.8 MHz) δ: 175.1 (C-7), 138.7 (C-3), 138.2 (C-1), 129.3 (C-5), 125.8 (C-4), 121.9 (C-6), 89.9 (C-2), 37.0 (C-8), 19.6 (C-9). Analytical data is in accordance with literature data.[1,2]

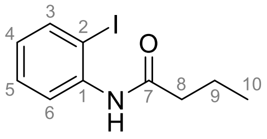

3c

Following general procedure A with butyryl chloride **2c** (3.74 mL, 36.0 mmol), *N*-(2-iodophenyl)butyramide **3c** was obtained as a white solid (6.34 g, 73%).

N-(2-Iodophenyl)butyramide **3c**. *Mp* 83-84 °C; IR (neat) υ: 3270 (w, NH), 3027 (w), 2961 (w), 1655 (s, C=O), 1524 (s), 1432 (s), 1287 (m), 1194 (m), 751 (s) cm^{-1}; ^{1}H NMR ($CDCl_3$, 500 MHz) δ: 8.24 (d, J = 7.8 Hz, H-6, 1H), 7.77 (d, J = 7.9 Hz, H-3, 1H), 7.44 (br s, NH, 1H), 7.34 (t, J = 7.8 Hz, H-5, 1H), 6.84 (t, J = 7.2 Hz, H-4, 1H), 2.42 (t, J = 7.4 Hz, H-8, 2H), 1.80 (sext, J = 7.4 Hz, H-9, 2H), 1.04 (t, J = 7.4 Hz, H-10, 3H); ^{13}C NMR ($CDCl_3$, 125.8 MHz) δ: 171.2 (C-7), 138.7 (C-3), 138.2 (C-1), 129.3 (C-5), 125.8 (C-4), 122.0 (C-6), 89.9 (C-2), 39.9 (C-8), 19.0 (C-9), 13.7 (C-10). Analytical data is in accordance with literature data.[1,2]

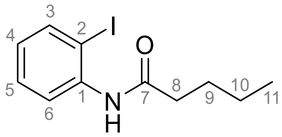

3d

Following general procedure A with pentanoyl chloride **2d** (4.27 mL, 36.0 mmol), *N*-(2-iodophenyl)pentanamide **3d** was obtained as a white solid (6.92 g, 76%).

N-(2-Iodophenyl)pentanamide **3d**. *Mp* 80–81 °C; IR (neat) υ: 3269 (m, NH), 2958 (m), 2928 (w), 2870 (w), 1653 (s, C=O), 1521 (s), 1432 (s), 1283 (s), 1184 (m), 752 (m) cm^{-1}; ^{1}H NMR ($CDCl_3$, 500 MHz) δ: 8.24 (d, J = 7.7 Hz, H-6, 1H), 7.77 (d, J = 7.8 Hz, H-3, 1H), 7.44 (br s, NH, 1H), 7.34 (t, J = 7.4 Hz, H-5, 1H), 6.84 (t, J = 7.4 Hz, H-4, 1H), 2.44 (t, J = 7.6 Hz, H-8, 2H), 1.75 (quint, J = 7.5 Hz, H-9, 2H), 1.44 (sext, J = 7.6 Hz, H-10, 2H), 0.97 (t, J = 7.4 Hz, H-11, 3H); ^{13}C NMR ($CDCl_3$, 125.8 MHz) δ: 171.4 (C-7), 138.7 (C-3), 138.2 (C-1), 129.3 (C-5), 125.8 (C-4), 121.9 (C-6), 89.8 (C-2), 37.7 (C-8), 27.6 (C-9), 22.3 (C-10), 13.8 (C-11). HRMS (+ESI) $[M + H]^+$: 304.0186, $C_{11}H_{15}INO$ requires 304.0198.

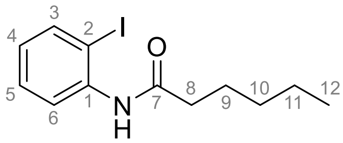

3e

Following general procedure A with hexanoyl chloride **2e** (5.03 mL, 36.0 mmol), *N*-(2-iodophenyl)hexanamide **3e** was obtained as a white solid (6.17 g, 65%).

N-(2-Iodophenyl)hexanamide **3e**. *Mp* 83–85 °C; IR (neat) υ: 3269 (m, NH), 2951 (m), 2930 (m), 2868 (w), 1661 (s, C=O), 1519 (s), 1432 (m), 1283 (m), 1182 (m), 757 (m) cm^{-1}; ^{1}H NMR ($CDCl_3$, 500 MHz) δ: 8.25 (d, *J* = 7.7 Hz, H-6, 1H), 7.78 (d, *J* = 7.9 Hz, H-3, 1H), 7.44 (br s, NH, 1H), 7.34 (t, *J* = 7.8 Hz, H-5, 1H), 6.84 (t, *J* = 7.4 Hz, H-4, 1H), 2.43 (t, *J* = 7.5 Hz, H-8, 2H), 1.77 (quint, *J* = 7.4 Hz, H-9, 2H), 1.36-1.43 (m, H-10, H-11, 4H), 0.93 (t, *J* = 7.0 Hz, H-12, 3H); ^{13}C NMR ($CDCl_3$, 125.8 MHz) δ: 171.4 (C-7), 138.7 (C-3), 138.2 (C-1), 129.2 (C-5), 125.8 (C-4), 121.9 (C-6), 89.8 (C-2), 38.0 (C-8), 31.3 (C-10), 25.3 (C-9), 22.4 (C-11), 13.9 (C-12). Analytical data is in accordance with literature data.[3]

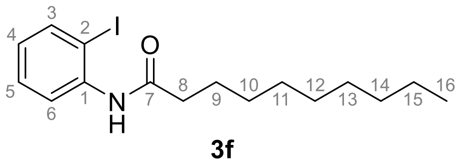

3f

Following general procedure A with decanoyl chloride **2f** (7.47 mL, 36.0 mmol), *N*-(2-iodophenyl)decanamide **3f** was obtained as a brown solid (9.32 g, 83%).

N-(2-Iodophenyl)decanamide **3f**. *Mp* 78-82 °C; IR (neat) υ: 3254 (m, NH), 3031 (w), 2957 (m), 2915 (s), 2849 (m), 1663 (s, C=O), 1529 (s), 1433 (m), 1289 (m), 1181 (m), 729 (m) cm^{-1}; ^{1}H NMR ($CDCl_3$, 500 MHz) δ: 8.23 (d, *J* = 7.7 Hz, H-6, 1H), 7.77 (d, *J* = 7.9 Hz, H-3, 1H), 7.44 (br s, NH, 1H), 7.34 (t, *J* = 7.8 Hz, H-5, 1H), 6.84 (t, *J* = 7.5 Hz, H-4, 1H), 2.43 (t, *J* = 7.4 Hz, H-8, 2H), 1.76 (quint, *J* = 7.4 Hz, H-9, 2H), 1.27-1.30 (m, H-10, H-11, H-12, H-13, H-14, H-15, 12H), 0.88 (t, *J* = 6.8 Hz, H-16, 3H); ^{13}C NMR ($CDCl_3$, 125.8 MHz) δ: 171.4 (C-7), 138.7 (C-3), 138.2 (C-1), 129.3 (C-5), 125.9 (C-4), 122.0 (C-6), 89.8 (C-2), 38.0 (C-8), 31.8 (C-14), 29.4 (C-13, C-12), 29.3 (C-10), 29.2 (C-11), 25.6 (C-9), 22.6 (C-15), 14.1 (C-16). HRMS (+ESI) $[M + H]^+$: 374.0970, $C_{16}H_{25}INO$ requires 374.0981.

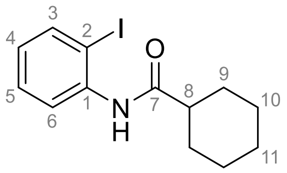

3g

Following general procedure A with cyclohexanecarbonyl chloride **2g** (1.93 mL, 14.0 mmol), *N*-(2-iodophenyl)cyclohexanecarboxamide **3g** was obtained as a light brown solid (2.97 g, 76%).

N-(2-Iodophenyl)cyclohexanecarboxamide **3g**. *Mp* 131–140 °C; IR (neat) υ: 3266 (m, NH), 2925 (m), 2851 (m), 1655 (s, C=O), 1524 (s), 1433 (m), 1280 (m), 1197 (m), 1017 (m), 749 (s) cm^{-1}; ^{1}H NMR ($CDCl_3$, 500 MHz) δ: 8.25 (dd, *J* = 8.2, 1.2 Hz, H-6, 1H), 7.76 (dd, *J* = 7.9, 1.2 Hz, H-3, 1H), 7.52 (br s, NH, 1H), 7.34 (td, *J* = 8.3, 1.2 Hz, H-5, 1H), 6.83 (td, *J* = 7.6, 1.2 Hz, H-4, 1H), 2.29 – 2.35 (m, H-8, 1H), 1.25-2.07 (m, H-9, H-10, H-11, 10H); ^{13}C NMR ($CDCl_3$, 125.8 MHz) δ: 174.3 (C-7), 138.7 (C-3), 138.2 (C-1), 129.2 (C-5), 125.7 (C-4), 121.9 (C-6), 88.5 (C-2), 46.6 (C-8), 29.7 (C-9), 25.69 (C-11), 25.67 (C-10). Analytical data is in accordance with literature data.[1,2]

[3] Curran, D. P., Yu, H. and Liu, H. Amide-based protecting/radical translocating (PRT) groups. Generation of radicals adjacent to carbonyls by 1, 5-hydrogen transfer reactions of o-iodoanilides. *Tetrahedron*, **1994**, 50, 7343-7366.

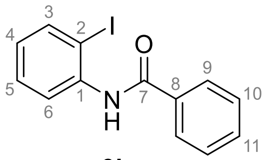

3h

Following general procedure A with benzoyl chloride **2h** (4.18 mL, 36.0 mmol), *N*-(2-iodophenyl)benzamide **3h** was obtained as a white solid (8.54 g, 89%).

N-(2-Iodophenyl)benzamide **3h**. *Mp* 136-139 °C; IR (neat) υ: 3209 (m, NH), 3090 (w), 1646 (s, C=O), 1513 (s), 1298 (s), 1161 (w), 745 (s) cm^{-1}; ^{1}H NMR (CDCl$_3$, 500 MHz) *δ:* 8.47 (dd, *J* = 8.2, 1.3 Hz, H-6, 1H), 8.30 (br s, NH, 1H), 7.98 (d, *J* = 7.2 Hz, H-9, 2H), 7.83 (dd, *J* = 7.9, 1.3 Hz, H-3, 1H), 7.59 (t, *J* = 7.3 Hz, H-11, 1H), 7.53 (t, *J* = 7.4 Hz, H-10, 2H), 7.41 (td, *J* = 7.2, 1.5 Hz, H-5, 1H), 6.89 (td, *J* = 7.6, 1.5 Hz, H-4, 1H); ^{13}C NMR (CDCl$_3$, 125.8 MHz) *δ:* 165.3 (C-7), 138.8 (C-3), 138.2 (C-1), 134.5 (C-8), 132.2 (C-11), 129.4 (C-5), 129.0 (C-10), 127.2 (C-9), 126.0 (C-4), 121.7 (C-6), 90.2 (C-2). Analytical data is in accordance with literature data.[2,4]

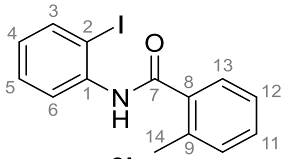

3i

Following general procedure A with 2-methylbenzoyl chloride **2i** (4.70 mL, 36.0 mmol), *N*-(2-iodophenyl)-2-methylbenzamide **3i** was obtained as a white solid (6.65 g, 66%).

N-(2-Iodophenyl)-2-methylbenzamide **3i**. *Mp* 103-106 °C; IR (neat) υ: 3255 (w, NH), 3020 (w), 2975 (w), 1650 (m, C=O), 1515 (m), 1302 (m), 1144 (w), 750 (s) cm^{-1}; ^{1}H NMR (CDCl$_3$, 500 MHz) *δ*: 8.42 (br s, NH, 1H), 7.82 (dd, *J* = 8.0, 1.2 Hz, H-3, H-6, 2H), 7.61 (d, *J* = 7.6 Hz, H-13, 1H), 7.29-7.42 (m, H-4, H-5, H-10, H-11, 4H), 6.90 (td, *J* = 7.7, 1.4 Hz, H-12, 1H), 2.57 (s, H-14, 3H); ^{13}C NMR (CDCl$_3$, 125.8 MHz) *δ*: 167.9 (C-7), 138.9 (C-3), 138.4 (C-1), 137.0 (C-8), 135.8 (C-9), 131.5 (C-11), 130.6 (C-10), 129.3 (C-12), 126.8 (C-5), 126.2 (C-13), 126.1 (C-4), 122.0 (C-6), 90.2 (C-2), 20.2 (C-14). Analytical data is in accordance with literature data.[4]

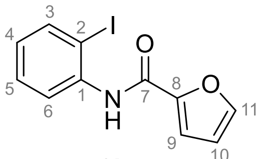

3j

Following general procedure A with 2-furoyl chloride **2j** (2.30 mL, 23.0 mmol), *N*-(2-iodophenyl)furan-2-carboxamide **3j** was obtained as a white solid (4.70 g, 77%).

N-(2-Iodophenyl)furan-2-carboxamide **3j**. *Mp* 81–82 °C; IR (neat) υ: 3364 (m, NH), 3134 (w), 1680 (s, C=O), 1582 (s), 1518 (s), 1300 (s), 1159 (m), 746 (s) cm^{-1}; ^{1}H NMR (CDCl$_3$, 500 MHz) *δ*: 8.54 (br s, NH, 1H), 8.40 (dd, *J* = 8.3, 1.4 Hz, H-6, 1H), 7.82 (dd, *J* = 8.0, 1.3 Hz, H-3, 1H), 7.58 (d, *J* = 1.4 Hz, H-11, 1H), 7.38 (td, *J* = 7.8, 1.3 Hz, H-5, 1H), 7.28 (d, *J* = 3.5 Hz, H-10, 1H), 6.88 (td, *J* = 7.6, 1.4 Hz, H-4, 1H), 6.58 (dd, *J* = 3.5, 1.4 Hz, H-9, 1H); ^{13}C NMR (CDCl$_3$, 125.8 MHz) *δ*: 156.0 (C-7), 147.7 (C-8), 144.7 (C-11), 139.0 (C-3), 137.9 (C-1), 129.3 (C-5), 126.0 (C-4),

[4] Prasad, D. J. C. and Sekar, G. Cu-catalyzed in situ generation of thiol using xanthate as a thiol surrogate for the one-pot synthesis of benzothiazoles and benzothiophenes. *Org. Biomol. Chem.*, 2013, **11**, 1659-1665 (supporting information).

121.6 (C-6), 115.7 (C-9), 112.7 (C-10), 89.7 (C-2). Analytical data is in accordance with literature data.[2]

3. Synthesis of substituted *N*-(2-cinnamylphenyl) amide compounds

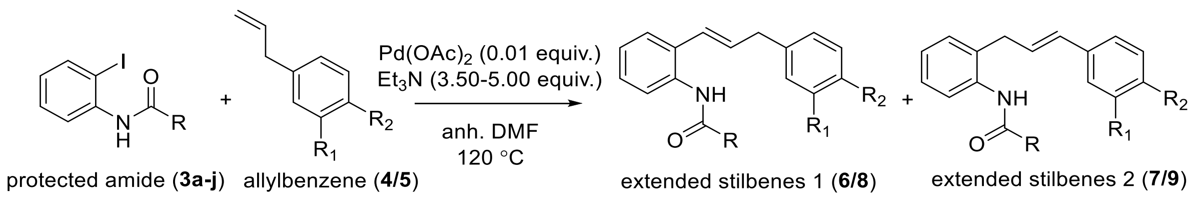

General Procedure B. In a dry three-necked flask equipped with a thermometer and a condenser, a solution of *N*-(2-iodophenyl) amide **3** (1.00 equiv.) in dry DMF (4.0 mL per mmol of substrate **3**) was heated up to 120 °C and stirred for 20 minutes under nitrogen. Palladium(II) acetate (1.00% equiv.), triethylamine (3.50-5.00 equiv.) and the requisite allylbenzene derivative **4** or **5** (1.60 equiv.) were then added successively. The reaction mixture was stirred at 120 °C until TLC analysis indicated complete consumption of **3** (3-6 h). After cooling, saturated NH_4Cl aqueous solution (6.0 mL per mmol of starting **3**) was added and the mixture was extracted with EtOAc (three times, with portions of 2.0 mL per mmol of starting **3**).-The combined organic fractions were washed with H_2O (6.0 mL per mmol of starting **3**), dried over anhydrous sodium sulphate, filtered and concentrated under reduced pressure. The ratio of product isomers was determined by ^{1}H NMR spectroscopy of the crude product. Purification by gradient elution column chromatography (*n*-hexane/ethyl acetate, 95:5 to 50:50) then yielded the desired Heck reaction products.

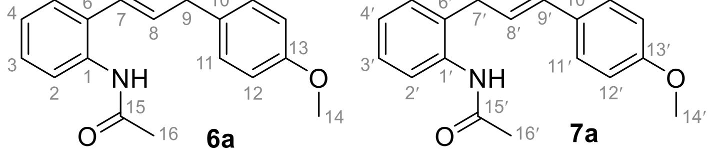

6a **7a**

Following general procedure B with *N*-(2-iodophenyl)acetamide **3a** (1.31 g, 5.00 mmol), 1-allyl-4-methoxybenzene **4** (1.23 ml, 8.00 mmol), $Pd(OAc)_2$ (11.2 mg, 50.0 μmol) and Et_3N (3.48 mL, 25.0 mmol), a 51:49 mixture of (*E*)-*N*-(2-(3-(4-methoxyphenyl)prop-1-en-1-yl)phenyl) acetamide **6a** and (*E*)-*N*-(2-(3-(4-

methoxyphenyl)allyl)phenyl)acetamide **7a** was produced (1.06 g, 75%). Further partial separation by isocratic elution column chromatography (chloroform/dichloromethane, 60:40) then yielded **7a** (0.16 g, 11%).

51:49 mixture of **6a** and **7a**. White solid. $R_f \approx 0.23$, [UV-active, EtOAc/Hexane 40%]. IR (neat) υ: 3286 (m, NH), 3004 (w), 2833 (w), 1653 (s, C=O), 1510 (s), 1453 (m), 1371 (m), 1273 (m), 1242 (s), 1179 (m), 1035 (m, C–O), 821 (m), 753 (s) cm^{-1}; HRMS (+ESI) $[M + H]^+$: 282.1475, $C_{18}H_{20}NO_2$ requires 282.1494.

(*E*)-*N*-(2-(3-(4-Methoxyphenyl)prop-1-en-1-yl)phenyl)acetamide **6a**. ^1H NMR ($CDCl_3$, 500 MHz) *δ:* 7.76 (d, *J* = 8.0 Hz, H-2, 1H), 7.41 (br s, NH, 1H), 7.38 (d, *J* = 8.0 Hz, H-5, 1H), 7.22 (dd, *J* = 8.0, 7.5 Hz, H-3, 1H), 7.16 (br d, *J* = 8.5 Hz, H-11, 2H), 7.11 (dd, *J* = 8.0, 7.5 Hz, H-4, 1H), 6.87 (br d, *J* = 8.5 Hz, H-12, 2H), 6.45 (d, *J* = 15.5 Hz, H-7, 1H), 6.25 (dt, *J* = 15.5, 7.0 Hz, H-8, 1H), 3.80 (s, H-14, 3H), 3.51 (d, *J* = 7.0 Hz, H-9, 2H), 2.14 (s, H-16, 3H); ^{13}C NMR ($CDCl_3$, 125.8 MHz) *δ:* 168.4 (C-15), 158.1 (C-13), 134.1 (C-1), 133.9 (C-8), 131.7 (C-10), 130.2 (C-6), 129.5 (C-11), 127.8 (C-3), 126.9 (C-5), 125.6 (C-7), 125.3 (C-4), 123.8 (C-2), 114.00 (C-12), 55.2 (C-14), 38.6 (C-9), 24.2 (C-16).

(*E*)-*N*-(2-(3-(4-Methoxyphenyl)allyl)phenyl)acetamide **7a**. ^1H NMR ($CDCl_3$, 500 MHz) *δ:* 7.82 (d, *J* = 8.0 Hz, H-2′, 1H), 7.28 (br d, *J* = 8.5 Hz, H-11′, 2H), 7.27 (dd, *J* = 8.0, 7.5 Hz, H-3′, 1H), 7.26-7.29 (m, NH, 1H), 7.23 (d, *J* = 8.0 Hz, H-5′, 1H), 7.13 (dd, *J* = 8.0, 7.5 Hz, H-4′, 1H), 6.84 (br d, *J* = 8.5 Hz, H-12′, 2H), 6.40 (d, *J* = 16.0 Hz, H-9′, 1H), 6.17 (dt, *J* = 16.0, 6.5 Hz, H-8′, 1H), 3.80 (s, H-14′, 3H), 3.51 (d, *J* = 6.5 Hz, H-7′, 2H), 2.11 (s, H-16′, 3H); ^{13}C NMR ($CDCl_3$, 125.8 MHz) *δ:* 168.3 (C-15′), 159.2 (C-13′), 136.0 (C-1′), 131.1 (C-9′), 130.6 (C-6′), 130.1 (C-5′), 129.4 (C-10′), 127.4 (C-3′), 127.3 (C-11′), 125.4 (C-8′), 125.3 (C-4′), 123.9 (C-2′), 113.96 (C-12′), 55.2 (C-14′), 36.0 (C-7′), 24.3 (C-16′).

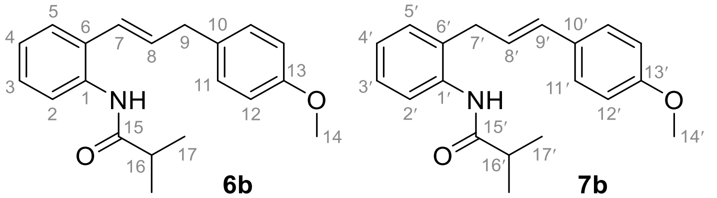

6b **7b**

Following general procedure B with *N*-(2-iodophenyl)isobutyramide **3b** (1.45 g, 5.00 mmol), 1-allyl-4-methoxybenzene **4** (1.23 ml, 8.00 mmol), $Pd(OAc)_2$ (11.2 mg, 50.0 μmol) and Et_3N (3.48 mL, 25.0 mmol), a 54:46 mixture of (*E*)-*N*-(2-(3-(4-methoxyphenyl)prop-1-en-1-yl)phenyl) isobutyramide **6b** and (*E*)-*N*-(2-(3-(4-methoxyphenyl)allyl)phenyl)isobutyramide **7b** was produced (1.24 g, 80%). Further partial separation by isocratic elution column chromatography (chloroform/dichloromethane, 60:40) then yielded **7b** (0.31 g, 20%).

54:46 mixture of **6b** and **7b**. White solid. R_f ≈ 0.31, [UV-active, EtOAc/Hexane 20%]. IR (neat) υ: 3268 (w, NH), 3032 (w), 2969 (w), 1651 (m, C=O), 1511 (s), 1452 (m), 1382 (w), 1287 (w), 1178 (w), 1033 (m, C–O), 834 (w), 746 (s) cm^{-1}; HRMS (+ESI) [M + H]$^+$: 310.1823, $C_{20}H_{24}NO_2$ requires 310.1807.

(*E*)-*N*-(2-(3-(4-Methoxyphenyl)prop-1-en-1-yl)phenyl)isobutyramide **6b**. ^{1}H NMR ($CDCl_3$, 500 MHz) *δ:* 7.86 (d, *J* = 8.0 Hz, H-2, 1H), 7.38 (br s, NH, 1H), 7.35 (d, *J* = 8.0 Hz, H-5, 1H), 7.22 (td, *J* = 8.0, 1.5 Hz, H-3, 1H), 7.15 (d, *J* = 8.5 Hz, H-11, 2H), 7.08-7.13 (m, H-4, 1H), 6.87 (d, *J* = 8.5 Hz, H-12, 2H), 6.40 (d, *J* = 15.0 Hz, H-7, 1H), 6.26 (dt, *J* = 15.0, 6.6 Hz, H-8, 1H), 3.80 (s, H-14, 3H), 3.51 (d, *J* = 6.6 Hz, H-9, 2H), 2.47 (sept, *J* = 6.8 Hz, H-16, 1H), 1.21 (d, *J* = 6.8 Hz, H-17, 6H); ^{13}C NMR ($CDCl_3$, 125.8 MHz) *δ:* 175.0 (C-15), 158.2 (C-13), 134.5 (C-8), 134.2 (C-1), 131.5 (C-10), 129.8 (C-6), 129.6 (C-11), 127.8 (C-3), 127.1 (C-5), 125.5 (C-7), 124.9 (C-4), 123.2 (C-2), 114.0 (C-12), 55.3 (C-14), 38.6 (C-9), 36.6 (C-16), 19.6 (C-17).

(*E*)-*N*-(2-(3-(4-Methoxyphenyl)allyl)phenyl)isobutyramide **7b**. ^{1}H NMR ($CDCl_3$, 500 MHz) *δ:* 7.94 (d, *J* = 7.5 Hz, H-2′, 1H), 7.38 (br s, NH, 1H), 7.24-7.27 (m, H-3′, H-11′, 3H), 7.23 (d, *J* = 7.5 Hz, H-5′, 1H), 7.08-7.13 (m, H-4′, 1H), 6.84 (d, *J* = 8.7 Hz, H-12′, 2H), 6.38 (d, *J* = 15.8 Hz, H-9′, 1H), 6.18 (dt, *J* = 15.8, 6.5 Hz, H-8′, 1H), 3.80 (s, H-14′, 3H), 3.52 (d, *J* = 6.5 Hz, H-7′, 2H), 2.47 (sept, *J* = 6.8 Hz, H-16′, 1H), 1.20 (d, *J* = 6.8 Hz, H-17′, 6H); ^{13}C NMR ($CDCl_3$, 125.8 MHz) *δ:* 175.1 (C-15′), 159.2 (C-13′), 135.6 (C-1′), 131.2 (C-9′), 130.2 (C-5′), 130.1 (C-6′), 129.4 (C-10′), 127.5 (C-3′), 127.3 (C-11′), 125.3 (C-8′), 125.0 (C-4′), 123.3 (C-2′), 114.0 (C-12′), 55.3 (C-14′), 36.5 (C-16′), 36.1 (C-7′), 19.6 (C-17′).

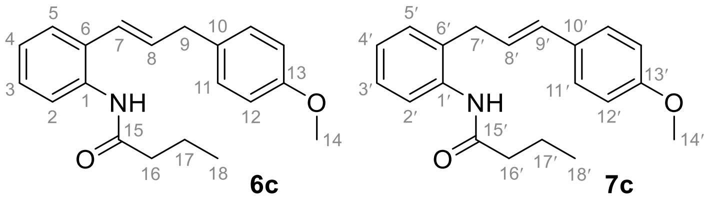

6c **7c**

Following general procedure B with *N*-(2-iodophenyl)butyramide **3c** (145 g, 5.00 mmol), 1-allyl-4-methoxybenzene **4** (1.23 ml, 8.00 mmol), $Pd(OAc)_2$ (11.2 mg, 50.0 μmol) and Et_3N (3.48 mL, 25.0 mmol), a 48:52 mixture of (*E*)-*N*-(2-(3-(4-methoxyphenyl)prop-1-en-1-yl)phenyl) butyramide **6c** and (*E*)-*N*-(2-(3-(4-methoxyphenyl)allyl)phenyl)butyramide **7c** was produced (1.33 g, 86%). Further partial separation by isocratic elution column chromatography (chloroform/dichloromethane, 60:40) then yielded **7c** (0.22 g, 14%).

48:52 mixture of **6c** and **7c**. White solid. R_f ≈ 0.22, [UV-active, EtOAc/Hexane 20%]. IR (neat) υ: 3290 (w, NH), 3005 (w), 2959 (w), 1648 (s, C=O), 1511 (s), 1453 (m), 1381 (w), 1291 (m), 1175 (m), 1159, 1037 (m, C–O), 829 (w), 747 (s) cm^{-1}; HRMS (+ESI) [M + H]$^+$: 310.1816, $C_{20}H_{24}NO_2$ requires 310.1807.

(*E*)-*N*-(2-(3-(4-Methoxyphenyl)prop-1-en-1-yl)phenyl)butyramide **6c**. ^{1}H NMR ($CDCl_3$, 500 MHz) δ: 7.84 (d, J = 7.6 Hz, H-2, 1H), 7.36 (d, J = 7.6 Hz, H-5, 1H), 7.34 (br s, NH, 1H), 7.16 (d, J = 8.2 Hz, H-11, 2H), 7.10-7.12 (m, H-3, H-4, 2H), 6.87 (d, J = 8.2 Hz, H-12, 2H), 6.41 (d, J = 15.3 Hz, H-7, 1H), 6.25 (dt, J = 15.3, 6.5 Hz, H-8, 1H), 3.80 (s, H-14, 3H), 3.52 (d, J = 6.5 Hz, H-9, 2H), 2.30 (t, J = 7.4 Hz, H-16, 2H), 1.67-1.72 (m, H-17, 2H), 0.96 (t, J = 7.4 Hz, H-18, 3H); ^{13}C NMR ($CDCl_3$, 125.8 MHz) δ: 171.2 (C-15), 158.2 (C-13), 134.4 (C-8), 134.2 (C-1), 131.6 (C-10), 129.8 (C-6), 129.6 (C-11), 127.8 (C-3), 127.1 (C-5), 125.5 (C-7), 125.1 (C-4), 123.4 (C-2), 114.0 (C-12), 55.3 (C-14), 39.5 (C-16), 38.6 (C-9), 18.7 (C-17), 13.5 (C-18).

(*E*)-*N*-(2-(3-(4-Methoxyphenyl)allyl)phenyl)butyramide **7c**. ^{1}H NMR ($CDCl_3$, 500 MHz) δ: 7.91 (d, J = 8.0 Hz, H-2′, 1H), 7.34 (br s, NH, 1H), 7.28 (br dd, J = 8.0, 7.5 Hz, H-3′, 1H), 7.27 (br d, J = 8.5 Hz H-11′, 2H), 7.23 (d, J = 7.5 Hz, H-5′, 1H), 7.12 (t, J = 7.5 Hz, H-4′, 1H), 6.85 (br d, J = 8.5 Hz H-12′, 2H), 6.41 (br d, J = 16.0 Hz, H-9′, 1H), 6.18 (dt, J = 16.0, 6.0 Hz, H-8′, 1H), 3.80 (s, H-14′, 3H), 3.52 (br d, J = 6.0 Hz, H-7′, 2H), 2.28 (t, J = 7.5 Hz, H-16′, 2H), 1.70 (sext, J = 7.5 Hz, H-17′, 2H), 0.95 (t, J = 7.5 Hz, H-18′, 3H); ^{13}C NMR ($CDCl_3$, 125.8 MHz) δ: 171.2 (C-15′), 159.2 (C-13′), 136.2 (C-1′), 131.2 (C-9′), 130.2 (C-5′), 130.0 (C-6′), 129.4 (C-10′), 127.5 (C-3′), 127.3 (C-11′), 125.3 (C-8′), 125.0 (C-4′), 123.5 (C-2′), 114.0 (C-12′), 55.3 (C-14′), 39.7 (C-16′), 36.2 (C-7′), 19.2 (C-17′), 13.7 (C-18′).

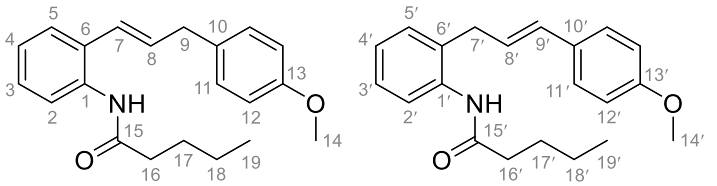

6d **7d**

Following general procedure B with *N*-(2-iodophenyl)pentanamide **3d** (1.52 g, 5.00 mmol), 1-allyl-4-methoxybenzene **4** (1.23 ml, 8.00 mmol), $Pd(OAc)_2$ (11.2 mg, 50.0 μmol) and Et_3N (3.48 mL, 25.0 mmol), a 47:53 mixture of (*E*)-*N*-(2-(3-(4-methoxyphenyl)prop-1-en-1-yl)phenyl)pentanamide **6d** and (*E*)-*N*-(2-(3-(4-methoxyphenyl)allyl)phenyl)pentanamide **7d** was produced (1.48 g, 91%).

47:53 mixture of **6d** and **7d**. White solid. $R_f \approx 0.41$, [UV-active, EtOAc/Hexane 20%]. IR (neat) υ: 3262 (w, NH), 3002 (w), 2959 (w), 1652 (s, C=O), 1509 (s), 1450 (m), 1380 (w), 1292 (w), 1174 (m), 1037 (m, C–O), 813 (w), 747 (s) cm^{-1}; HRMS (+ESI) [M + H]$^{+}$: 324.1958, $C_{21}H_{26}NO_2$ requires 324.1964.

(*E*)-*N*-(2-(3-(4-Methoxyphenyl)prop-1-en-1-yl)phenyl)pentanamide **6d**. ^{1}H NMR ($CDCl_3$, 500 MHz) δ: 7.88 (d, J = 7.8 Hz, H-2, 1H), 7.38 (br s, NH, 1H), 7.37 (d, J = 7.8 Hz, H-5, 1H), 7.27–7.31 (m, H-3, 1H), 7.18 (d, J = 8.5 Hz, H-11, 2H), 7.10–7.16 (m, H-4, 1H), 6.89 (d, J = 8.5 Hz, H-12, 2H), 6.44 (d, J = 15.2 Hz, H-7, 1H), 6.27 (dt, J = 15.2, 6.3 Hz, H-8, 1H), 3.82 (s, H-14, 3H), 3.54 (d, J = 6.3 Hz, H-9, 2H), 2.34 (t, J = 7.5 Hz, H-16, 2H), 1.64–1.73 (m, H-17, 2H), 1.40–1.46 (m, H-18, 2H), 0.97 (t, J

= 7.5 Hz, H-19, 3H); ^{13}C NMR ($CDCl_3$, 125.8 MHz) *δ:* 171.3 (C-15), 158.2 (C-13), 134.3 (C-8), 134.2 (C-1), 131.6 (C-10), 129.8 (C-6), 129.5 (C-11), 127.8 (C-3), 127.1 (C-5), 125.6 (C-7), 125.0 (C-4), 123.4 (C-2), 114.0 (C-12), 55.3 (C-14), 38.7 (C-9), 37.4 (C-16), 27.8 (C-17), 22.3 (C-18), 13.7 (C-19).

(*E*)-*N*-(2-(3-(4-Methoxyphenyl)allyl)phenyl)pentanamide **7d**. ^{1}H NMR ($CDCl_3$, 500 MHz) *δ*: 7.94 (d, *J* = 7.7 Hz, H-2′, 1H), 7.38 (br s, NH, 1H), 7.30 (d, *J* = 8.8 Hz, H-11′, 2H), 7.27-7.31 (m, H-3′, 1H), 7.25 (d, *J* = 7.7 Hz, H-5′, 1H), 7.10−7.16 (m, H-4′, 1H), 6.87 (d, *J* = 8.8 Hz, H-12′, 2H), 6.42 (d, *J* = 15.8 Hz, H-9′, 1H), 6.20 (dt, *J* = 15.8, 6.0 Hz, H-8′, 1H), 3.82 (s, H-14′, 3H), 3.54 (d, *J* = 6.0 Hz, H-7′, 2H), 2.32 (t, *J* = 7.5 Hz, H-16′, 2H), 1.64−1.73 (m, H-17′, 2H), 1.34−1.39 (m, H-18′, 2H), 0.90 (t, *J* = 7.5 Hz, H-19′, 3H); ^{13}C NMR ($CDCl_3$, 125.8 MHz) *δ*: 171.3 (C-15′), 159.2 (C-13′), 136.3 (C-1′), 131.2 (C-9′), 130.2 (C-5′), 129.9 (C-6′), 129.4 (C-10′), 127.5 (C-3′), 127.3 (C-11′), 125.3 (C-8′), 125.0 (C-4′), 123.3 (C-2′), 114.0 (C-12′), 55.3 (C-14′), 37.6 (C-16′), 36.2 (C-7′), 27.8 (C-17′), 22.4 (C-18′), 13.8 (C-19′).

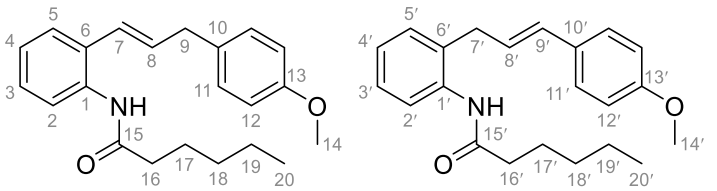

6e **7e**

Following general procedure B with *N*-(2-iodophenyl)hexanamide **3e** (1.60 g, 5.00 mmol), 1-allyl-4-methoxybenzene **4** (1.23 ml, 8.00 mmol), $Pd(OAc)_2$ (11.2 mg, 50.0 μmol) and Et_3N (3.48 mL, 25.0 mmol), a 49:51 mixture of (*E*)-*N*-(2-(3-(4-methoxyphenyl)prop-1-en-1-yl)phenyl)hexanamide **6e** and (*E*)-*N*-(2-(3-(4-methoxyphenyl)allyl)phenyl)hexanamide **7e** was produced (1.21 g, 72%).

49:51 mixture of **6e** and **7e**. White solid. $R_f \approx 0.29$, [UV-active, EtOAc/Hexane 20%]. IR (neat) υ: 3278 (w, NH), 2952 (w), 2929 (w), 1651 (s, C=O), 1510 (s), 1451 (m), 1378 (w), 1296 (m), 1176 (m), 1037 (m, C−O), 824 (w), 747 (s) cm^{-1}; HRMS (+ESI) [M + H]$^+$:338.2103, $C_{22}H_{28}NO_2$ requires 338.2120.

(*E*)-*N*-(2-(3-(4-Methoxyphenyl)prop-1-en-1-yl)phenyl)hexanamide **6e**. ^{1}H NMR ($CDCl_3$, 500 MHz) *δ:* 7.85 (d, *J* = 7.5 Hz, H-2, 1H), 7.36 (br s, NH, 1H), 7.35 (d, *J* = 7.5 Hz, H-5, 1H), 7.27−7.30 (m, H-3, 1H), 7.16 (d, *J* = 8.5 Hz, H-11, 2H), 7.09−7.13 (m, H-4, 1H), 6.87 (d, *J* = 8.5 Hz, H-12, 2H), 6.43 (d, *J* = 15.0 Hz, H-7, 1H), 6.24 (dt, *J* = 15.0, 6.8 Hz, H-8, 1H), 3.80 (s, H-14, 3H), 3.52 (d, *J* = 6.8 Hz, H-9, 2H), 2.29 (t, *J* = 7.2 Hz, H-16, 2H), 1.63−1.71 (m, H-17, 2H), 1.34−1.36 (m, H-18, 2H), 1.27−1.29 (m, H-19, 2H), 0.85 (t, *J* = 7.2 Hz, H-20, 3H); ^{13}C NMR ($CDCl_3$, 125.8 MHz) *δ:* 171.3 (C-15), 158.2 (C-13), 134.4 (C-8), 134.3 (C-1), 131.6 (C-10), 129.8 (C-6), 129.5 (C-11), 127.8 (C-3), 127.1 (C-5), 125.6 (C-7), 124.99 (C-4), 123.4 (C-2), 113.99 (C-12), 55.2 (C-14), 38.7 (C-9), 37.8 (C-16), 31.4 (C-18), 25.39 (C-17), 22.4 (C-19), 13.93 (C-20).

(*E*)-*N*-(2-(3-(4-Methoxyphenyl)allyl)phenyl)hexanamide **7e**. ^{1}H NMR ($CDCl_3$, 500 MHz) δ: 7.92 (d, *J* = 7.8 Hz, H-2′, 1H), 7.36 (br s, NH, 1H), 7.27−7.30 (m, H-3′, 1H), 7.27 (d, *J* = 8.8 Hz, H-11′, 2H), 7.22 (d, *J* = 7.8 Hz, H-5′, 1H), 7.09−7.13 (m, H-4′, 1H), 6.84 (d, *J* = 8.5 Hz, H-12′, 2H), 6.40 (d, *J* = 15.5 Hz, H-9′, 1H), 6.18 (dt, *J* = 15.5, 6.5 Hz, H-8′, 1H), 3.80 (s, H-14′, 3H), 3.52 (d, *J* = 6.5 Hz, H-7′, 2H), 2.29 (t, *J* = 7.2 Hz, H-16′, 2H), 1.63−1.71 (m, H-17′, 2H), 1.34−1.36 (m, H-18′, 2H), 1.27−1.29 (m, H-19′, 2H), 0.85 (t, *J* = 7.2 Hz, H-20′, 3H); ^{13}C NMR ($CDCl_3$, 125.8 MHz) δ: 171.3 (C-15′), 159.2 (C-13′), 136.3 (C-1′), 131.2 (C-9′), 130.3 (C-5′), 130.2 (C-6′), 129.4 (C-10′), 127.5 (C-3′), 127.3 (C-11′), 125.3 (C-8′), 125.01 (C-4′), 123.3 (C-2′), 114.02 (C-12′), 55.3 (C-14′), 37.6 (C-16′), 36.2 (C-7′), 31.4 (C-18′), 25.43 (C-17′), 22.3 (C-19′), 13.87 (C-20′).

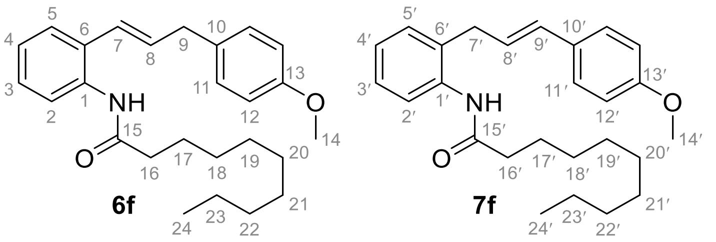

Following general procedure B with *N*-(2-iodophenyl)decanamide **3f** (1.87 g, 5.00 mmol), 1-allyl-4-methoxybenzene **4** (1.23 ml, 8.00 mmol), $Pd(OAc)_2$ (11.2 mg, 50.0 μmol) and Et_3N (3.48 mL, 25.0 mmol), a 45:55 mixture of (*E*)-*N*-(2-(3-(4-methoxyphenyl)prop-1-en-1-yl)phenyl)decanamide **6f** and (*E*)-*N*-(2-(3-(4-methoxyphenyl)allyl)phenyl)decanamide **7f** was produced (1.56 g, 79%). Further partial separation by isocratic elution column chromatography (chloroform/dichloromethane, 60:40) then yielded **7f** (0.39 g, 20%).

45:55 mixture of **6f** and **7f**. Light brown solid. $R_f \approx 0.42$, [UV-active, EtOAc/Hexane 20%]. IR (neat) υ: 3297 (w, NH), 2954 (w), 2920 (m), 1648 (m, C=O), 1509 (s), 1453 (m), 1378 (w), 1293 (m), 1174 (m), 1034 (m, C−O), 827 (w), 748 (s) cm^{-1}; HRMS (+ESI) $[M + H]^+$: 394.2741, $C_{26}H_{36}NO_2$ requires 394.2746.

(*E*)-*N*-(2-(3-(4-Methoxyphenyl)prop-1-en-1-yl)phenyl)decanamide **6f**. ^{1}H NMR ($CDCl_3$, 500 MHz) δ: 7.82 (d, *J* = 7.7 Hz, H-2, 1H), 7.42 (br s, NH, 1H), 7.36 (d, *J* = 7.7 Hz, H-5, 1H), 7.21−7.25 (m, H-3, 1H), 7.16 (d, *J* = 8.5 Hz, H-11, 2H), 7.08−7.13 (m, H-4, 1H), 6.87 (d, *J* = 8.5 Hz, H-12, 2H), 6.44 (d, *J* = 15.5 Hz, H-7, 1H), 6.24 (dt, *J* = 15.5, 6.5 Hz, H-8, 1H), 3.80 (s, H-14, 3H), 3.51 (d, *J* = 6.5 Hz, H-9, 2H), 2.27−2.33 (m, H-16, 2H), 1.62−1.70 (m, H-17, 2H), 1.15−1.28 (m, H-18, H-19, H-20, H-21, H-22, H-23, 12H), 0.89 (t, *J* = 6.9 Hz, H-24, 3H); ^{13}C NMR ($CDCl_3$, 125.8 MHz) δ: 171.4 (C-15), 158.2 (C-13), 134.3 (C-1), 134.1 (C-8), 131.6 (C-10), 130.1 (C-6), 129.5 (C-11), 127.8 (C-3), 127.0 (C-5), 125.6 (C-7), 125.0 (C-4), 123.5 (C-2), 113.97 (C-12), 55.2 (C-14), 38.6 (C-9), 37.8 (C-16), 31.81 (C-22), 29.4 (C-21), 29.3 (C-20), 29.2 (C-18, C-19), 25.75 (C-17), 22.6 (C-23), 14.0 (C-24).

(*E*)-*N*-(2-(3-(4-Methoxyphenyl)allyl)phenyl)decanamide **7f**. ^{1}H NMR ($CDCl_3$, 500 MHz) *δ:* 7.90 (d, *J* = 7.5 Hz, H-2′, 1H), 7.42 (br s, NH, 1H), 7.27 (d, *J* = 8.6 Hz, H-11′, 2H), 7.23 (d, *J* = 7.5 Hz, H-5′, 1H), 7.21−7.25 (m, H-3′, 1H), 7.08−7.13 (m, H-4′, 1H), 6.84 (d, *J* = 8.5 Hz, H-12′, 2H), 6.41 (d, *J* = 16.0 Hz, H-9′, 1H), 6.18 (dt, *J* = 16.0, 6.6 Hz, H-8′, 1H), 3.80 (s, H-14′, 3H), 3.51 (d, *J* = 6.6 Hz, H-7′, 2H), 2.27−2.33 (m, H-16′, 2H), 1.62−1.70 (m, H-17′, 2H), 1.15−1.28 (m, H-18′, H-19′, H-20′, H-21′, H-22′, H-23′, 12H), 0.89 (t, *J* = 6.9 Hz, H-24′, 3H); ^{13}C NMR ($CDCl_3$, 125.8 MHz) *δ:* 171.4 (C-15′), 159.2 (C-13′), 136.2 (C-1′), 131.2 (C-9′), 130.1 (C-5′), 129.9 (C-6′), 129.4 (C-10′), 127.4 (C-3′), 127.2 (C-11′), 125.3 (C-8′), 125.0 (C-4′), 123.5 (C-2′), 113.99 (C-12′), 55.2 (C-14′), 37.5 (C-16′), 36.1 (C-7′), 31.79 (C-22′), 29.4 (C-21′), 29.3 (C-20′), 29.2 (C-18′, C-19′), 25.70 (C-17′), 22.6 (C-23′), 14.0 (C-24′).

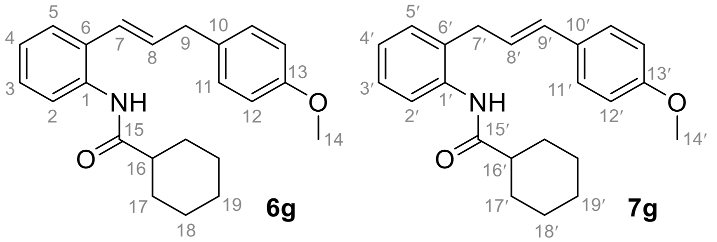

6g **7g**

Following general procedure B with *N*-(2-iodophenyl)cyclohexanecarboxamide **3g** (1.65 g, 5.00 mmol), 1-allyl-4-methoxybenzene **4** (1.23 ml, 8.00 mmol), $Pd(OAc)_2$ (11.2 mg, 50.0 μmol) and Et_3N (3.48 mL, 25.0 mmol), a 49:51 mixture of (*E*)-*N*-(2-(3-(4-methoxyphenyl)prop-1-en-1-yl)phenyl)cyclohexanecarboxamide **6g** and (*E*)-*N*-(2-(3-(4-methoxyphenyl)allyl)phenyl)cyclohexanecarboxamide **7g** was produced (1.19 g, 69%).

49:51 mixture of **6g** and **7g**. White solid. $R_f \approx 0.31$, [UV-active, EtOAc/Hexane 20%]. IR (neat) υ: 3260 (w, NH), 3034 (w), 2931 (w), 1652 (s, C=O), 1511 (s), 1450 (m), 1384 (w), 1300 (w), 1175 (w), 1039 (m, C−O), 824 (w), 747 (s) cm^{-1}; HRMS (+ESI) [M + H]$^+$: 350.2123, $C_{23}H_{28}NO_2$ requires 350.2120.

(*E*)-*N*-(2-(3-(4-Methoxyphenyl)prop-1-en-1-yl)phenyl)cyclohexanecarboxamide **6g**. ^{1}H NMR ($CDCl_3$, 500 MHz) *δ:* 7.88 (d, *J* = 7.8 Hz, H-2, 1H), 7.38 (br s, NH, 1H), 7.34 (d, *J* = 7.8 Hz, H-5, 1H), 7.23 (t, *J* = 7.8 Hz, H-3, 1H), 7.16 (d, *J* = 8.6 Hz, H-11, 2H), 7.11 (t, *J* = 7.8 Hz, H-4, 1H), 6.88 (d, *J* = 8.6 Hz, H-12, 2H), 6.43 (d, *J* = 15.7 Hz, H-7, 1H), 6.27 (dt, *J* = 15.7, 5.8 Hz, H-8, 1H), 3.80 (s, H-14, 3H), 3.53 (d, *J* = 5.8 Hz, H-9, 2H), 2.18 (td, *J* = 11.7, 2.7 Hz, H-16, 1H), 1.92 (d, *J* = 12.6 Hz, H-17α, 2H), 1.75−1.83 (m, H-17β, 2H), 1.65-1.72 (m, H-19β, 1H), 1.40−1.49 (m, H-18α, 2H), 1.15−1.34 (m, H-18β, H-19α, 3H); ^{13}C NMR ($CDCl_3$, 125.8 MHz) *δ:* 174.1 (C-15), 158.2 (C-13), 134.5 (C-8), 134.3 (C-1), 131.5 (C-10), 130.1 (C-6), 129.7 (C-11), 127.8 (C-3), 127.1 (C-5), 125.6 (C-7), 124.8 (C-4), 123.1 (C-2), 114.0 (C-12), 55.2 (C-14), 46.4 (C-16), 38.6 (C-9), 29.7 (C-17), 25.7 (C-18, C-19).

(*E*)-*N*-(2-(3-(4-Methoxyphenyl)allyl)phenyl)cyclohexanecarboxamide **7g**. ^{1}H NMR ($CDCl_3$, 500 MHz) *δ:* 7.97 (d, *J* = 7.6 Hz, H-2′, 1H), 7.38 (br s, NH, 1H), 7.28

(d, J = 8.8 Hz, H-11′, 2H), 7.24−7.28 (m, H-3′, 1H), 7.21−7.24 (m, H-5′, 1H), 7.09 (t, J = 7.6 Hz, H-4′, 1H), 6.85 (d, J = 8.8 Hz, H-12′, 2H), 6.36 (d, J = 15.8 Hz, H-9′, 1H), 6.18 (dt, J = 15.8, 6.0 Hz, H-8′, 1H), 3.81 (s, H-14′, 3H), 3.52 (d, J = 6.0 Hz, H-7′, 2H), 2.18 (td, J = 11.7, 2.7 Hz, H-16′, 1H), 1.92 (d, J = 12.6 Hz, H-17α′, 2H), 1.75−1.83 (m, H-17β′, 2H), 1.65-1.72 (m, H-19β′, 1H), 1.40−1.49 (m, H-18α′, 2H), 1.15−1.34 (m, H-18β′, H-19α′, 3H); ^{13}C NMR ($CDCl_3$, 125.8 MHz) δ: 174.1 (C-15′), 159.2 (C-13′), 136.4 (C-1′), 131.4 (C-9′), 130.2 (C-5′), 130.1 (C-6′), 129.3 (C-10′), 127.5 (C-3′), 127.3 (C-11′), 125.3 (C-8′), 124.8 (C-4′), 123.2 (C-2′), 114.1 (C-12′), 55.3 (C-14′), 46.5 (C-16′), 36.3 (C-7′), 29.8 (C-17′), 25.7 (C-18′, C-19′).

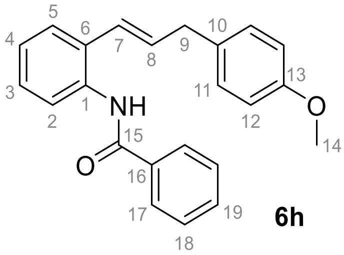

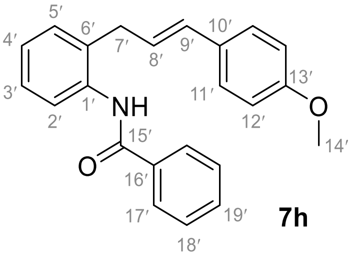

Following general procedure B with *N*-(2-iodophenyl)benzamide **3h** (1.62 g, 5.00 mmol), 1-allyl-4-methoxybenzene **4** (1.23 ml, 8.00 mmol), $Pd(OAc)_2$ (11.2 mg, 50.0 μmol) and Et_3N (3.48 mL, 25.0 mmol), a 47:53 mixture of (*E*)-*N*-(2-(3-(4-methoxyphenyl)prop-1-en-1-yl)phenyl)benzamide **6h** and (*E*)-*N*-(2-(3-(4-methoxyphenyl)allyl)phenyl)benzamide **7h** was produced (1.34 g, 78%). Further partial separation by isocratic elution column chromatography (chloroform/dichloromethane, 60:40) then yielded **7h** (0.37 g, 22%).

47:53 mixture of **6h** and **7h**. White solid. $R_f \approx 0.43$, [UV-active, EtOAc/Hexane 20%]. IR (neat) υ: 3259 (w, NH), 3030 (w), 2834 (w), 1647 (s, C=O), 1512 (s), 1483 (s), 1440 (w), 1300 (m), 1176 (m), 1037 (m, C−O), 815 (w), 753 (s) cm^{-1}; HRMS (+ESI) [M + H]$^+$: 344.1674, $C_{23}H_{22}NO_2$ requires 344.1651.

(*E*)-*N*-(2-(3-(4-Methoxyphenyl)prop-1-en-1-yl)phenyl)benzamide **6h**. ^{1}H NMR ($CDCl_3$, 500 MHz) δ: 7.98 (d, J = 7.9 Hz, H-2, 1H), 7.75 (br s, NH, 1H), 7.70 (d, J = 7.4 Hz, H-17, 2H), 7.49 (t, J = 7.4 Hz, H-19, 1H), 7.41 (d, J = 7.9 Hz, H-5, 1H), 7.29 (t, J = 7.4 Hz, H-18, 2H), 7.26-7.33 (m, H-3, 1H), 7.08 (d, J = 8.7 Hz, H-11, 2H), 7.06 – 7.09 (m, H-4, 1H), 6.78 (d, J = 8.7 Hz, H-12, 2H), 6.39 (d, J = 15.7 Hz, H-7, 1H), 6.26 (dt, J = 15.7, 6.0 Hz, H-8, 1H), 3.72 (s, H-14, 3H), 3.45 (d, J = 6.0 Hz, H-9, 2H); ^{13}C NMR ($CDCl_3$, 125.8 MHz) δ: 165.4 (C-15), 158.1 (C-13), 134.9 (C-8, C-16), 134.3 (C-1), 131.8 (C-19), 131.4 (C-10), 130.02 (C-6), 129.7 (C-11), 128.8 (C-18), 127.9 (C-3), 127.3 (C-5), 127.1 (C-17), 125.6 (C-7), 125.1 (C-4), 123.1 (C-2), 114.0 (C-12), 55.3 (C-14), 38.6 (C-9).

(*E*)-*N*-(2-(3-(4-Methoxyphenyl)allyl)phenyl)benzamide **7h**. ^{1}H NMR ($CDCl_3$, 500 MHz) δ: 8.16 (br s, NH, 1H), 8.04 (d, J = 7.7 Hz, H-2′, 1H), 7.70 (d, J = 7.4 Hz, H-17′, 2H), 7.43 (t, J = 7.4 Hz, H-19′, 1H), 7.29 (t, J = 7.4 Hz, H-18′, 2H), 7.26-7.33 (m, H-3′, 1H), 7.22 (d, J = 8.7 Hz, H-11′, 2H), 7.20-7.23 (m, H-5′, 1H), 7.10 (td, J = 7.7,

1.2 Hz, H-4′, 1H), 6.80 (d, *J* = 8.7 Hz, H-12′, 2H), 6.42 (d, *J* = 16.0 Hz, H-9′, 1H), 6.17 (dt, *J* = 16.0, 6.0 Hz, H-8′, 1H), 3.76 (s, H-14′, 3H), 3.53 (d, *J* = 6.0 Hz, H-7′, 2H); ^{13}C NMR ($CDCl_3$, 125.8 MHz) δ: 165.4 (C-15′), 159.3 (C-13′), 136.6 (C-1′), 134.8 (C-16′), 134.8 (C-8′), 131.7 (C-9′, C-19′), 130.3 (C-5′), 129.99 (C-6′), 129.1 (C-10′), 128.7 (C-18′), 127.7 (C-3′), 127.4 (C-11′), 127.0 (C-17′), 125.2 (C-8′), 125.1 (C-4′), 123.1 (C-2′), 114.1 (C12′), 55.2 (C-14′), 36.8 (C-7′).

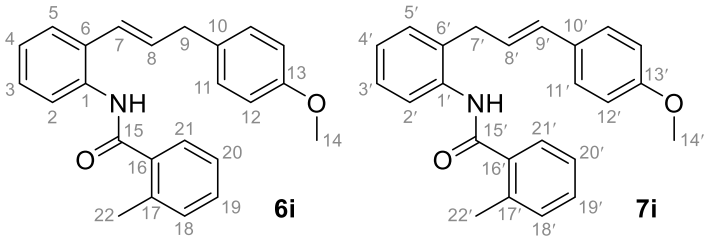

Following general procedure B with *N*-(2-iodophenyl)-2-methylbenzamide **3i** (1.69 g, 5.00 mmol), 1-allyl-4-methoxybenzene **4** (1.23 ml, 8.00 mmol), $Pd(OAc)_2$ (11.2 mg, 50.0 μmol) and Et_3N (3.48 mL, 25.0 mmol), a 49:51 mixture of (*E*)-*N*-(2-(3-(4-methoxyphenyl)prop-1-en-1-yl)phenyl)-2-methylbenzamide **6i** and (*E*)-*N*-(2-(3-(4-methoxyphenyl)allyl)phenyl)-2-methyl benzamide **7i** was produced (1.03 g, 58%). Further complete separation by isocratic elution column chromatography (chloroform/dichloromethane, 60:40) then yielded pure **6i** (0.46 g, 26%) and **7i** (0.55 g, 32%).

49:51 mixture of **6i** and **7i**. White solid. $R_f \approx 0.55$, [UV-active, EtOAc/Hexane 25%]. White solid. IR (neat) υ: 3275 (w, NH), 3027 (w), 2997 (w), 1646 (s, C=O), 1514 (s), 1444 (m), 1306 (m), 1271 (m), 1249, 1177 (m), 1037 (m, C–O), 827 (w), 748 (s) cm^{-1}; HRMS (+ESI) $[M + H]^+$: 358.1801, $C_{24}H_{24}NO_2$ requires 358.1807.

(*E*)-*N*-(2-(3-(4-Methoxyphenyl)prop-1-en-1-yl)phenyl)-2-methylbenzamide **6i**. ^{1}H NMR ($CDCl_3$, 500 MHz) δ: 8.02 (d, *J* = 7.5 Hz, H-2, 1H), 7.76 (br s, NH, 1H), 7.43–7.35 (m, H-5, H-19, H-21, 3H), 7.31–7.26 (m, H-3, H-18, 2H), 7.18–7.13 (m, H-4, H-20, 2H), 7.11 (d, *J* = 8.5 Hz, H-11, 2H), 6.82 (d, *J* = 8.5 Hz, H-12, 2H), 6.44 (br d, *J* = 15.5 Hz, H-7, 1H), 6.29 (dt, *J* = 15.5, 6.5 Hz, H-8, 1H), 3.79 (s, H-14, 3H), 3.50 (br d, *J* = 6.5 Hz, H-9, 2H), 2.51 (s, H-22, 3H); ^{13}C NMR ($CDCl_3$, 125.8 MHz) δ: 168.0 (C-15), 158.1 (C-13), 136.54 (C-16), 136.3 (C-17), 134.6 (C-8), 134.2 (C-1), 131.36 (C-10), 131.28 (C-19), 130.3 (C-6), 130.20 (C-18), 129.6 (C-11), 127.9 (C-3), 127.2 (C-5), 126.57 (C-21), 125.9 (C-20), 125.5, 125.4 (C-4, C-7), 123.3 (C-2), 114.0 (C12), 55.2 (C-14), 38.5 (C-9), 19.9 (C-22).

(*E*)-*N*-(2-(3-(4-Methoxyphenyl)allyl)phenyl)-2-methylbenzamide **7i**. ^{1}H NMR ($CDCl_3$, 500 MHz) δ: 8.16 (d, *J* = 7.5 Hz, H-2′, 1H), 7.75 (br s, NH, 1H), 7.37 (br d, *J* = 8.0 Hz, H-21′, 1H), 7.34 (dd, *J* = 7.5, 7.0 Hz, H-19′, 1H), 7.33 (td, *J* = 7.5, 1.0 Hz, H-3′, 1H), 7.27 (dd, *J* = 7.5, 1.0 Hz, H-5′, 1H), 7.23 (d, *J* = 7.5 Hz, H-18′, 1H), 7.17 (br d, *J* = 8.5 Hz, H-11′, 2H), 7.16 (ddd, *J* = 8.0, 7.0, 1.0, H-20′, 1H), 7.08 (t, *J* = 7.5 Hz, H-4′, 1H), 6.82 (br d, *J* = 8.5 Hz, H-12′, 2H), 6.30 (dt, *J* = 16.0, 1.5 Hz, H-9′, 1H),

6.16 (dt, *J* = 16.0, 6.5 Hz, H-8′, 1H), 3.81 (s, H-14′, 3H), 3.55 (dd, *J* = 6.5, 1.5 Hz, H-7′, 2H), 2.48 (s, H-22′, 3H); ^{13}C NMR ($CDCl_3$, 125.8 MHz) δ: 167.9 (C-15′), 159.2 (C-13′), 136.7 (C-1′), 136.48 (C-16′), 136.2 (C-17′), 131.5 (C-19′), 131.33 (C-9′), 130.3, 130.17 (C-5′, C18′), 129.9 (C-6′), 129.3 (C-10′), 127.6 (C-3′), 127.3 (C-11′), 126.65 (C-21′), 125.8 (C-20′), 125.2 (C-8′), 125.0 (C-4′), 122.9 (C-2′), 113.9 (C12′), 55.3 (C-14′), 36.3 (C-7′), 19.9 (C-22′).

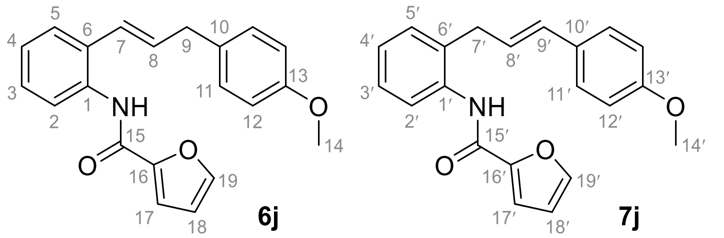

6j **7j**

Following general procedure B with *N*-(2-iodophenyl)furan-2-carboxamide **3j** (626 mg, 2.00 mmol), 1-allyl-4-methoxybenzene **4** (491 μl, 3.20 mmol), $Pd(OAc)_2$ (4.48 mg, 20.0 μmol) and Et_3N (1.39 mL, 10.0 mmol), a 47:53 mixture of (*E*)-*N*-(2-(3-(4-methoxyphenyl)prop-1-en-1-yl)phenyl)furan-2-carboxamide **6j** and (*E*)-*N*-(2-(3-(4-methoxyphenyl)allyl)phenyl)furan-2-carboxamide **7j** was produced (0.24 g, 36%).

47:53 mixture of **6j** and **7j**. Amber oil. $R_f \approx 0.42$, [UV-active, EtOAc/Hexane 20%]. Amber oil. IR (neat) υ: 3355 (w, NH), 3126 (w), 2934 (w), 1669 (m, C=O), 1510 (s), 1450 (m), 1301 (m), 1164 (m), 1010 (m, C−O), 835 (w), 753 (s) cm^{-1}; HRMS (+ESI) [M + H]$^+$:334.1445, $C_{21}H_{20}NO_3$ requires 334.1443.

(*E*)-*N*-(2-(3-(4-Methoxyphenyl)prop-1-en-1-yl)phenyl)furan-2-carboxamide **6j**. ^{1}H NMR ($CDCl_3$, 500 MHz) δ: 8.44 (br s, NH, 1H), 8.10 (d, *J* = 8.2 Hz, H-19, 1H), 7.26-7.31 (m, H-2, H-3, H-5, 3H), 7.18 (d, *J* = 7.5 Hz, H-11, 2H), 7.12-7.15 (m, H-4, H-17, 2H), 6.86 (d, *J* = 7.5 Hz, H-12, 2H), 6.58 (d, *J* = 15.8 Hz, H-7, 1H), 6.54-6.57 (m, H-18, 1H), 6.31 (dt, *J* = 15.8, 6.2 Hz, H-8, 1H), 3.80 (s, H-14, 3H), 3.55 (d, *J* = 6.2 Hz, H-7, 2H); ^{13}C NMR ($CDCl_3$, 125.8 MHz) δ: 158.1 (C-13), 156.1 (C-15), 148.0 (C-16), 144.2 (C-19), 134.8 (C-8), 133.7 (C-1), 131.5 (C-10), 129.7 (C-11), 129.48 (C-6), 127.9 (C-3), 125.5 (C-5), 125.1 (C-7), 125.0 (C-4), 122.6 (C-2), 115.12 (C-17), 114.0 (C-12), 112.5 (C-18) , 55.2 (C-14), 38.7 (C-9).

(*E*)-*N*-(2-(3-(4-Methoxyphenyl)allyl)phenyl)furan-2-carboxamide **7j**. ^{1}H NMR ($CDCl_3$, 500 MHz) δ: 8.15 (br s, NH, 1H), 8.10 (d, *J* = 8.2 Hz, H-19′, 1H), 7.39 (d, *J* = 7.5 Hz, H-2′, 1H), 7.29 (d, *J* = 7.6 Hz, H-11′, 2H), 7.26-7.31 (m, H-3′, H-5′, 2H), 7.12-7.15 (m, H-4′, H-17′, 2H), 6.85 (d, *J* = 7.6 Hz, H-12′, 2H), 6.54-6.57 (m, H-18′, 1H), 6.52 (d, *J* = 15.5 Hz, H-9′, 1H), 6.20 (dt, *J* = 15.5, 6.1 Hz, H-8′, 1H), 3.80 (s, H-14, 3H), 3.61 (d, *J* = 6.1 Hz, H-7′, 2H); ^{13}C NMR ($CDCl_3$, 125.8 MHz) δ: 159.2 (C-13′), 156.0 (C-15′), 148.0 (C-16′), 144.2 (C-19′), 135.9 (C-1′), 131.9 (C-9′), 130.2 (C-5′), 130.0 (C-6′), 129.54 (C-10′), 127.6 (C-3′), 127.3 (C-11′), 125.0 (C-8′), 124.8 (C-4′), 122.9 (C-2′), 115.08 (C-17′), 114.0 (C-12′), 112.4 (C-18′), 55.2 (C-14′), 36.6 (C-7′).

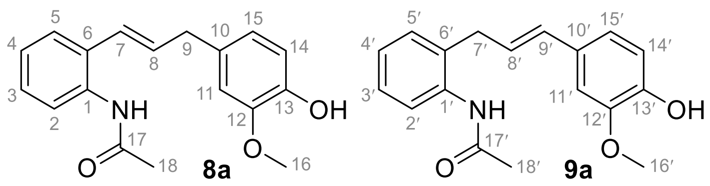

Following general procedure B with *N*-(2-iodophenyl)acetamide **3a** (1.31 g, 5.00 mmol), 4-allyl-2-methoxyphenol **5** (1.23 mL, 8.00 mmol), $Pd(OAc)_2$ (11.2 mg, 50.0 µmol) and Et_3N (2.44 mL, 17.5 mmol), a 38:62 mixture of (*E*)-*N*-(2-(3-(4-hydroxy-3-methoxyphenyl)prop-1-en-1-yl)phenyl)acetamide **8a** and (*E*)-*N*-(2-(3-(4-hydroxy-3-methoxyphenyl)allyl)phenyl) acetamide **9a** was produced (0.74 g, 49%). Further partial separation by isocratic elution column chromatography (chloroform/dichloromethane, 60:40) then yielded pure **9a** (0.52 g, 34 %).

38:62 mixture of **8a** and **9a**. Yellowish-white solid. $R_f \approx 0.13$, [UV-active, EtOAc/Hexane 40%]. IR (neat) υ: 3348 (w, OH), 3229 (w, NH), 3125 (w), 2965 (w), 1636 (m, C=O), 1517 (s), 1460 (w), 1374 (w), 1273 (s), 1236 (m), 1175 (m), 1033 (m, C–O), 868 (w), 822 (w), 797 (w), 756 (s) cm^{-1}; HRMS (+ESI) $[M + H]^+$: 298.1435, $C_{18}H_{20}NO_3$ requires 298.1443.

(*E*)-*N*-(2-(3-(4-Hydroxy-3-methoxyphenyl)prop-1-en-1-yl)phenyl)acetamide **8a**. ^1H NMR ($CDCl_3$, 500 MHz) δ: 7.77 (d, J = 8.0 Hz, H-2, 1H), 7.38 (d, J = 7.5 Hz, H-5, 1H), 7.29 (br s, NH, 1H), 6.64-7.17 (m, H-3, H-4, H-11, H-14, H-15, 5H), 6.45 (br d, J = 15.5 Hz, H-7, 1H), 6.26 (dt, J = 15.5, 7.0 Hz, H-8, 1H), 5.56 (s, OH, 1H), 3.89 (s, H-16, 3H), 3.51 (d, J = 7.0 Hz, H-9, 2H), 2.15 (s, H-18, 3H); ^{13}C NMR ($CDCl_3$, 125.8 MHz) δ: 168.3 (C-17), 146.6 (C-12), 144.2 (C-13), 134.2 (C-1), 134.0 (C-8), 131.5 (C-10), 130.5 (C-6), 127.9 (C-3), 127.0 (C-5), 125.7 (C-7), 125.4 (C-4), 123.8 (C-2), 121.2 (C-15), 114.43 (C-14), 111.2 (C-11), 55.95 (C-16), 39.2 (C-9), 24.2 (C-18).

(*E*)-*N*-(2-(3-(4-Hydroxy-3-methoxyphenyl)allyl)phenyl)acetamide **9a**. ^1H NMR ($CDCl_3$, 500 MHz) δ: 7.86 (d, J = 8.0 Hz, H-2′, 1H), 7.29 (br s, NH, 1H), 7.29 (ddd, J = 8.0, 7.5, 1.0 Hz, H-3′, 1H), 7.24 (d, J = 8.0 Hz, H-5′, 1H), 7.15 (dd, J = 8.0, 7.5 Hz, H-4′, 1H), 6.89–6.82 (m, H11′, H14′, H15′, 3H), 6.38 (dt, J = 16.0, 1.5 Hz, H-9′, 1H), 6.16 (dt, J = 16.0, 6.5 Hz, H-8′, 1H), 5.64 (s, OH, 1H), 3.89 (s, H-16′, 3H), 3.53 (dd, J = 6.5, 1.5 Hz, H-7′, 2H), 2.12 (s, H-18′, 3H); ^{13}C NMR ($CDCl_3$, 125 MHz) δ: 168.2 (C-17′), 146.7 (C-12′), 145.6 (C-13′), 136.1 (C-1′), 131.5 (C-9′), 130.44 (C-6′), 130.25 (C-5′), 129.2 (C-10′), 127.6 (C-3′), 125.4, 125.3 (C-4′, C-8′), 123.8 (C-2′), 120.0 (C-15′), 114.48 (C-14′), 108.0 (C-11′), 55.90 (C-16′), 36.1 (C-7′), 24.4 (C-18′).

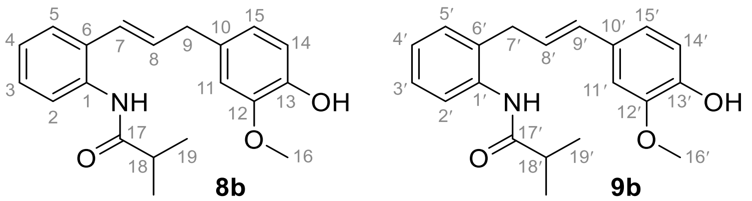

Following general procedure B with *N*-(2-iodophenyl)isobutyramide **3b** (1.45 g, 5.00 mmol), 4-allyl-2-methoxyphenol **5** (1.23 mL, 8.00 mmol), $Pd(OAc)_2$ (11.2 mg, 50.0 µmol) and Et_3N (2.44 mL, 17.5 mmol), a 21:79 mixture of (*E*)-*N*-(2-(3-(4-hydroxy-3-methoxyphenyl)prop-1-en-1-yl)phenyl)isobutyramide **8b** and (*E*)-*N*-(2-(3-(4-hydroxy-3-methoxyphenyl)allyl)phenyl)isobutyramide **9b** was produced (1.19 g, 75%). Further partial separation by isocratic elution column chromatography (chloroform/dichloromethane, 60:40) then yielded pure **9b** (0.69 g, 43 %).

21:79 mixture of **8b** and **9b**. White solid. $R_f \approx 0.11$, [UV-active, EtOAc/Hexane 20%]. IR (neat) υ: 3398 (w, OH), 3291 (m, NH), 3016 (w), 2970 (w), 1654 (s, C=O), 1509 (s), 1446 (m), 1371 (w), 1273 (s), 1236 (s), 1155 (m), 1038 (m, C−O), 848 (m), 812 (w), 798 (w), 753 (s) cm^{-1}; HRMS (+ESI) $[M + H]^+$: 326.1755, $C_{20}H_{24}NO_3$ requires 326.1756.

(*E*)-*N*-(2-(3-(4-Hydroxy-3-methoxyphenyl)prop-1-en-1-yl)phenyl)isobutyramide **8b**. ^1H NMR ($CDCl_3$, 500 MHz) δ: 7.84 (d, *J* = 7.6 Hz, H-2, 1H), 7.43 (d, *J* = 7.6 Hz, H-5, 1H), 7.36 (br s, NH, 1H), 7.21 (td, *J* = 7.6, 1.7 Hz, H-3, 1H), 7.06 (t, *J* = 7.6 Hz, H-4, 1H), 6.72-6.74 (m, H-11, H-14, H-15, 3H), 6.42 (d, *J* = 15.8 Hz, H-7, 1H), 6.27 (dt, *J* = 15.8, 6.5 Hz, H-8, 1H), 5.61 (br s, OH, 1H), 3.88 (s, H-16, 3H), 3.50 (d, *J* = 6.5 Hz, H-9, 2H), 2.47 (sept, *J* = 7.0 Hz, H-18, 1H), 1.20 (d, *J* = 7.0 Hz, H-19, 6H); ^{13}C NMR ($CDCl_3$, 125.8 MHz) δ: 175.1 (C-17), 146.7 (C-13), 144.2 (C-12), 135.7 (C-1), 134.2 (C-8), 131.4 (C-10), 130.0 (C-6), 127.9 (C-3), 127.0 (C-5), 125.6 (C-7), 124.9 (C-4), 122.8 (C-2), 121.3 (C-15), 114.7 (C-14), 111.2 (C-11), 55.9 (C-16), 39.2 (C-9), 36.7 (C-18), 19.6 (C-19).

(*E*)-*N*-(2-(3-(4-Hydroxy-3-methoxyphenyl)allyl)phenyl)isobutyramide **9b**. ^1H NMR ($CDCl_3$, 500 MHz) δ: 7.95 (d, *J* = 8.0 Hz, H-2′, 1H), 7.35 (br s, NH, 1H), 7.29 (ddd, *J* = 8.0, 7.5, 1.0 Hz, H-3′, 1H), 7.23 (br d, *J* = 7.5 Hz, H-5′, 1H), 7.13 (t, *J* = 7.5 Hz, H-4′, 1H), 6.85 (s, H-11′, 1H), 6.84 (AB system, δ_A = 6.84, δ_B = 6.85, J_{AB} = 9.0 Hz, H15′, H14′, 2H), 6.38 (br d, *J* = 16.0 Hz, H-9′, 1H), 6.16 (dt, *J* = 16.0, 6.0 Hz, H-8′, 1H), 5.73 (br s, OH, 1H), 3.87 (s, H-16′, 3H), 3.53 (br d, *J* = 6.0 Hz, H-7′, 2H), 2.47 (sept, *J* = 7.0 Hz, H-18′, 1H), 1.21 (d, *J* = 7.0 Hz, H-19′, 6H); ^{13}C NMR ($CDCl_3$, 125.8 MHz) δ: 175.1 (C-17′), 146.6 (C-12′), 145.5 (C-13′), 136.3 (C-1′), 131.6 (C-9′), 130.2 (C-5′), 130.0 (C-6′), 129.2 (C-10′), 127.5 (C-3′), 125.1, 125.0 (C-4′, C-8′), 123.4 (C-2′), 119.9 (C-15′), 114.4 (C-14′), 108.0 (C-11′), 55.9 (C-16′), 36.7 (C-18′), 36.0 (C-7′), 19.6 (C-19′).

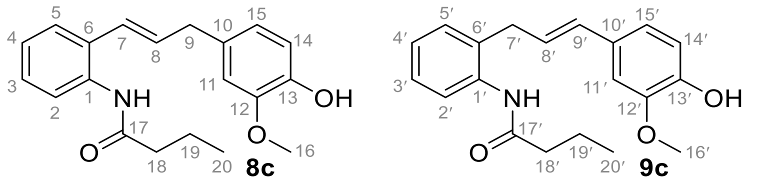

Following general procedure B with *N*-(2-iodophenyl)butyramide **3c** (1.45 g, 5.00 mmol), 4-allyl-2-methoxyphenol **5** (1.23 mL, 8.00 mmol), $Pd(OAc)_2$ (11.2 mg, 50.0 μmol) and Et_3N (2.44 mL, 17.5 mmol), a 26:74 mixture of (*E*)-*N*-(2-(3-(4-hydroxy-3-methoxyphenyl)prop-1-en-1-yl)phenyl)butyramide **8c** and (*E*)-*N*-(2-(3-(4-hydroxy-3-methoxyphenyl)allyl)phenyl)butyramide **9c** was produced (1.31 g, 79%).

26:74 mixture of **8c** and **9c**. Yellowish-white solid. $R_f \approx 0.22$, [UV-active, EtOAc/Hexane 30%]. Yellowish-white solid. IR (neat) υ: 3399 (w, OH), 3269 (w, NH), 3053 (w), 2936 (w), 1646 (s, C=O), 1510 (s), 1453 (m), 1376 (w), 1271 (s), 1229 (s), 1155 (m), 1034 (m, C−O), 849 (w), 806 (w), 794 (w), 756 (s) cm^{-1}; HRMS (+ESI) $[M + H]^+$: 326.1768, $C_{20}H_{24}NO_3$ requires 326.1756.

(*E*)-*N*-(2-(3-(4-Hydroxy-3-methoxyphenyl)prop-1-en-1-yl)phenyl)butyramide **8c**. ^1H NMR ($CDCl_3$, 500 MHz) δ: 7.82 (d, *J* = 7.7 Hz, H-2, 1H), 7.32 (br s, NH, 1H), 7.37 (d, *J* = 7.7 Hz, H-5, 1H), 7.08-7.09 (m, H-3, 1H), 7.01 (t, *J* = 7.7 Hz, H-4, 1H), 6.73-6.75 (m, H-11, H-14, H-15, 3H), 6.42 (d, *J* = 15.3 Hz, H-7, 1H), 6.26 (dt, *J* = 15.3, 7.1 Hz, H-8, 1H), 5.57 (s, OH, 1H), 3.88 (s, H-16, 3H), 3.50 (d, *J* = 7.1 Hz, H-9, 2H), 2.29 (t, *J* = 7.3 Hz, H-18, 2H), 1.71 (sext, *J* = 7.3 Hz, H-19, 2H), 0.96 (t, *J* = 7.3 Hz, H-20, 3H); ^{13}C NMR ($CDCl_3$, 125.8 MHz) δ: 171.2 (C-17), 146.6 (C-13), 144.2 (C-12), 134.2 (C-1), 134.1 (C-8), 131.4 (C-10), 130.0 (C-6), 127.9 (C-3), 127.0 (C-5), 125.6 (C-7), 125.1 (C-4), 123.5 (C-2), 121.2 (C-15), 114.4 (C-14), 111.2 (C-11), 56.0 (C-16), 39.7 (C-18), 39.2 (C-9), 19.2 (C-19), 13.7 (C-20).

(*E*)-*N*-(2-(3-(4-Hydroxy-3-methoxyphenyl)allyl)phenyl)butyramide **9c**. ^1H NMR ($CDCl_3$, 500 MHz) δ: 7.92 (d, *J* = 7.7 Hz, H-2′, 1H), 7.32 (br s, NH, 1H), 7.28 (t, *J* = 7.7 Hz, H-3′, 1H), 7.23 (d, *J* = 7.7 Hz, H-5′, 1H), 7.13 (t, *J* = 7.7 Hz, H-4′, 1H), 6.88 (s, H-11′, 1H), 6.84 (d, *J* = 8.2 Hz, H-15′, 1H), 6.82 (d, *J* = 8.2 Hz, H-14′, 1H), 6.38 (d, *J* = 15.9 Hz, H-9′, 1H), 6.16 (dt, *J* = 15.9, 6.2 Hz, H-8′, 1H), 5.69 (s, OH, 1H), 3.88 (s, H-16′, 3H), 3.52 (d, *J* = 6.2 Hz, H-7′, 2H), 2.29 (t, *J* = 7.3 Hz, H-18′, 2H), 1.71 (sext, *J* = 7.3 Hz, H-19′, 2H), 0.96 (t, *J* = 7.3 Hz, H-20′, 3H); ^{13}C NMR ($CDCl_3$, 125.8 MHz) δ: 171.2 (C-17′), 146.7 (C-12′), 145.5 (C-13′), 136.3 (C-1′), 131.6 (C-9′), 130.2 (C-5′), 130.0 (C-6′), 129.2 (C-10′), 127.5 (C-3′), 125.2, 125.1 (C-4′, C-8′), 123.5 (C-2′), 119.9 (C-15′), 114.4 (C-14′), 108.0 (C-11′), 55.9 (C-16′), 39.7 (C-18′), 36.1 (C-7′), 19.2 (C-19′), 13.7 (C-20′).

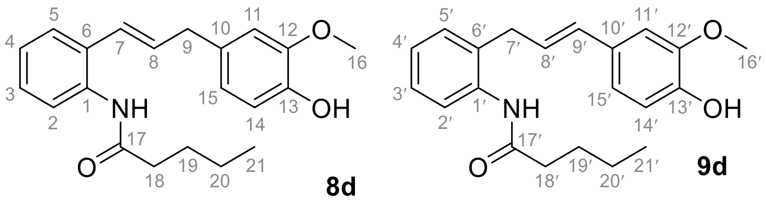

Following general procedure B with *N*-(2-iodophenyl)pentanamide **3d** (1.52 g, 5.00 mmol), 4-allyl-2-methoxyphenol **5** (1.23 mL, 8.00 mmol), $Pd(OAc)_2$ (11.2 mg, 50.0 µmol) and Et_3N (2.44 mL, 17.5 mmol), a 22:78 mixture of (*E*)-*N*-(2-(3-(4-hydroxy-3-methoxyphenyl)prop-1-en-1-yl)phenyl)pentanamide **8d** and (*E*)-*N*-(2-(3-(4-hydroxy-3-methoxyphenyl)allyl)phenyl)pentanamide **9d** was produced (1.50 g, 82%).

22:78 mixture of **8d** and **9d**. Yellowish-white solid. $R_f \approx 0.13$, [UV-active, EtOAc/Hexane 20%]. IR (neat) ʋ: 3302 (w, OH), 3270 (w, NH), 3027 (w), 2961 (w), 1647 (s, C=O), 1510 (s), 1452 (m), 1377 (w), 1272 (m), 1229 (s), 1155 (m), 1039 (m, C–O), 850 (w), 818 (w), 782 (w), 755 (s) cm^{-1}; HRMS (+ESI) $[M + H]^+$:340.1908, $C_{21}H_{26}NO_3$ requires 340.1913.

(*E*)-*N*-(2-(3-(4-Hydroxy-3-methoxyphenyl)prop-1-en-1-yl)phenyl)pentanamide **8d**. ^1H NMR ($CDCl_3$, 500 MHz) δ: 7.80 (d, J = 7.5 Hz, H-2, 1H), 7.09-7.41 (m, NH, H-3, H-4, H-5, 4H), 6.72-6.73 (m, H-11, H-14, H-15, 3H), 6.43 (d, J = 15.3 Hz, H-7, 1H), 6.26 (dt, J = 15.3, 6.9 Hz, H-8, 1H), 5.64 (s, OH, 1H), 3.88 (s, H-16, 3H), 3.50 (d, J = 6.9 Hz, H-9, 2H), 2.30 (t, J = 7.5 Hz, H-18, 2H), 1.63 – 1.66 (m, H-19, 2H), 1.32 – 1.37 (m, H-20, 2H), 0.94 (t, J = 7.5 Hz, H-21, 3H); ^{13}C NMR ($CDCl_3$, 125.8 MHz) δ: 171.4 (C-17), 146.6 (C-13), 144.2 (C-12), 134.2 (C-1), 134.0 (C-8), 131.4 (C-10), 130.1 (C-6), 127.8 (C-3), 127.0 (C-5), 125.7 (C-7), 125.1 (C-4), 123.5 (C-2), 121.2 (C-15), 114.4 (C-14), 111.2 (C-11), 55.9 (C-16), 39.2 (C-9), 37.3 (C-18), 27.8 (C-19), 22.31 (C-20), 13.72 (C-21).

(*E*)-*N*-(2-(3-(4-Hydroxy-3-methoxyphenyl)allyl)phenyl)pentanamide **9d**. ^1H NMR ($CDCl_3$, 500 MHz) δ: 7.91 (d, J = 7.8 Hz, H-2′, 1H), 7.37 (br s, NH, 1H), 7.28 (t, J = 7.8 Hz, H-3′, 1H), 7.23 (d, J = 7.8 Hz, H-5′, 1H), 7.13 (t, J = 7.8 Hz, H-4′, 1H), 6.86 (s, H-11′, 1H), 6.85 (d, J = 8.2 Hz, H-15′, 1H), 6.83 (d, J = 8.2 Hz, H-14′, 1H), 6.38 (d, J = 15.9 Hz, H-9′, 1H), 6.16 (dt, J = 15.9, 6.2 Hz, H-8′, 1H), 5.77 (s, OH, 1H), 3.87 (s, H-16′, 3H), 3.52 (d, J = 6.2 Hz, H-7′, 2H), 2.30 (t, J = 7.4 Hz, H-18′, 2H), 1.63 – 1.66 (m, H-19′, 2H), 1.32 – 1.37 (m, H-20′, 2H), 0.88 (t, J = 7.4 Hz, H-21′, 3H); ^{13}C NMR ($CDCl_3$, 125.8 MHz) δ: 171.4 (C-17′), 146.7 (C-12′), 145.5 (C-13′), 136.2 (C-1′), 131.6 (C-9′), 130.2 (C-5′), 130.1 (C-6′), 129.2 (C-10′), 127.5 (C-3′), 125.2, 125.1 (C-4′, C-8′), 123.5 (C-2′), 119.9 (C-15′), 114.5 (C-14′), 108.0 (C-11′), 55.8 (C-16′), 37.5 (C-18′), 36.1 (C-7′), 27.8 (C-19′), 22.34 (C-20′), 13.68 (C-21′).

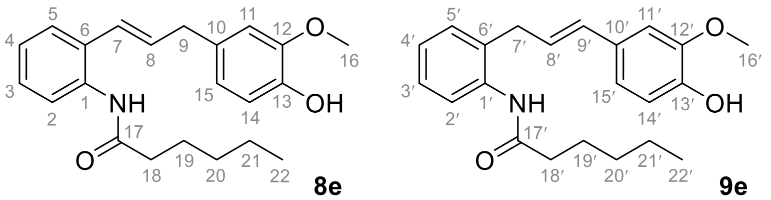

8e **9e**

Following general procedure B with *N*-(2-iodophenyl)hexanamide **3e** (1.59 g, 5.00 mmol), 4-allyl-2-methoxyphenol **5** (1.23 mL, 8.00 mmol), $Pd(OAc)_2$ (11.2 mg, 50.0 µmol) and Et_3N (2.44 mL, 17.5 mmol), a 21:79 mixture of (*E*)-*N*-(2-(3-(4-hydroxy-3-methoxyphenyl)prop-1-en-1-yl)phenyl)hexanamide **8e** and (*E*)-*N*-(2-(3-(4-hydroxy-3-methoxyphenyl)allyl)phenyl)hexanamide **9e** was produced (1.00 g, 56%).

21:79 mixture of **8e** and **9e**. White solid. $R_f \approx 0.14$, [UV-active, EtOAc/Hexane 20%]. IR (neat) υ: 3394 (w, OH), 3292 (w, NH), 3027 (w), 2955 (w), 1648 (s, C=O), 1511 (s), 1450 (m), 1372 (w), 1274 (s), 1236 (s), 1154 (m), 1038 (m, C–O), 851 (w), 817 (w), 785 (w), 754 (m) cm^{-1}; HRMS (+ESI) $[M + H]^+$: 354.2060, $C_{22}H_{28}NO_3$ requires 354.2069.

(*E*)-*N*-(2-(3-(4-Hydroxy-3-methoxyphenyl)prop-1-en-1-yl)phenyl)hexanamide **8e**. ^1H NMR ($CDCl_3$, 500 MHz) δ: 7.81 (d, *J* = 8.1 Hz, H-2, 1H), 7.09-7.38 (m, NH, H-3, H-4, H-5, 4H), 6.72-6.73 (m, H-11, H-14, H-15, 3H), 6.44 (d, *J* = 15.6 Hz, H-7, 1H), 6.25 (dt, *J* = 15.6, 6.9 Hz, H-8, 1H), 5.60 (s, OH, 1H), 3.88 (s, H-16, 3H), 3.50 (d, *J* = 6.9 Hz, H-9, 2H), 2.30 (t, *J* = 7.2 Hz, H-18, 2H), 1.65-1.68 (m, H-19, 2H), 1.34 – 1.36 (m, H-20, H-21, 4H), 0.92 (t, *J* = 7.2 Hz, H-22, 3H); ^{13}C NMR ($CDCl_3$, 125.8 MHz) δ: 171.4 (C-17), 146.6 (C-13), 144.2 (C-12), 134.3 (C-1), 134.1 (C-8), 131.5 (C-10), 130.0 (C-6), 127.9 (C-3), 127.0 (C-5), 125.7 (C-7), 125.1 (C-4), 123.5 (C-2), 121.2 (C-15), 114.4 (C-14), 111.2 (C-11), 55.91 (C-16), 39.2 (C-9), 37.5 (C-18), 31.4 (C-20), 25.4 (C-19), 22.4 (C-21), 13.9 (C-22).

(*E*)-*N*-(2-(3-(4-Hydroxy-3-methoxyphenyl)allyl)phenyl)hexanamide **9e**. ^1H NMR ($CDCl_3$, 500 MHz) δ: 7.92 (d, *J* = 7.7 Hz, H-2′, 1H), 7.36 (br s, NH, 1H), 7.28 (t, *J* = 7.7 Hz, H-3′, 1H), 7.24 (d, *J* = 7.7 Hz, H-5′, 1H), 7.13 (t, *J* = 7.7 Hz, H-4′, 1H), 6.86 (s, H-11′, 1H), 6.85 (d, *J* = 8.2 Hz, H-15′, 1H), 6.84 (d, *J* = 8.2 Hz, H-14′, 1H), 6.38 (d, *J* = 15.9 Hz, H-9′, 1H), 6.15 (dt, *J* = 15.9, 6.2 Hz, H-8′, 1H), 5.73 (s, OH, 1H), 3.87 (s, H-16′, 3H), 3.52 (d, *J* = 6.2 Hz, H-7′, 2H), 2.30 (t, *J* = 7.5 Hz, H-18′, 2H), 1.65-1.68 (m, H-19′, 2H), 1.28 – 1.29 (m, H-20′, H-21′, 4H), 0.86 (t, *J* = 7.5 Hz, H-22′, 3H); ^{13}C NMR ($CDCl_3$, 125.8 MHz) δ: 171.4 (C-17′), 146.7 (C-12′), 145.6 (C-13′), 136.3 (C-1′), 131.6 (C-9′), 130.2 (C-5′), 130.0 (C-6′), 129.2 (C-10′), 127.5 (C-3′), 125.2, 125.1 (C-4′, C-8′), 123.5 (C-2′), 119.9 (C-15′), 114.5 (C-14′), 108.0 (C-11′), 55.86 (C-16′), 37.8 (C-18′), 36.1 (C-7′), 31.4 (C-20′), 25.4 (C-19′), 22.3 (C-21′), 13.8 (C-22′).

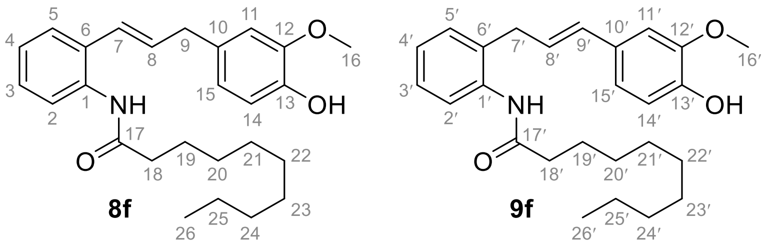

Following general procedure B with *N*-(2-iodophenyl)decanamide **3f** (1.87 g, 5.00 mmol), 4-allyl-2-methoxyphenol **5** (1.23 mL, 8.00 mmol), $Pd(OAc)_2$ (11.2 mg, 50.0 μmol) and Et_3N (2.44 mL, 17.5 mmol), a 28:72 mixture of (*E*)-*N*-(2-(3-(4-hydroxy-3-methoxyphenyl)prop-1-en-1-yl)phenyl)decanamide **8f** and (*E*)-*N*-(2-(3-(4-hydroxy-3-methoxyphenyl)allyl)phenyl)decanamide **9f** was produced (1.19 g, 57%).

28:72 mixture of **8f** and **9f**. Brown solid. $R_f \approx 0.19$, [UV-active, EtOAc/Hexane 20%]. Brown solid. IR (neat) υ: 3399 (w, OH), 3271 (w, NH), 3068 (w), 2923 (m), 2853 (m), 1650 (s, C=O), 1510 (s), 1452 (m), 1377 (w), 1270 (m), 1226 (s), 1155 (m), 1039 (m, C−O), 850 (w), 822 (w), 787 (w), 753 (s) cm^{-1}; HRMS (+ESI) $[M + H]^+$: 410.2691, $C_{26}H_{36}NO_3$ requires 410.2695.

(*E*)-*N*-(2-(3-(4-Hydroxy-3-methoxyphenyl)prop-1-en-1-yl)phenyl)decanamide **8f**. ^1H NMR ($CDCl_3$, 500 MHz) δ: 7.83 (d, *J* = 7.9 Hz, H-2, 1H), 7.37 (d, *J* = 7.9 Hz, H-5, 1H), 7.32 (br s, NH, 1H), 7.30-7.32 (m, H-3, 1H), 7.07-7.09 (m, H-4, 1H), 6.73-6.74 (m, H-11, H-14, H-15, 3H), 6.44 (d, *J* = 15.4 Hz, H-7, 1H), 6.25 (dt, *J* = 15.4, 6.8 Hz, H-8, 1H), 5.54 (s, OH, 1H), 3.89 (s, H-16, 3H), 3.50 (d, *J* = 6.8 Hz, H-9, 2H), 2.30 (t, *J* = 7.3 Hz, H-18, 2H), 1.62 – 1.70 (m, H-19, 2H), 1.23 – 1.29 (m, H-20, H-21, H-22, H-23, H-24, H-25, 12H), 0.87 (t, *J* = 7.3 Hz, H-26, 3H); ^{13}C NMR ($CDCl_3$, 125.8 MHz) δ: 171.4 (C-17), 146.6 (C-13), 144.2 (C-12), 134.1 (C-1), 134.1 (C-8), 131.5 (C-10), 129.9 (C-6), 127.9 (C-3), 127.1 (C-5), 125.1 (C-7, C-4), 123.5 (C-2), 121.2 (C-15), 114.4 (C-14), 111.1 (C-11), 55.9 (C-16, OCH_3), 39.3 (C-9), 37.7 (C-18), 31.8 (C-24), 29.5 (C-23), 29.4 (C-22), 29.34 (C-20), 29.27 (C-21), 25.8 (C-19), 22.6 (C-25), 14.1 (C-26).

(*E*)-*N*-(2-(3-(4-Hydroxy-3-methoxyphenyl)allyl)phenyl)decanamide **9f**. ^1H NMR ($CDCl_3$, 500 MHz) δ: 7.93 (d, *J* = 6.8 Hz, H-2′, 1H), 7.32 (br s, NH, 1H), 7.29 (t, *J* = 6.8 Hz, H-3′, 1H), 7.23 (d, *J* = 6.8 Hz, H-5′, 1H), 7.13 (t, *J* = 6.8 Hz, H-4′, 1H), 6.86 (s, H-11′, 1H), 6.85 (d, *J* = 8.2 Hz, H-15′, 1H), 6.83 (d, *J* = 8.2 Hz, H-14′, 1H), 6.38 (d, *J* = 15.9 Hz, H-9′, 1H), 6.16 (dt, *J* = 15.9, 6.2 Hz, H-8′, 1H), 5.65 (s, OH, 1H), 3.88 (s, H-16′, 3H), 3.52 (d, *J* = 6.2 Hz, H-7′, 2H), 2.30 (t, *J* = 7.3 Hz, H-18′, 2H), 1.62 – 1.70 (m, H-19′, 2H), 1.23 – 1.29 (m, H-20′, H-21′, H-22′, H-23′, H-24′, H-25′, 12H), 0.87 (t, *J* = 7.3 Hz, H-26′, 3H); ^{13}C NMR ($CDCl_3$, 125.8 MHz) δ: 171.4 (C-17′), 146.7 (C-12′), 145.5 (C-13′), 136.3 (C-1′), 131.6 (C-9′), 130.2 (C-5′), 129.9 (C-6′), 129.2 (C-10′), 127.6 (C-3′), 125.2 (C-4′, C-8′), 123.45 (C-2′), 120.0 (C-15′), 114.5 (C-14′), 107.9 (C-11′), 55.87 (C-16′), 37.9 (C-18′), 36.2 (C-7′), 31.8 (C-24′), 29.4 (C-23′), 29.32 (C-22′), 29.29 (C-20′), 29.2 (C-21′), 25.8 (C-19′), 22.6 (C-25′), 14.1 (C-26′).

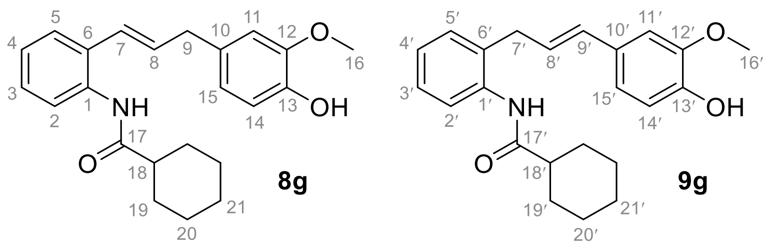

Following general procedure B with *N*-(2-iodophenyl)cyclohexanecarboxamide **3g** (658 mg, 2.00 mmol), 4-allyl-2-methoxyphenol **5** (496 μL, 3.20 mmol), $Pd(OAc)_2$ (4.48 mg, 20.0 μmol) and Et_3N (976 μL, 7.00 mmol), a 19:81 mixture of (*E*)-*N*-(2-(3-(4-hydroxy-3-methoxyphenyl)prop-1-en-1-yl)phenyl)cyclohexanecarboxamide **8g** and (*E*)-*N*-(2-(3-(4-hydroxy-3-methoxyphenyl)allyl)phenyl)cyclohexanecarboxamide **9g** was produced (0.47 g, 62%).

19:81 mixture of **8g** and **9g**. White solid. $R_f \approx 0.16$, [UV-active, EtOAc/Hexane 20%]. IR (neat) ʋ: 3394 (w, OH), 3290 (m, NH), 3030 (w), 2928 (m), 1649 (s, C=O), 1512 (s), 1447 (m), 1386 (w), 1280 (m), 1226 (s), 1156 (m), 1039 (m, C−O), 859 (w), 830 (w), 783 (w), 750 (m) cm^{-1}; HRMS (+ESI) $[M + H]^+$: 366.2065, $C_{23}H_{28}NO_3$ requires 366.2069.

(*E*)-*N*-(2-(3-(4-Hydroxy-3-methoxyphenyl)prop-1-en-1-yl)phenyl)cyclohexanecarboxamide **8g**. ^1H NMR ($CDCl_3$, 500 MHz) δ: 7.84 (d, *J* = 8.0 Hz, H-2, 1H), 7.39 (br s, NH, 1H), 7.37 (t, *J* = 8.0 Hz, H-5, 1H), 7.25 – 7.29 (m, H-3, 1H), 7.08 – 7.11 (m, H-4, 1H), 6.73 – 6.74 (m, H-11, H-14, H-15, 3H), 6.38 (d, *J* = 15.3 Hz, H-7, 1H), 6.27 (dt, *J* = 15.3, 6.4 Hz, H-8, 1H), 5.57 (s, OH, 1H), 3.89 (s, H-16, 3H), 3.50 (d, *J* = 6.4 Hz, H-9, 2H), 2.15 – 2.21 (m, H-18, 1H), 1.92 (d, *J* = 11.5 Hz, H-19α, 2H), 1.75-1.77 (m, H-19β, 2H), 1.42 – 1.49 (m, H-20α, 2H), 1.16 – 1.28 (m, H-20β, H-21, 4H); ^{13}C NMR ($CDCl_3$, 125.8 MHz) δ: 174.2 (C-17), 146.6 (C-13), 144.2 (C-12), 134.3 (C-1), 134.2 (C-8), 131.3 (C-10), 129.4 (C-6), 127.8 (C-3), 127.0 (C-5), 125.6 (C-4), 125.0 (C-7), 123.4 (C-2), 121.3 (C-15), 114.4 (C-14), 111.3 (C-11), 55.92 (C-16), 46.3 (C-18), 39.2 (C-9), 29.8 (C-19), 25.7 (C-20, C-21).

(*E*)-*N*-(2-(3-(4-Hydroxy-3-methoxyphenyl)allyl)phenyl)cyclohexanecarboxamide **9g**. ^1H NMR ($CDCl_3$, 500 MHz) δ: 7.96 (d, *J* = 7.6 Hz, H-2′, 1H), 7.39 (br s, NH, 1H), 7.28 (t, *J* = 7.6 Hz, H-3′, 1H), 7.23 (d, *J* = 7.6 Hz, H-5′, 1H), 7.11 (t, *J* = 7.6 Hz, H-4′, 1H), 6.87 (d, *J* = 8.2 Hz, H-15′, 1H), 6.85 (s, H-11′, 1H), 6.84 (d, *J* = 8.2 Hz, H-14′, 1H), 6.41 (d, *J* = 15.9 Hz, H-9′, 1H), 6.16 (dt, *J* = 15.9, 6.2 Hz, H-8′, 1H), 5.69 (s, OH, 1H), 3.88 (s, H-16′, 3H), 3.53 (d, *J* = 6.2 Hz, H-7′, 2H), 2.15 – 2.21 (m, H-18′, 1H), 1.92 (d, *J* = 11.5 Hz, H-19α′, 2H), 1.75-1.77 (m, H-19β′, 2H), 1.42 – 1.49 (m, H-20α′, 2H), 1.16 – 1.28 (m, H-20β′, H-21′, 4H); ^{13}C NMR ($CDCl_3$, 125.8 MHz) δ: 174.2 (C-17′), 146.7 (C-12′), 145.5 (C-13′), 136.4 (C-1′), 131.8 (C-9′), 130.2 (C-5′), 129.8 (C-6′), 129.2 (C-10′), 127.5 (C-3′), 125.2 (C-8′), 124.9 (C-4′), 123.3 (C-2′), 120.0 (C-15′), 114.5 (C-14′), 107.9 (C-11′), 55.87 (C-16′), 46.5 (C-18′), 36.2 (C-7′), 29.8 (C-19′,), 25.7 (C-20′, C-21′).

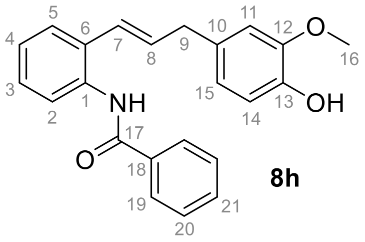
8h
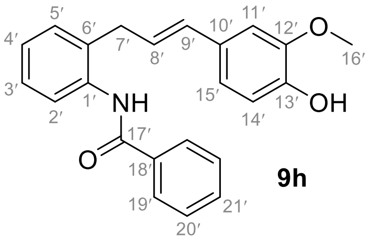
9h

Following general procedure B with *N*-(2-iodophenyl)benzamide **3h** (1.62 g, 5.00 mmol), 4-allyl-2-methoxyphenol **5** (1.23 mL, 8.00 mmol), $Pd(OAc)_2$ (11.2 mg, 50.0 µmol) and Et_3N (2.44 mL, 17.5 mmol), a 29:71 mixture of (*E*)-*N*-(2-(3-(4-hydroxy-3-methoxyphenyl)prop-1-en-1-yl)phenyl)benzamide **8h** and (*E*)-*N*-(2-(3-(4-hydroxy-3-methoxyphenyl)allyl)phenyl)benzamide **9h** was produced (1.35 g, 81%). Further partial separation by isocratic elution column chromatography (chloroform/dichloromethane, 60:40) then yielded **9h** (0.56 g, 34%).

29:71 mixture of **8h** and **9h**. Orange solid. $R_f \approx 0.21$, [UV-active, EtOAc/Hexane 20%]. IR (neat) υ: 3528 (w, OH), 3225 (w, NH), 3062 (w), 3027, 2965 (w), 1649 (m, C=O), 1516 (s), 1484 (m), 1372 (w), 1272 (s), 1247 (m), 1157 (m), 1026 (m, C−O), 866 (w), 819 (w), 793 (w), 754 (s) cm^{-1}; HRMS (+ESI) $[M + H]^+$: 360.1596, $C_{23}H_{22}NO_3$ requires 360.1600.

(*E*)-*N*-(2-(3-(4-Hydroxy-3-methoxyphenyl)prop-1-en-1-yl)phenyl)benzamide **8h**. ^{1}H NMR ($CDCl_3$, 500 MHz) δ: 6.68-8.22 (m, NH, H-3, H-4, H-5, H-11, H-14, H-15, H-19, H-20, H-21, 12H), 8.04 (d, J = 7.5 Hz, H-2, 1H), 6.47-6.50 (m, H-7, 1H), 6.30-6.37 (m, H-8, 1H), 5.50 (s, OH, 1H), 3.88 (s, H-16, 3H), 3.51 (d, J = 6.0 Hz, H-9, 2H); ^{13}C NMR ($CDCl_3$, 125.8 MHz), characteristic signals, δ: 134.7 (C-8), 134.3 (C-1), 128.8 (C-20), 127.9 (C-3), 125.0 (C-4), 123.2 (C-2), 121.3 (C-15), 114.3 (C-14), 111.2 (C-11), 55.85 (C-16), 39.2 (C-9).

(*E*)-*N*-(2-(3-(4-Hydroxy-3-methoxyphenyl)allyl)phenyl)benzamide **9h**. ^{1}H NMR ($CDCl_3$, 500 MHz) δ: 8.22 (s, NH, 1H), 8.12 (d, J = 7.5 Hz, H-2′, 1H), 7.77 (d, J = 7.5 Hz, H-19′, 2H), 7.51 (t, J = 7.5 Hz, H-21′, 1H), 7.36 (t, J = 7.5 Hz, H-3′, H-20′, 3H), 7.29 (d, J = 7.5 Hz, H-5′, 1H), 7.18 (t, J = 7.5 Hz, H-4′, 1H), 6.87 (s, H-11′, 1H), 6.86 (AB system, δ_A = 6.84, δ_B = 6.88, J_{AB} = 8.5 Hz, H15′, H14′, 2H), 6.46 (br d, J = 16.0 Hz, H-9′, 1H), 6.22 (dt, J = 16.0, 5.5 Hz, H-8′, 1H), 5.66 (s, OH, 1H), 3.88 (s, H-16′, 3H), 3.61 (d, J = 5.5 Hz, H-7′, 2H); ^{13}C NMR ($CDCl_3$, 125.8 MHz) δ: 165.4 (C-17′), 146.7 (C-12′), 145.6 (C-13′), 136.7 (C-1′), 134.8 (C-18′), 132.2 (C-9′), 131.8 (C-21′), 130.4 (C-5′), 129.9 (C-6′), 128.9 (C-10′), 128.8 (C-20′), 127.8 (C-3′), 127.1 (C-19′), 125.2 (C-8′), 125.0 (C-4′), 123.2 (C-2′), 120.2 (C-15′), 114.5 (C-14′), 107.9 (C-11′), 55.87 (C-16′), 36.8 (C-7′).

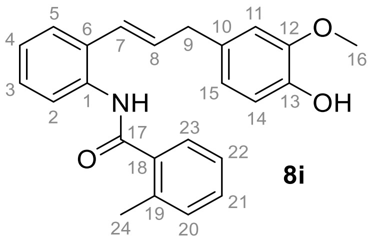
8i

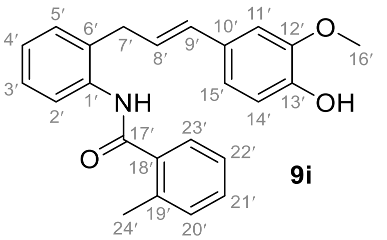
9i

Following general procedure B with *N*-(2-iodophenyl)-2-methylbenzamide **3i** (1.69 g, 5.00 mmol), 4-allyl-2-methoxyphenol **5** (1.23 mL, 8.00 mmol), $Pd(OAc)_2$ (11.2 mg, 50.0 μmol) and Et_3N (2.44 mL, 17.5 mmol), a 22:78 mixture of (*E*)-*N*-(2-(3-(4-hydroxy-3-methoxyphenyl)prop-1-en-1-yl)phenyl)-2-methylbenzamide **8i** and (*E*)-*N*-(2-(3-(4-hydroxy-3-methoxyphenyl)allyl)phenyl)-2-methylbenzamide **9i** was produced (1.50 g, 79%).

22:78 mixture of **8i** and **9i**. Brownish-white solid. $R_f \approx 0.18$, [UV-active, EtOAc/Hexane 20%]. IR (neat) υ: 3388 (w, OH), 3271 (w, NH), 3024 (w), 2965 (w), 1646 (s, C=O), 1515 (s), 1451 (m), 1379 (w), 1267 (s), 1236 (s), 1157 (w), 1032 (m, C–O), 866 (w), 834 (w), 790 (w), 751 (s) cm^{-1}; HRMS (+ESI) $[M + H]^+$: 374.1743, $C_{24}H_{24}NO_3$ requires 374.1756.

(*E*)-*N*-(2-(3-(4-Hydroxy-3-methoxyphenyl)prop-1-en-1-yl) phenyl)-2-methylbenzamide **8i**. ^{1}H NMR ($CDCl_3$, 500 MHz) δ: 8.00 (d, *J* = 8.0 Hz, H-2, 1H), 7.74 (br s, NH, 1H), 7.42 (d, *J* = 8.0 Hz, H-5, 1H), 7.36-7.40 (m, H-20, H-21, 2H), 7.35 (d, *J* = 7.5 Hz, H-23, 1H), 7.28 (t, *J* = 8.0 Hz, H-3, 1H), 7.15-7.21 (m, H-4, H-22, 2H), 6.68 (s, H-11, 1H), 6.72 (d, *J* = 8.2 Hz, H-14, H-15, 2H), 6.46 (d, *J* = 15.9 Hz, H-7, 1H), 6.30 (dt, *J* = 15.9, 6.3 Hz, H-8, 1H), 5.50 (s, OH, 1H), 3.80 (s, H-16, 3H), 3.48 (d, *J* = 6.3 Hz, H-9, 2H), 2.50 (s, H-24, 3H); ^{13}C NMR ($CDCl_3$, 125.8 MHz) δ: 167.9 (C-17), 146.3 (C-12), 144.3 (C-13), 136.7 (C-18), 136.4 (C-19), 134.3 (C-8), 134.1 (C-1), 131.3 (C-21), 131.2 (C-10), 130.24 (C-20), 130.0 (C-6), 127.9 (C-3), 127.1 (C-5), 126.5 (C-23), 126.1 (C-22), 125.8 (C-7), 125.4 (C-4), 125.0 (C-2), 121.2 (C-15), 114.37 (C-14), 111.2 (C-11), 55.7 (C-16), 39.1 (C-9), 19.9 (C-24).

(*E*)-*N*-(2-(3-(4-Hydroxy-3-methoxyphenyl)allyl)phenyl)-2-methylbenzamide **9i**. ^{1}H NMR ($CDCl_3$, 500 MHz) δ: 8.16 (d, *J* = 7.9 Hz, H-2′, 1H), 7.74 (br s, NH, 1H), 7.36-7.40 (m, H-20′, H-21′, 2H), 7.32 (d, *J* = 7.5 Hz, H-23′, 1H), 7.28 (t, *J* = 7.9 Hz, H-3′, 1H), 7.23 (d, *J* = 7.9 Hz, H-5′, 1H), 7.17 (t, *J* = 7.5 Hz, H-22′, 1H), 7.09 (t, *J* = 7.9 Hz, H-4′, 1H), 6.76 (s, H-11′, 1H), 6.83 (d, *J* = 8.2 Hz, H-14′, H-15′, 2H), 6.28 (d, *J* = 15.9 Hz, H-9′, 1H), 6.14 (dt, *J* = 15.9, 6.2 Hz, H-8′, 1H), 5.61 (s, OH, 1H), 3.86 (s, H-16′, 3H), 3.56 (d, *J* = 6.2 Hz, H-7′, 2H), 2.47 (s, H-24′, 3H); ^{13}C NMR ($CDCl_3$, 125.8 MHz) δ: 167.9 (C-17′), 146.6 (C-12′), 145.5 (C-13′), 136.7 (C-18′), 136.5 (C-19′), 136.2 (C-1′), 131.8 (C-9′), 131.3 (C-21′), 130.3 (C-5′), 130.15 (C-20′), 130.0 (C-6′), 129.0 (C-10′), 127.6 (C-3′), 126.7 (C-23′), 125.9 (C-22′), 125.5 (C-4′), 125.2 (C-8′), 124.8 (C-2′), 119.9 (C-15′), 114.39 (C-14′), 108.0 (C-11′), 55.8 (C-16′), 36.3 (C-7′), 19.9 (C-24′).

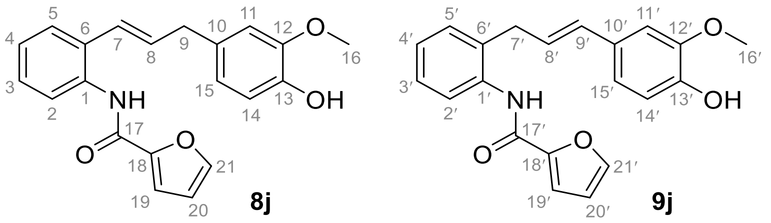

Following general procedure B with *N*-(2-iodophenyl)furan-2-carboxamide **3j** (1.10 g, 3.23 mmol), 4-allyl-2-methoxyphenol **5** (801 μL, 5.17 mmol), $Pd(OAc)_2$ (7.26 mg, 30.0 μmol) and Et_3N (1.58 mL, 11.3 mmol), a 26:74 mixture of (*E*)-*N*-(2-(3-(4-hydroxy-3-methoxyphenyl)prop-1-en-1-yl)phenyl)furan-2-carboxamide **8j** and (*E*)-*N*-(2-(3-(4-hydroxy-3-methoxyphenyl)allyl)phenyl)furan-2-carboxamide **9j** was produced (1.04 g, 83%).

26:74 mixture of **8j** and **9j**. Yellow solid. $R_f \approx 0.26$, [UV-active, EtOAc/Hexane 30%]. IR (neat) υ: 3416 (w, OH), 3216 (w, NH), 3039 (w), 2954 (w), 1617 (m, C=O), 1511 (m), 1460 (m), 1350 (m), 1266 (m), 1227 (m), 1153 (m), 1035 (m, C−O), 885 (w), 826 (w), 785 (w), 758 (s) cm^{-1}; HRMS (-ESI) $[M - H]^-$: 348.1245, $C_{21}H_{19}NO_4$ requires 348.1236.

(*E*)-*N*-(2-(3-(4-Hydroxy-3-methoxyphenyl)prop-1-en-1-yl)phenyl)furan-2-carboxamide **8j**. ^{1}H NMR ($CDCl_3$, 500 MHz) δ: 8.43 (br s, NH, 1H), 8.12 (d, *J* = 7.9 Hz, H-21, 1H), 7.39 (d, *J* = 7.0 Hz, H-2, 1H), 7.29 – 7.33 (m, H-3, H-5, 2H), 7.15 – 7.20 (m, H-4, H-19, 2H), 6.78 (s, H-11, 1H), 6.75-6.77 (m, H-14, H-15, 2H), 6.55-6.58 (m, H-7, 1H), 6.54 (d, *J* = 7.9 Hz, H-20, 1H), 6.30-6.34 (m, H-8, 1H), 5.52 (s, OH, 1H), 3.88 (s, H-16, 3H), 3.54 (d, *J* = 6.0 Hz, H-9, 2H).

(*E*)-*N*-(2-(3-(4-Hydroxy-3-methoxyphenyl)allyl)phenyl)furan-2-carboxamide **9j**. ^{1}H NMR ($CDCl_3$, 500 MHz) δ: 8.43 (br s, NH, 1H), 8.12 (d, *J* = 7.9 Hz, H-21′, 1H), 7.29 – 7.33 (m, H-2′, H-3′, H-5′, 3H), 7.15 – 7.20 (m, H-4′, H-19′, 2H), 6.89 (s, H-11′, 1H), 6.87 (d, *J* = 8.2 Hz, H-15′, 1H), 6.86 (d, *J* = 8.2 Hz, H-14′, 1H), 6.55 (d, *J* = 15.9 Hz, H-9′, 1H), 6.54 (d, *J* = 7.9 Hz, H-20′, 1H), 6.18 (dt, *J* = 15.9, 6.2 Hz, H-8′, 1H), 5.62 (s, OH, 1H), 3.88 (s, H-16′, 3H), 3.62 (d, *J* = 6.2 Hz, H-7′, 2H); ^{13}C NMR ($CDCl_3$, 125.8 MHz) δ: 156.1 (C-17′), 148.0 (C-18′), 146.7 (C-12′), 145.5 (C-13′), 144.1 (C-21′), 136.0 (C-1′), 132.3 (C-9′), 130.3 (C-5′), 130.0 (C-6′), 129.4 (C-10′), 127.7 (C-3′), 125.1 (C-4′), 124.7 (C-8′), 122.9 (C-2′), 120.2 (C-15′), 115.1 (C-19′), 114.3 (C-14′), 112.5 (C-20′), 107.7 (C-11′), 55.9 (C-16′), 36.6 (C-7′).

4. NMR Spectra

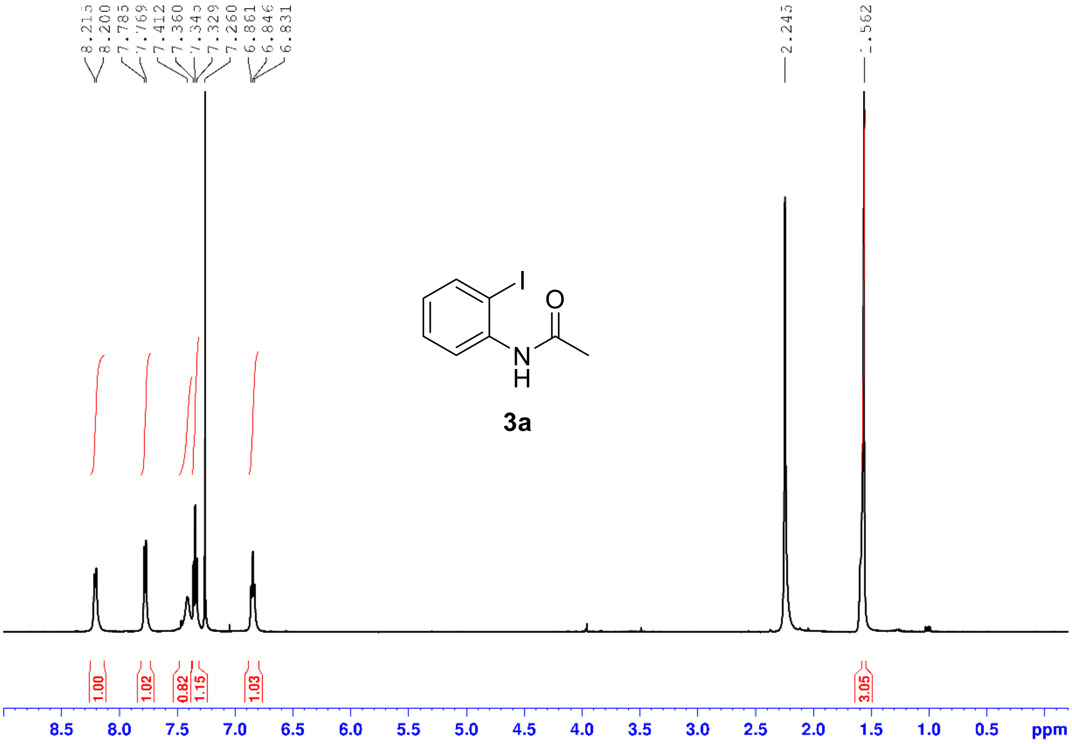

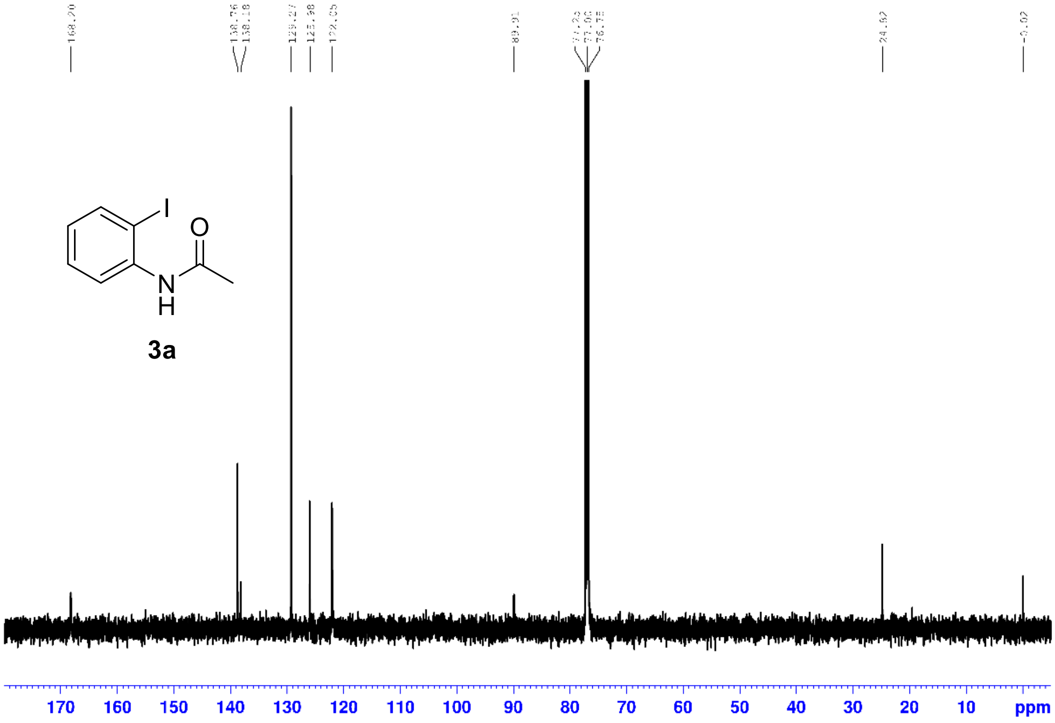

Figure S1: 500 MHz ^{1}H- and 126 MHz ^{13}C-NMR spectra of compound **3a** in $CDCl_3$.

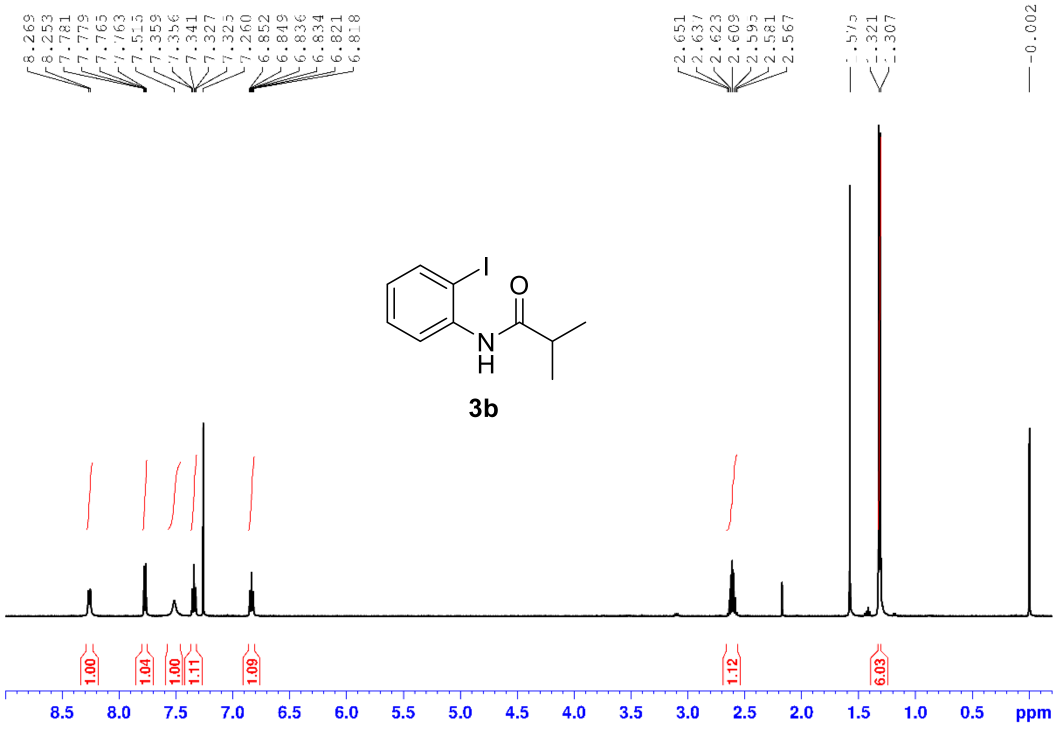

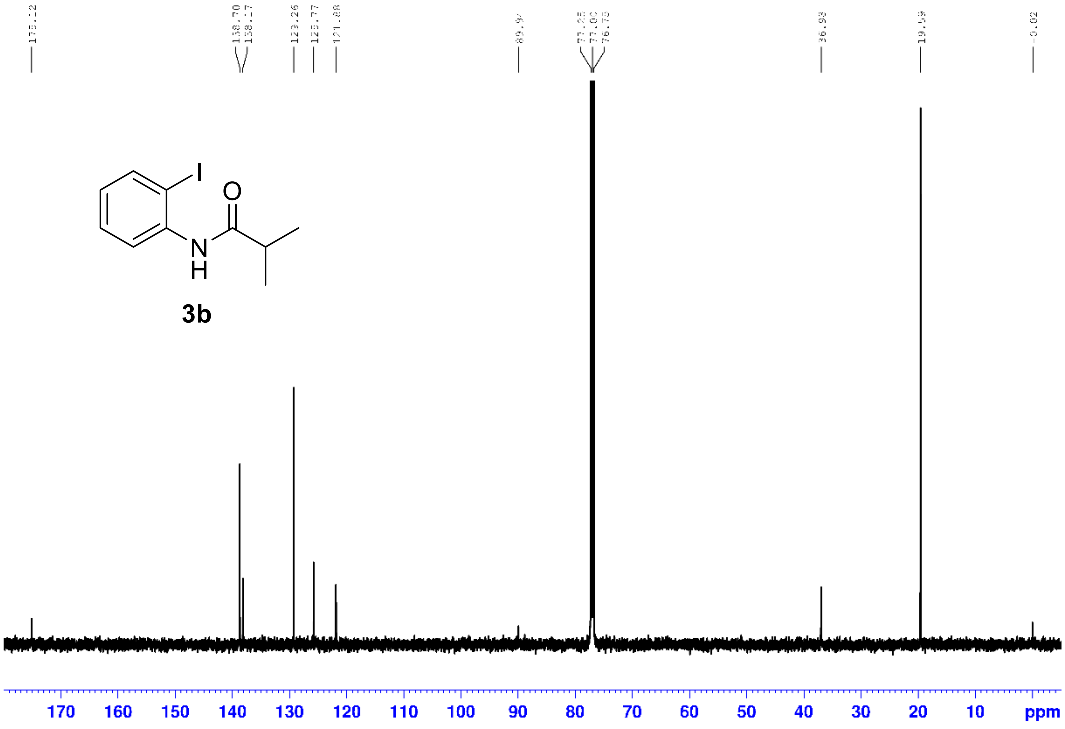

Figure S2: 500 MHz ^{1}H- and 126 MHz ^{13}C-NMR spectra of compound **3b** in $CDCl_3$.

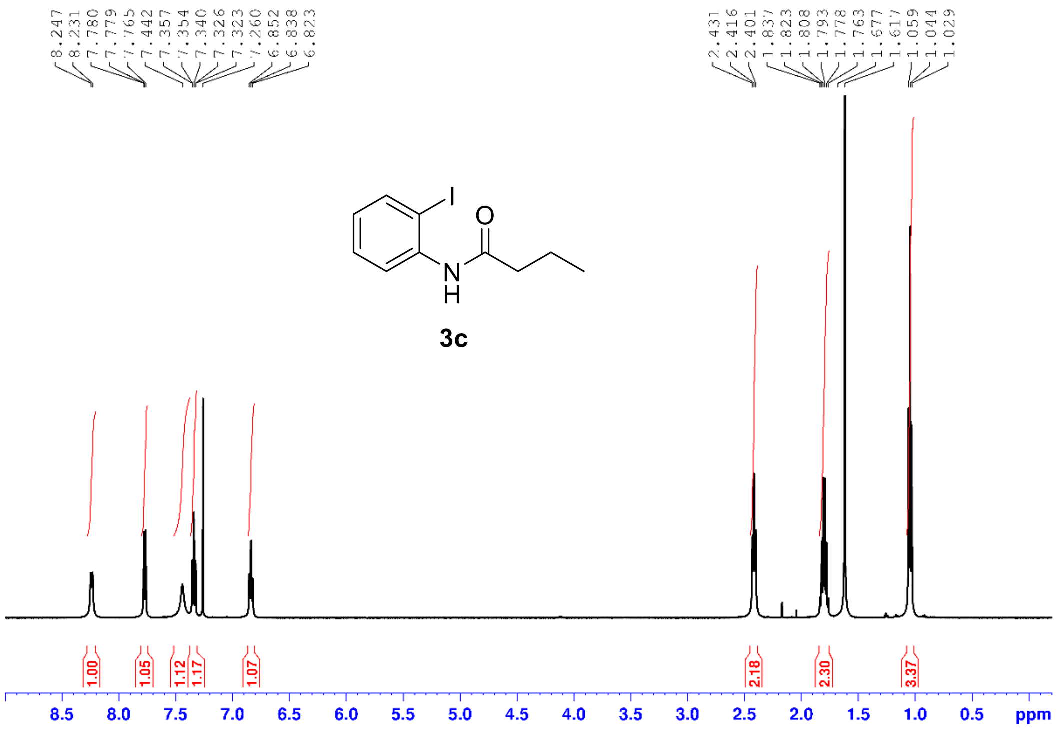

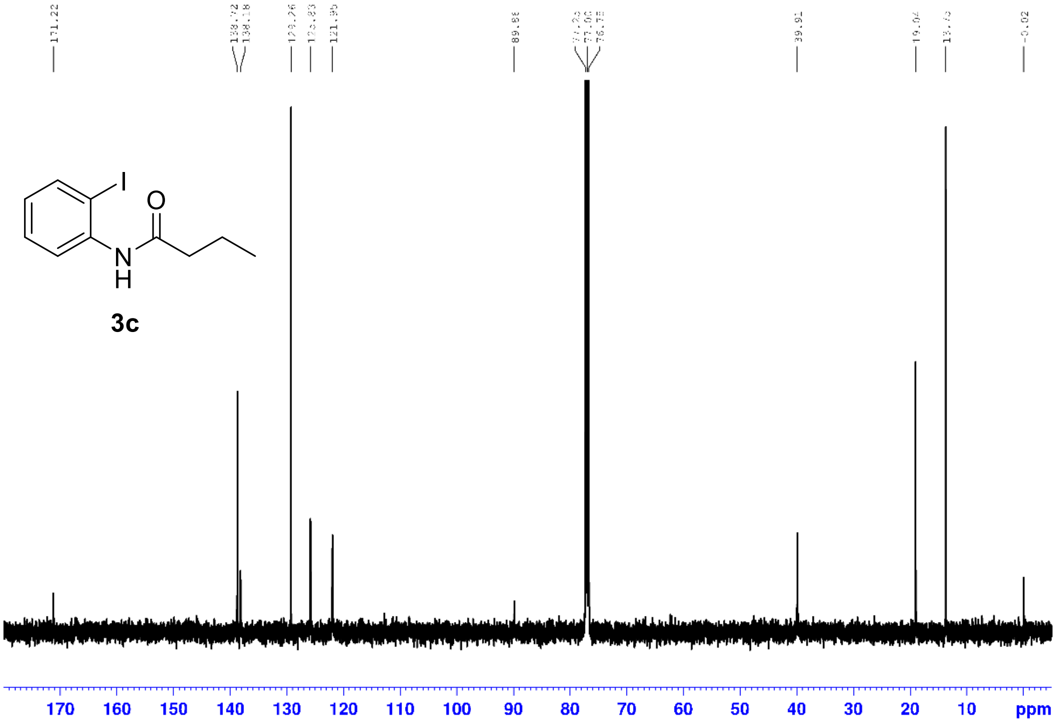

Figure S3: 500 MHz ^{1}H- and 126 MHz ^{13}C-NMR spectra of compound **3c** in $CDCl_3$.

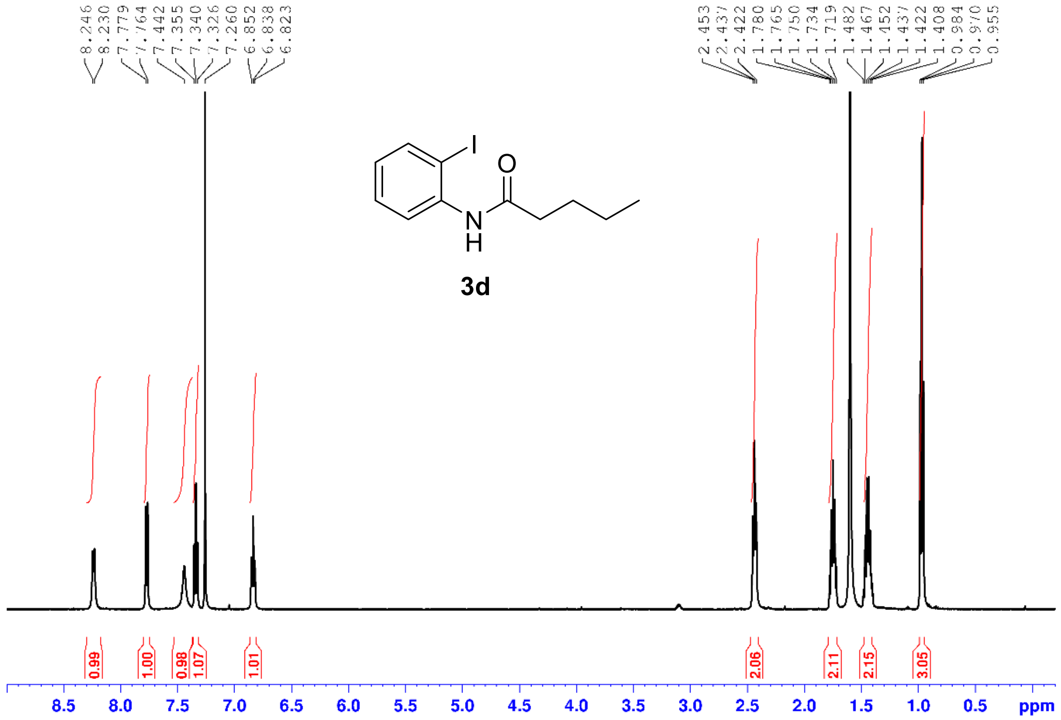

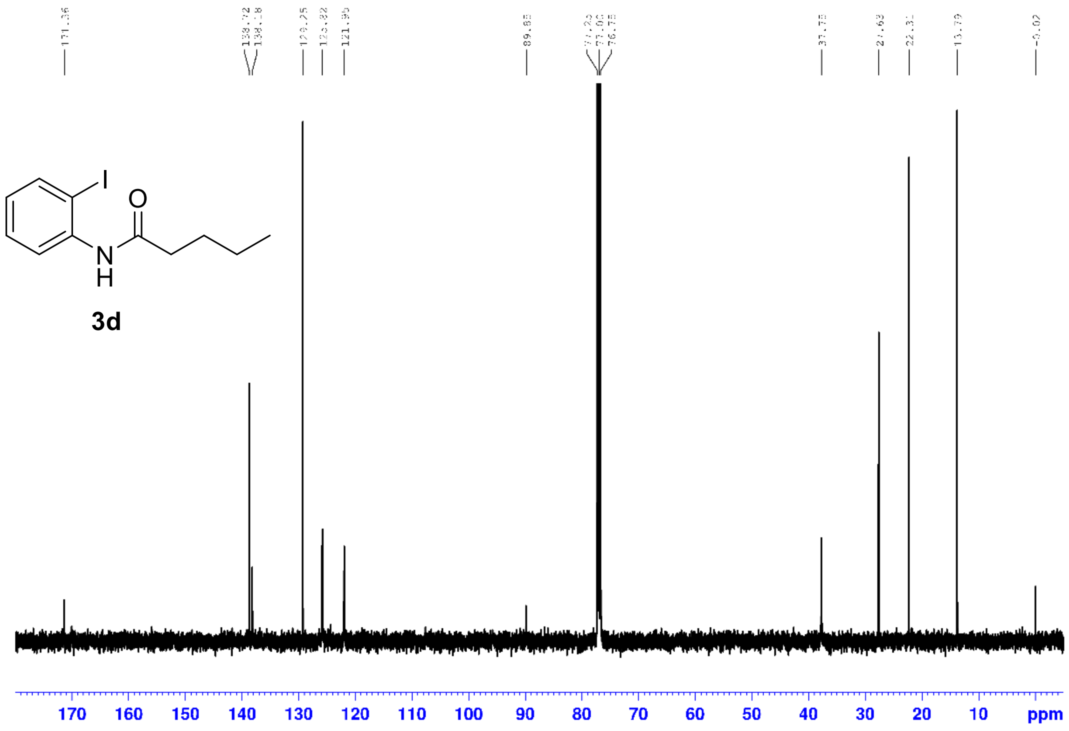

Figure S4: 500 MHz ^{1}H- and 126 MHz ^{13}C-NMR spectra of compound **3d** in $CDCl_3$.

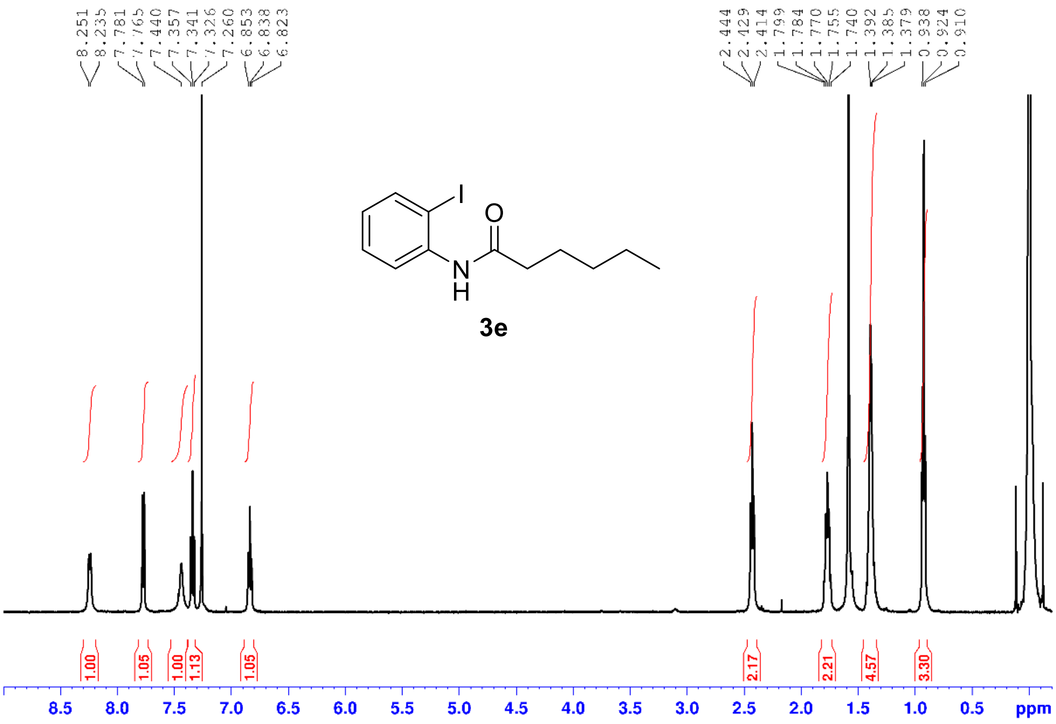

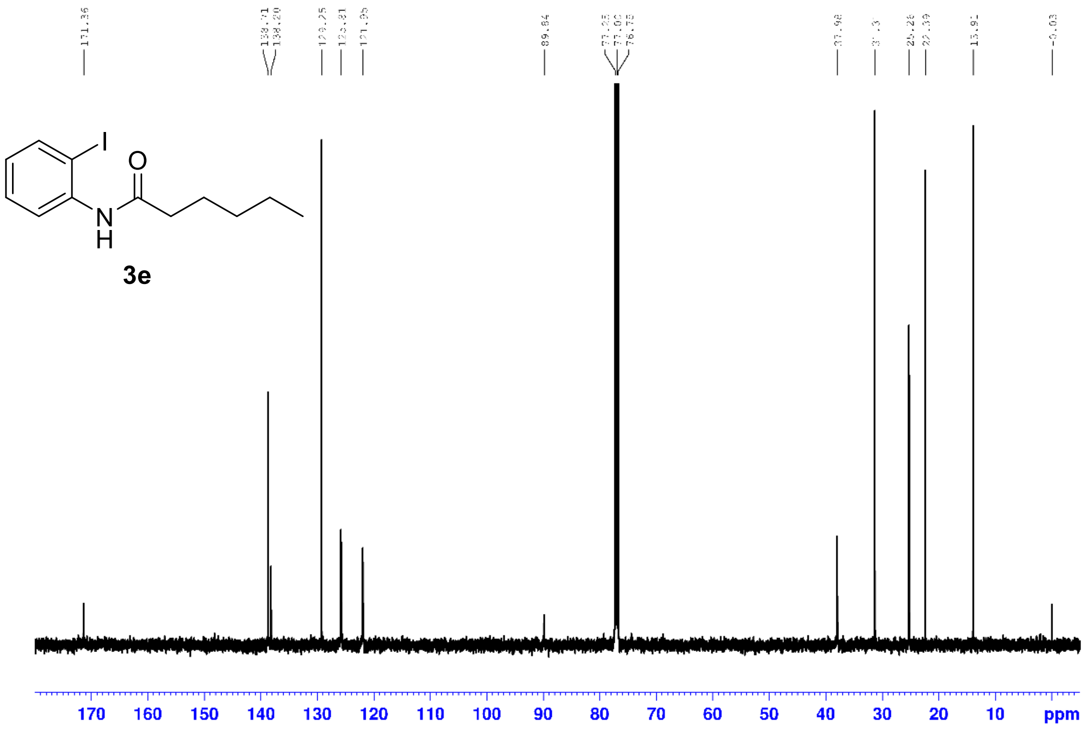

Figure S5: 500 MHz ^{1}H- and 126 MHz ^{13}C-NMR spectra of compound **3e** in $CDCl_3$.

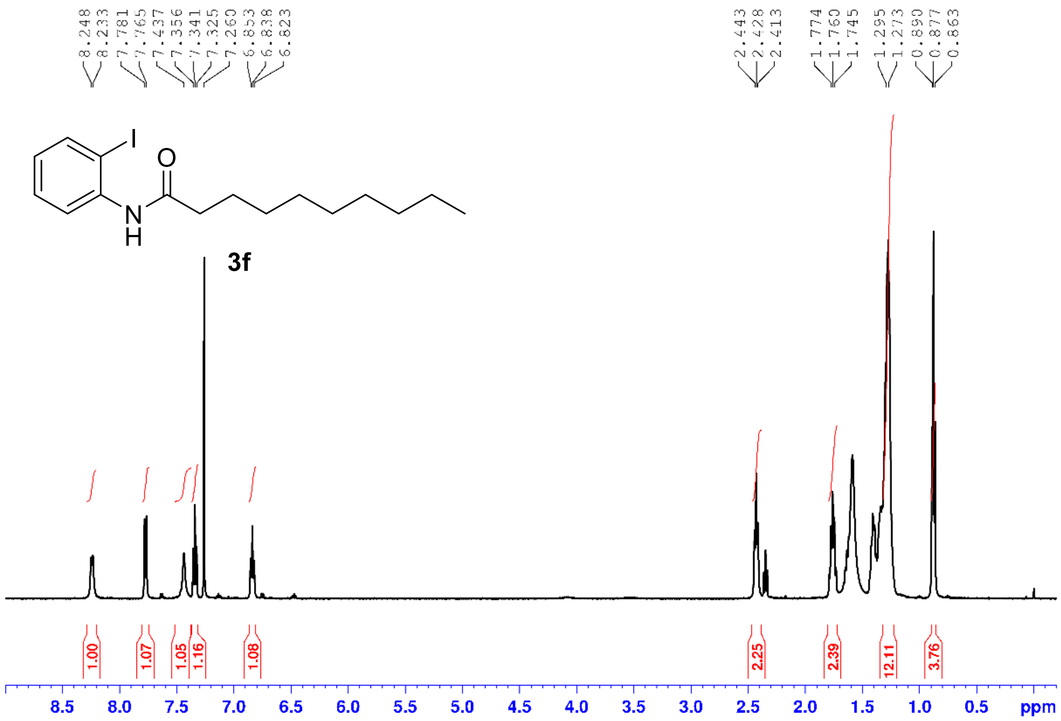

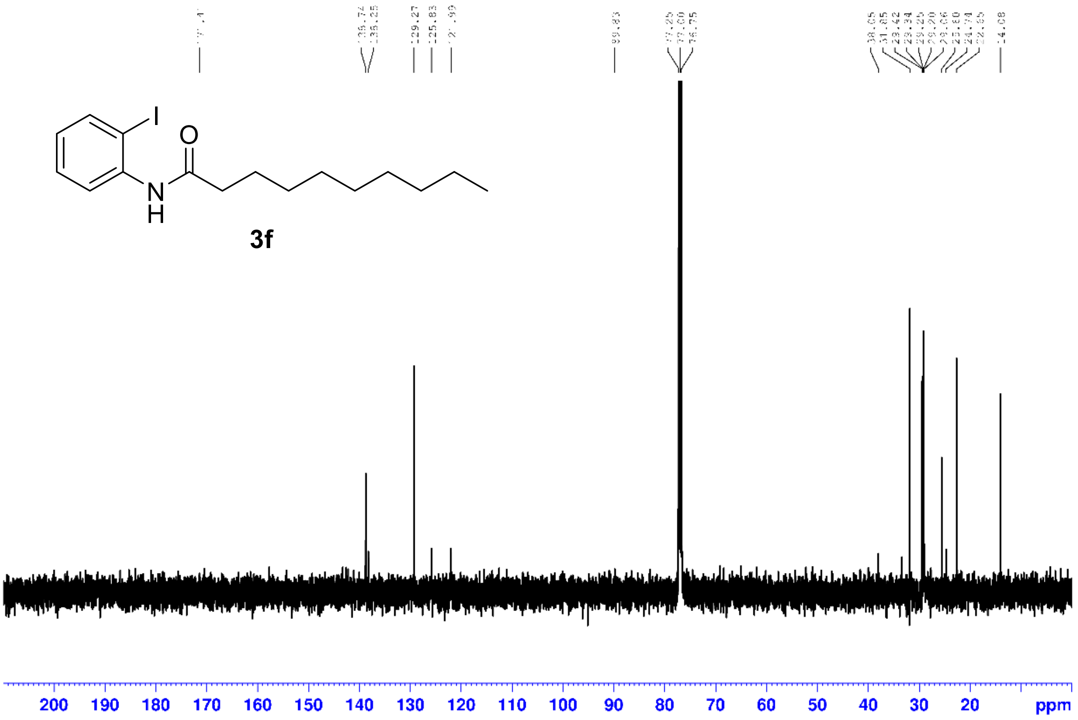

Figure S6: 500 MHz ^{1}H- and 126 MHz ^{13}C-NMR spectra of compound **3f** in $CDCl_3$.

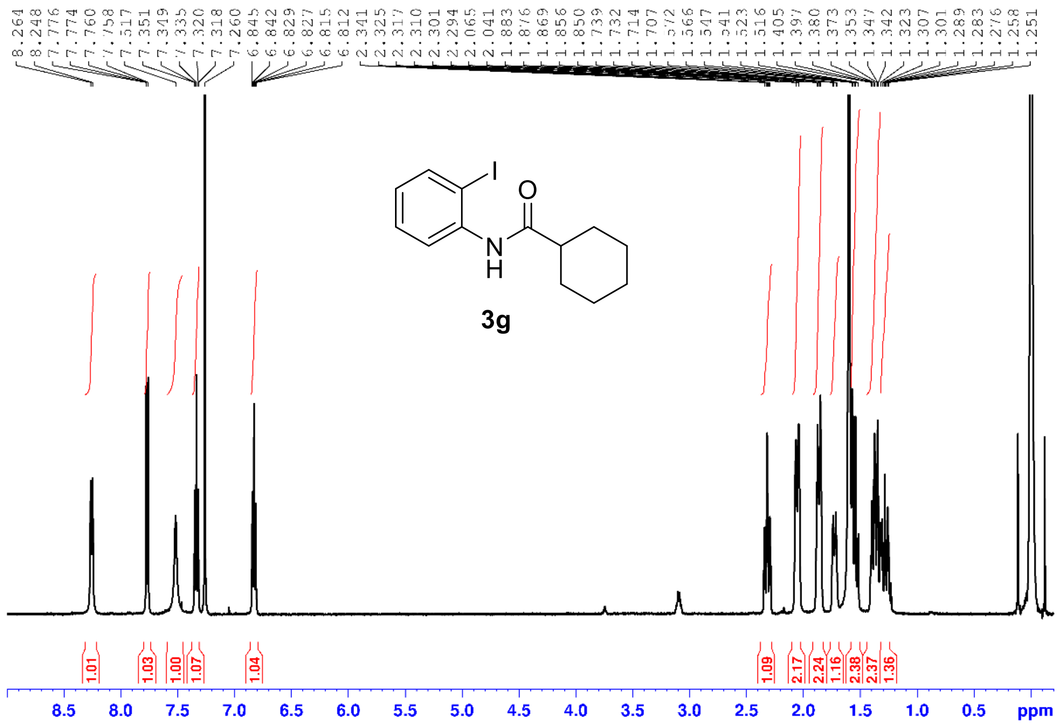

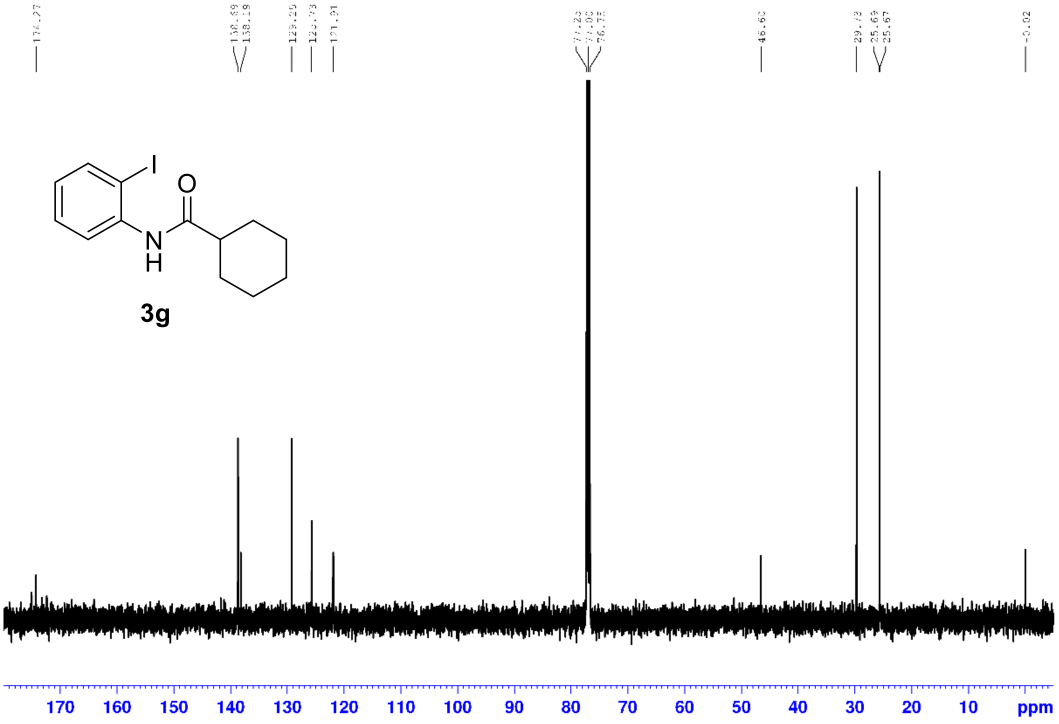

Figure S7: 500 MHz ^{1}H- and 126 MHz ^{13}C-NMR spectra of compound **3g** in $CDCl_3$.

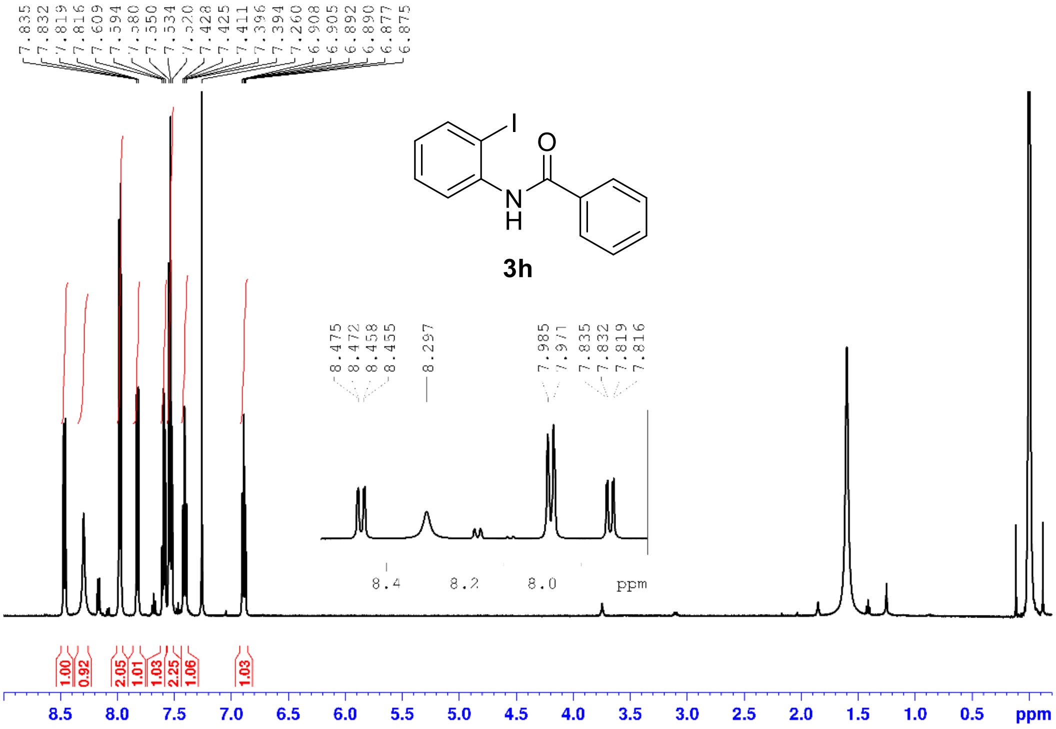

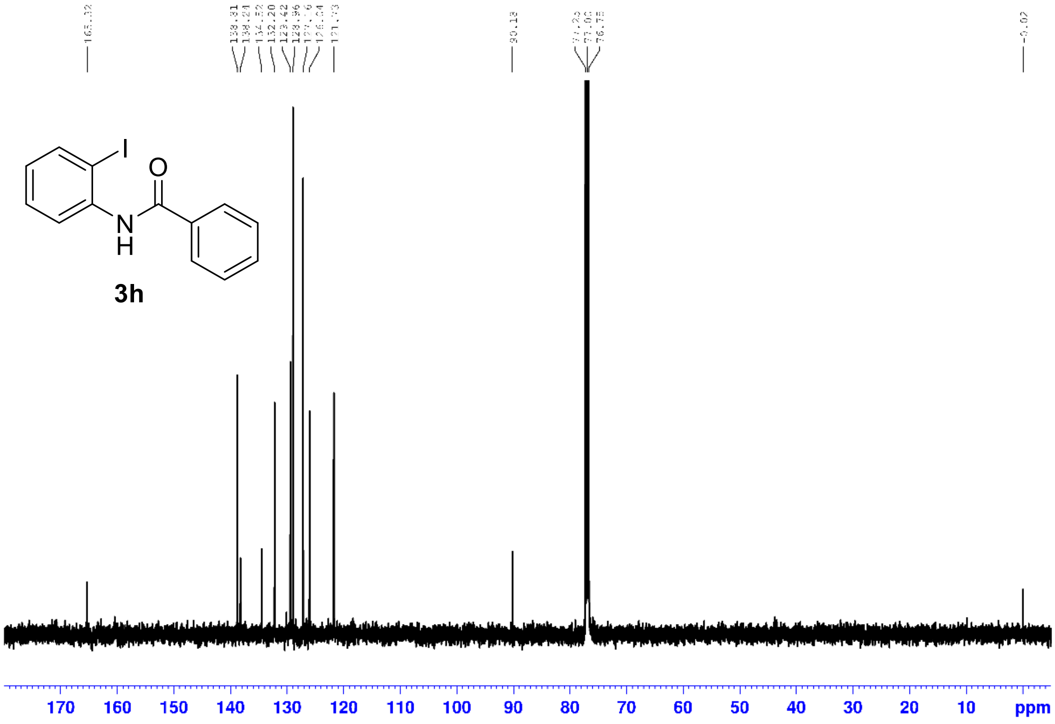

Figure S8: 500 MHz ^{1}H- and 126 MHz ^{13}C-NMR spectra of compound **3h** in $CDCl_3$.

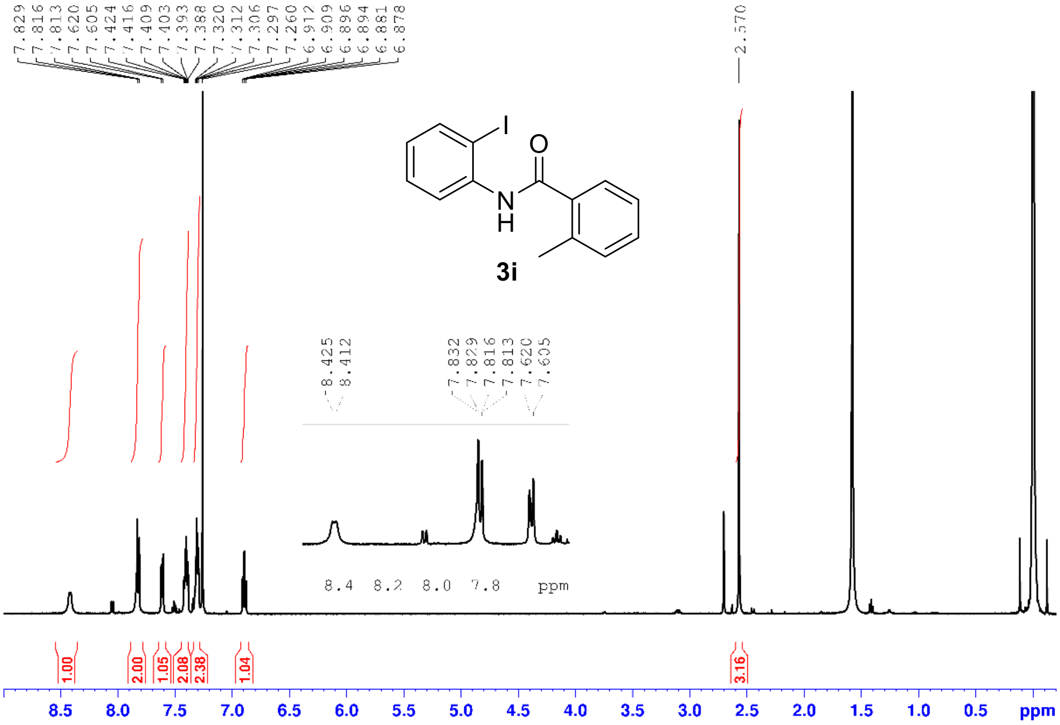

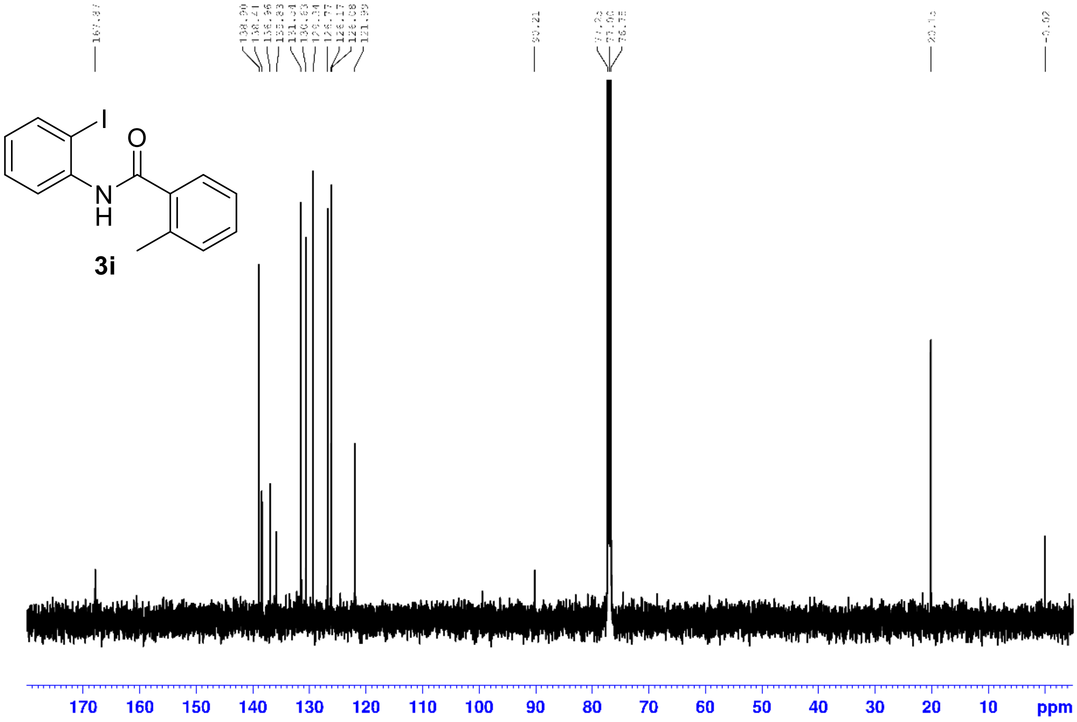

Figure S9: 500 MHz ^{1}H- and 126 MHz ^{13}C-NMR spectra of compound **3i** in $CDCl_3$.

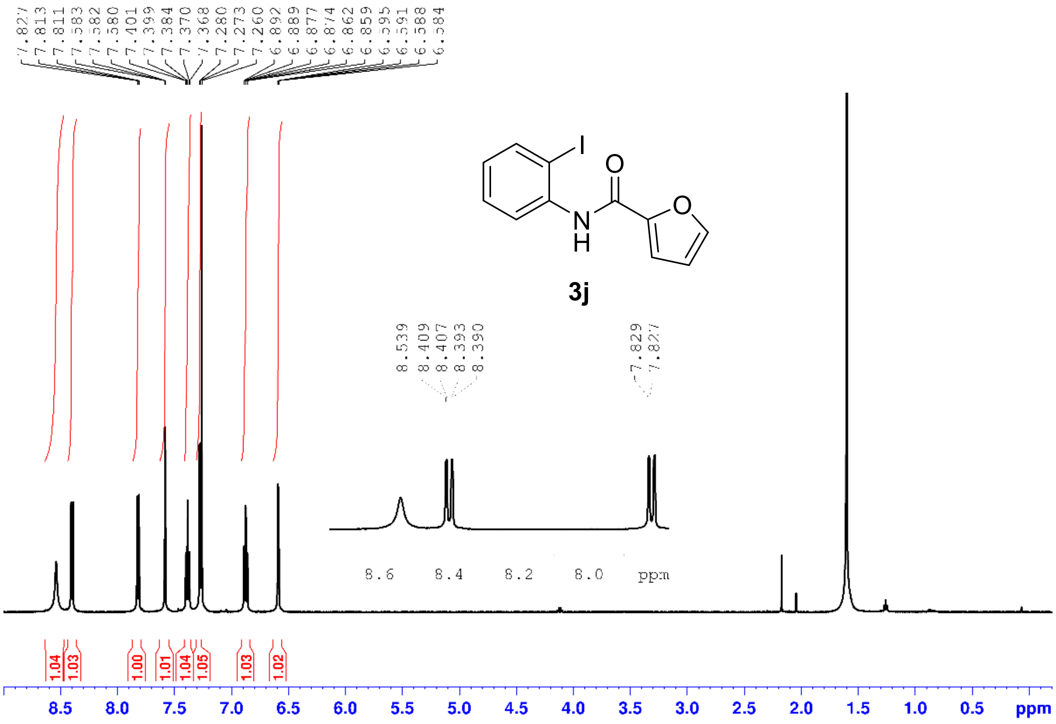

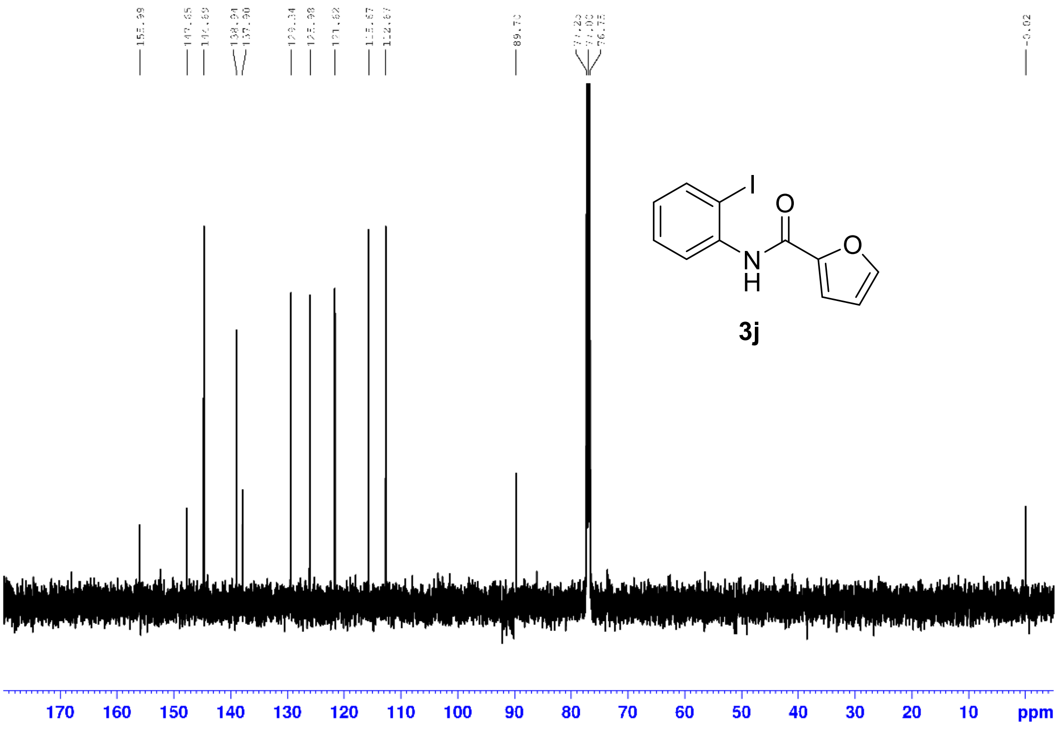

Figure S10: 500 MHz ^{1}H- and 126 MHz ^{13}C-NMR spectra of compound **3j** in $CDCl_3$.

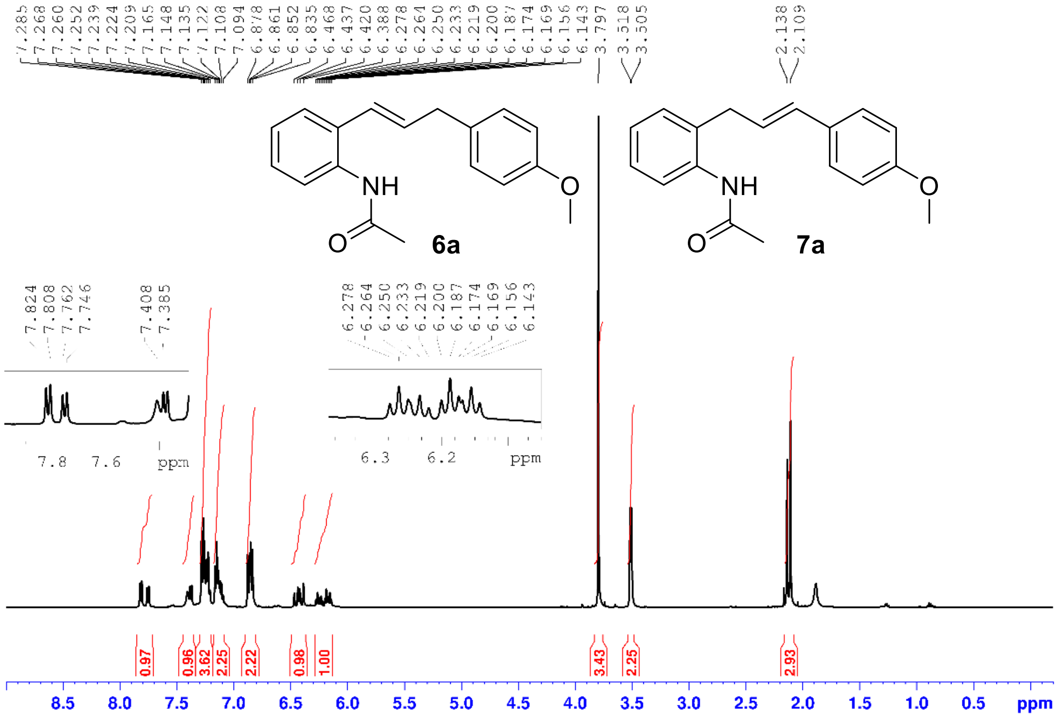

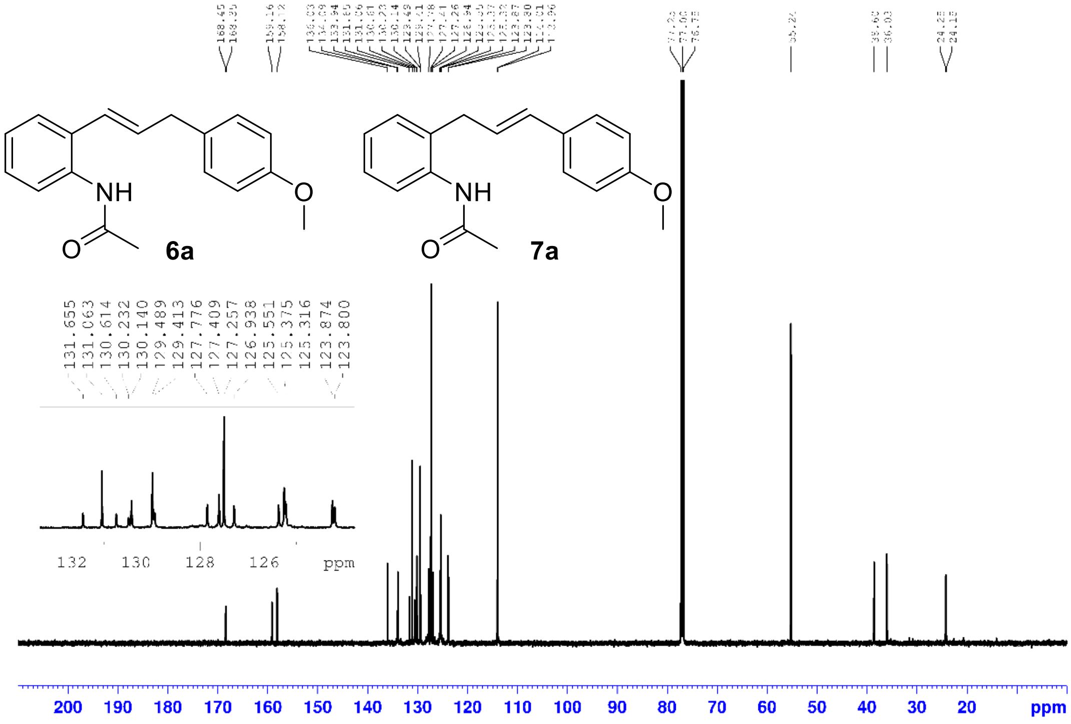

Figure S11: 500 MHz ^{1}H- and 126 MHz ^{13}C-NMR spectra of compound **6a,7a** in $CDCl_3$.

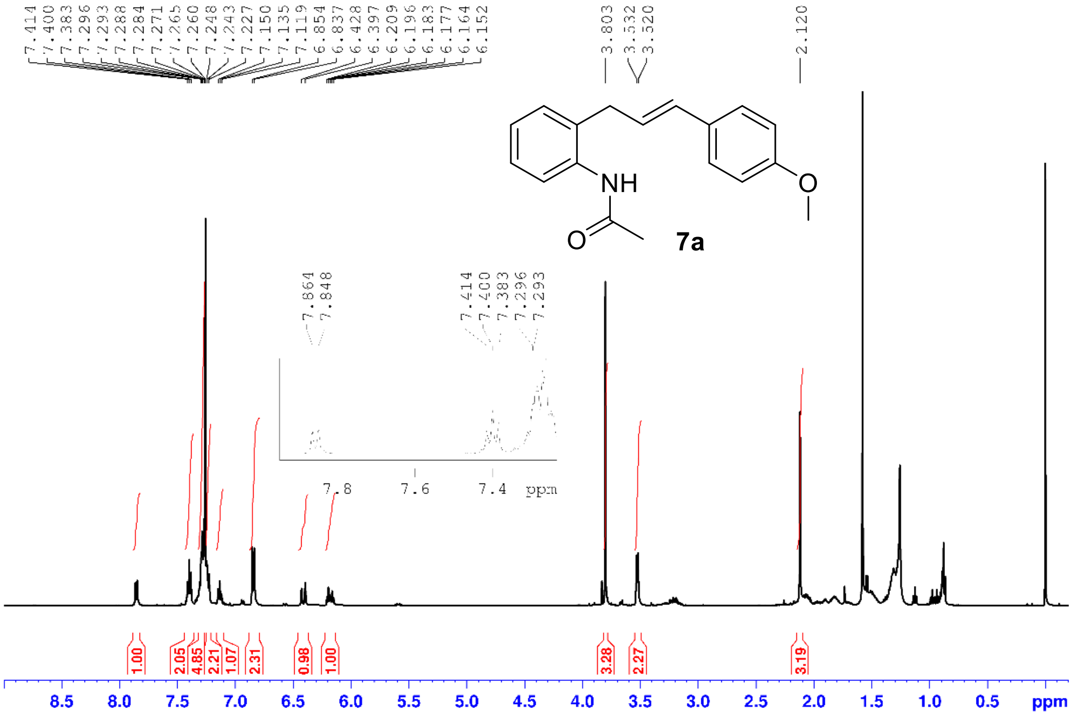

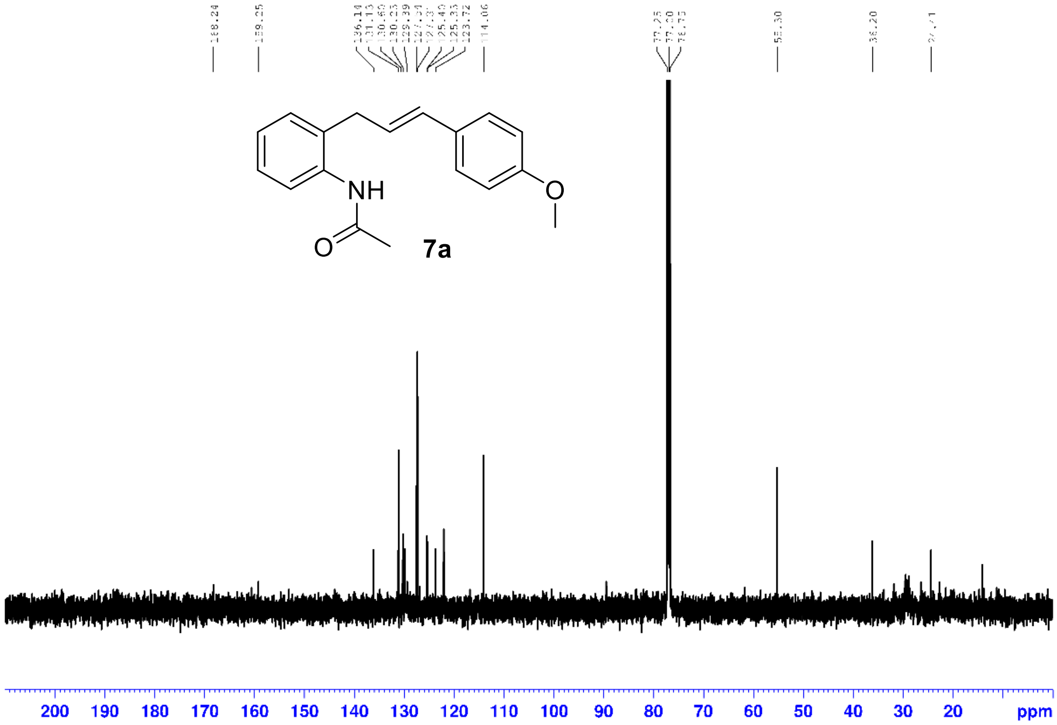

Figure S12: 500 MHz ^{1}H- and 126 MHz ^{13}C-NMR spectra of compound **7a** in $CDCl_3$.

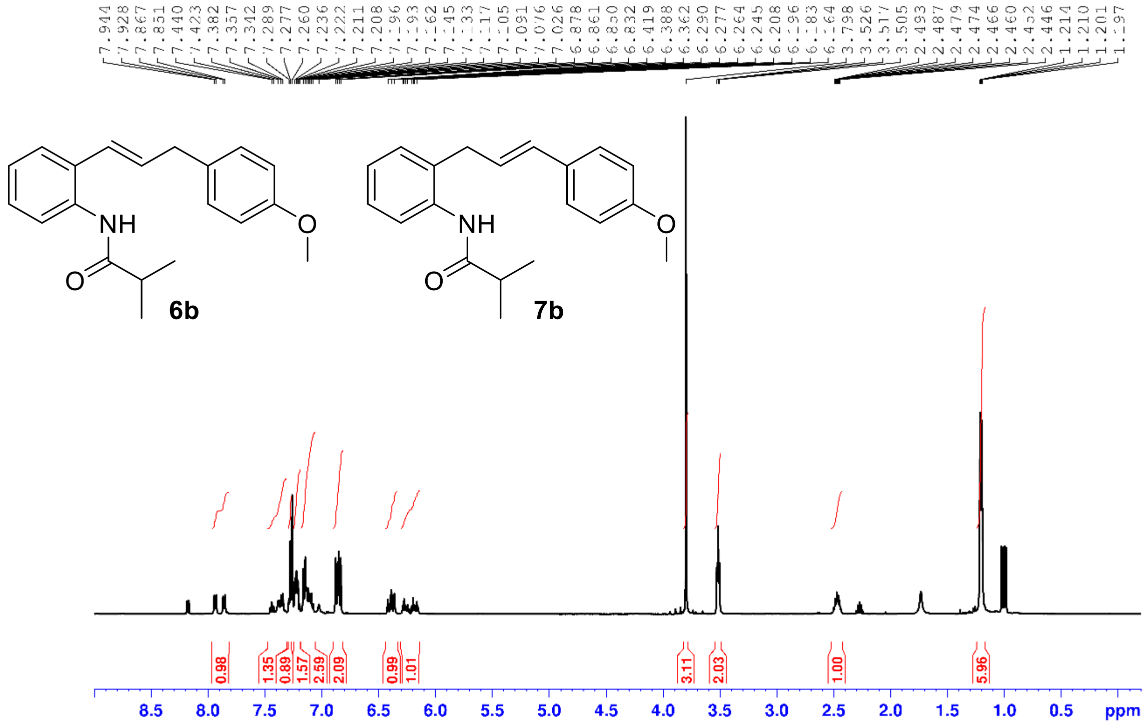

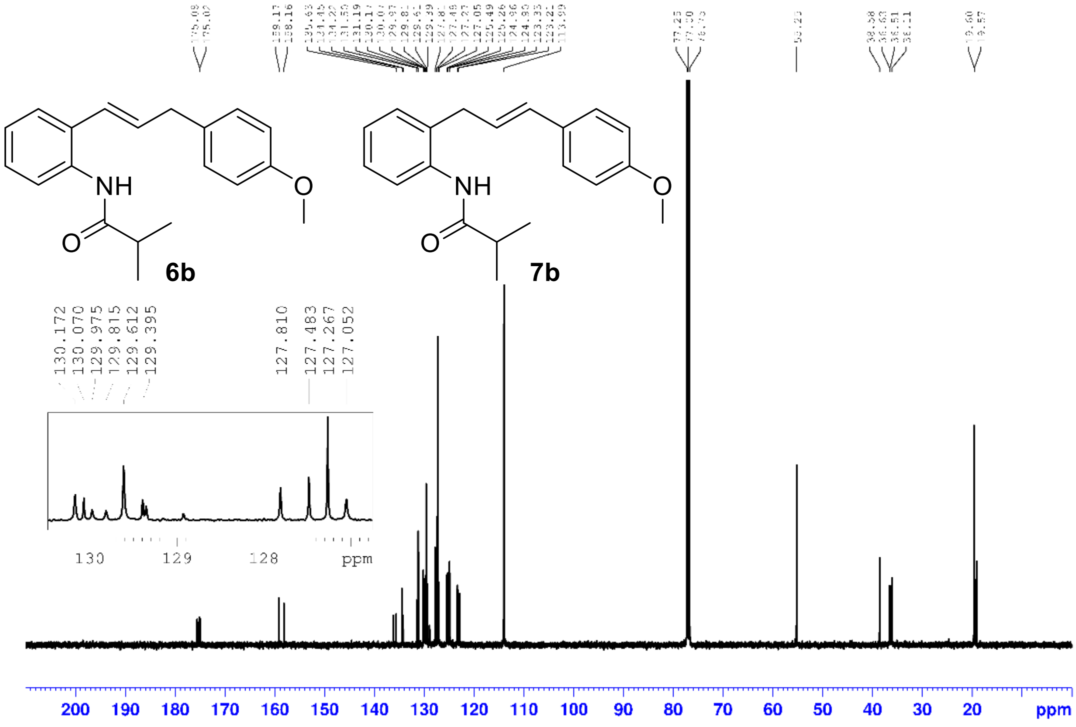

Figure S13: 500 MHz ^{1}H- and 126 MHz ^{13}C-NMR spectra of compound **6b,7b** in $CDCl_3$.

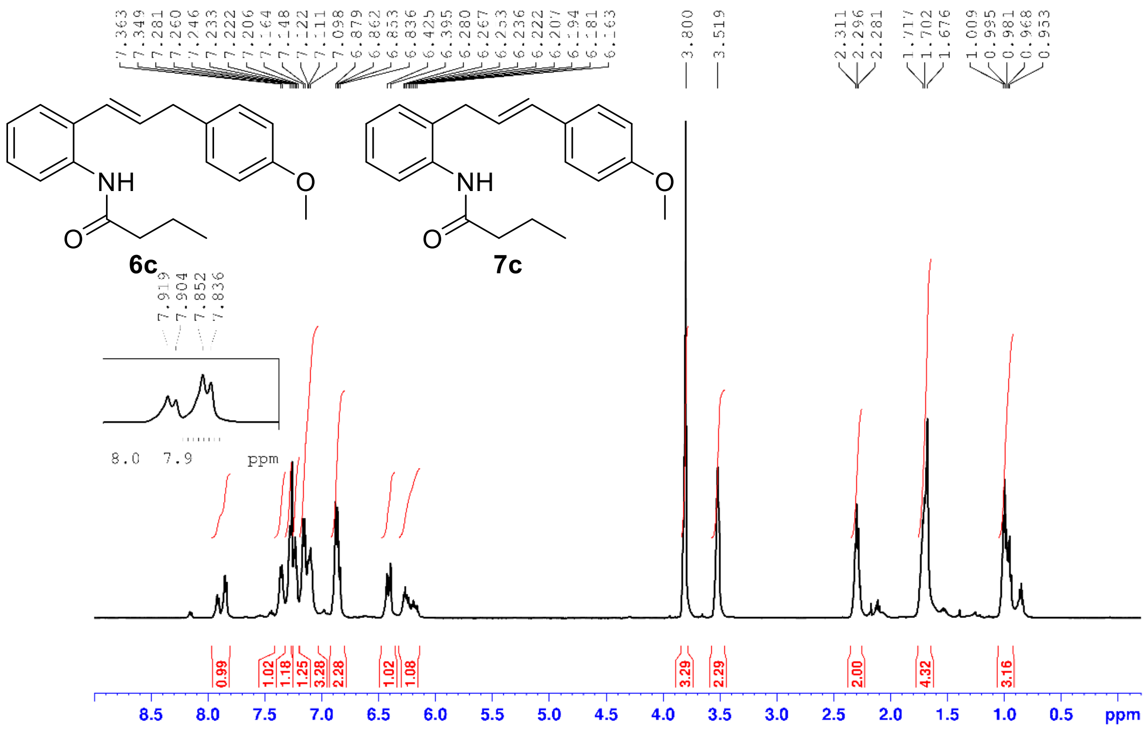

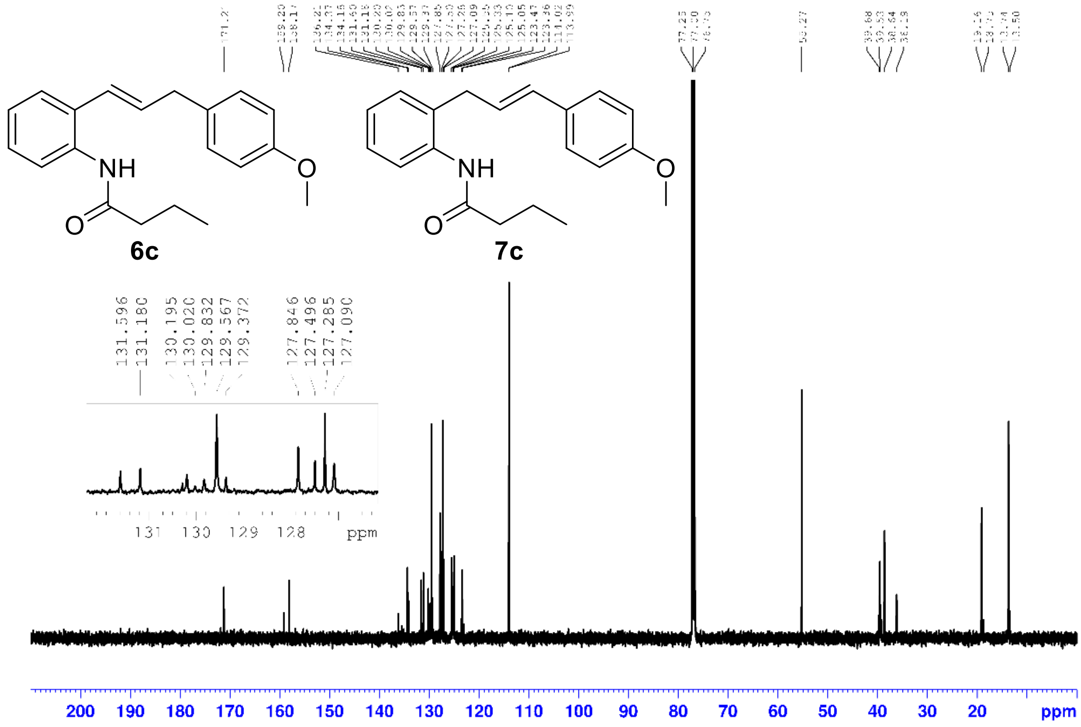

Figure S14: 500 MHz ^{1}H- and 126 MHz ^{13}C-NMR spectra of compound **6c,7c** in $CDCl_3$.

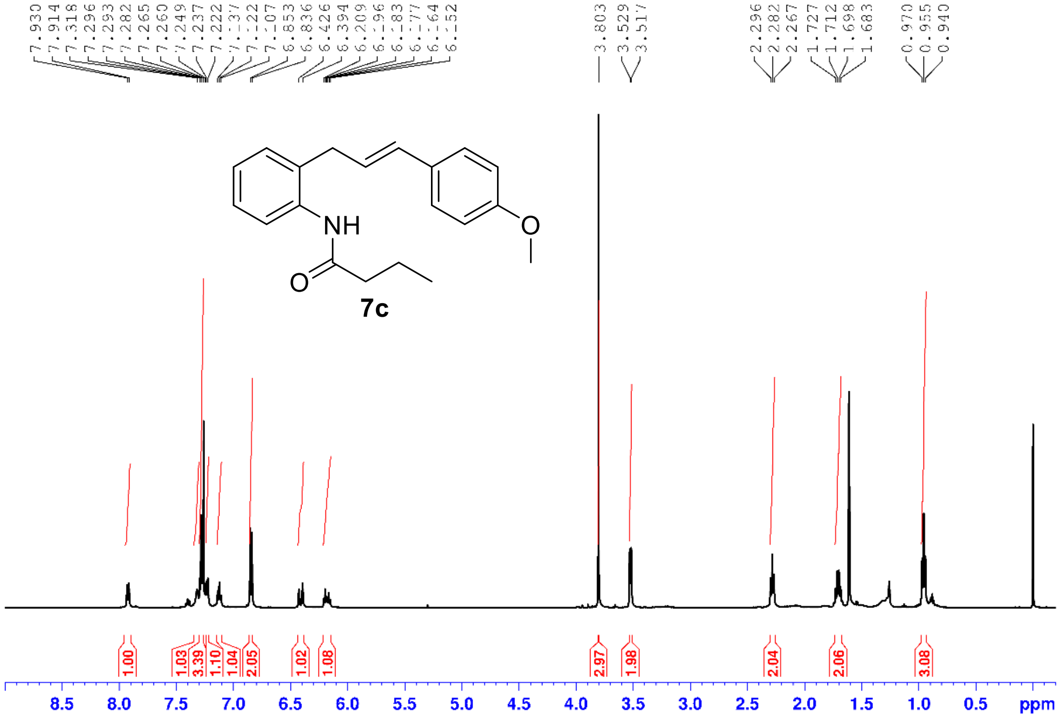

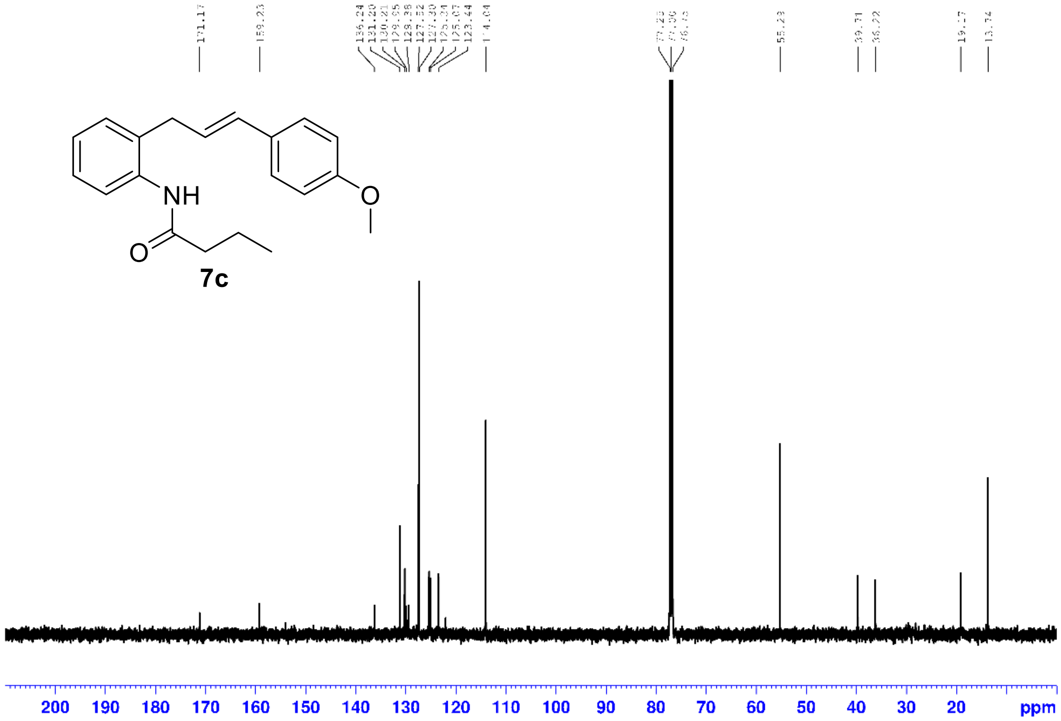

Figure S15: 500 MHz ^{1}H- and 126 MHz ^{13}C-NMR spectra of compound **7c** in $CDCl_3$.

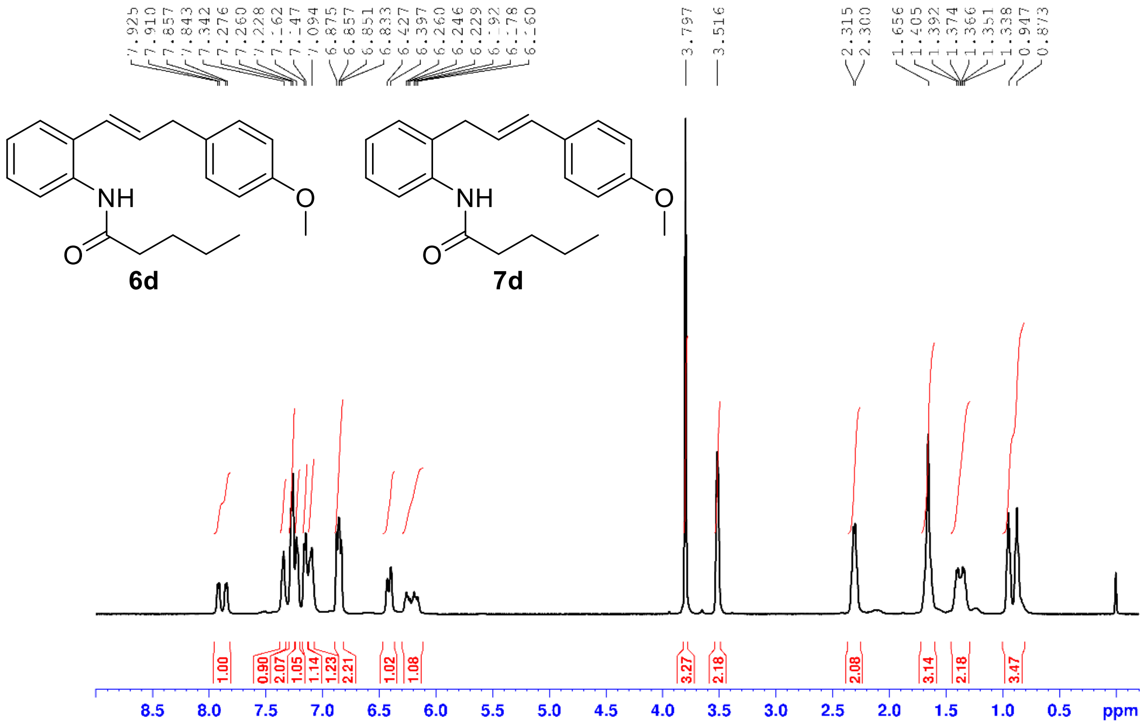

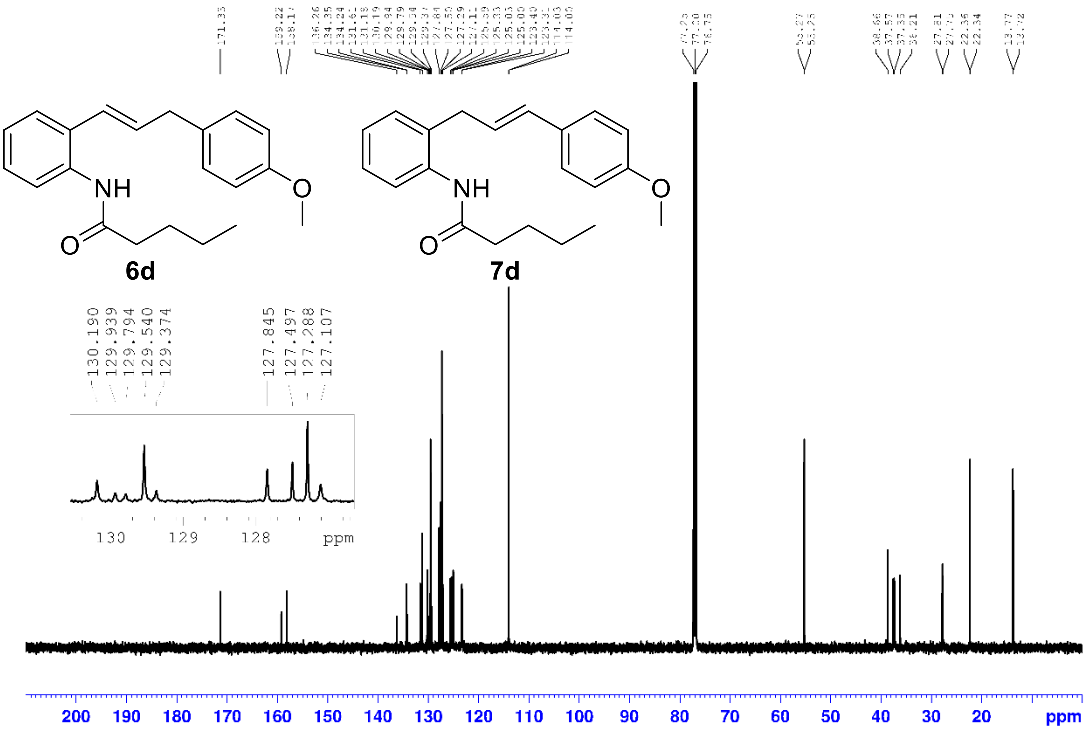

Figure S16: 500 MHz ^{1}H- and 126 MHz ^{13}C-NMR spectra of compound **6d,7d** in $CDCl_3$.

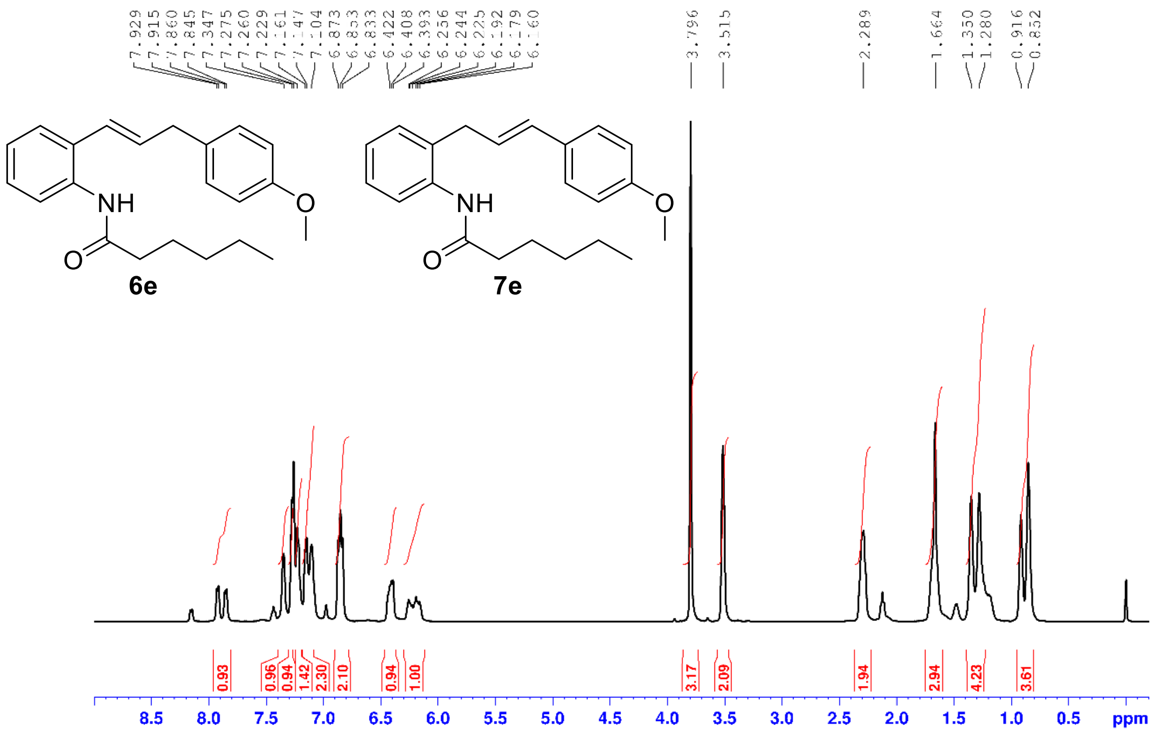

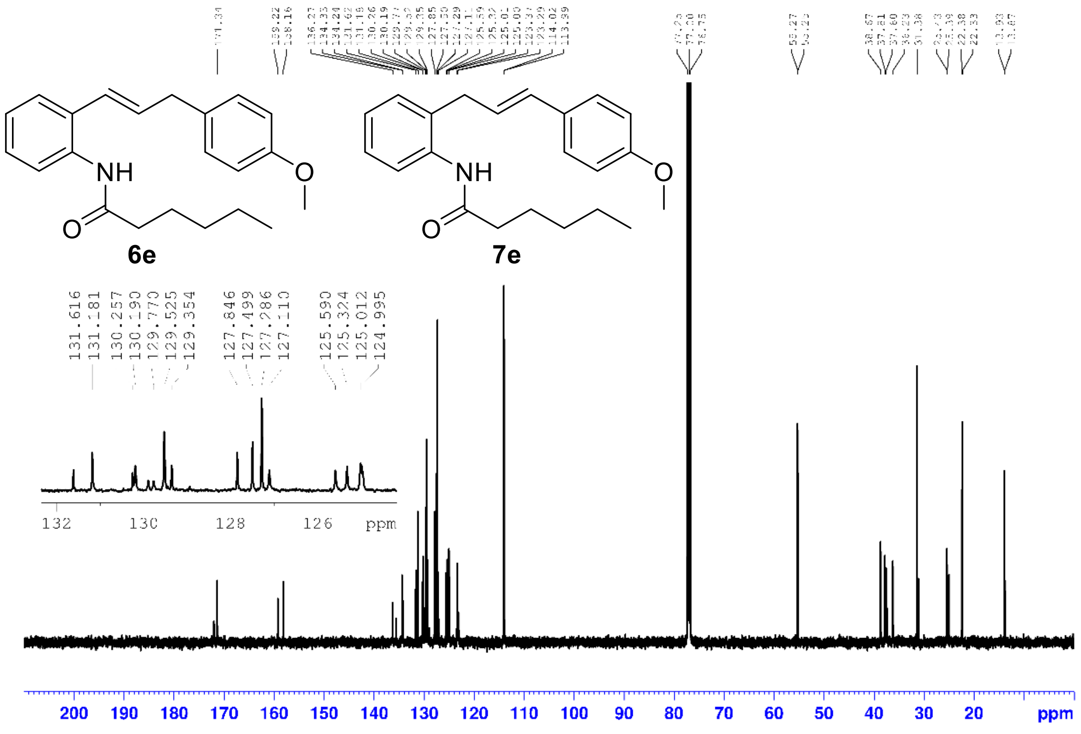

Figure S17: 500 MHz ^{1}H- and 126 MHz ^{13}C-NMR spectra of compound **6e,7e** in $CDCl_3$.

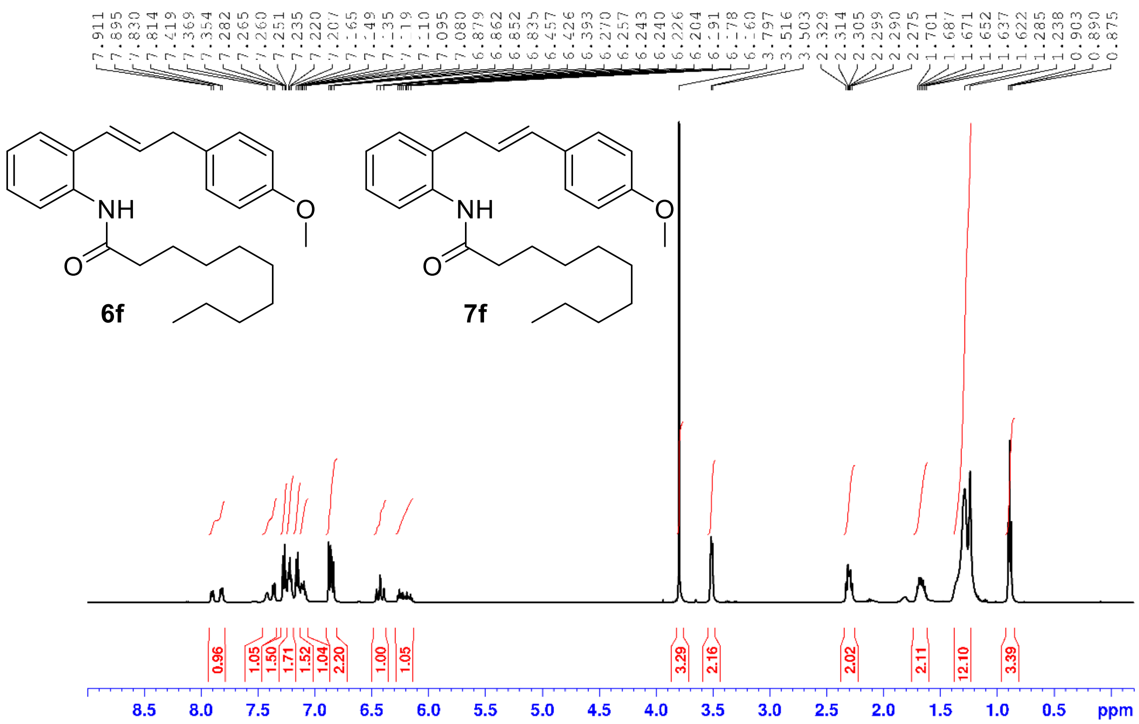

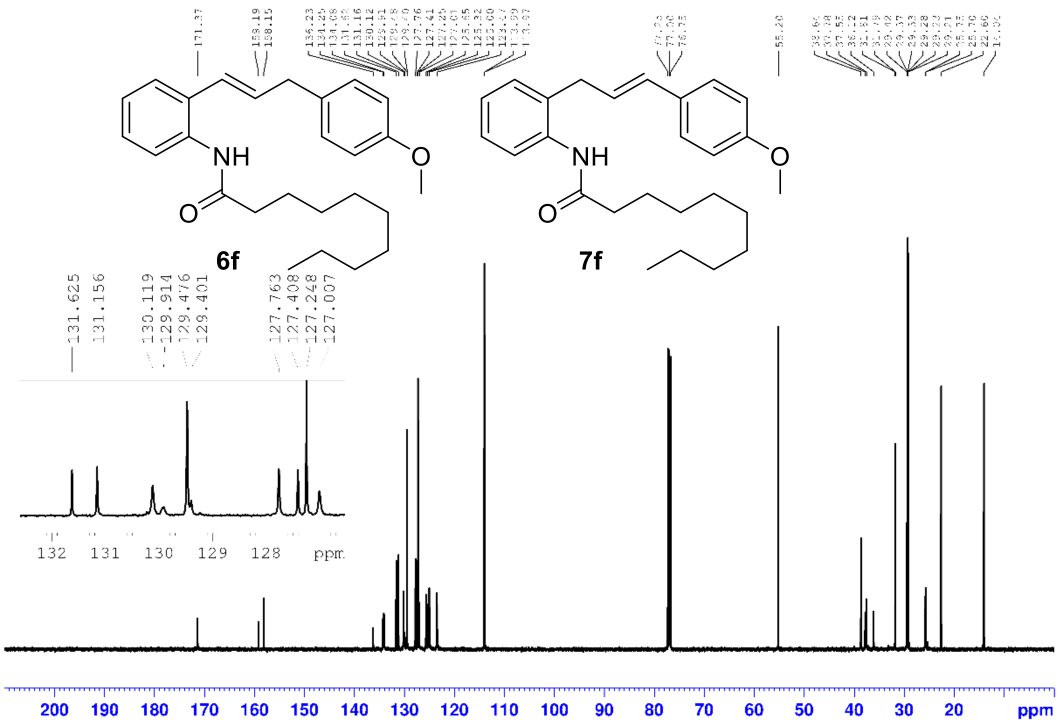

Figure S18: 500 MHz ^{1}H- and 126 MHz ^{13}C-NMR spectra of compound **6f,7f** in $CDCl_3$.

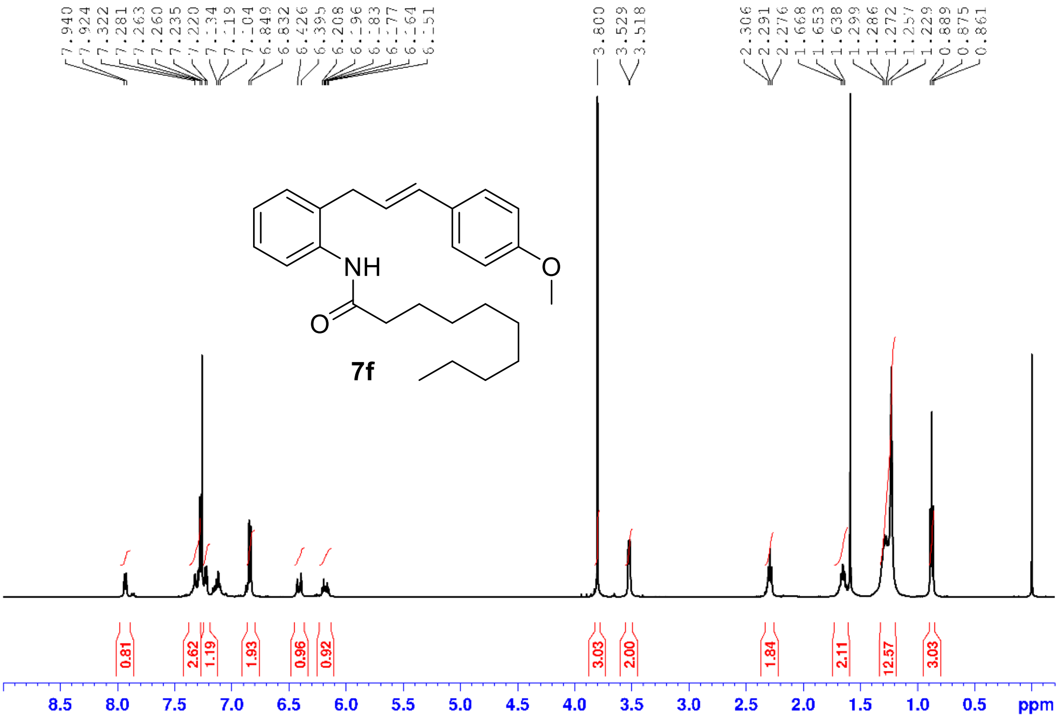

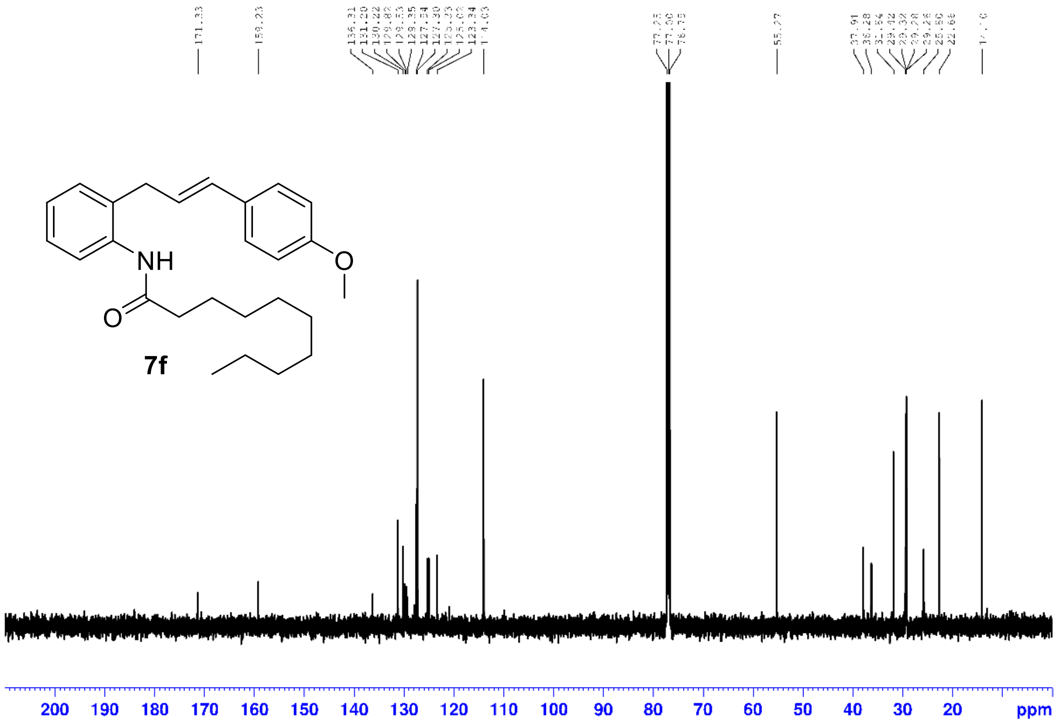

Figure S19: 500 MHz ^{1}H- and 126 MHz ^{13}C-NMR spectra of compound **7f** in $CDCl_3$.

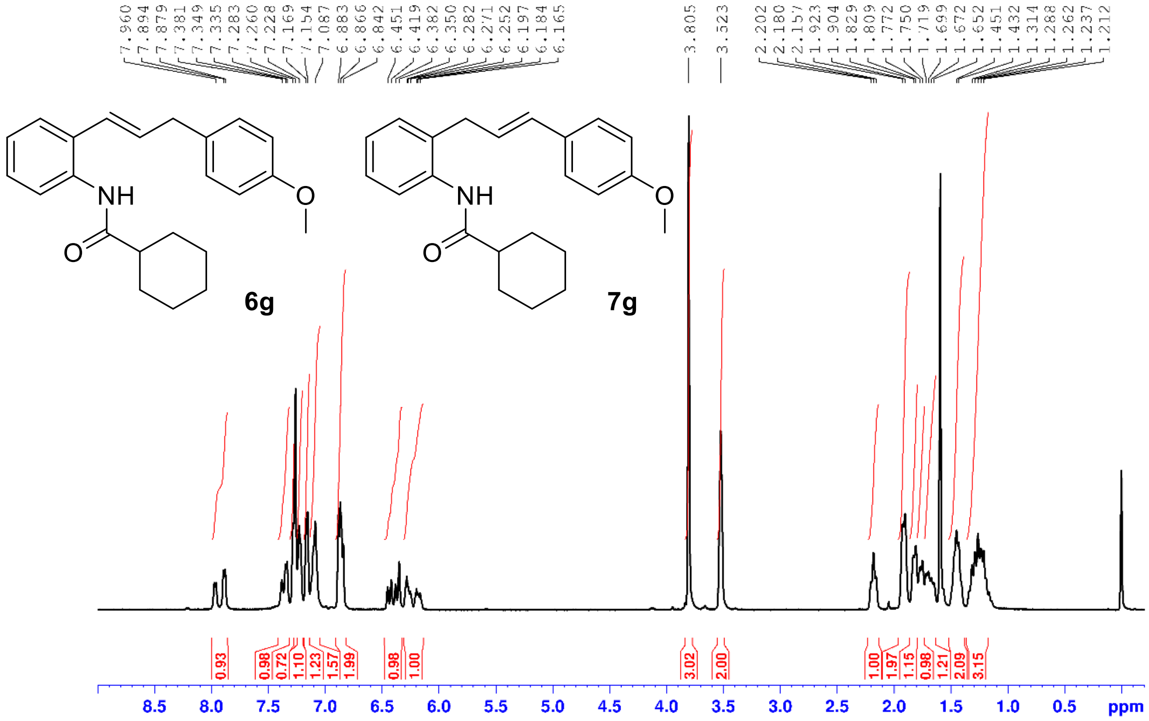

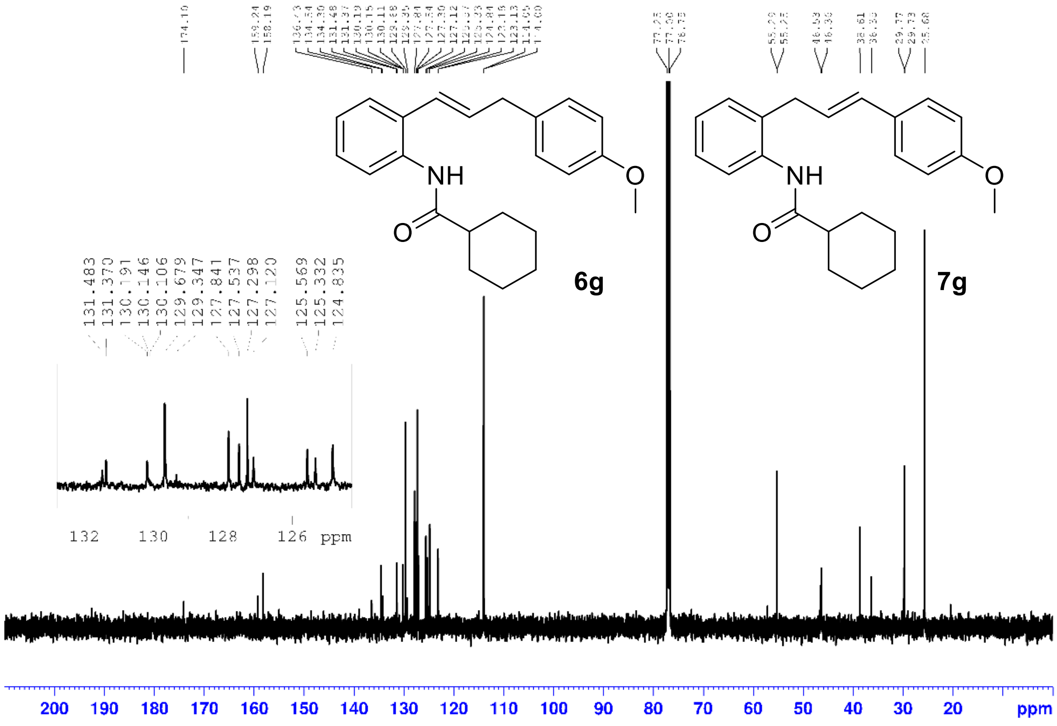

Figure S20: 500 MHz ^{1}H- and 126 MHz ^{13}C-NMR spectra of compound **6g,7g** in $CDCl_3$.

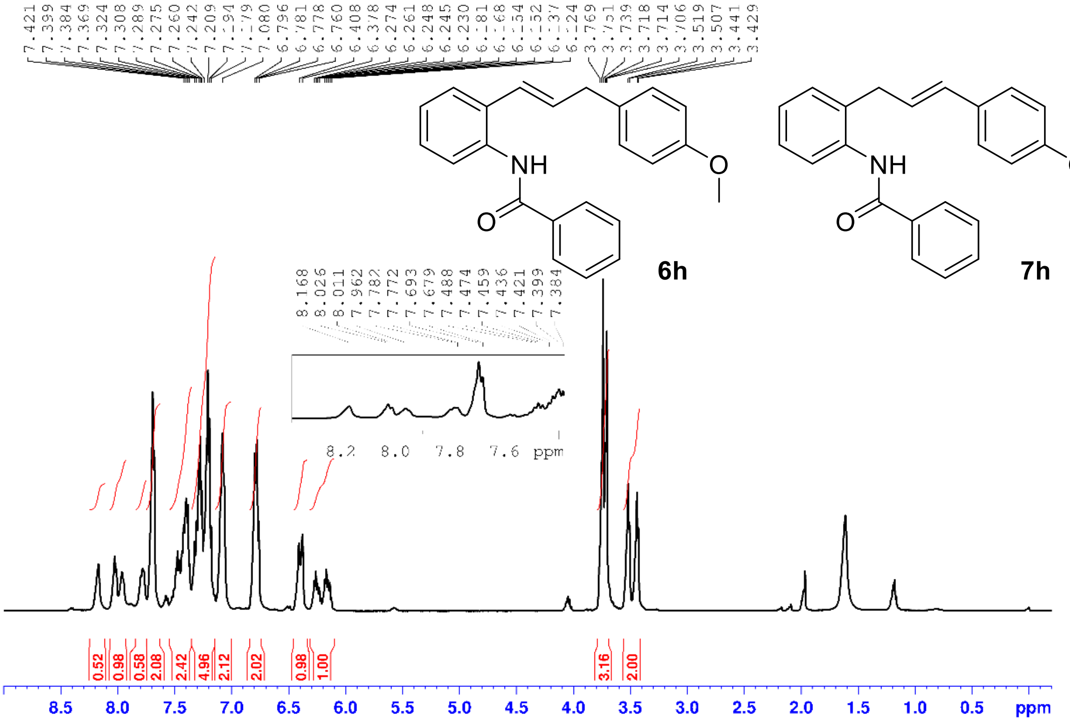

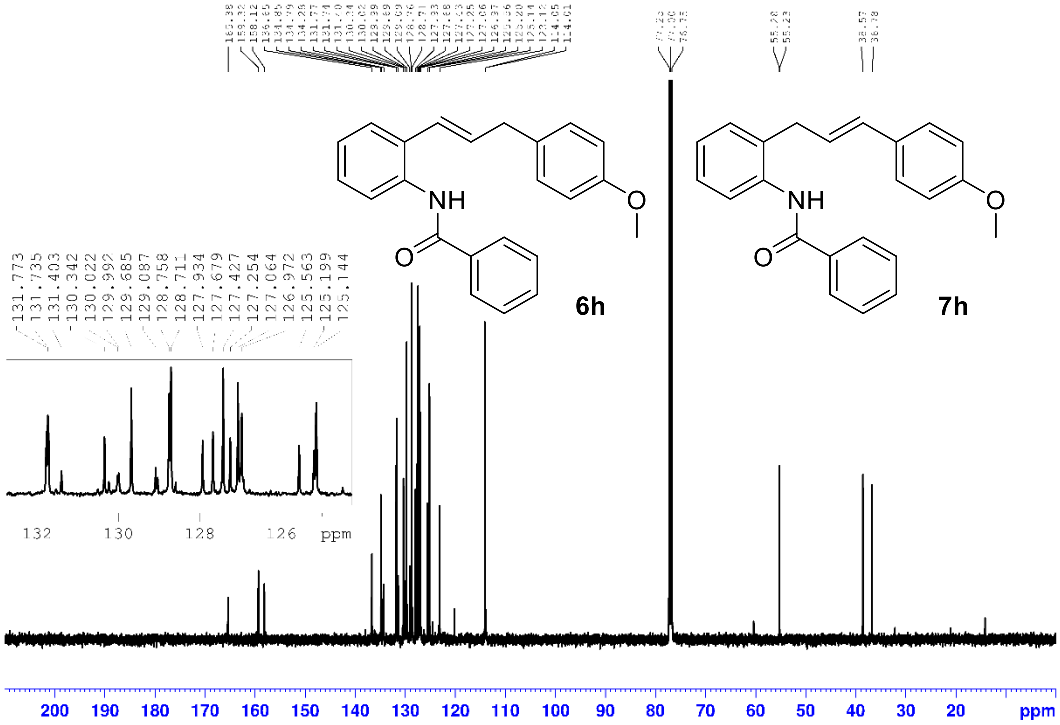

Figure S21: 500 MHz ^{1}H- and 126 MHz ^{13}C-NMR spectra of compound **6h,7h** in $CDCl_3$.

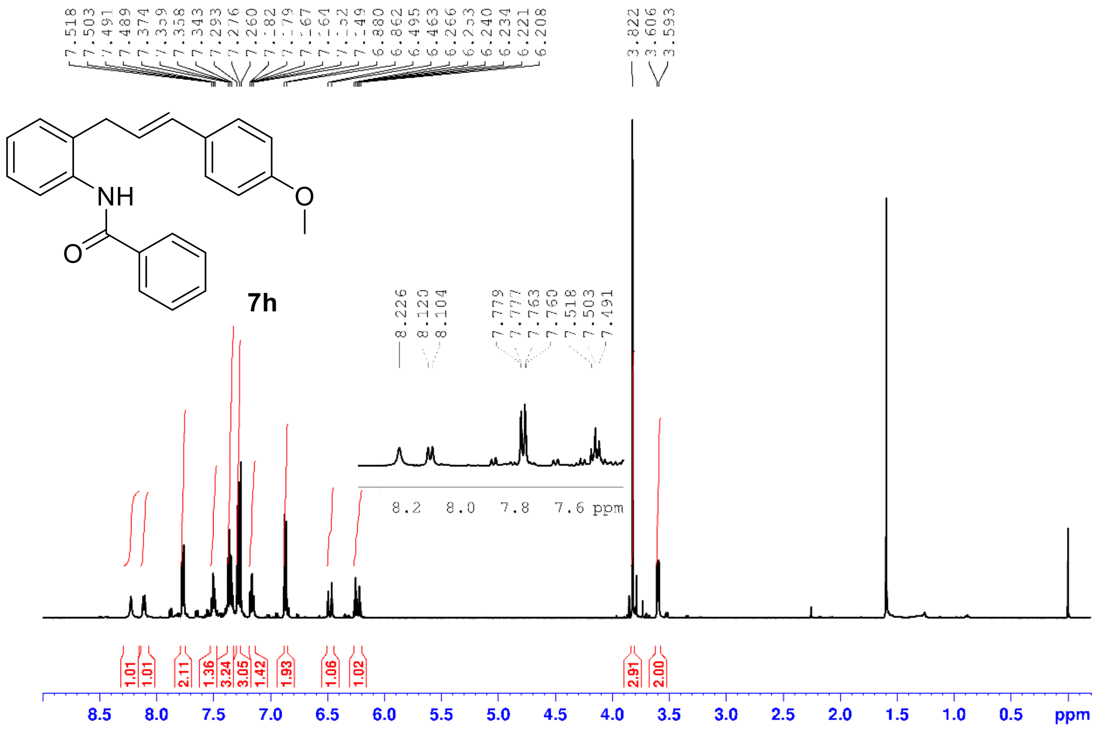

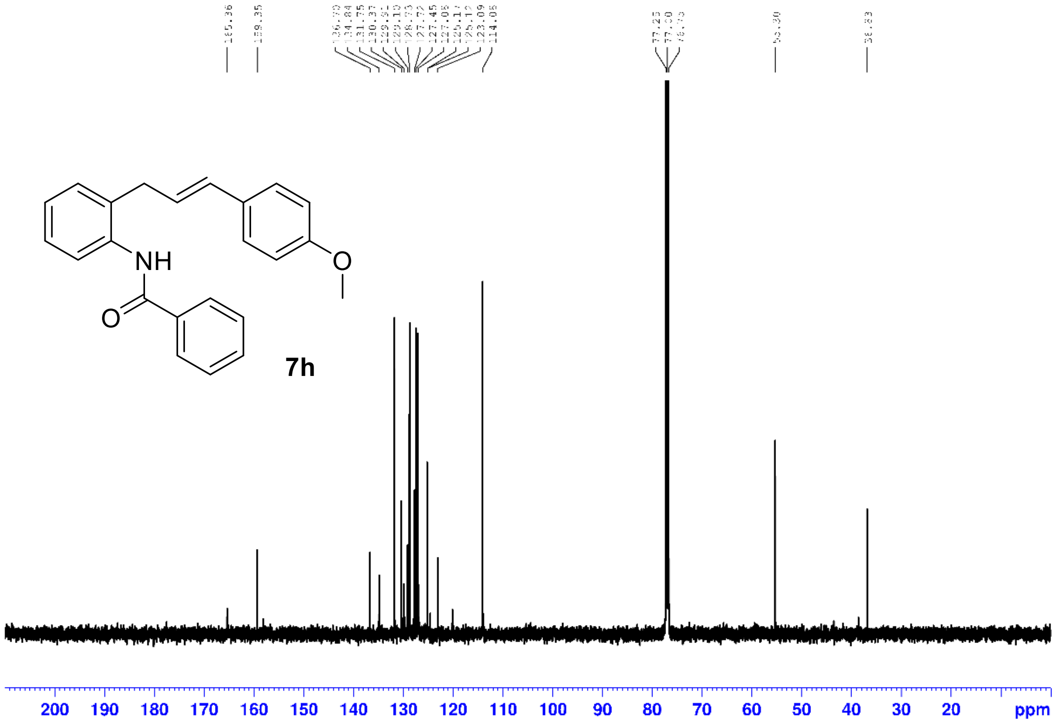

Figure S22: 500 MHz ^{1}H- and 126 MHz ^{13}C-NMR spectra of compound **7h** in $CDCl_3$.

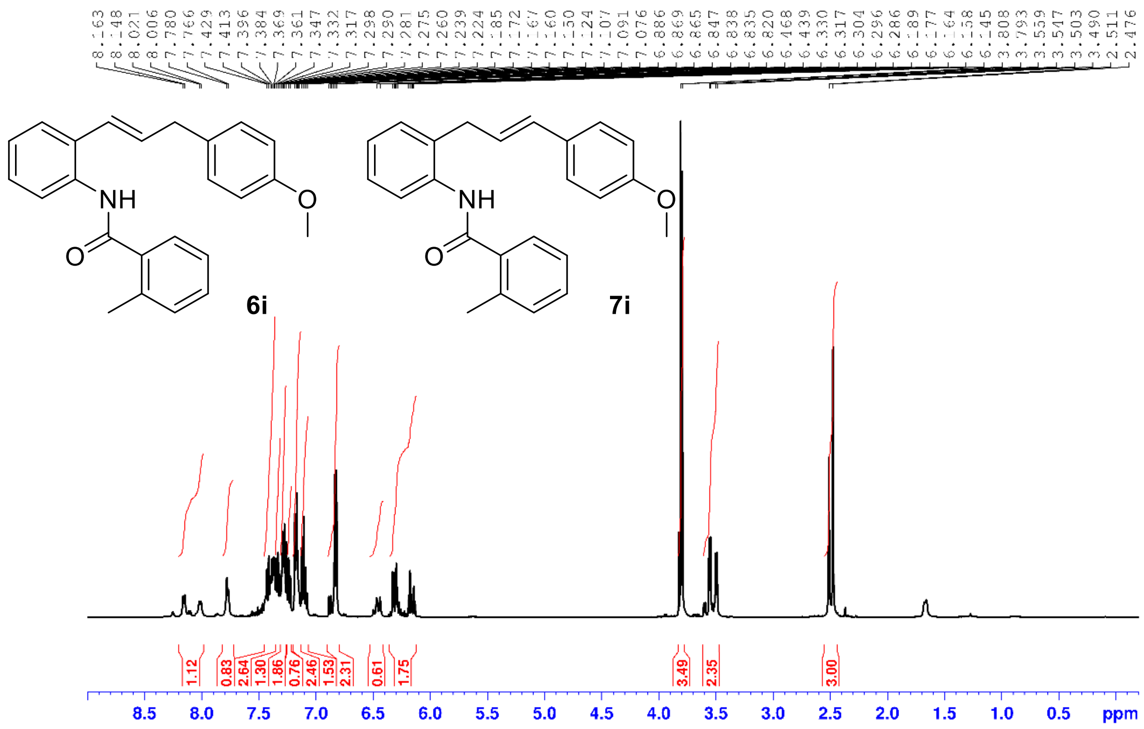

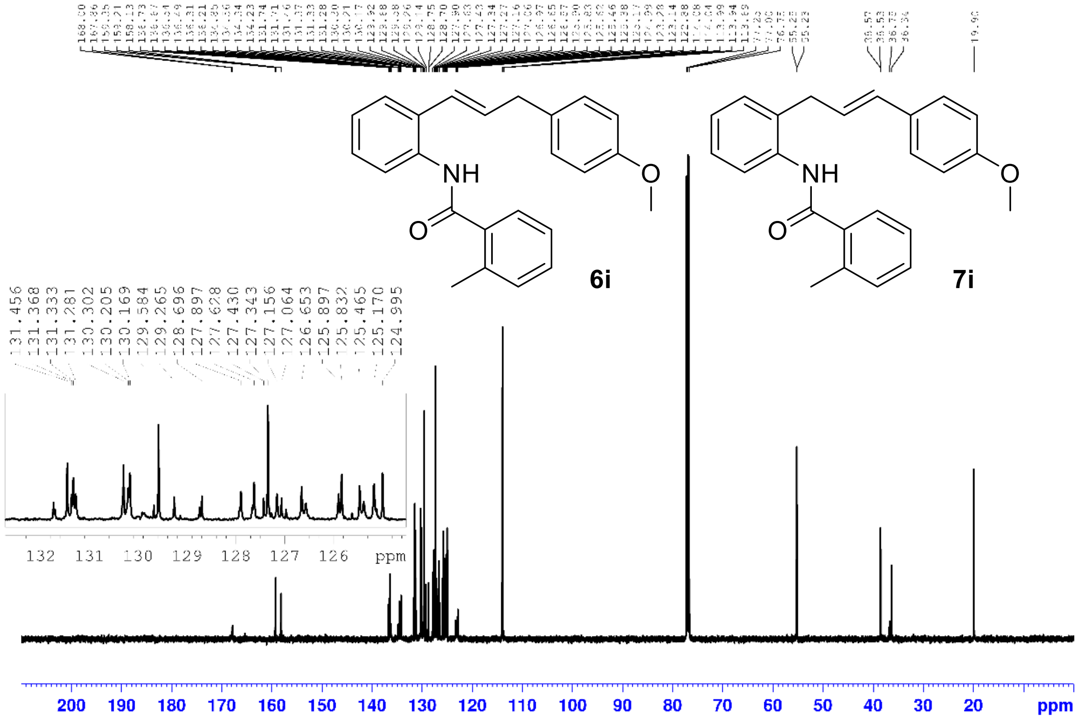

Figure S23: 500 MHz ^{1}H- and 126 MHz ^{13}C-NMR spectra of compound **6i,7i** in $CDCl_3$.

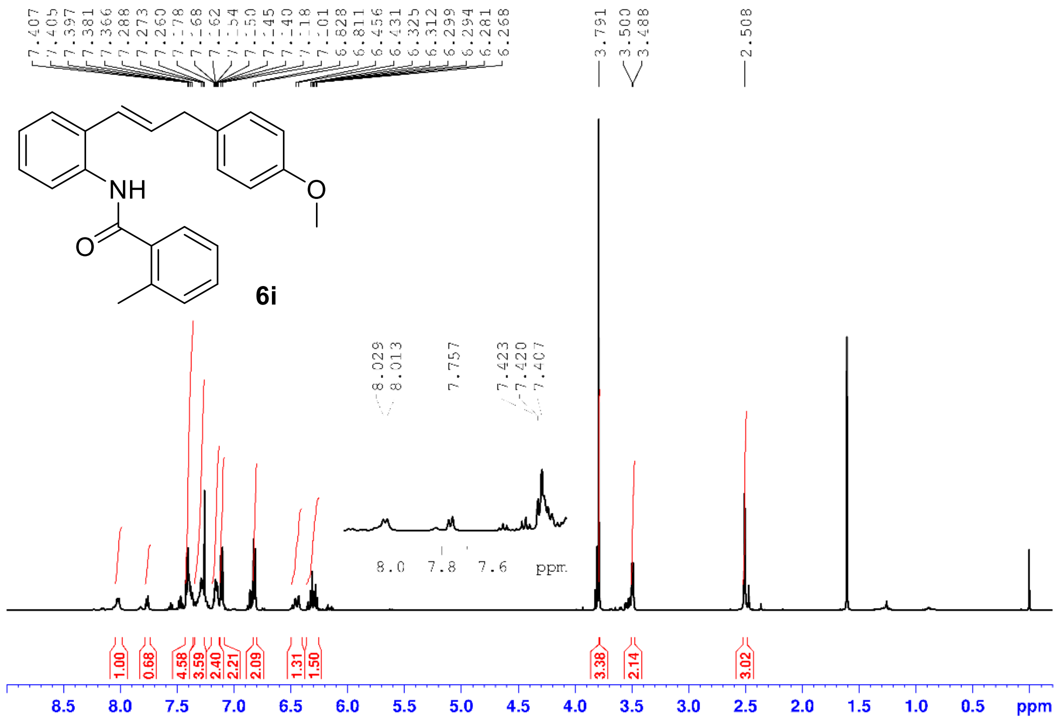

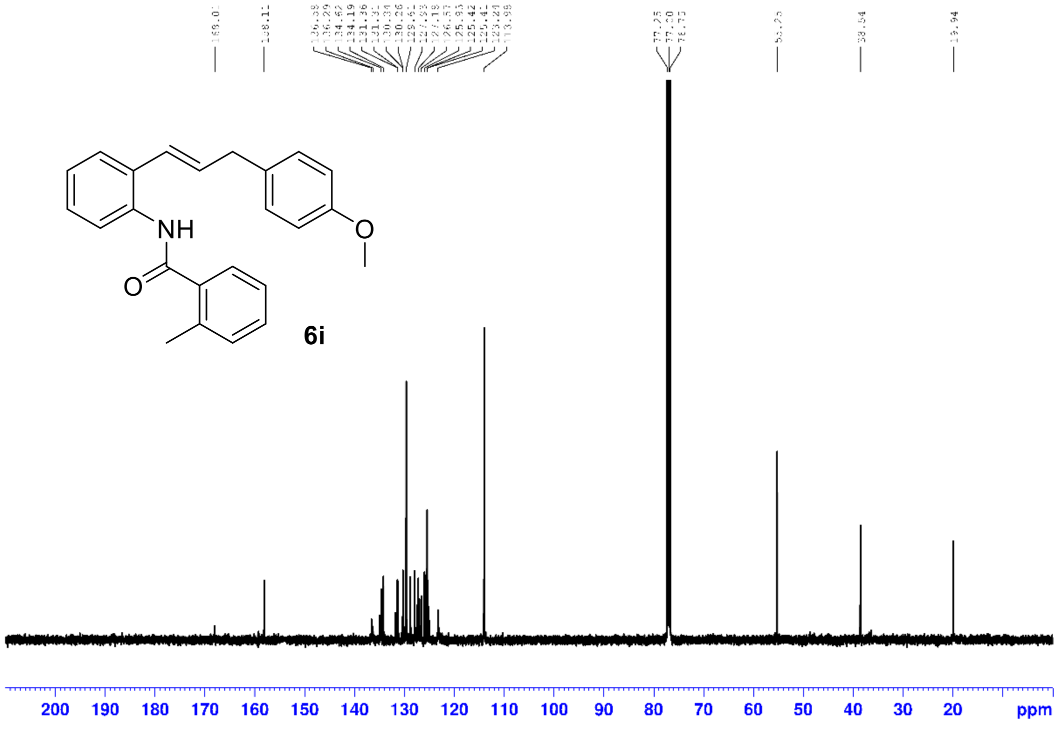

Figure S24: 500 MHz ^{1}H- and 126 MHz ^{13}C-NMR spectra of compound **6i** in $CDCl_3$.

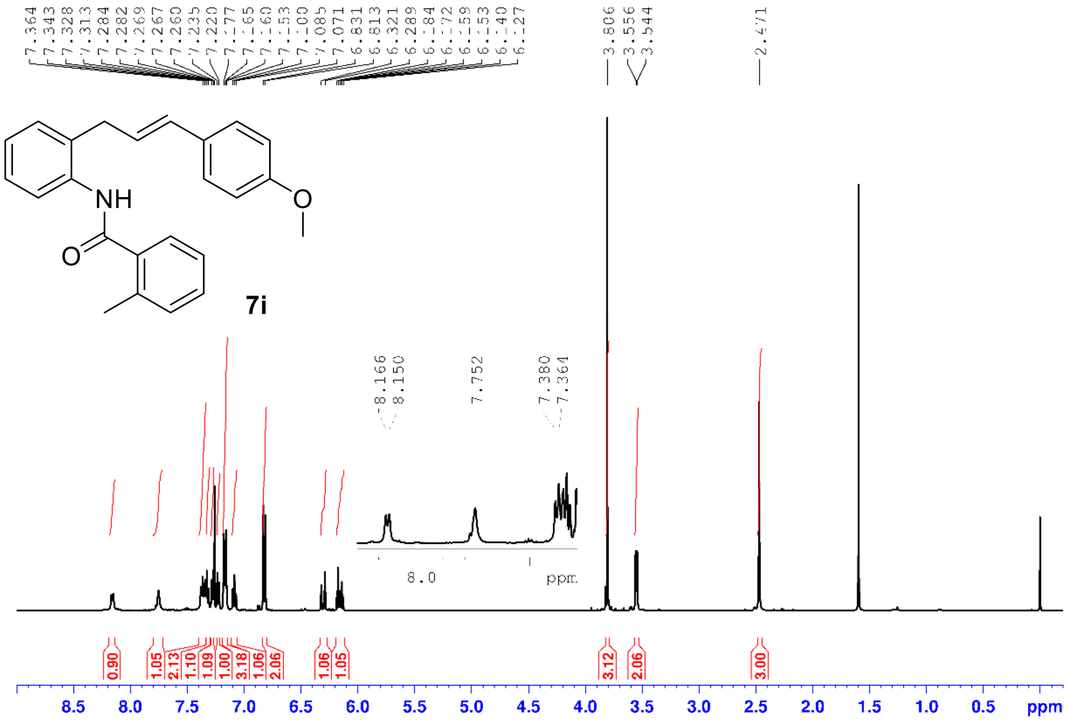

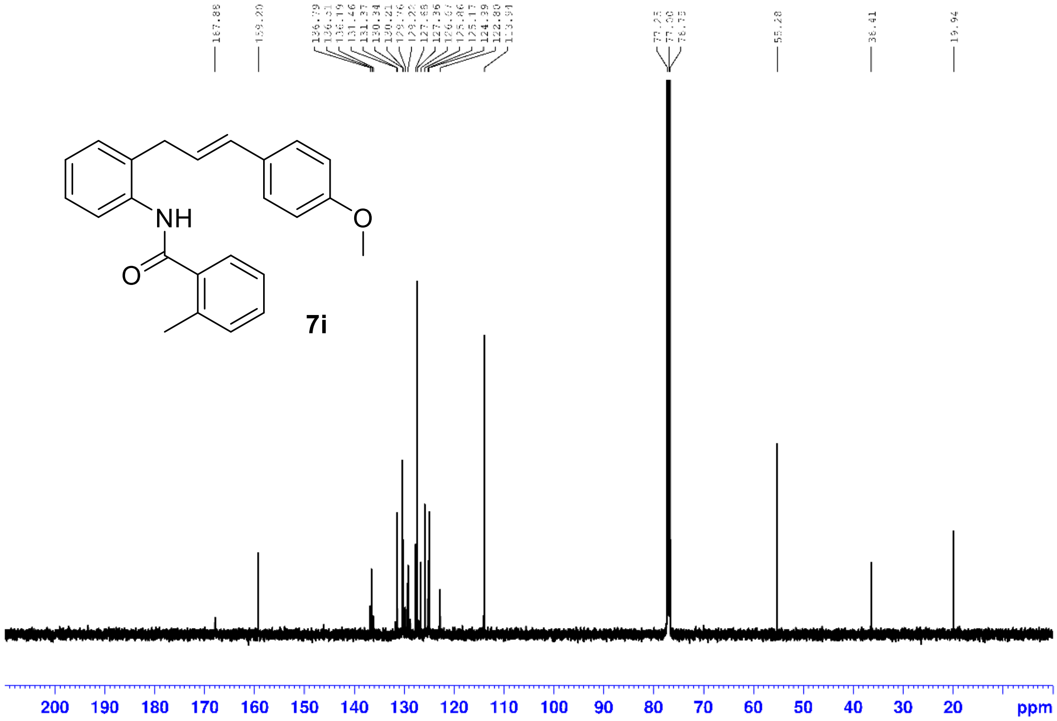

Figure S25: 500 MHz ^{1}H- and 126 MHz ^{13}C-NMR spectra of compound **7i** in $CDCl_3$.

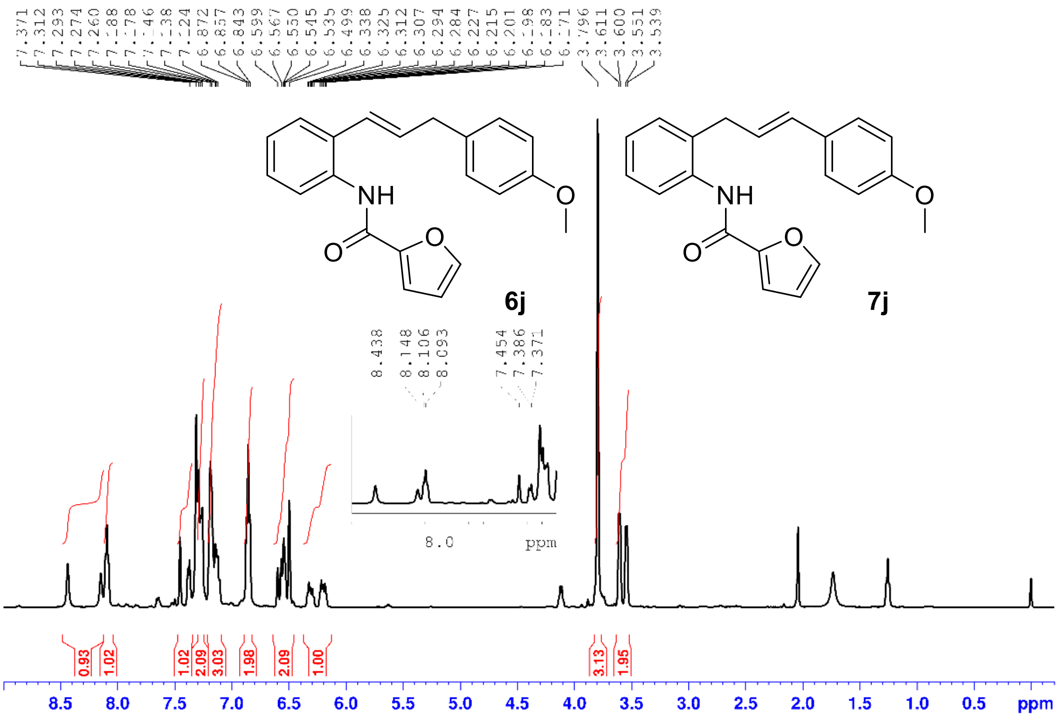

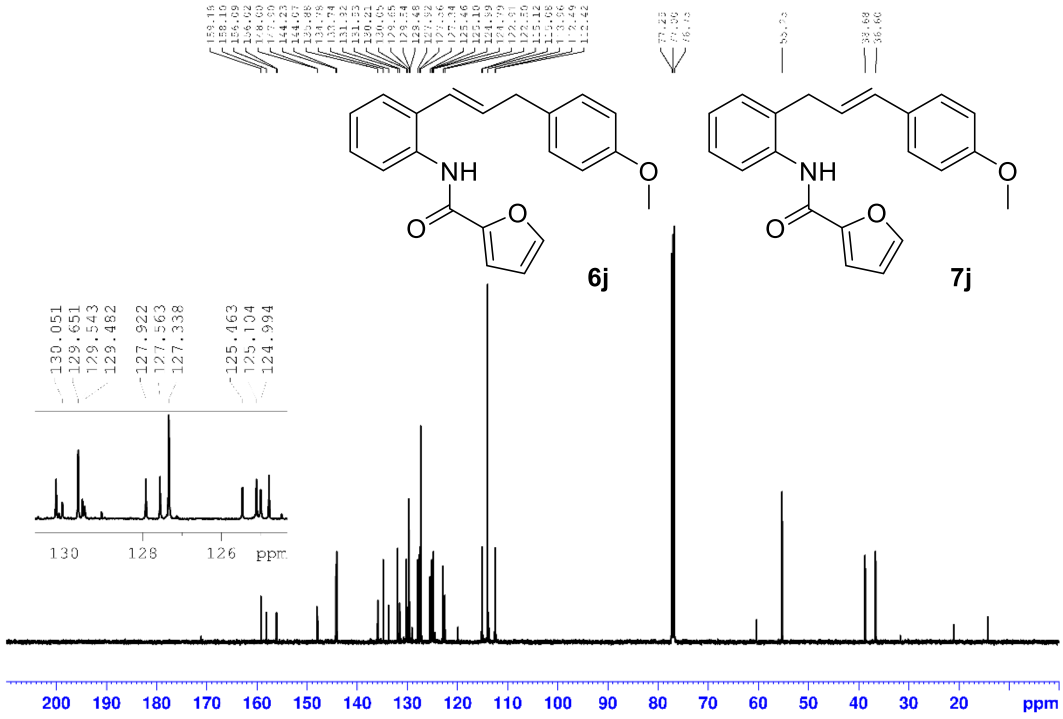

Figure S26: 500 MHz ^{1}H- and 126 MHz ^{13}C-NMR spectra of compound **6j,7j** in $CDCl_3$.

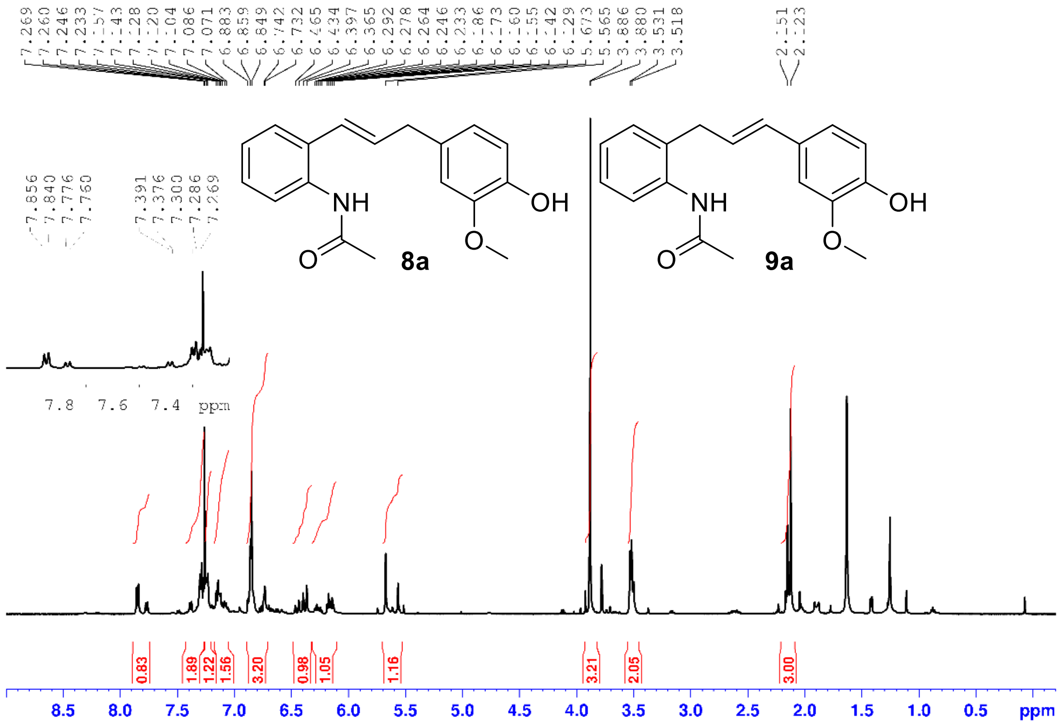

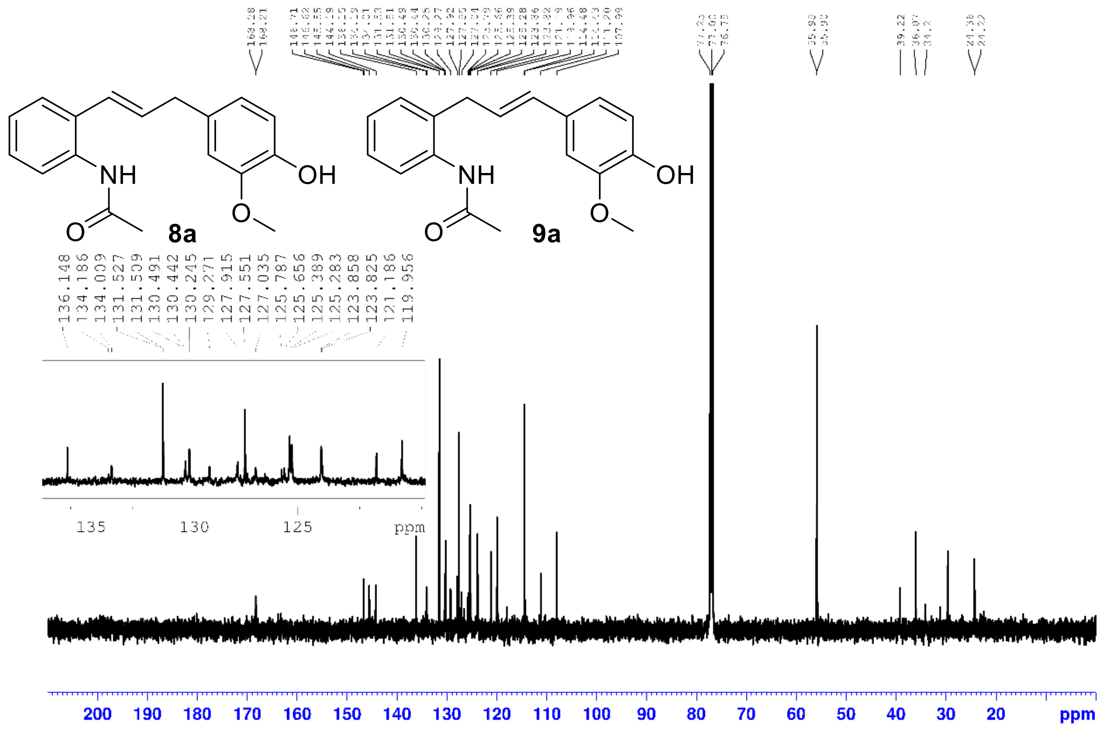

Figure S27: 500 MHz ^{1}H- and 126 MHz ^{13}C-NMR spectra of compound **8a,9a** in $CDCl_3$.

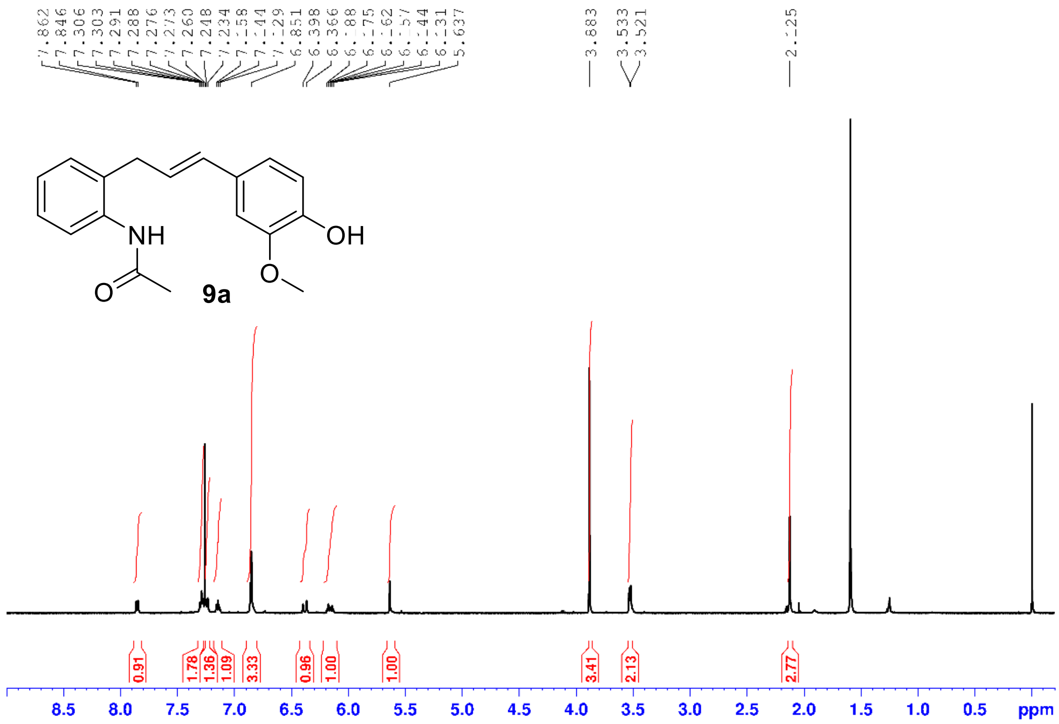

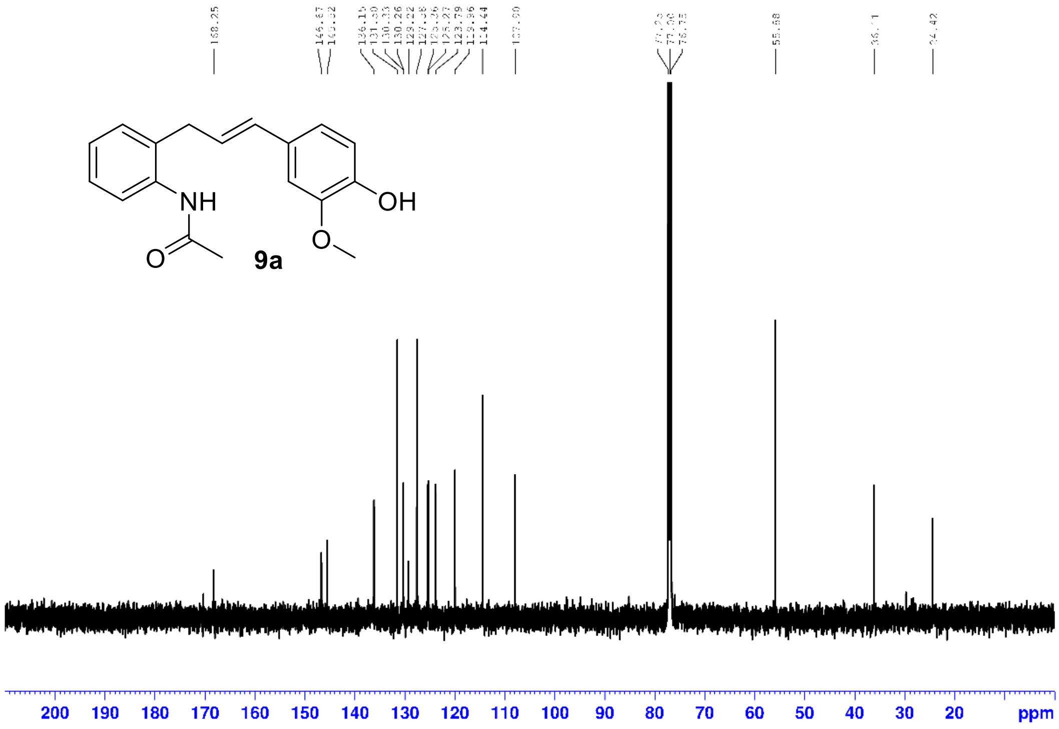

Figure S28: 500 MHz ^{1}H- and 126 MHz ^{13}C-NMR spectra of compound **9a** in $CDCl_3$.

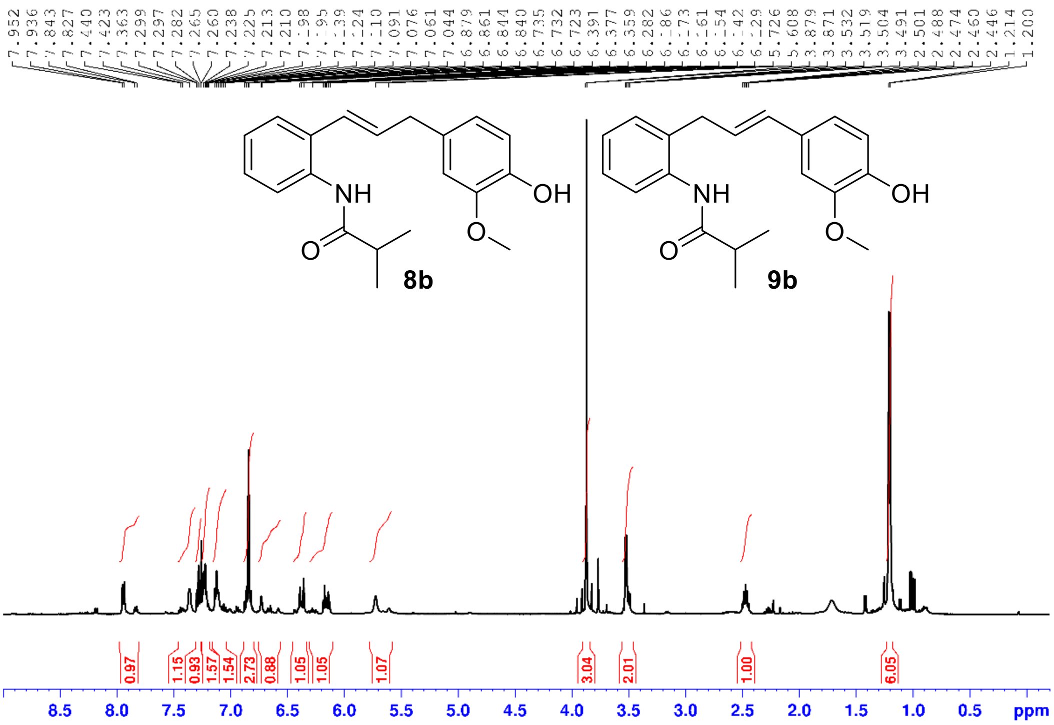

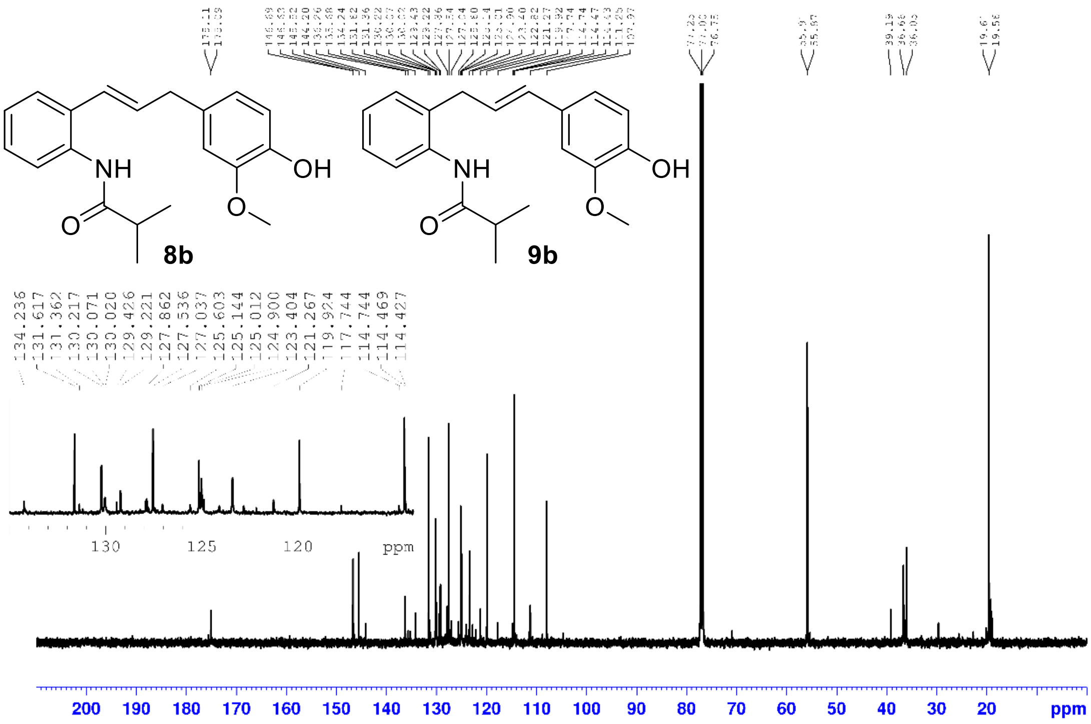

Figure S29: 500 MHz ^{1}H- and 126 MHz ^{13}C-NMR spectra of compound **8b,9b** in $CDCl_3$.

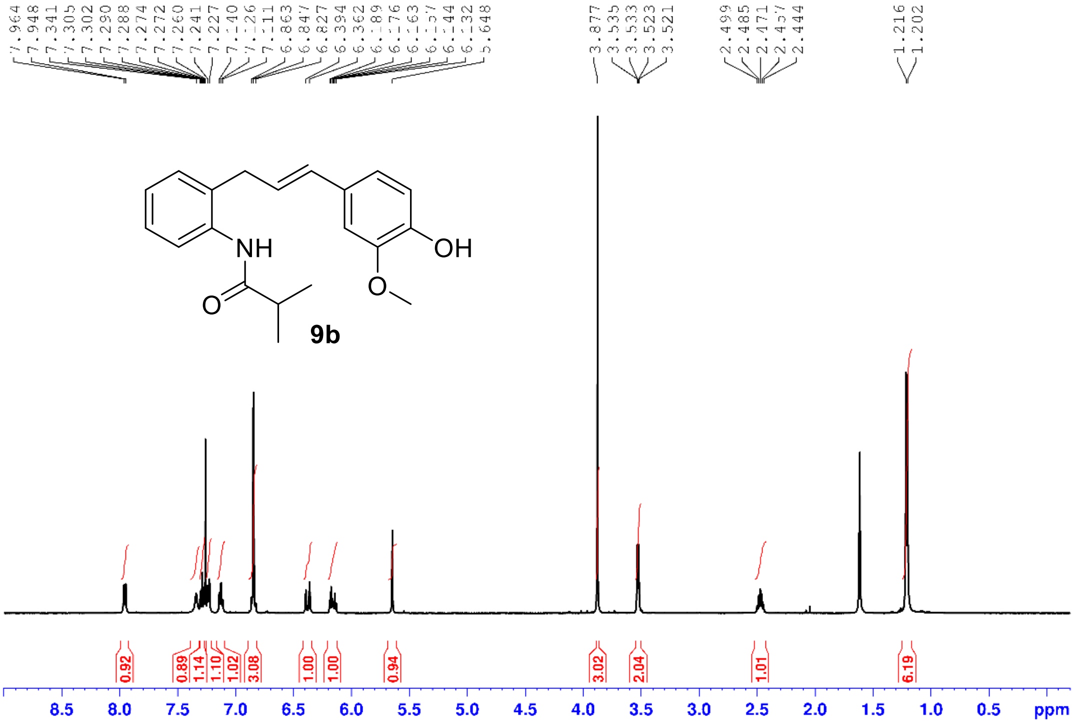

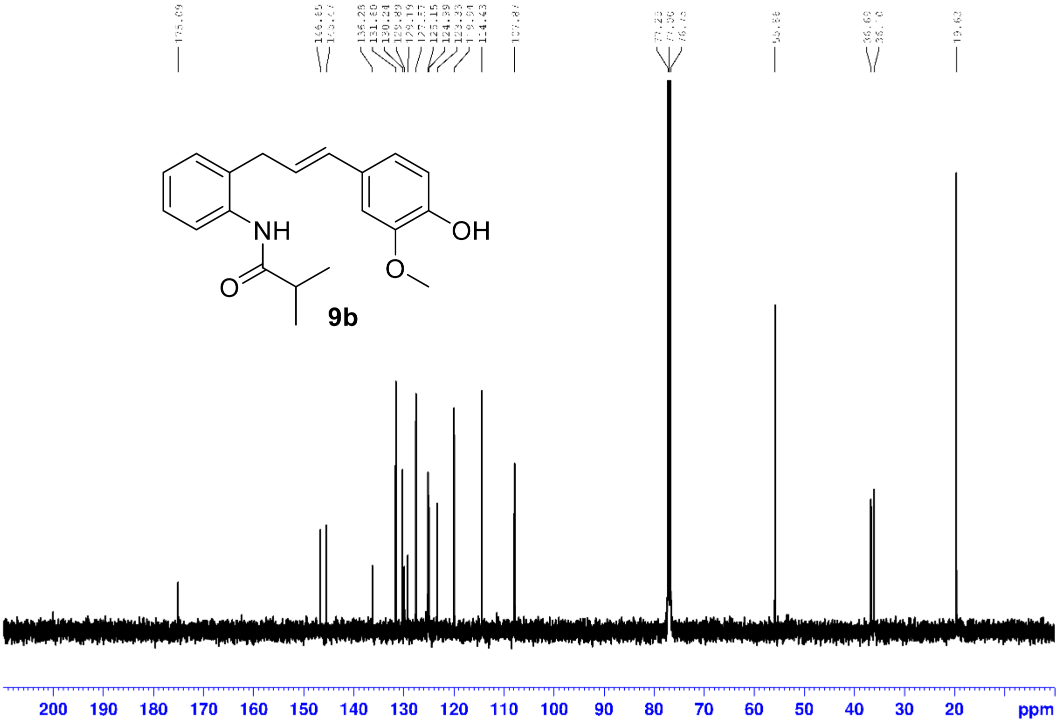

Figure S30: 500 MHz ^{1}H- and 126 MHz ^{13}C-NMR spectra of compound **9b** in $CDCl_3$.

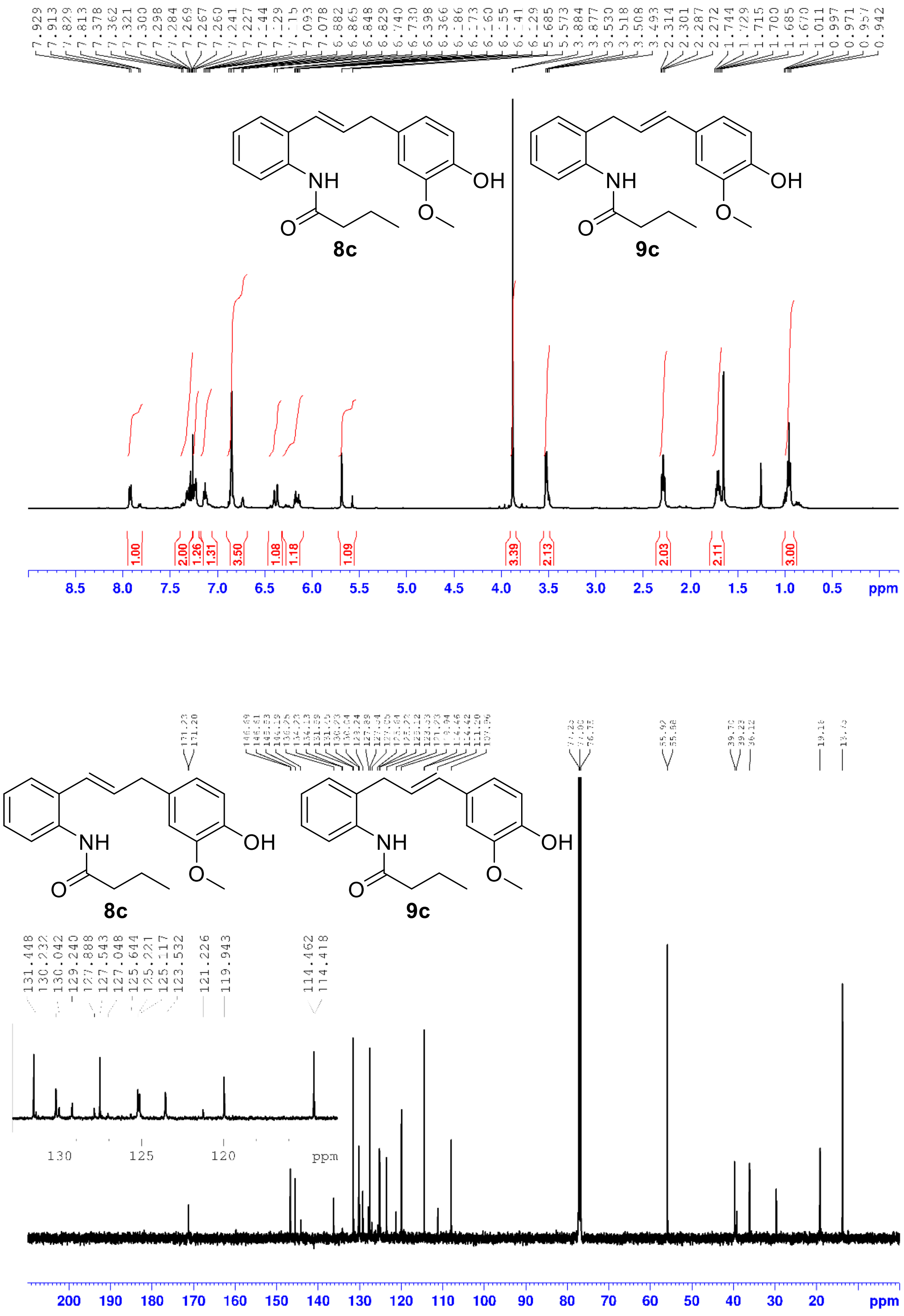

Figure S31: 500 MHz ^{1}H- and 126 MHz ^{13}C-NMR spectra of compound **8c,9c** in $CDCl_3$.

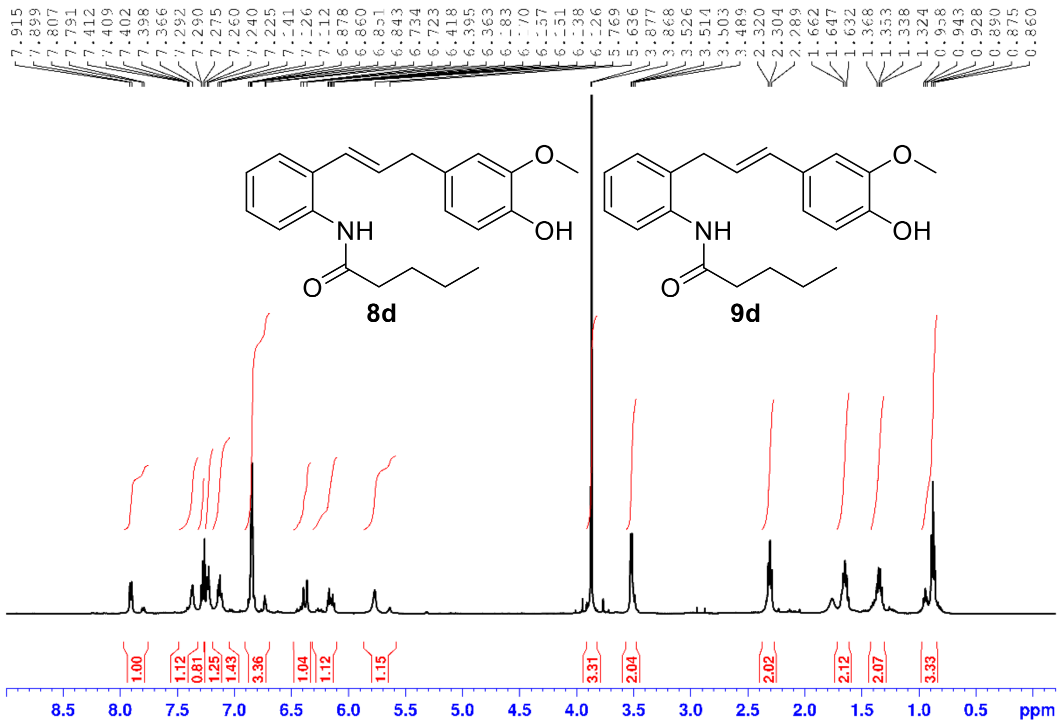

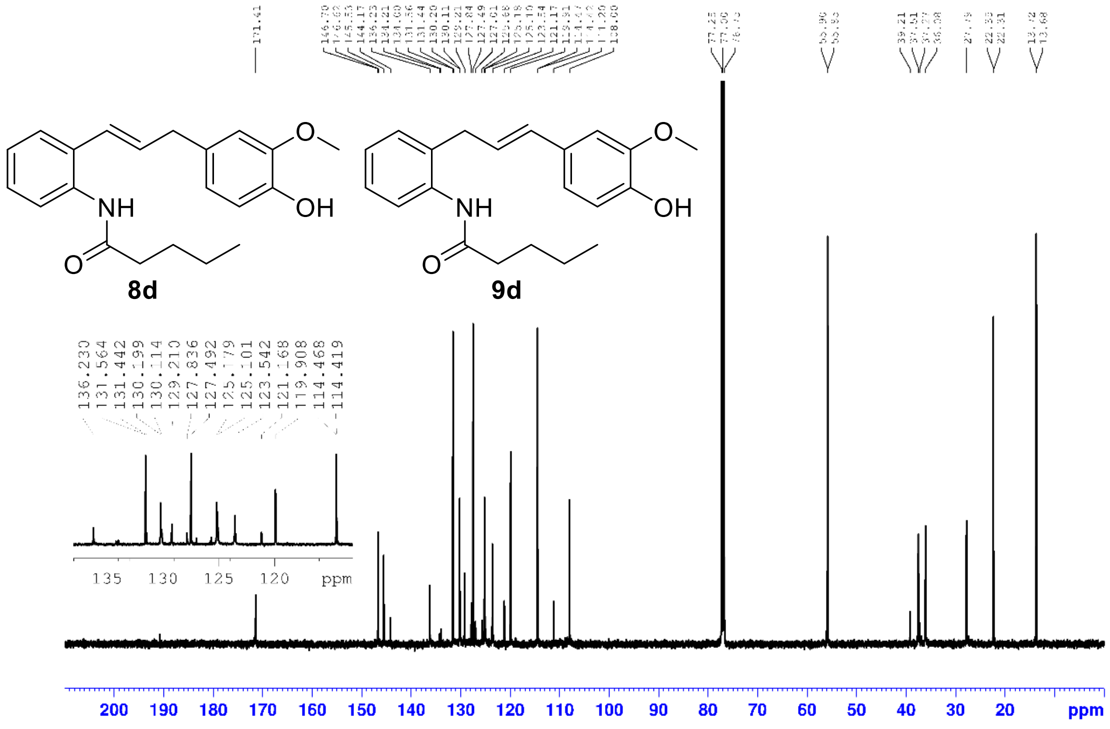

Figure S32: 500 MHz ^{1}H- and 126 MHz ^{13}C-NMR spectra of compound **8d,9d** in $CDCl_3$.

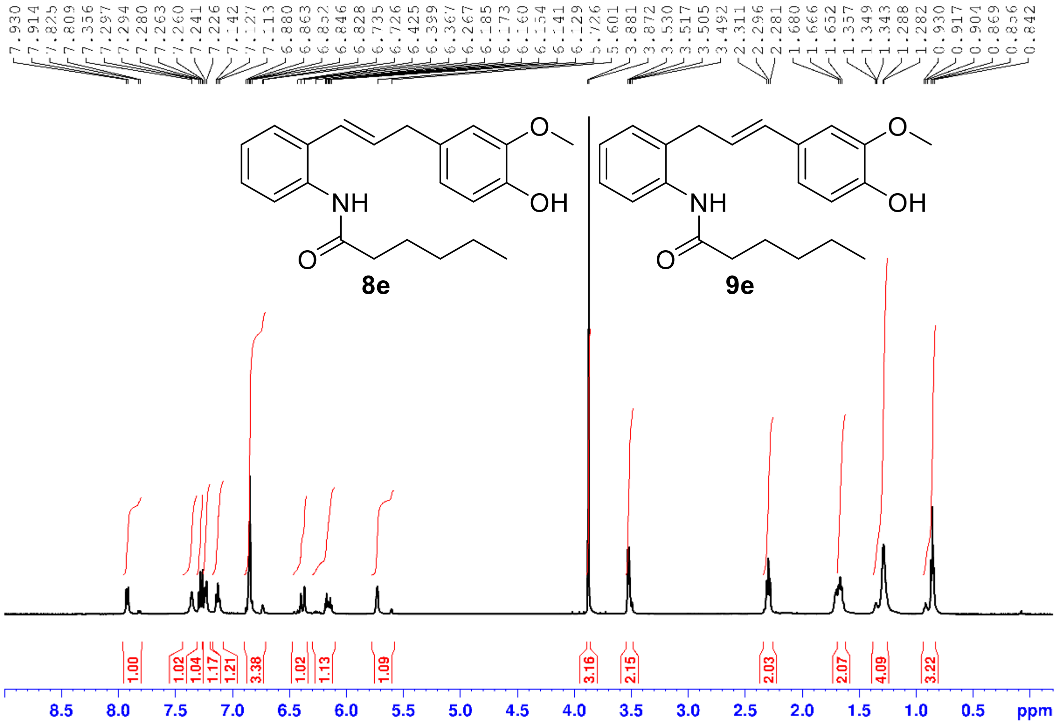

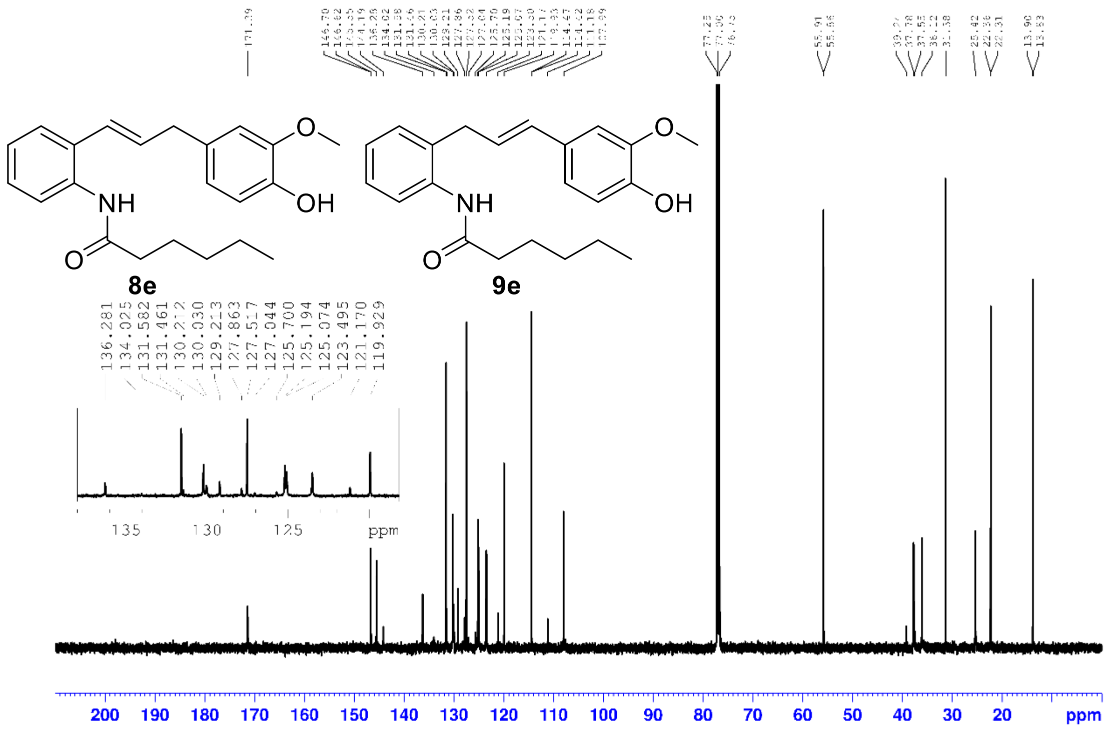

Figure S33: 500 MHz ^{1}H- and 126 MHz ^{13}C-NMR spectra of compound **8e,9e** in $CDCl_3$.

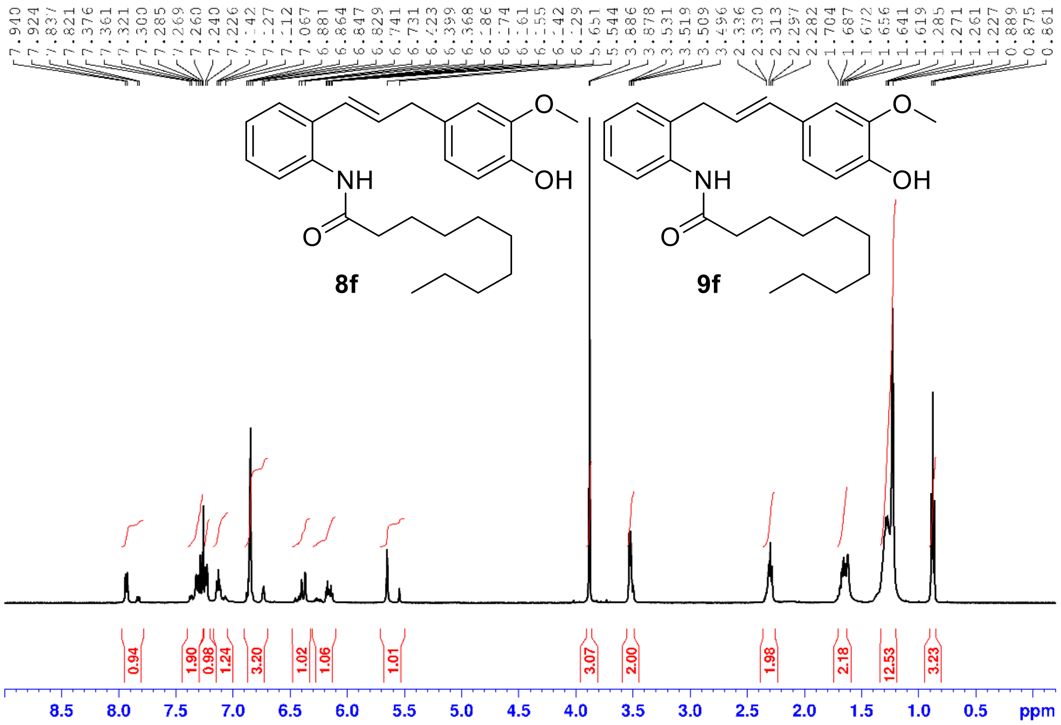

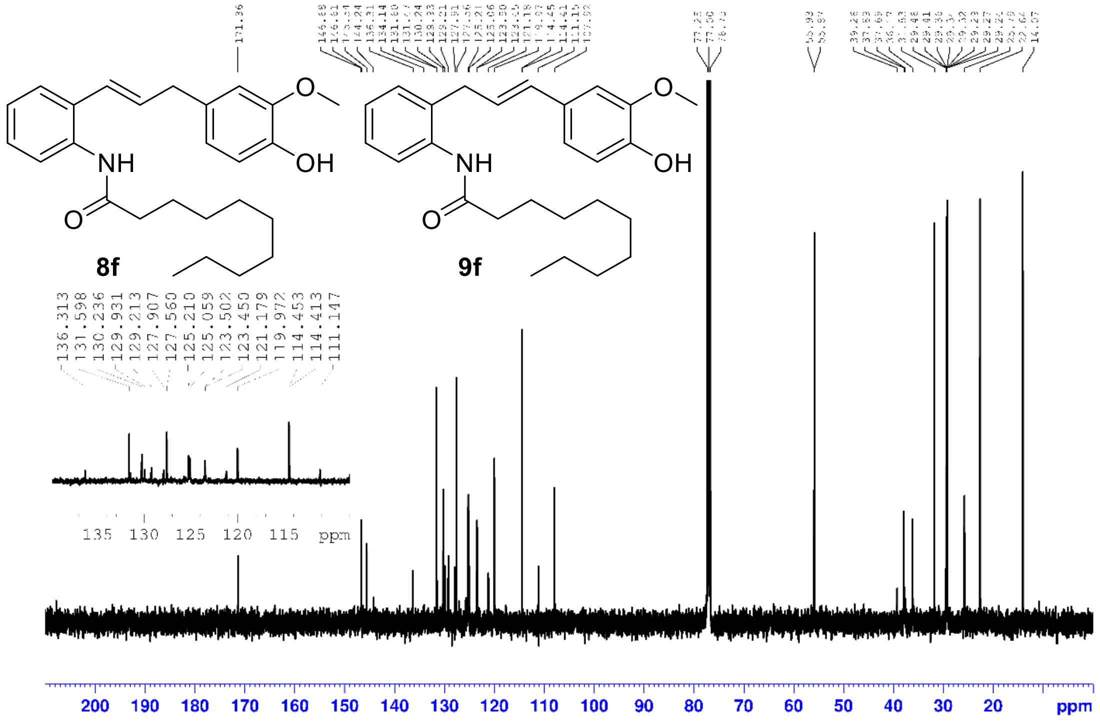

Figure S34: 500 MHz ^{1}H- and 126 MHz ^{13}C-NMR spectra of compound **8f,9f** in $CDCl_3$.

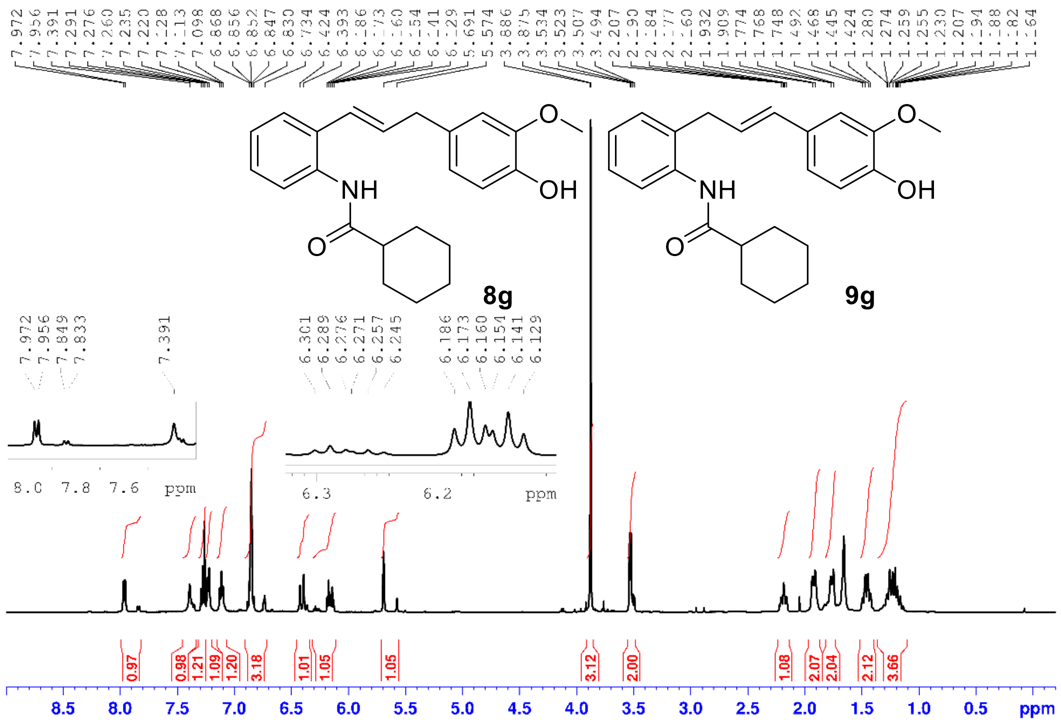

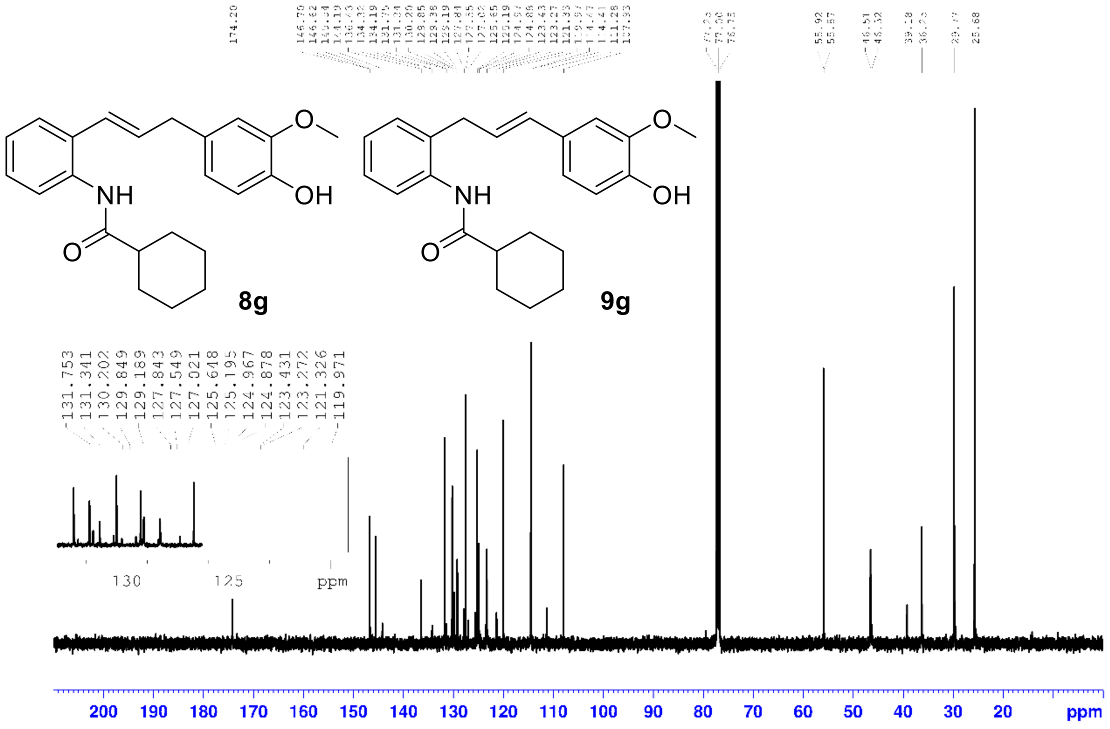

Figure S35: 500 MHz ^{1}H- and 126 MHz ^{13}C-NMR spectra of compound **8g,9g** in $CDCl_3$.

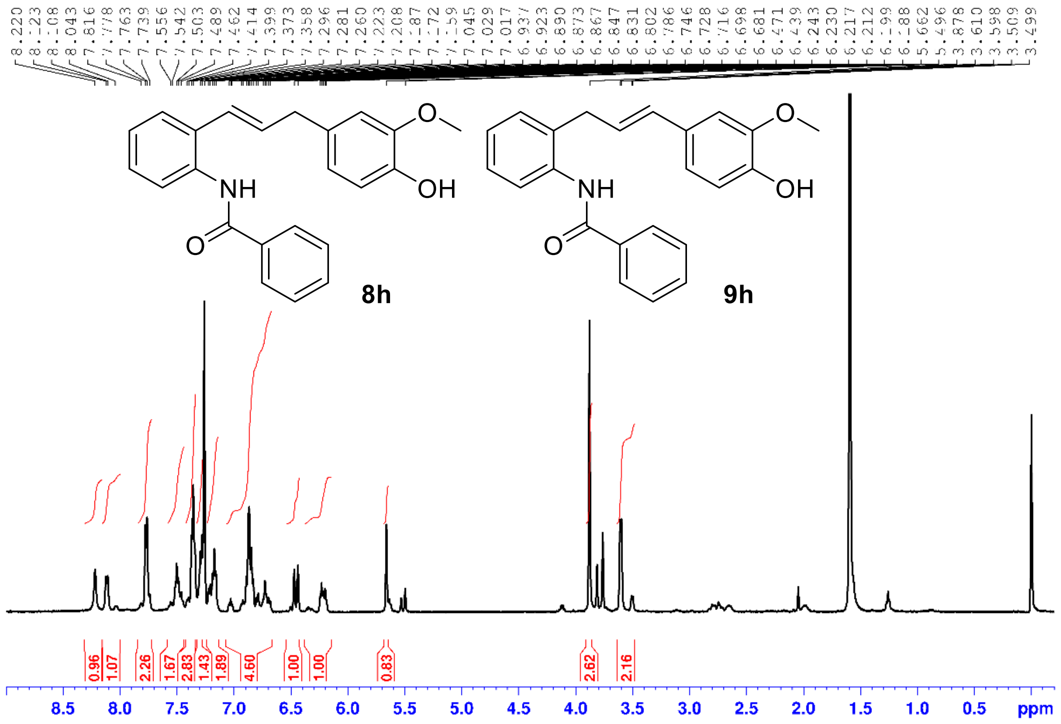

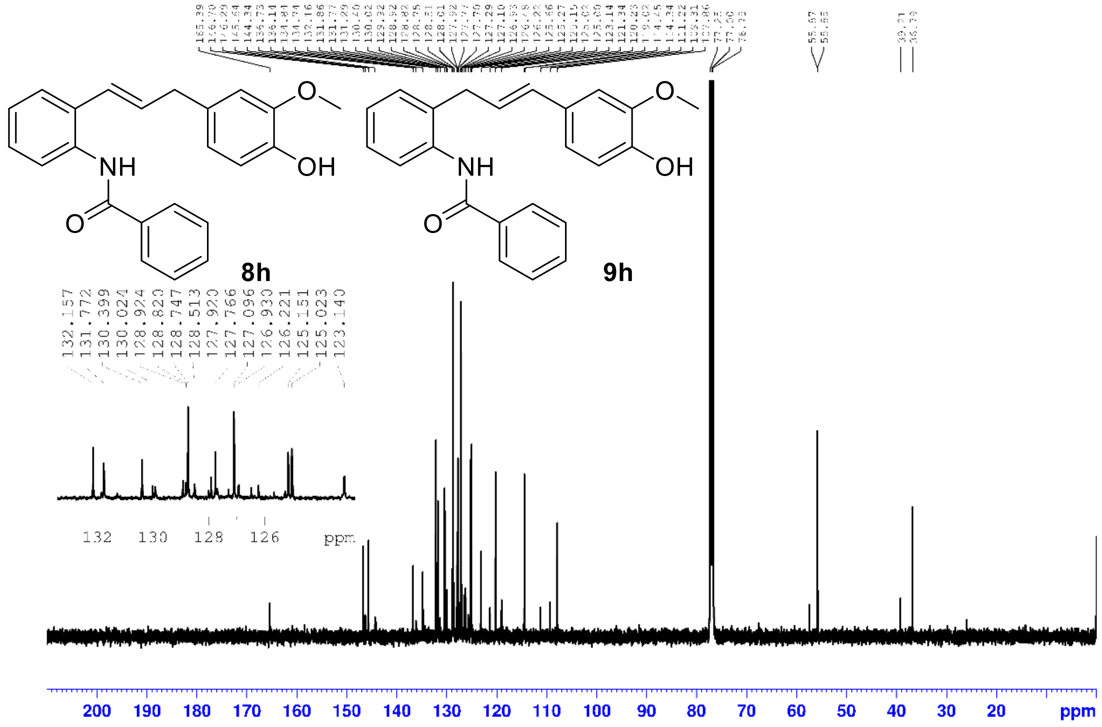

Figure S36: 500 MHz ^{1}H- and 126 MHz ^{13}C-NMR spectra of compound **8h,9h** in $CDCl_3$.

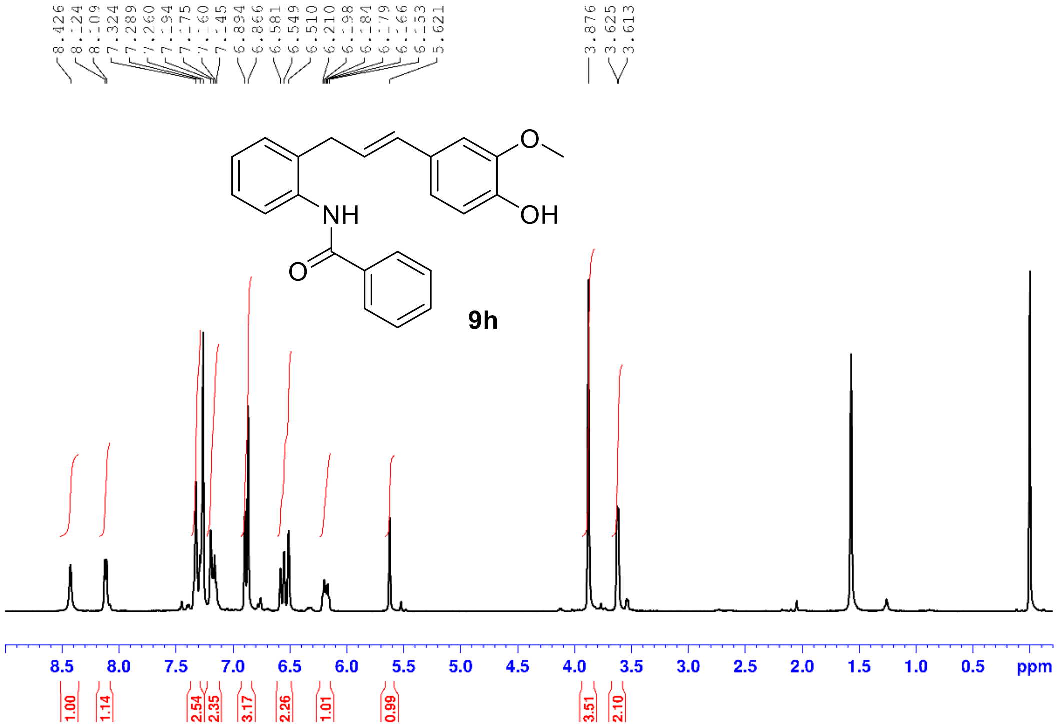

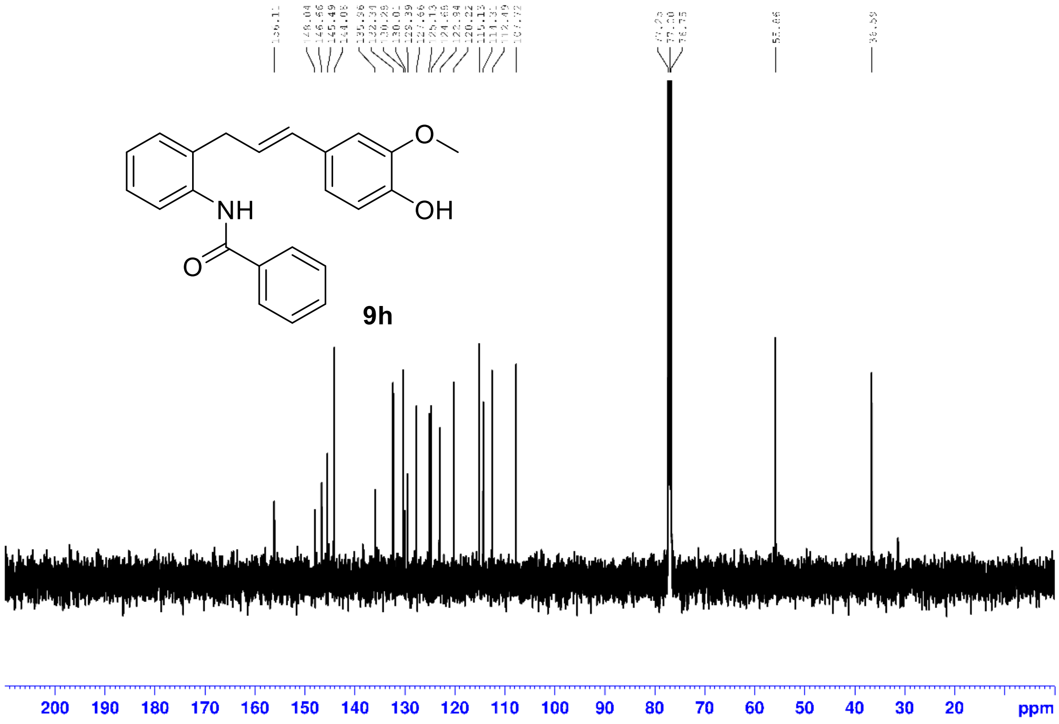

Figure S37: 500 MHz ^{1}H- and 126 MHz ^{13}C-NMR spectra of compound **9h** in $CDCl_3$.

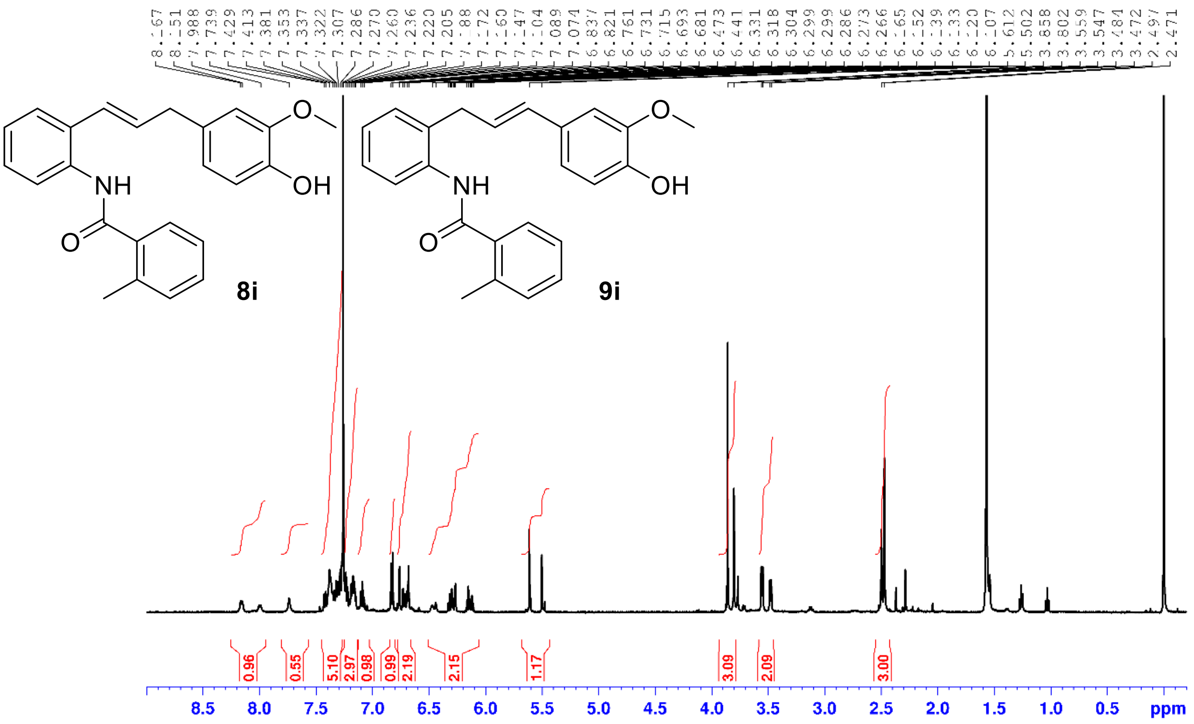

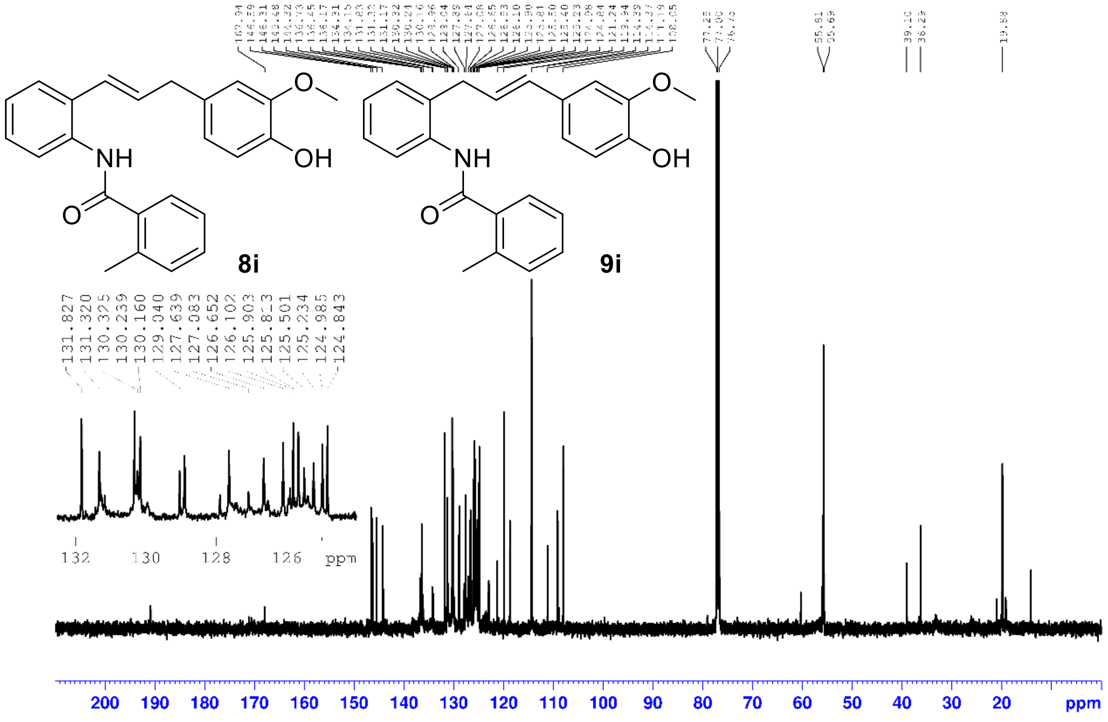

Figure S38: 500 MHz ^{1}H- and 126 MHz ^{13}C-NMR spectra of compound **8i,9i** in $CDCl_3$.

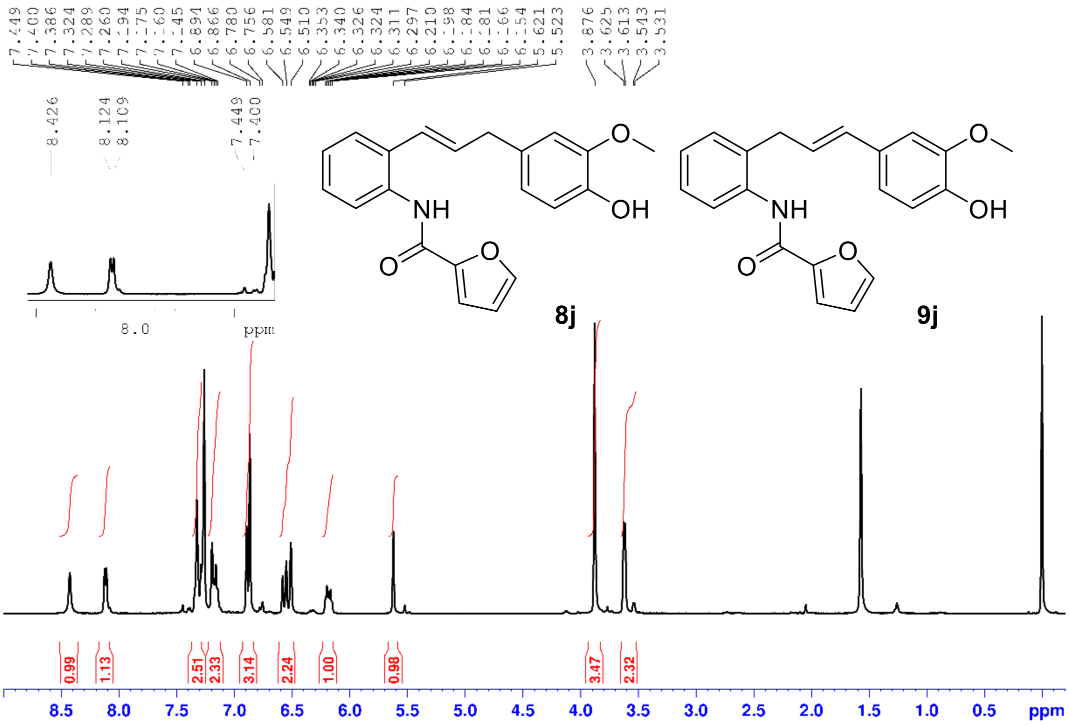

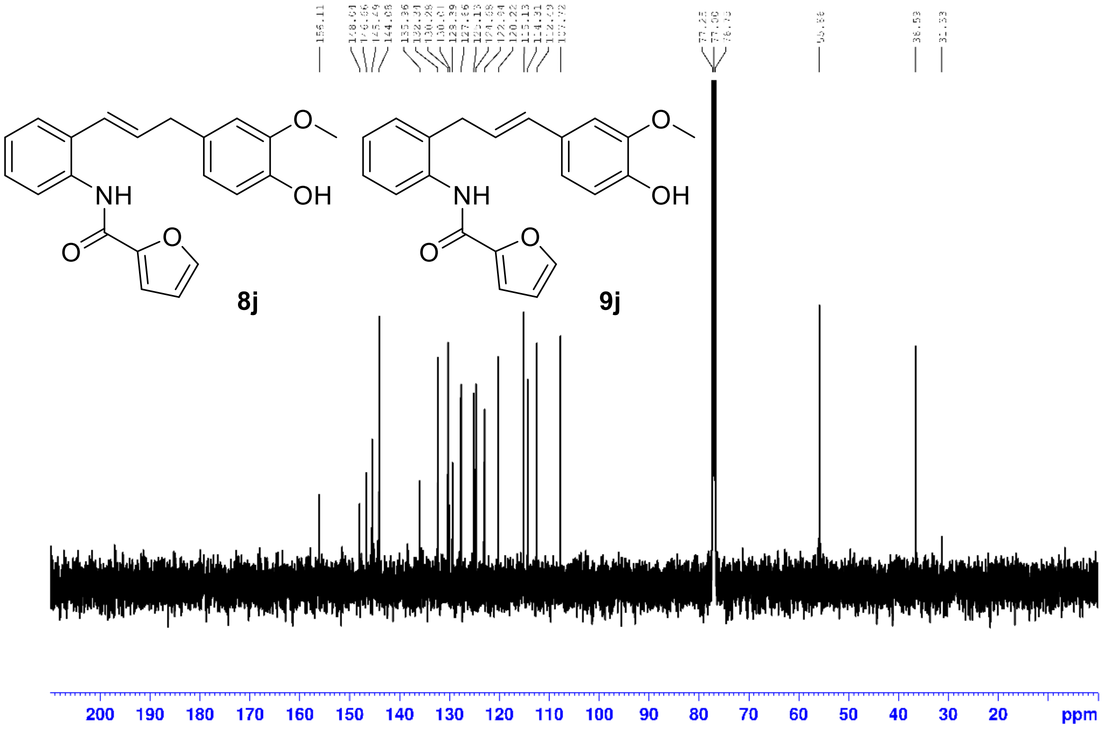

Figure S39: 500 MHz ^{1}H- and 126 MHz ^{13}C-NMR spectra of compound **8j,9j** in $CDCl_3$.

5. ATR-IR Spectra

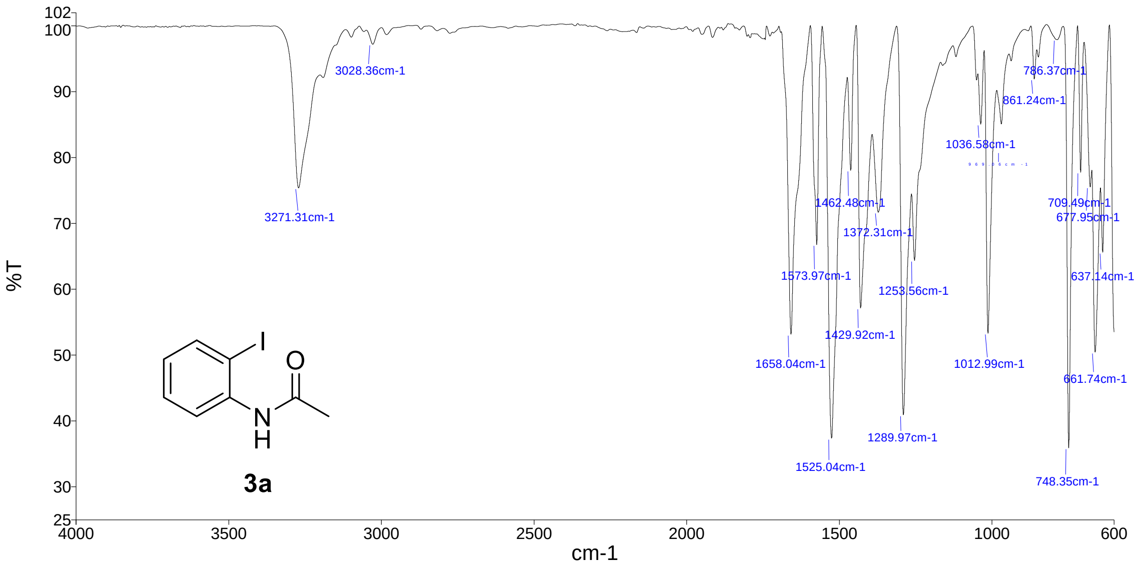

Figure S40: ATR-FTIR spectrum of compound **3a**.

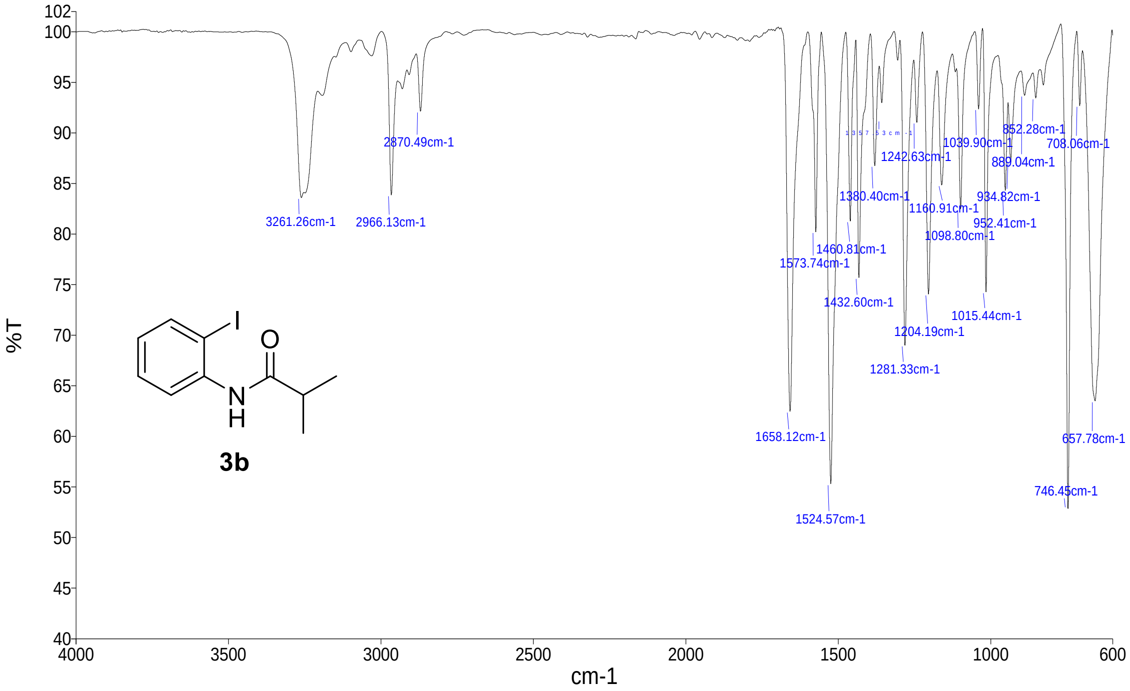

Figure S41: ATR-FTIR spectrum of compound **3b**.

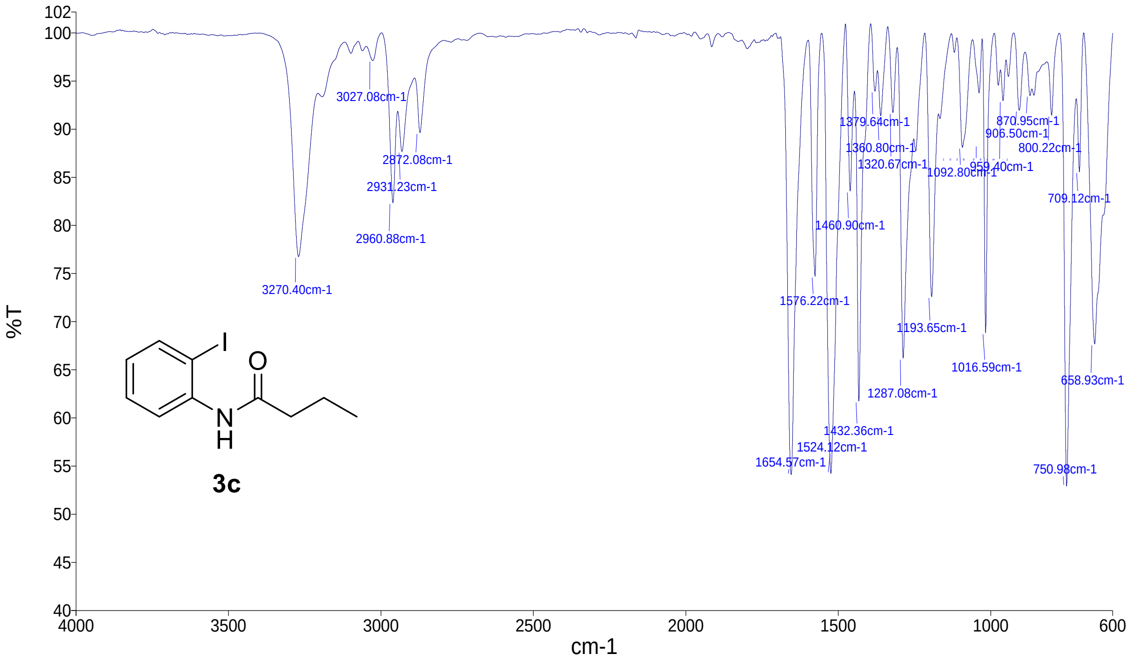

Figure S42: ATR-FTIR spectrum of compound **3c**.

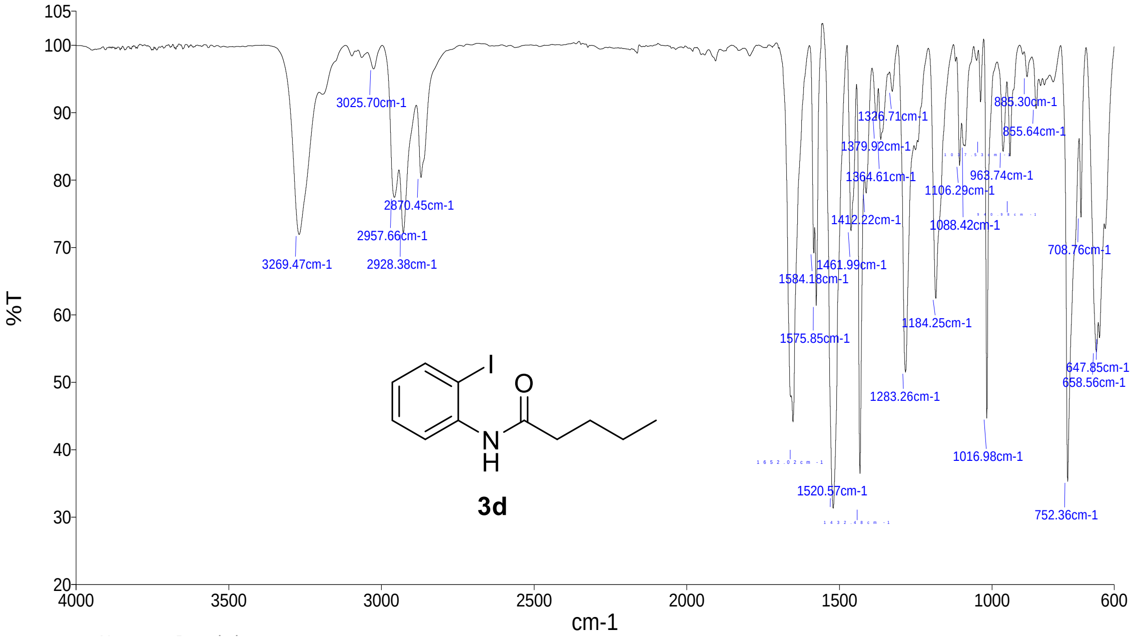

Figure S43: ATR-FTIR spectrum of compound **3d**.

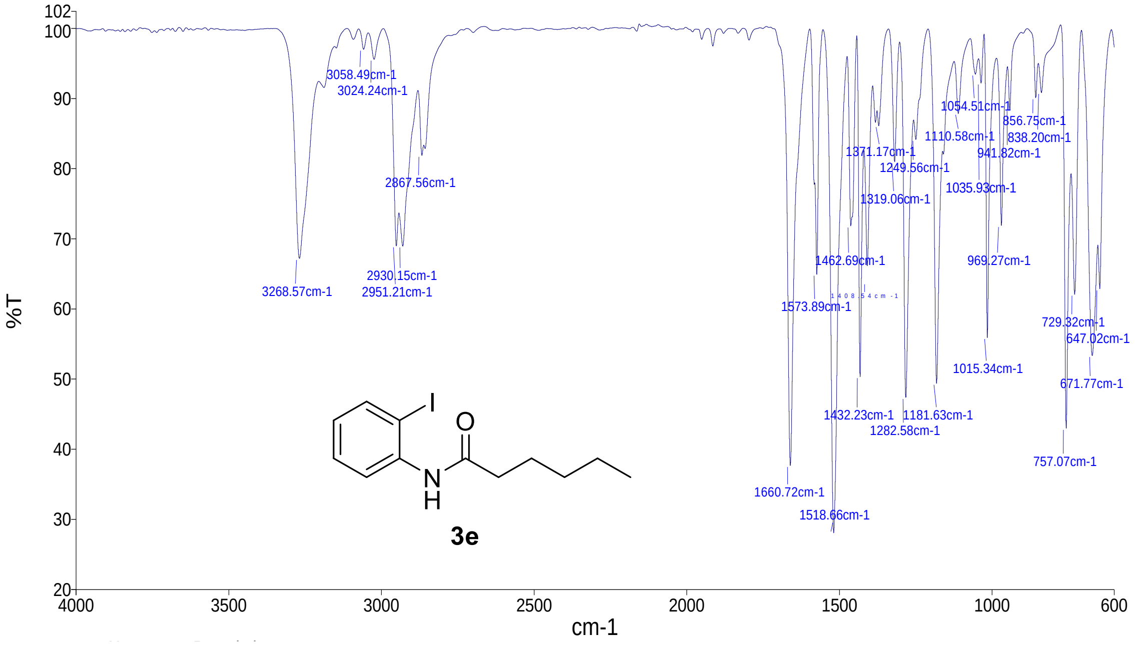

Figure S44: ATR-FTIR spectrum of compound **3e**.

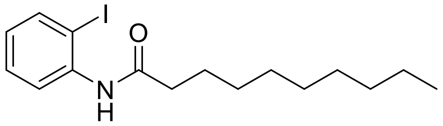

3f

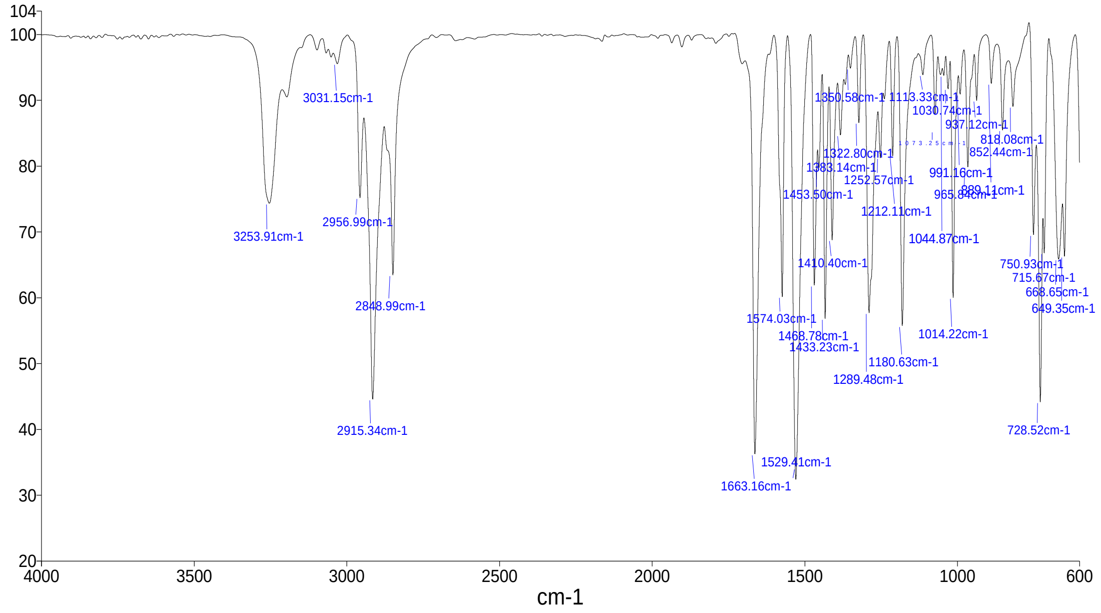

Figure S45: ATR-FTIR spectrum of compound **3f**.

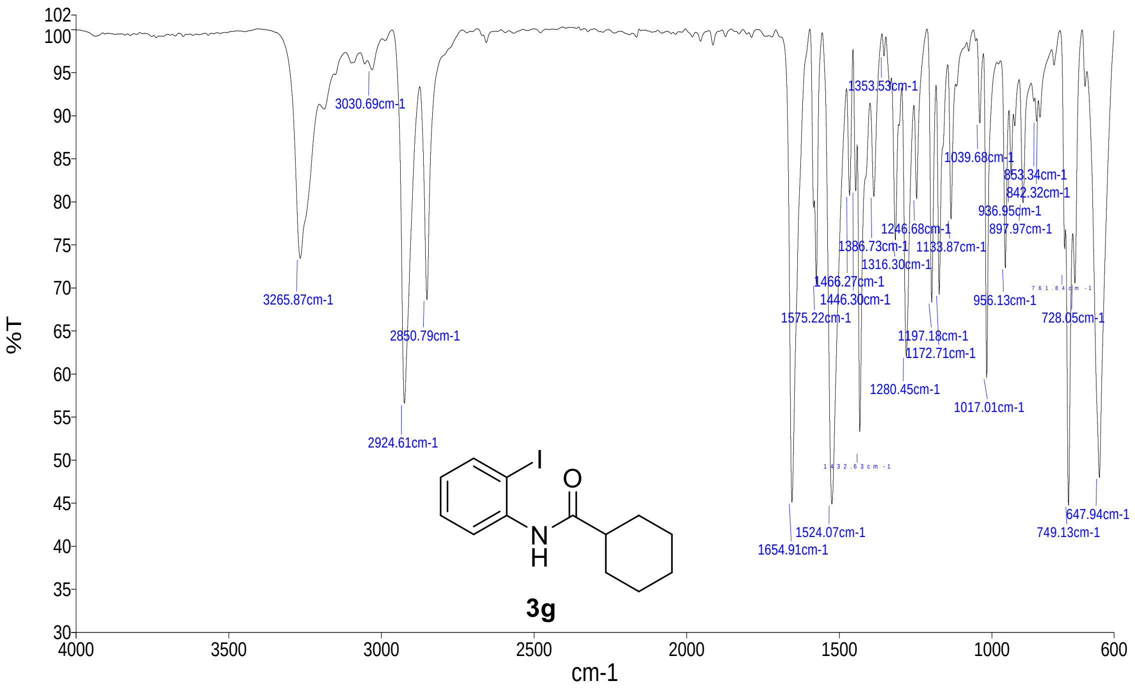

Figure S46: ATR-FTIR spectrum of compound **3g**.

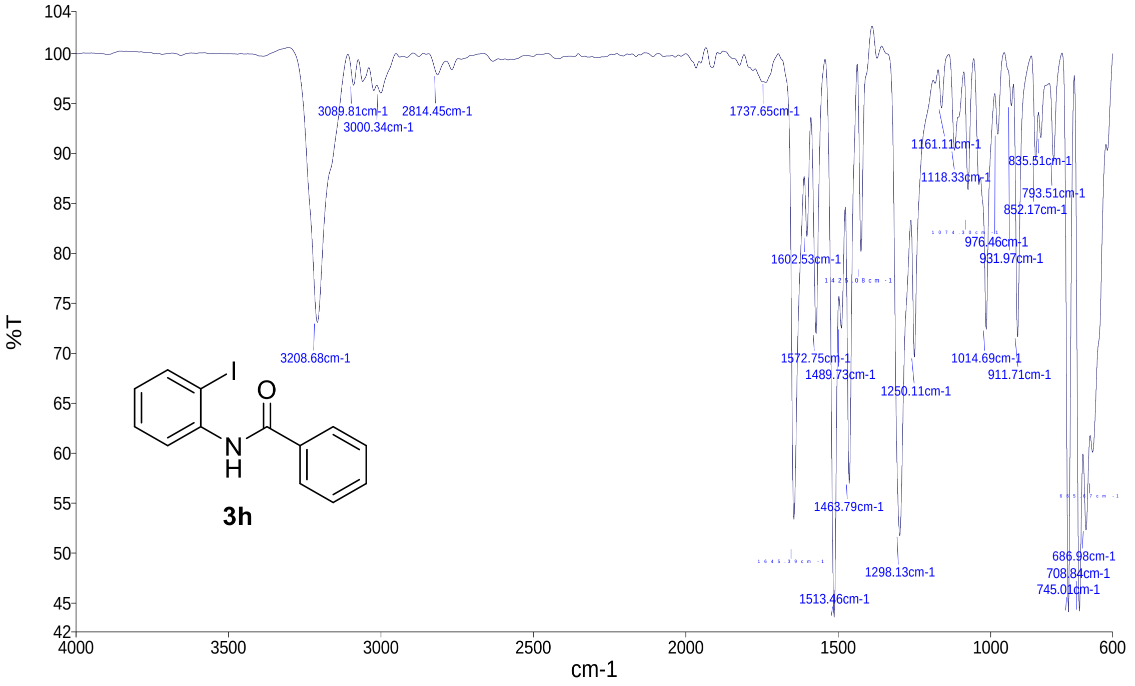

Figure S47: ATR-FTIR spectrum of compound **3h**.

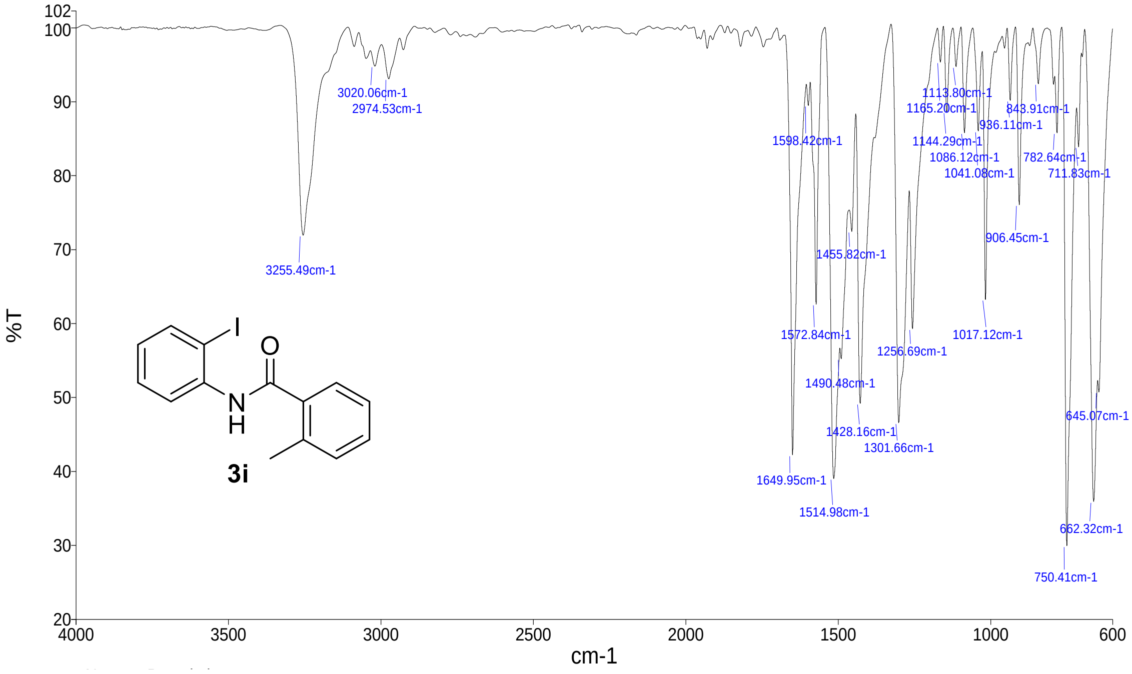

Figure S48: ATR-FTIR spectrum of compound **3i**.

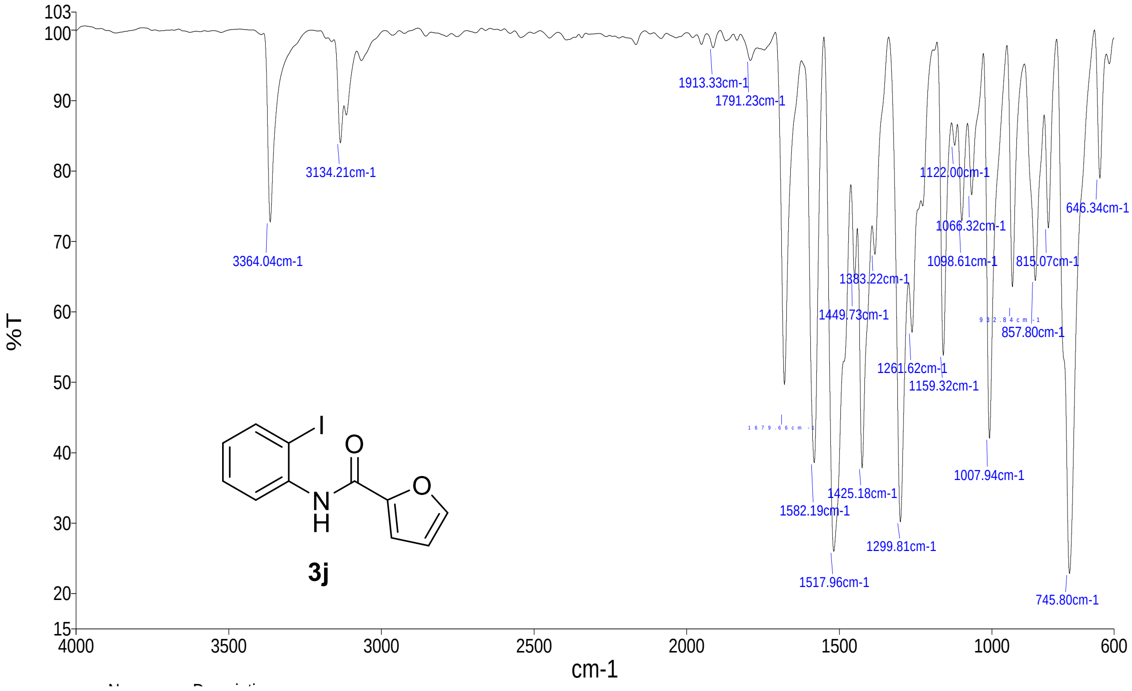

Figure S49: ATR-FTIR spectrum of compound **3j**.

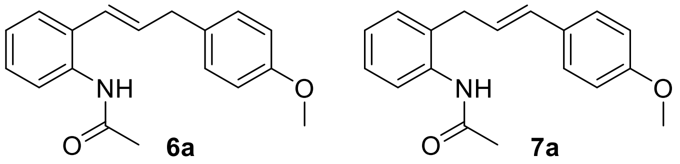

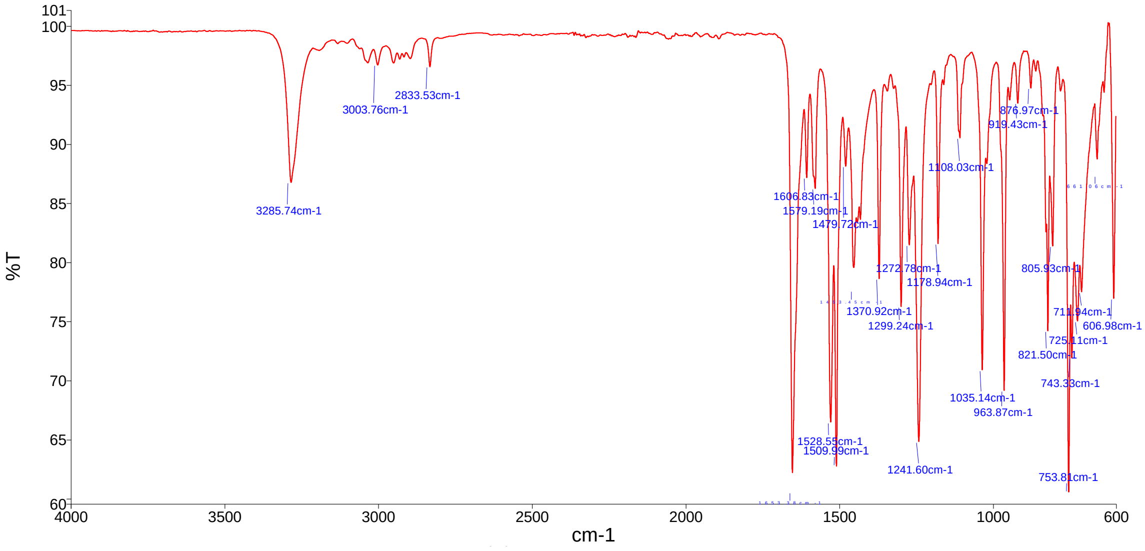

Figure S50: ATR-FTIR spectrum of compound **6a,7a**.

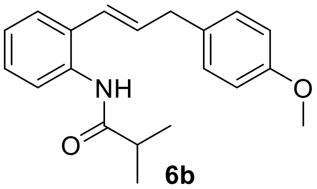

6b

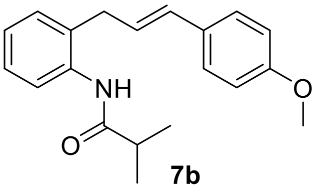

7b

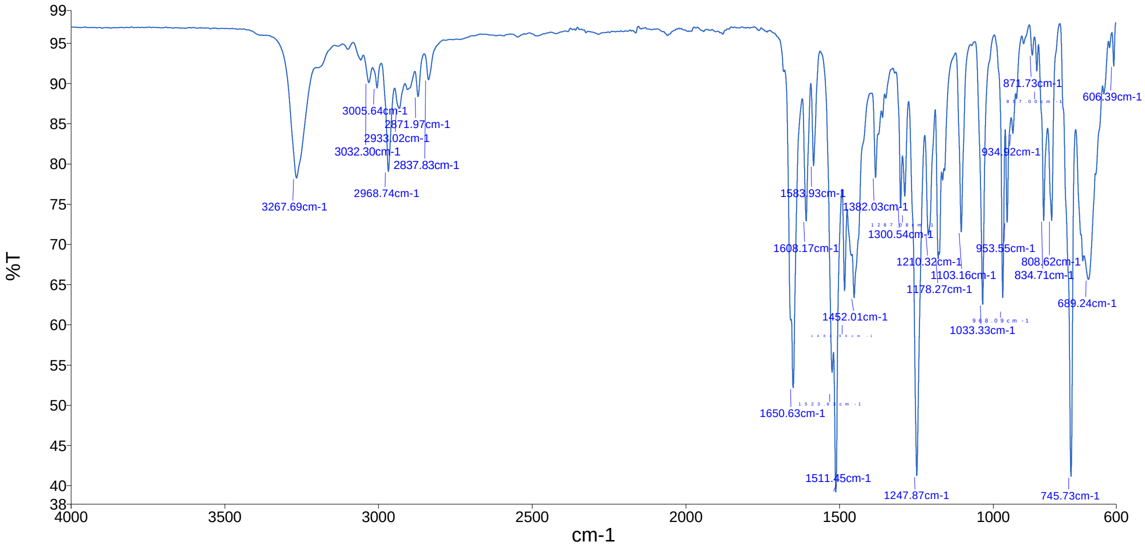

Figure S51: ATR-FTIR spectrum of compound **6b,7b**.

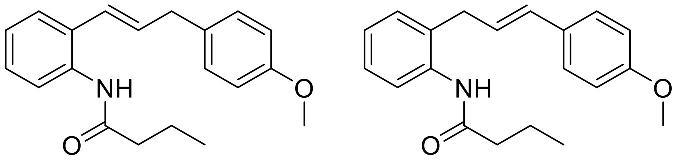

6c **7c**

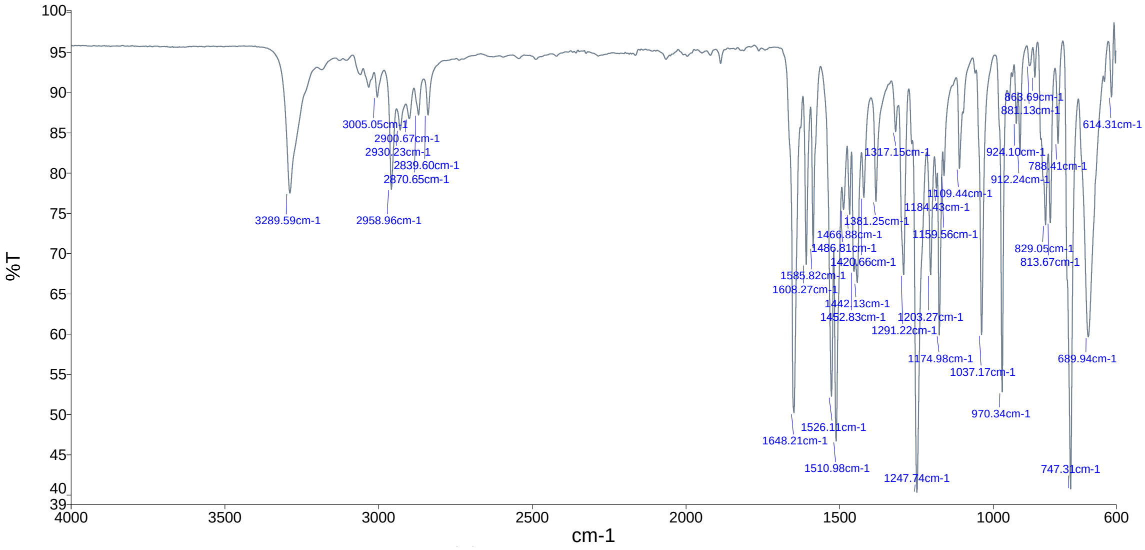

Figure S52: ATR-FTIR spectrum of compound **6c,7c**.

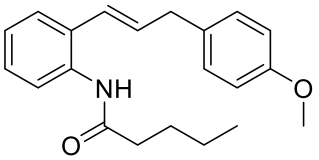

6d

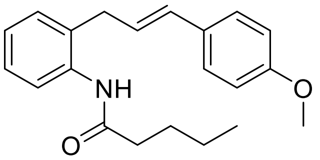

7d

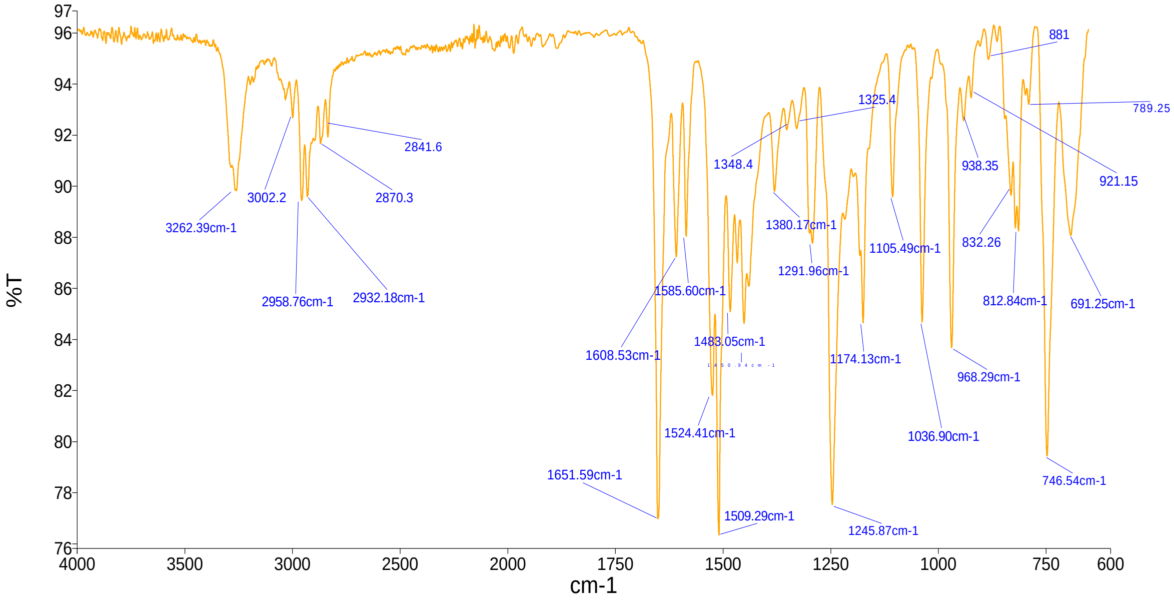

Figure S53: ATR-FTIR spectrum of compound **6d,7d**.

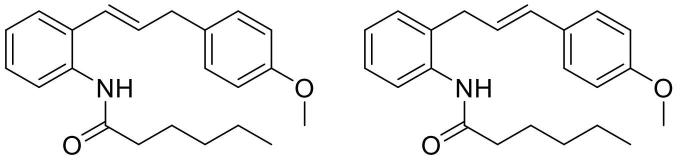

6e **7e**

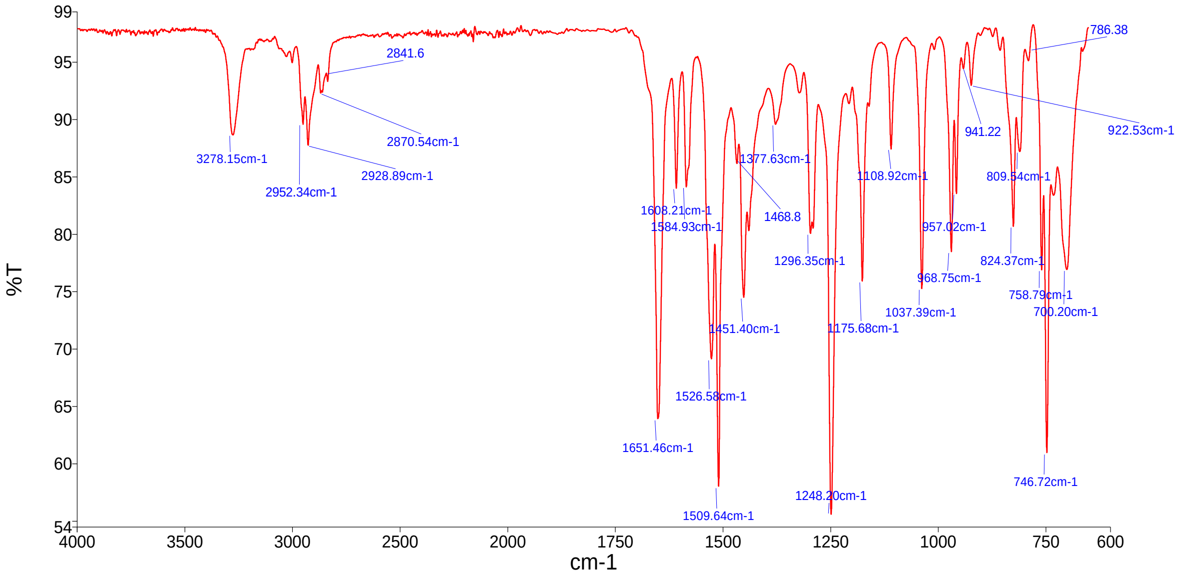

Figure S54: ATR-FTIR spectrum of compound **6e,7e**.

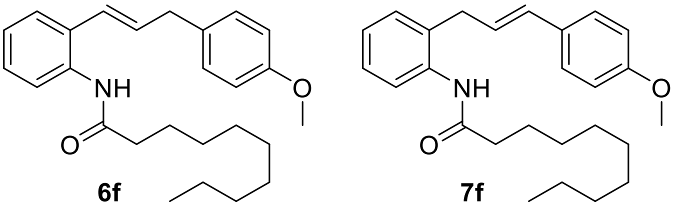

6f 7f

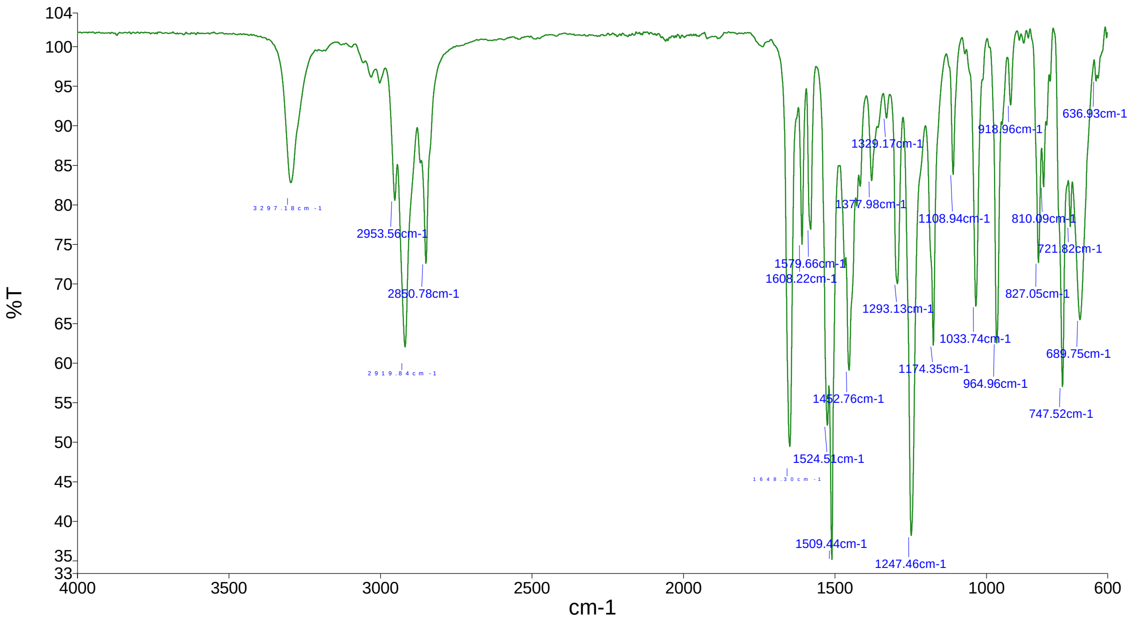

Figure S55: ATR-FTIR spectrum of compound **6f,7f**.

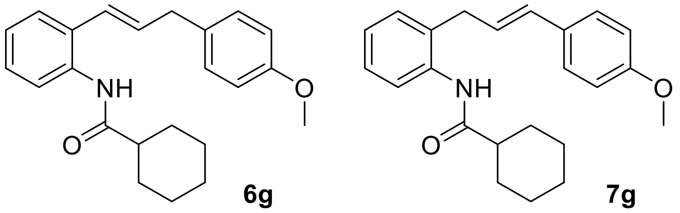

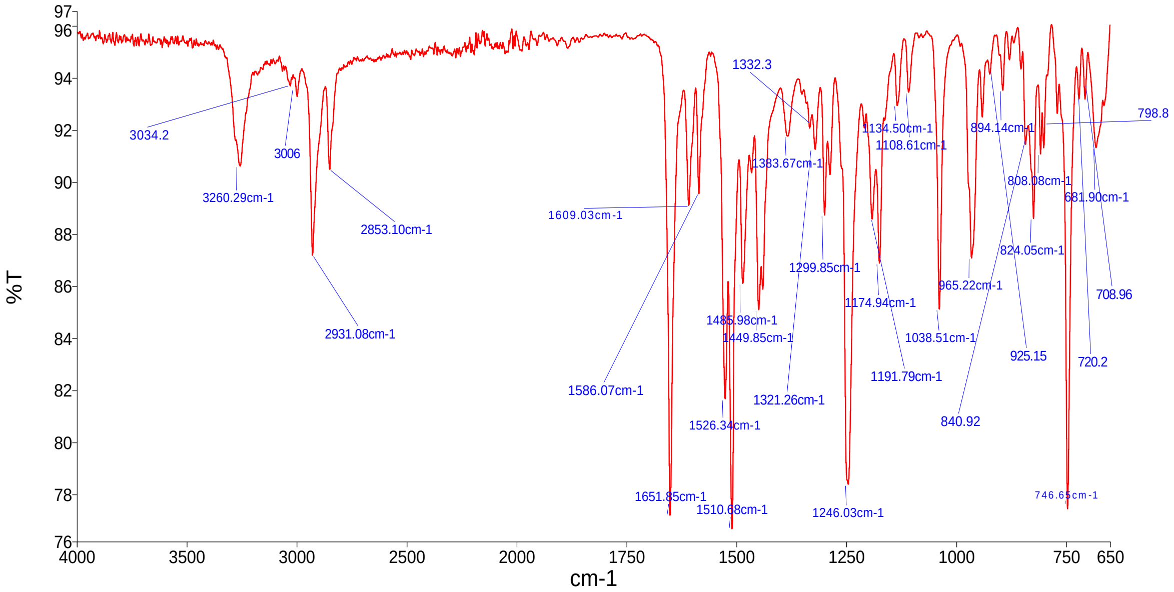

Figure S56: ATR-FTIR spectrum of compound **6g,7g**.

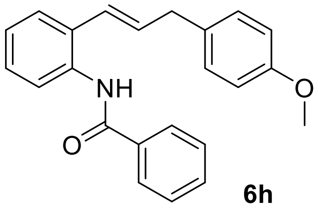

6h

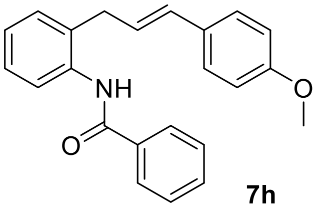

7h

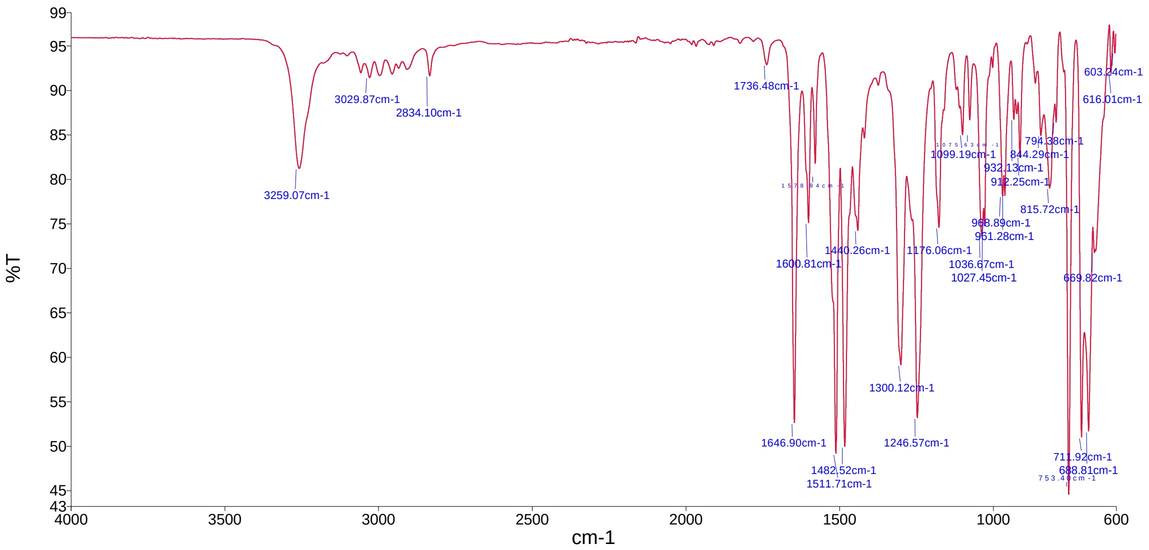

Figure S57: ATR-FTIR spectrum of compound **6h,7h**.

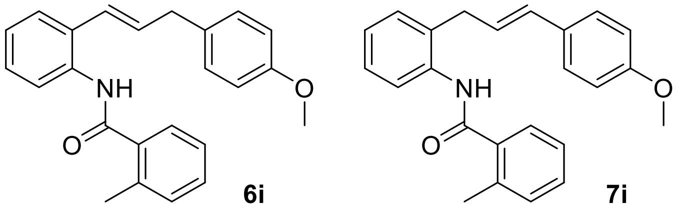

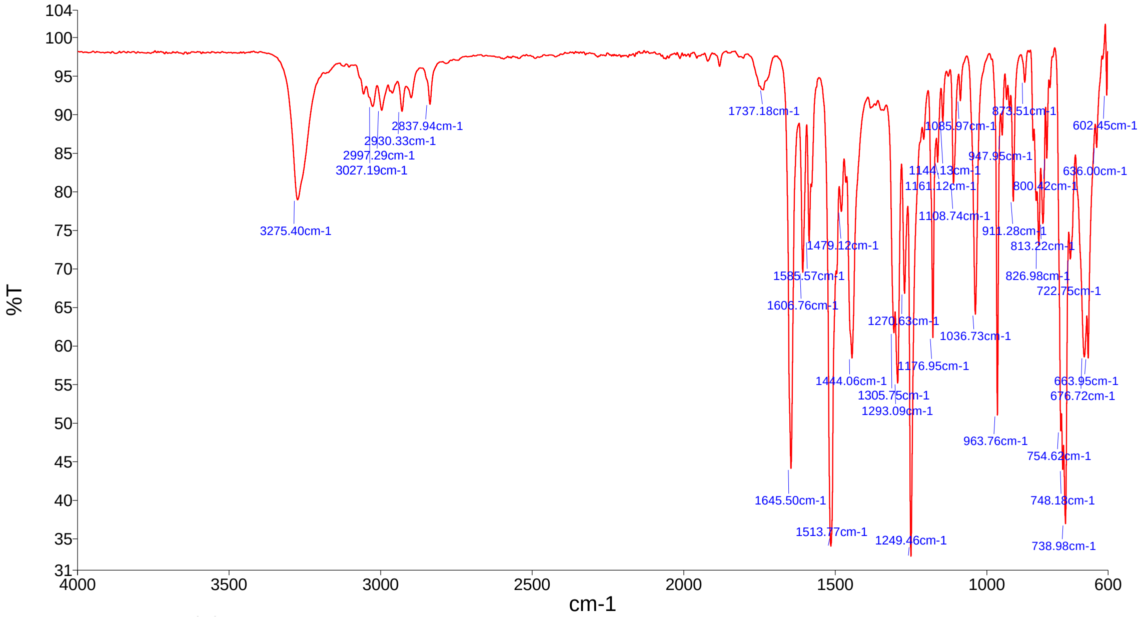

Figure S58: ATR-FTIR spectrum of compound **6i,7i**.

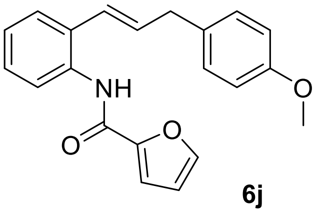

6j

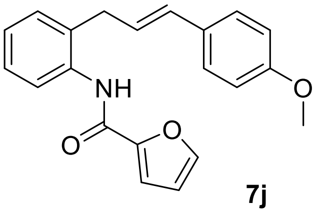

7j

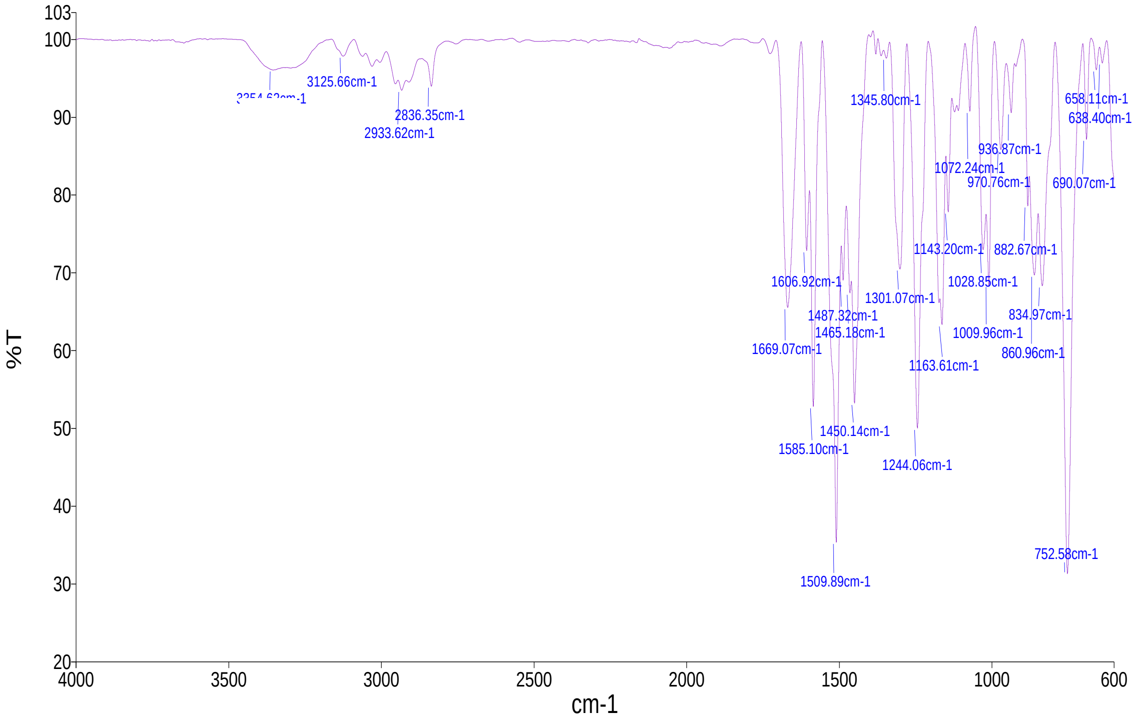

Figure S59: ATR-FTIR spectrum of compound **6j,7j**.

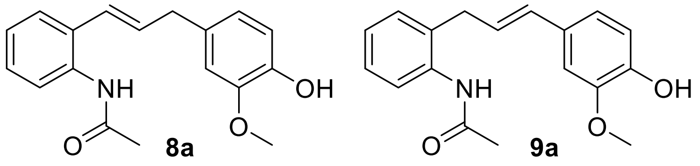

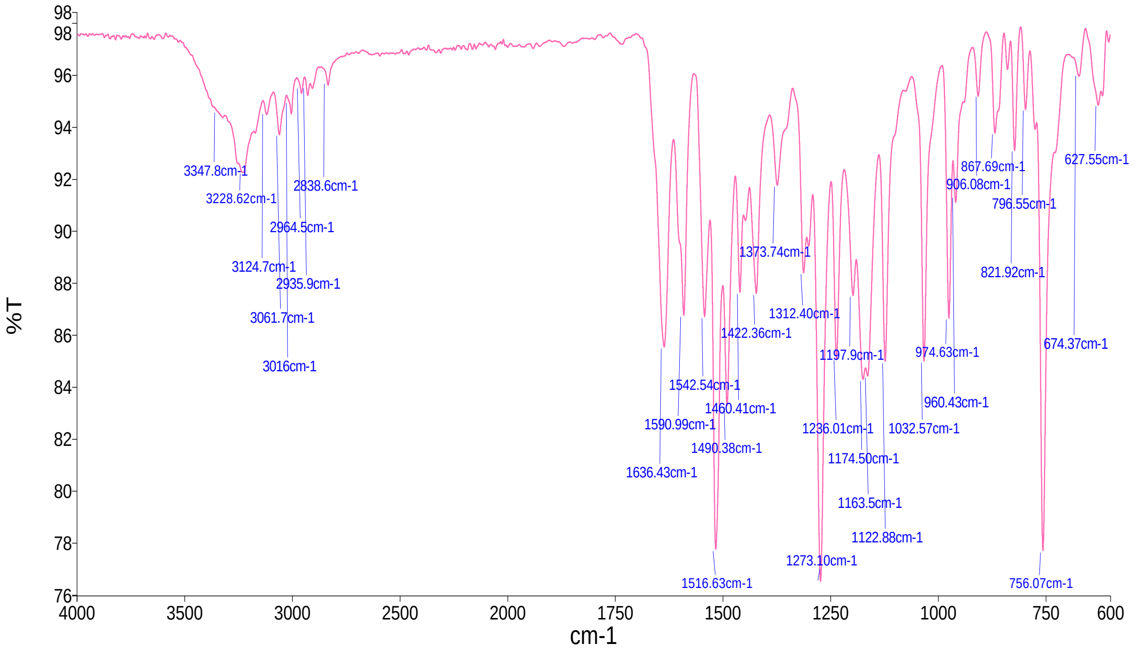

Figure S60: ATR-FTIR spectrum of compound **8a,9a**.

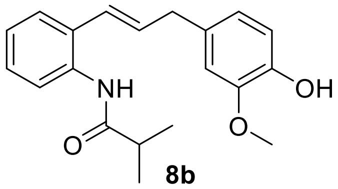

8b

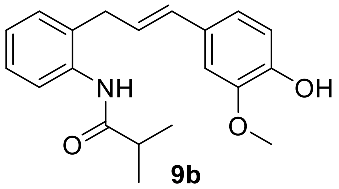

9b

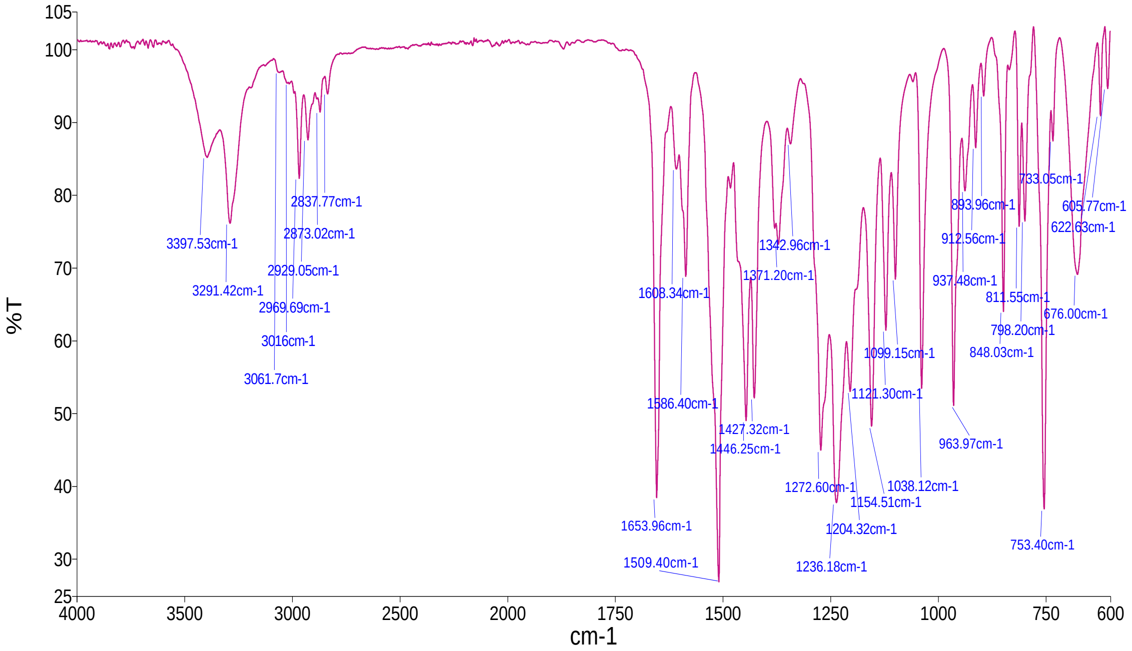

Figure S61: ATR-FTIR spectrum of compound **8b,9b**.

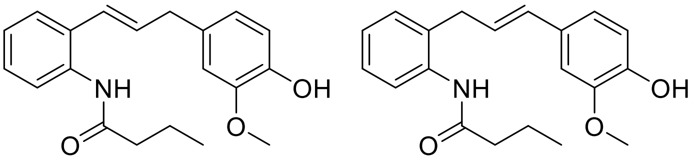

8c **9c**

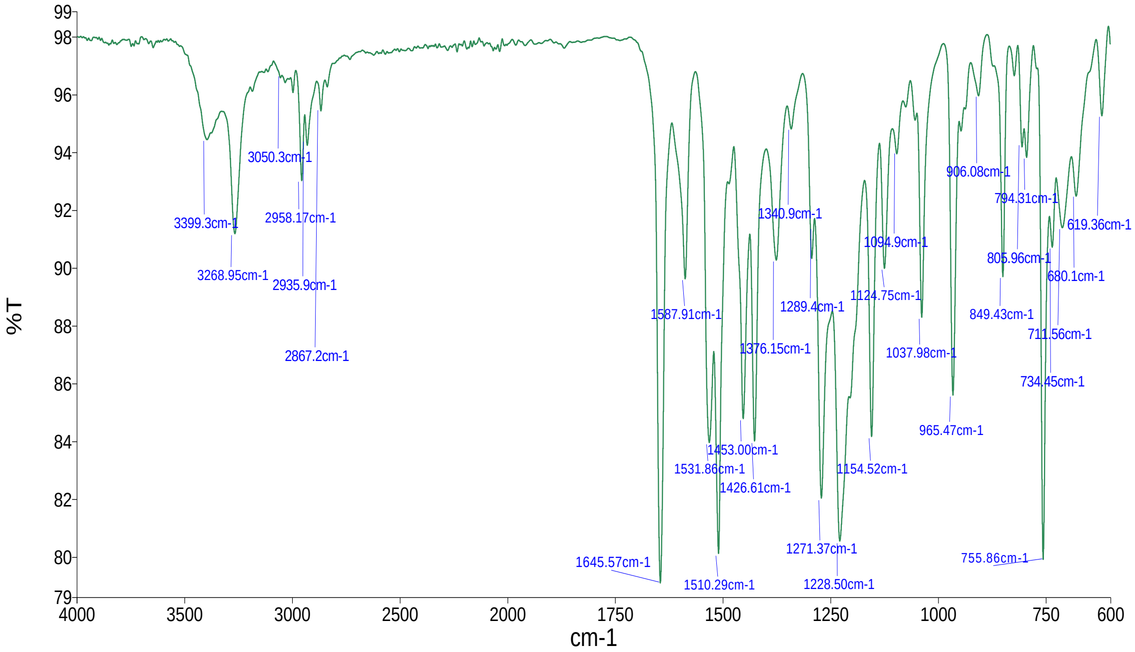

Figure S62: ATR-FTIR spectrum of compound **8c,9c**.

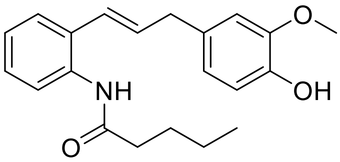

8d

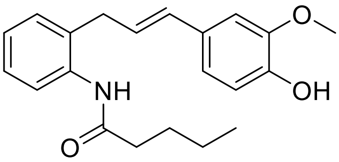

9d

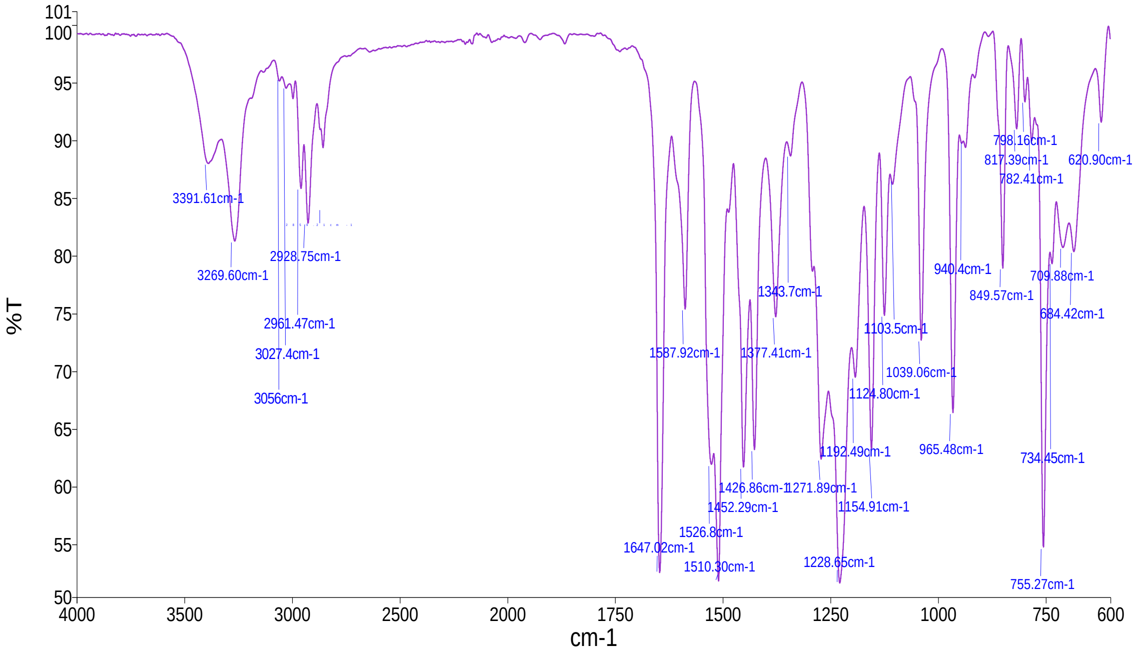

Figure S63: ATR-FTIR spectrum of compound **8d,9d**.

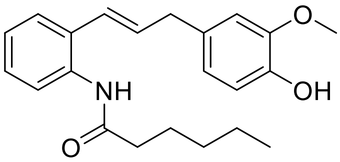

8e

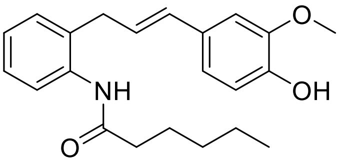

9e

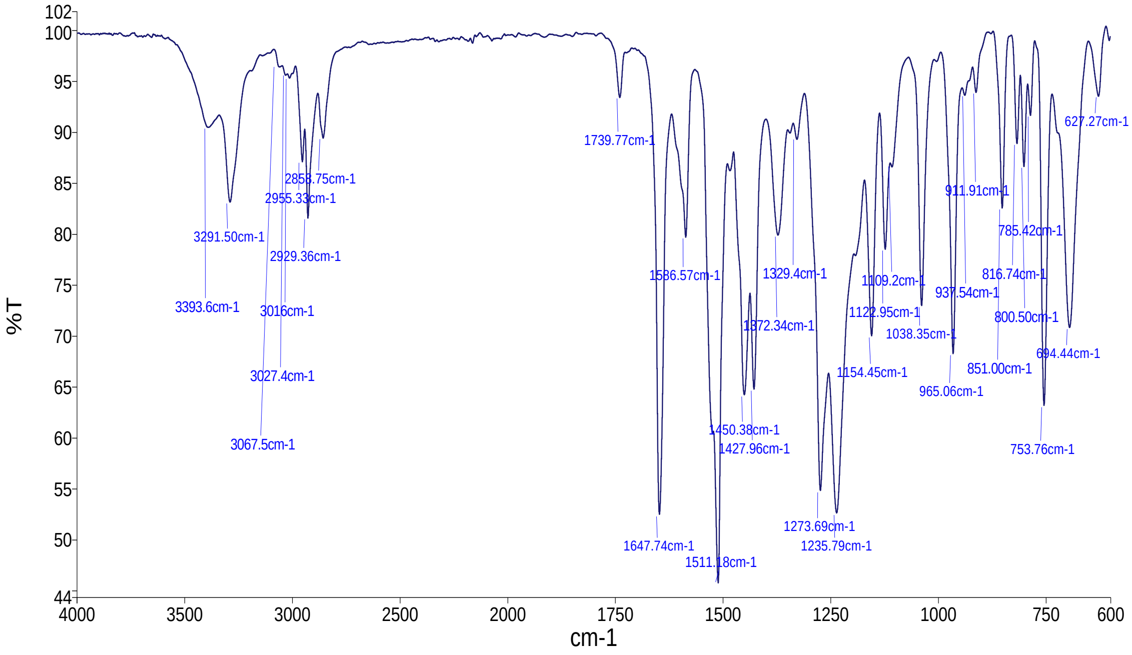

Figure S64: ATR-FTIR spectrum of compound **8e,9e**.

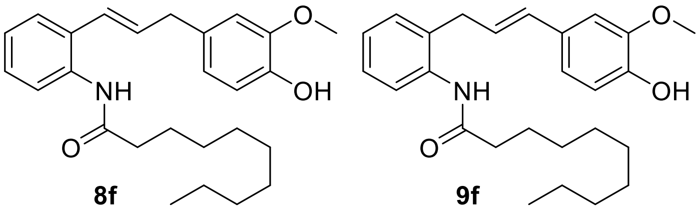

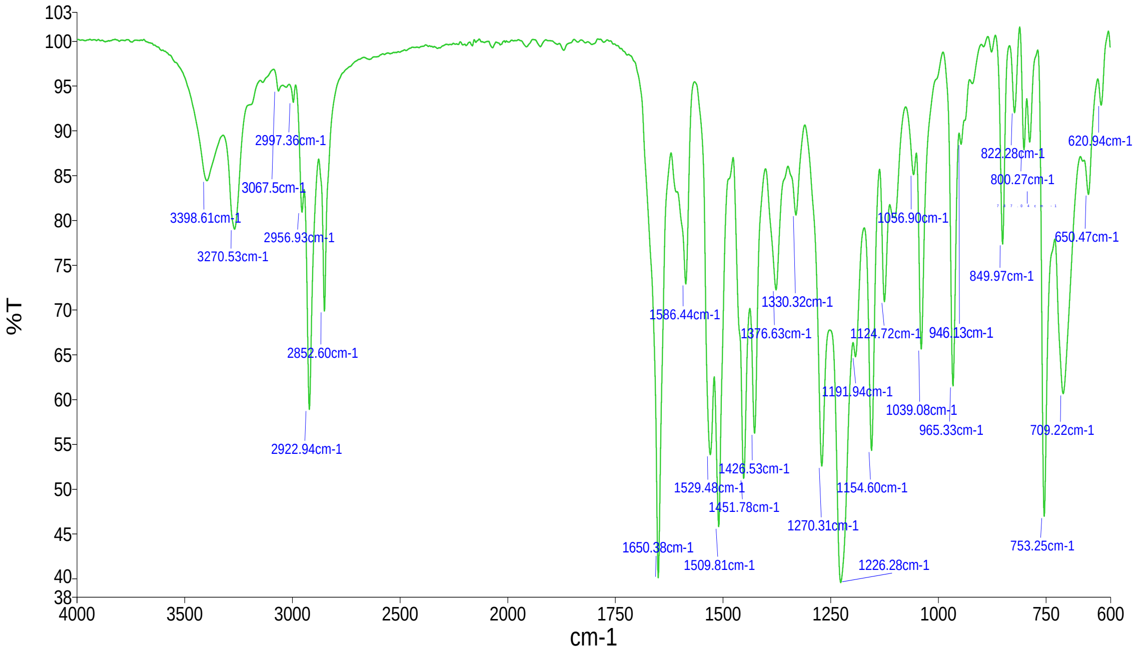

Figure S65: ATR-FTIR spectrum of compound **8f,9f**.

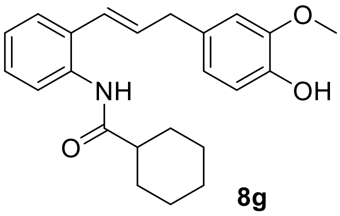

8g

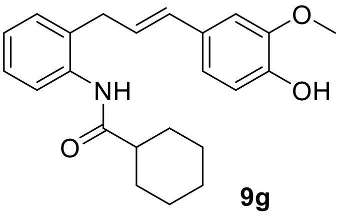

9g

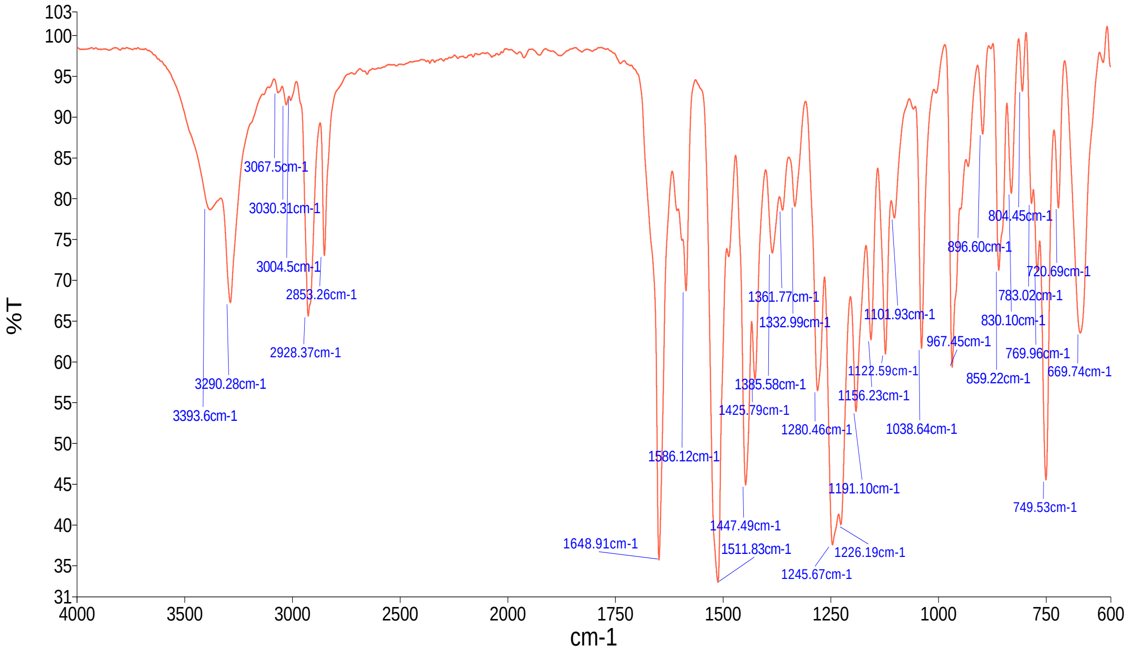

Figure S66: ATR-FTIR spectrum of compound **8g**,**9g**.

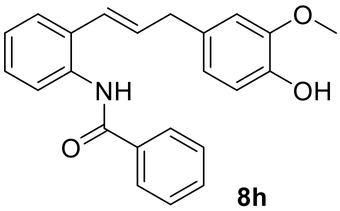

8h

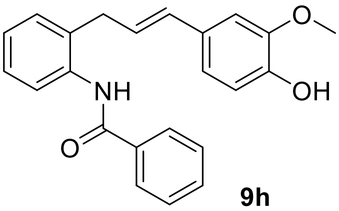

9h

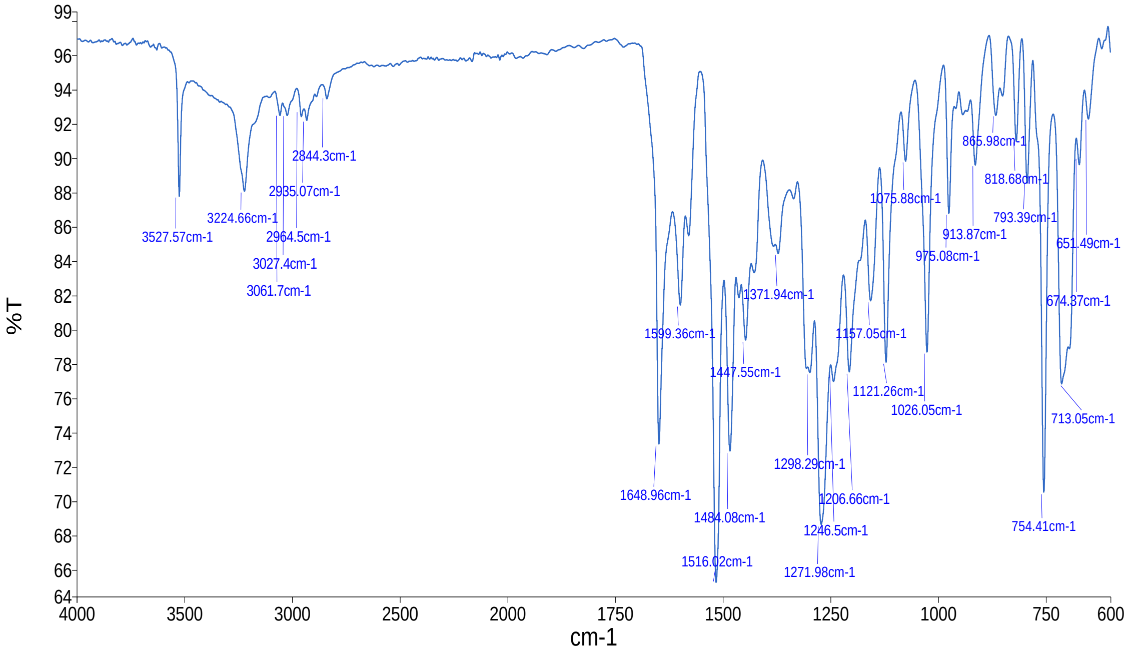

Figure S67: ATR-FTIR spectrum of compound **8h,9h**.

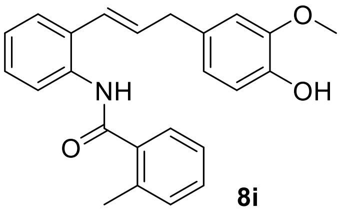

8i

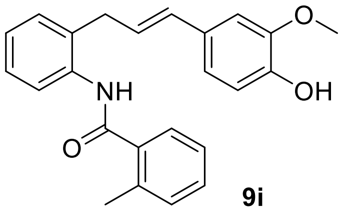

9i

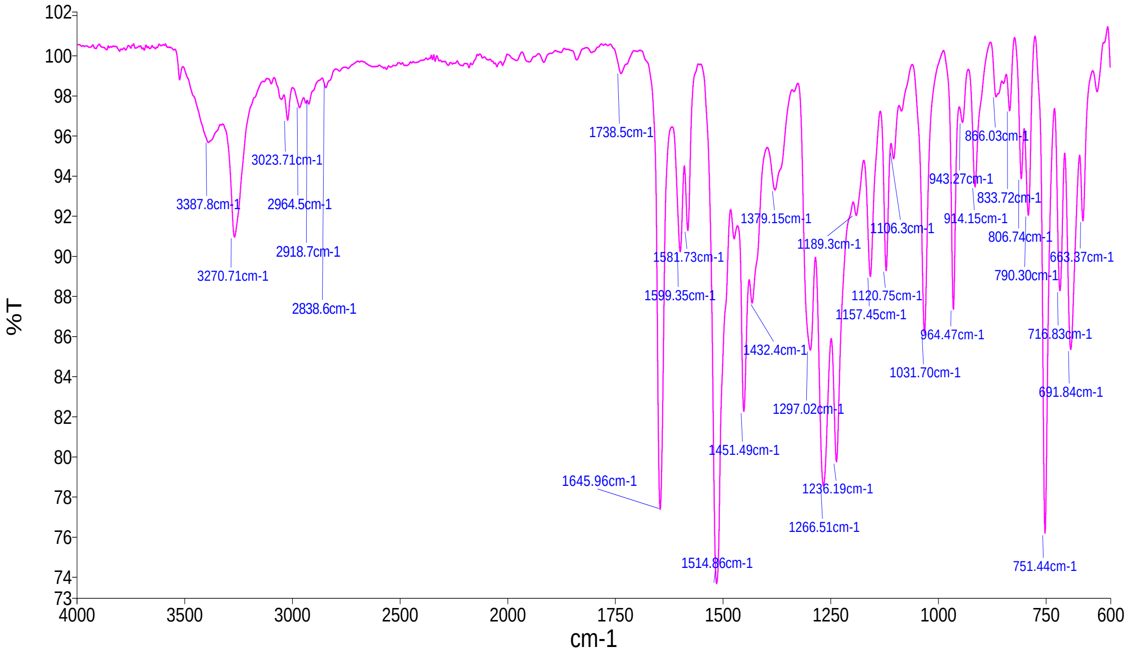

Figure S68: ATR-FTIR spectrum of compound **8i,9i**.

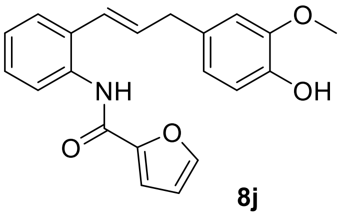

8j

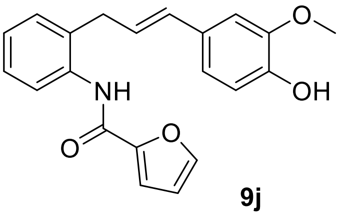

9j

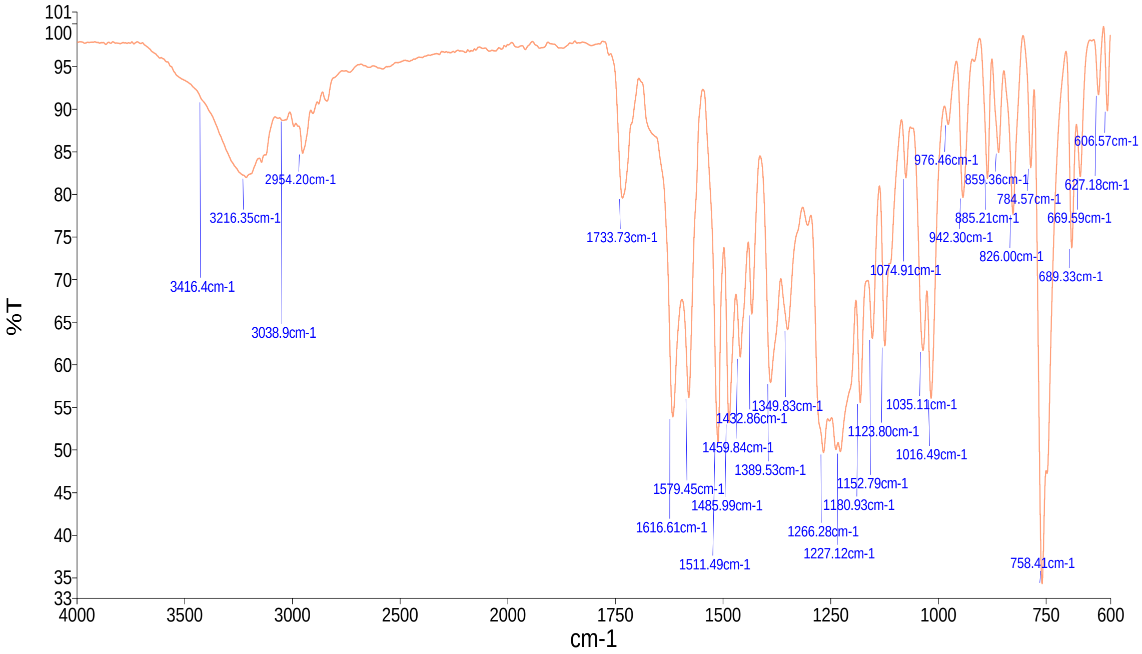

Figure S69: ATR-FTIR spectrum of compound **8j,9j**.

6. HR-MS Spectra

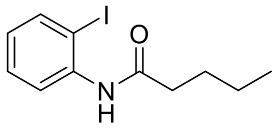

3d

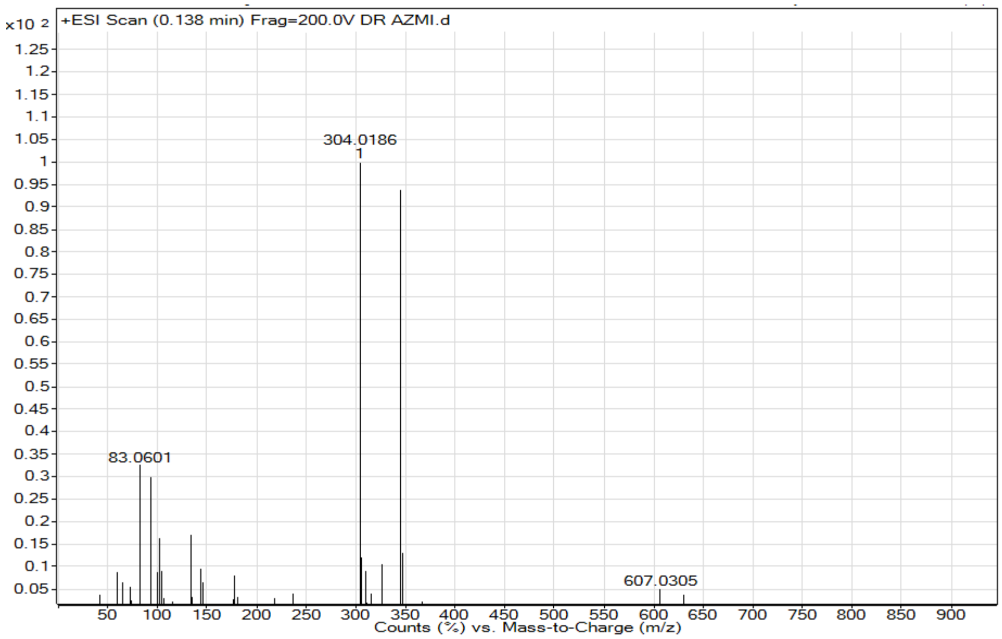

Figure S70: HR-MS spectrum of compound **3d**.

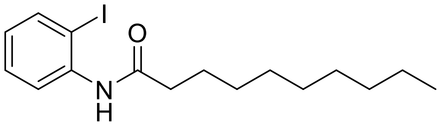

3f

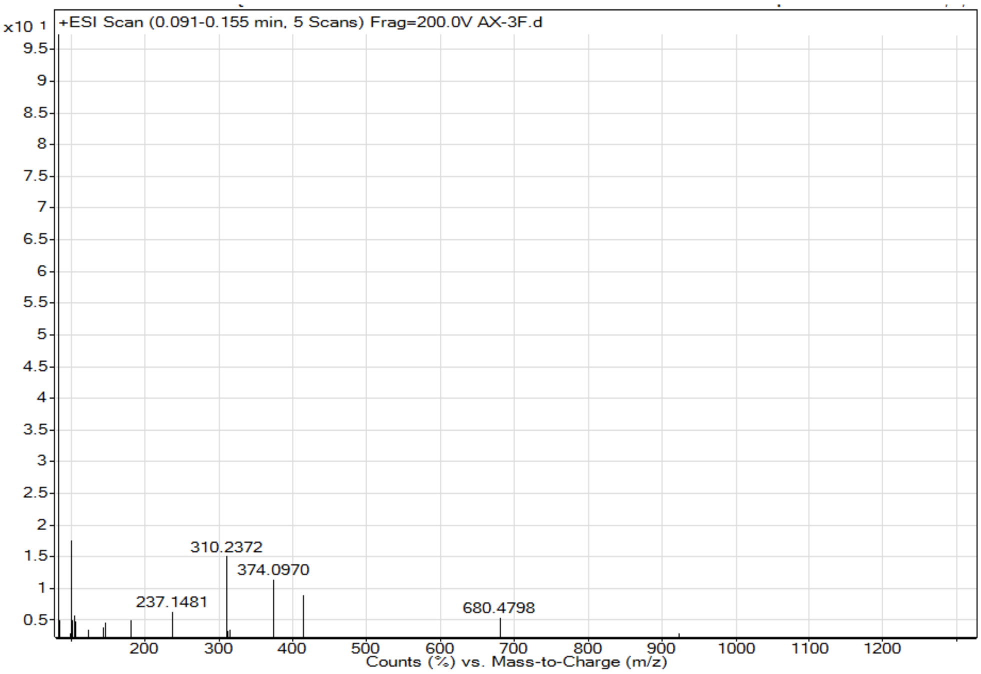

Figure S71: HR-MS spectrum of compound **3f**.

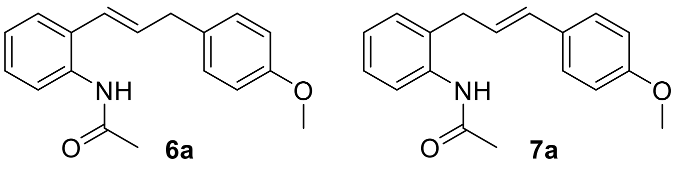

6a 7a

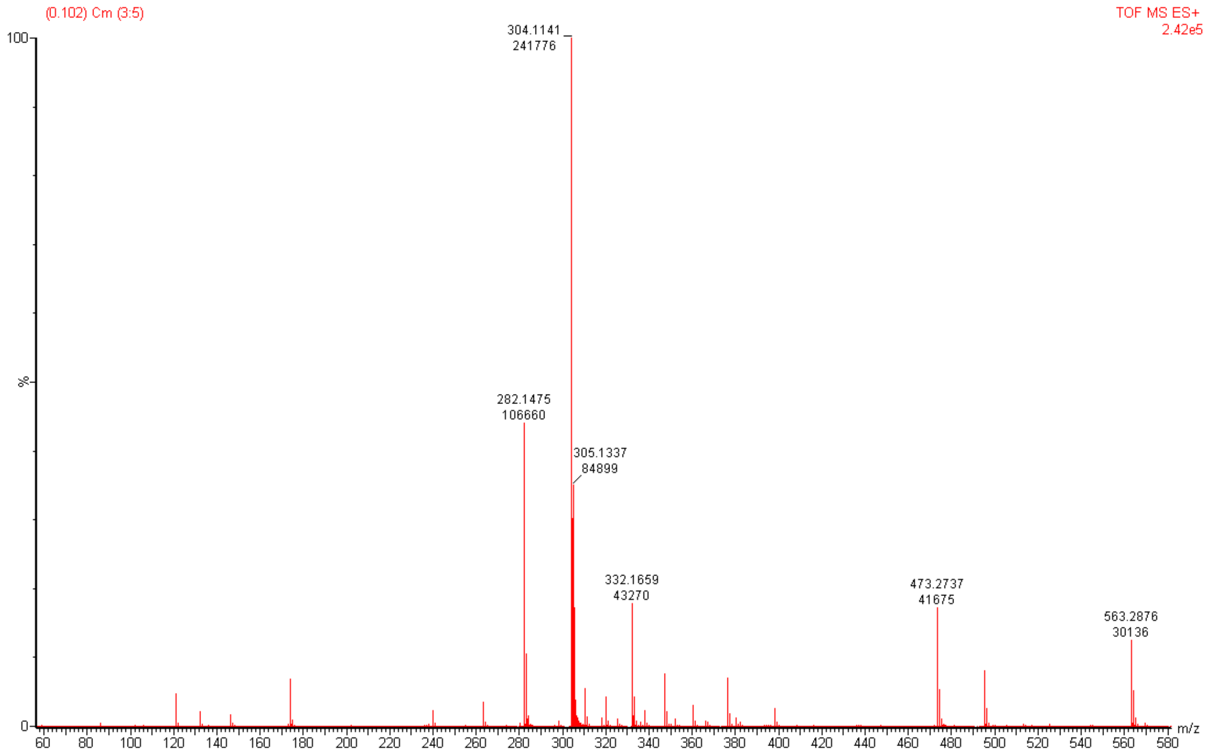

Figure S72: HR-MS spectrum of compound **6a,7a**.

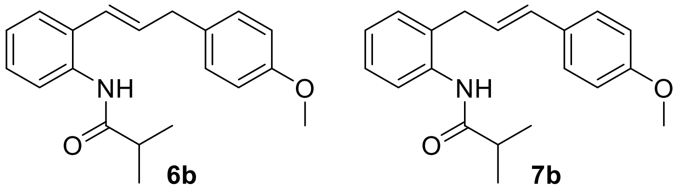

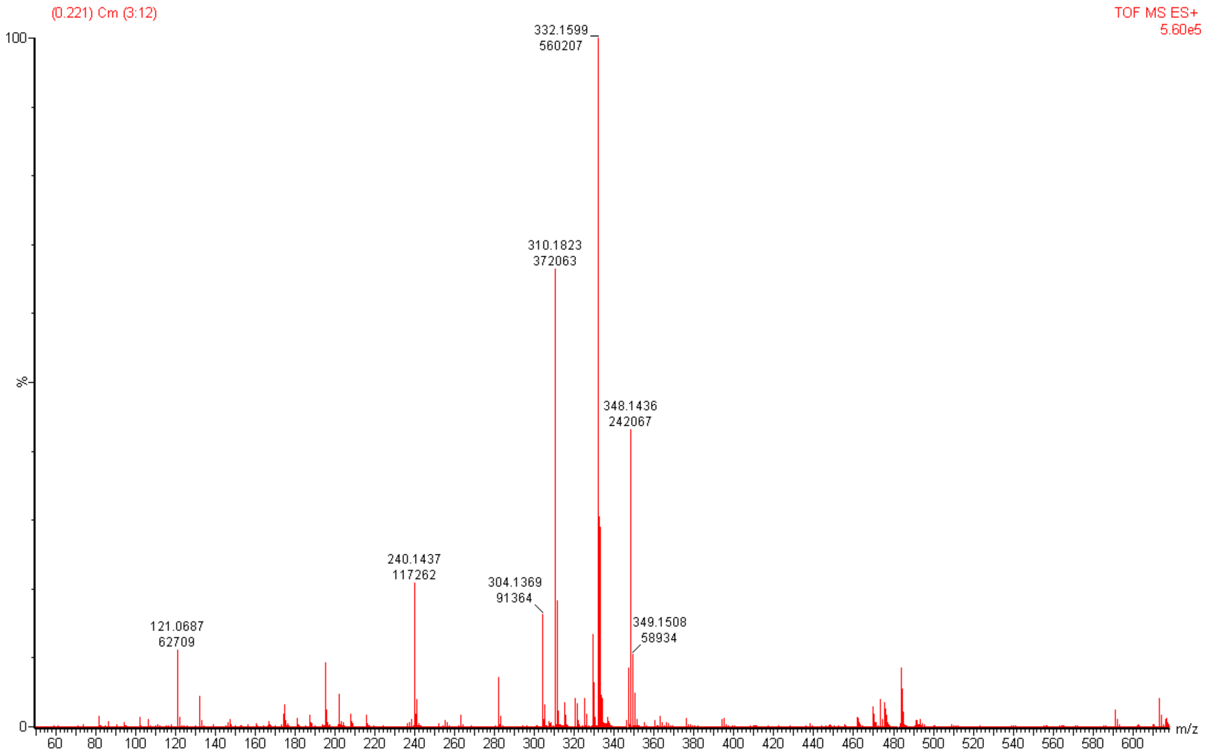

Figure S73: HR-MS spectrum of compound **6b,7b**.

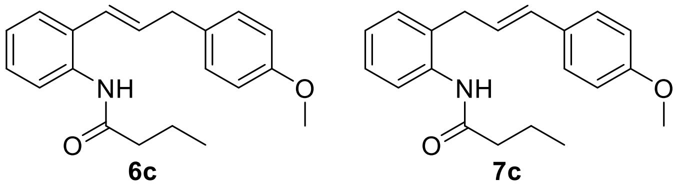

6c

7c

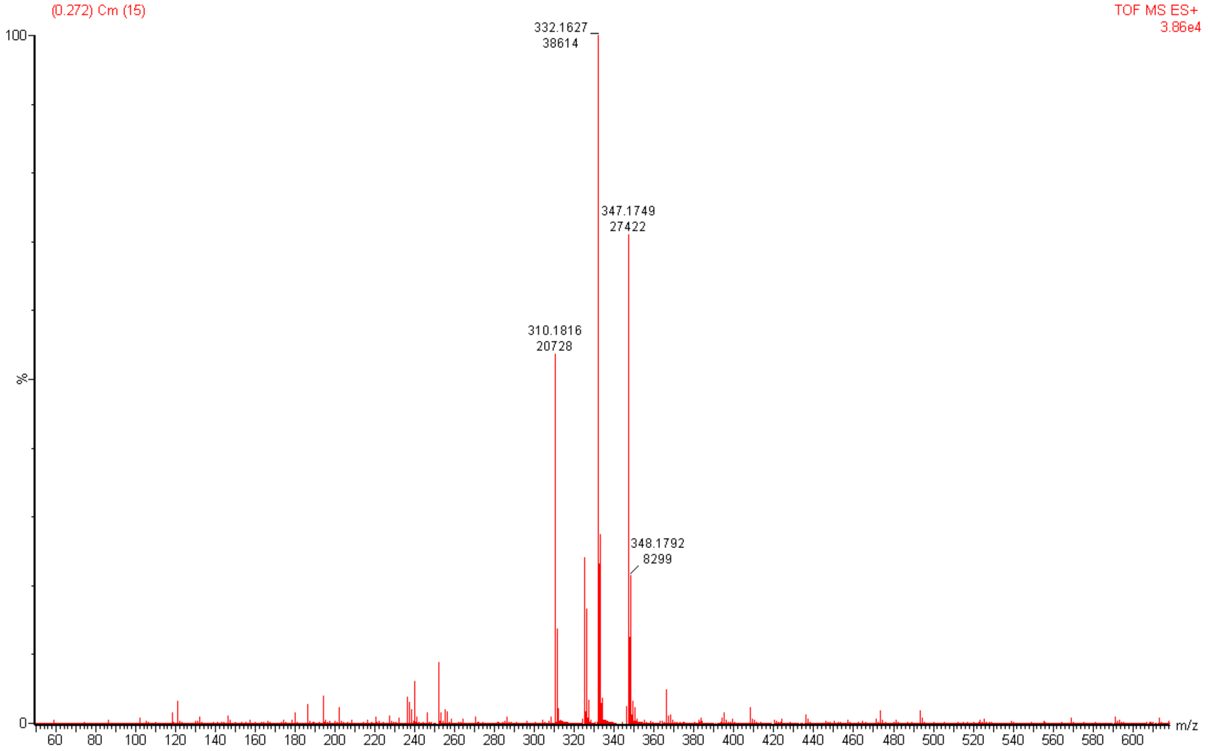

Figure S74: HR-MS spectrum of compound **6c,7c**.

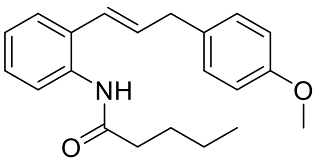

6d

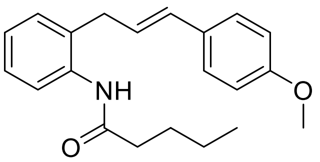

7d

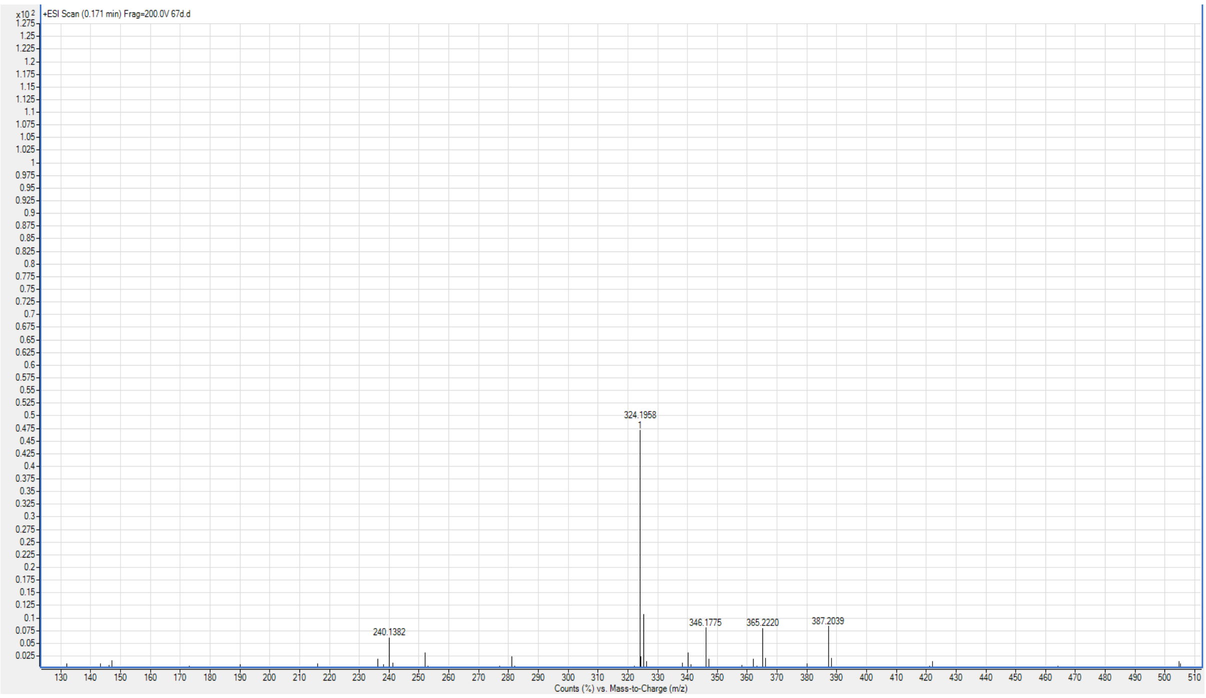

Figure S75: HR-MS spectrum of compound **6d,7d**.

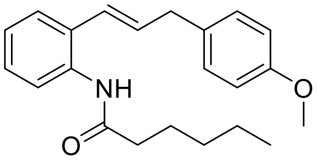

6e

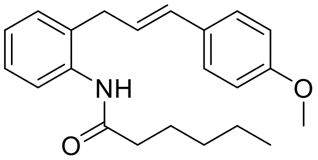

7e

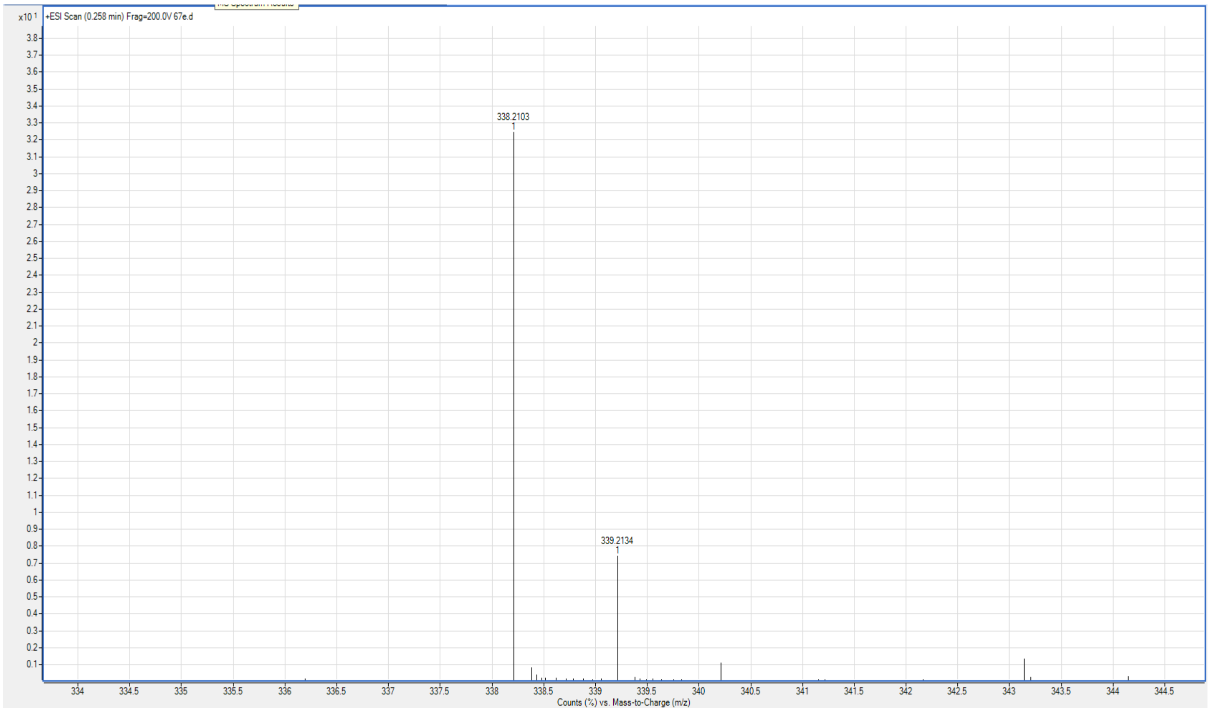

Figure S76: HR-MS spectrum of compound **6e,7e**.

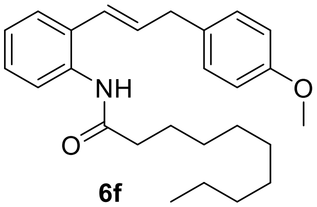

6f

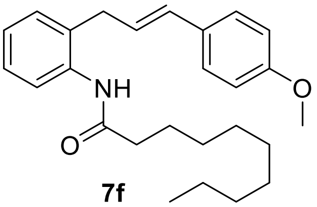

7f

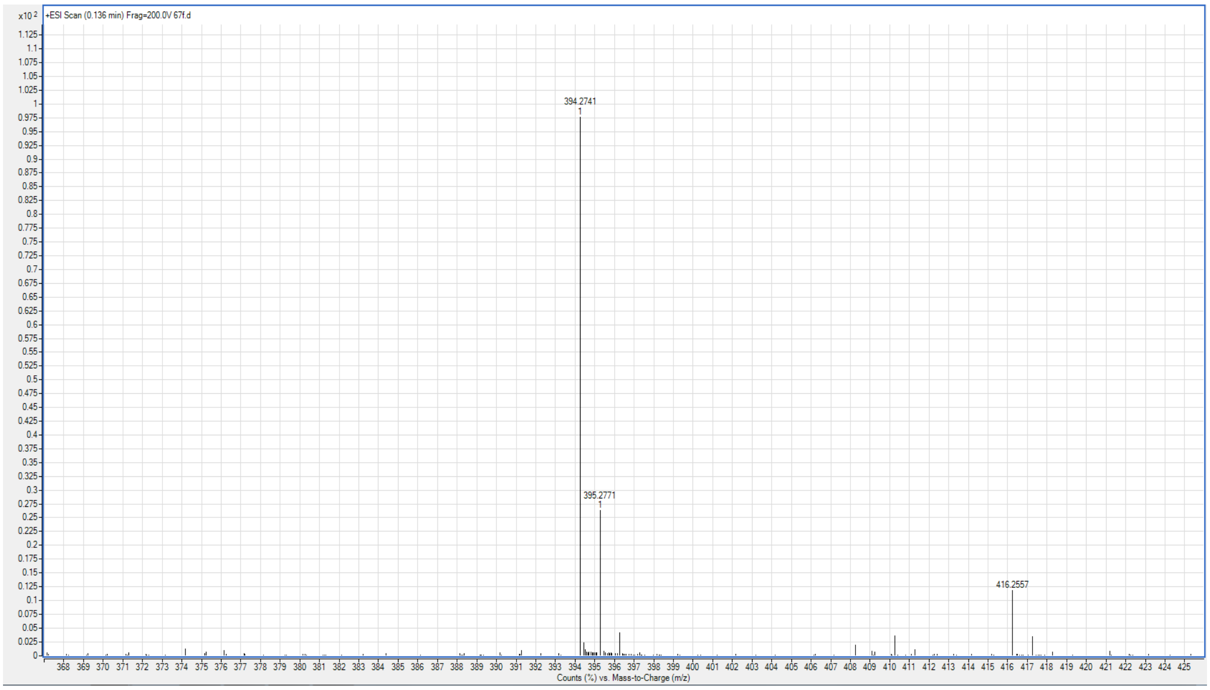

Figure S77: HR-MS spectrum of compound **6f,7f**.

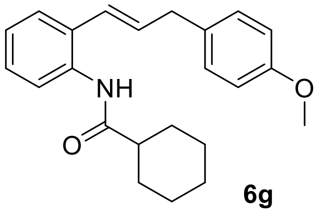

6g

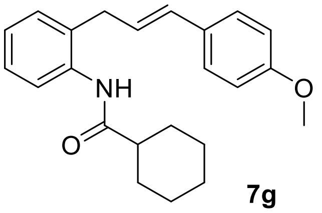

7g

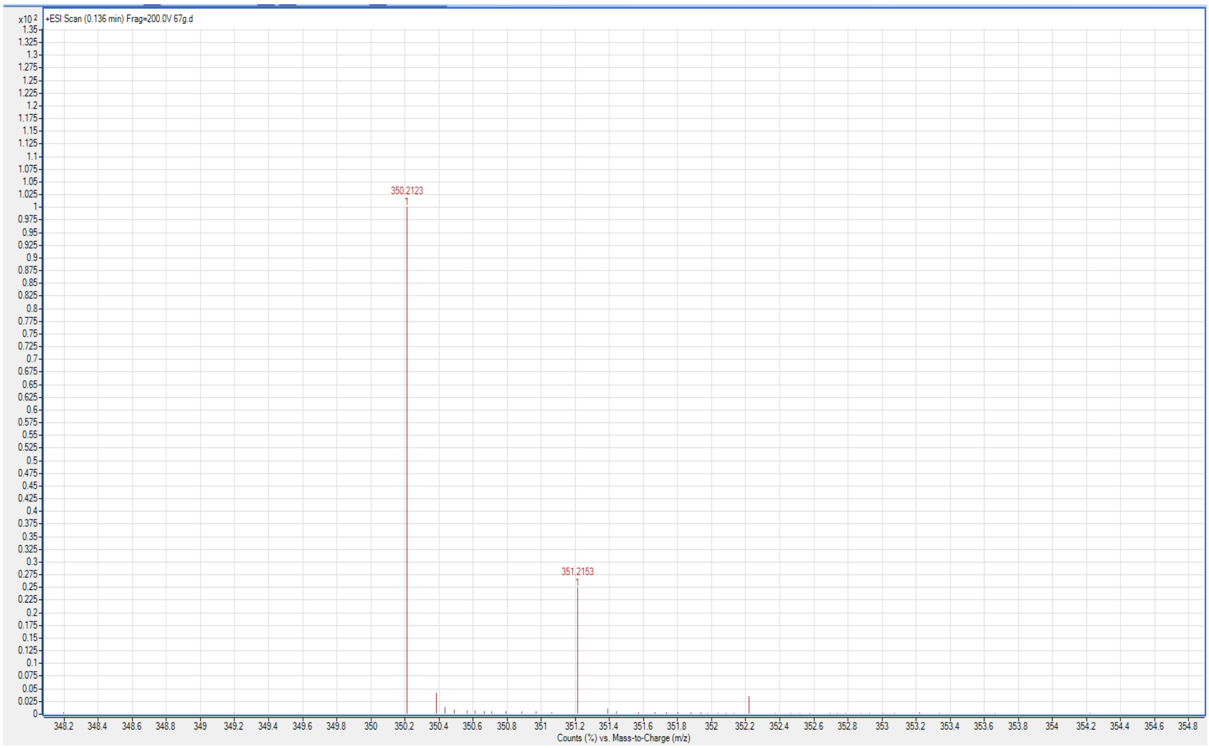

Figure S78: HR-MS spectrum of compound **6g,7g**.

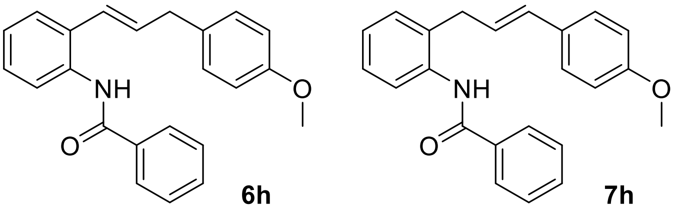

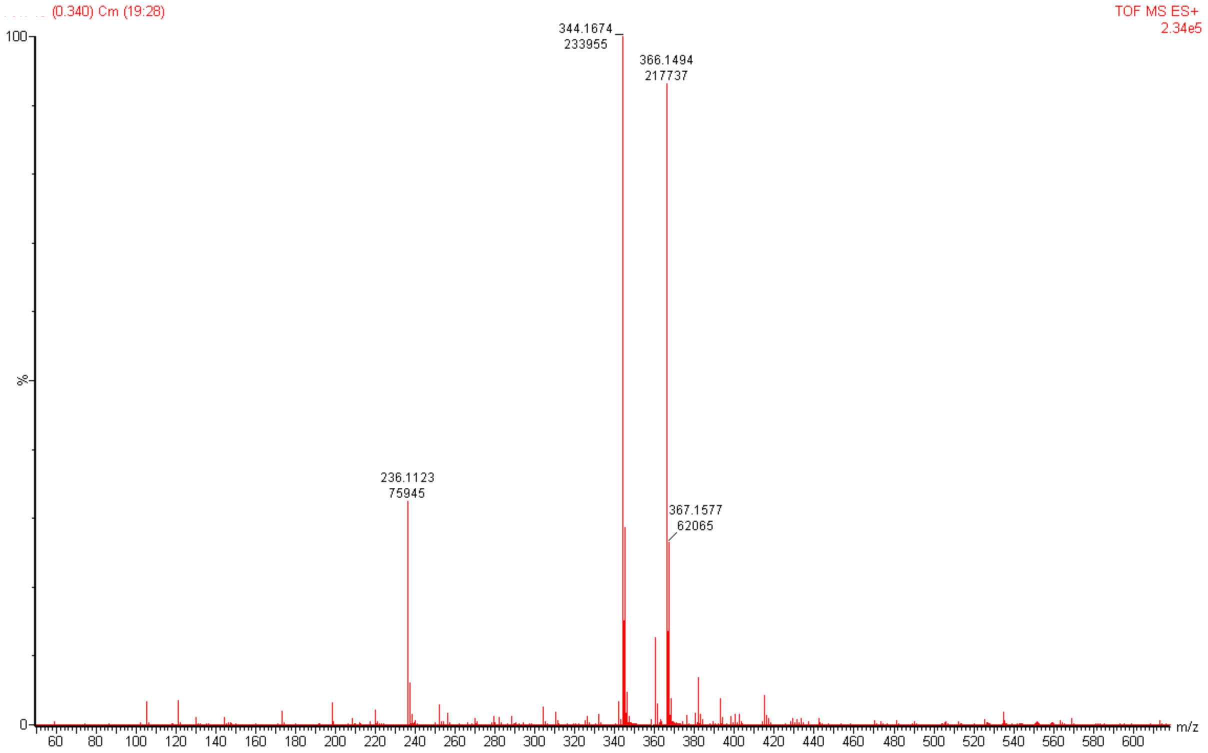

Figure S79: HR-MS spectrum of compound **6h,7h**.

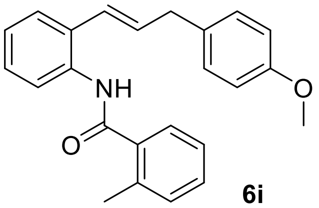

6i

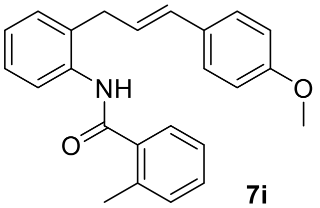

7i

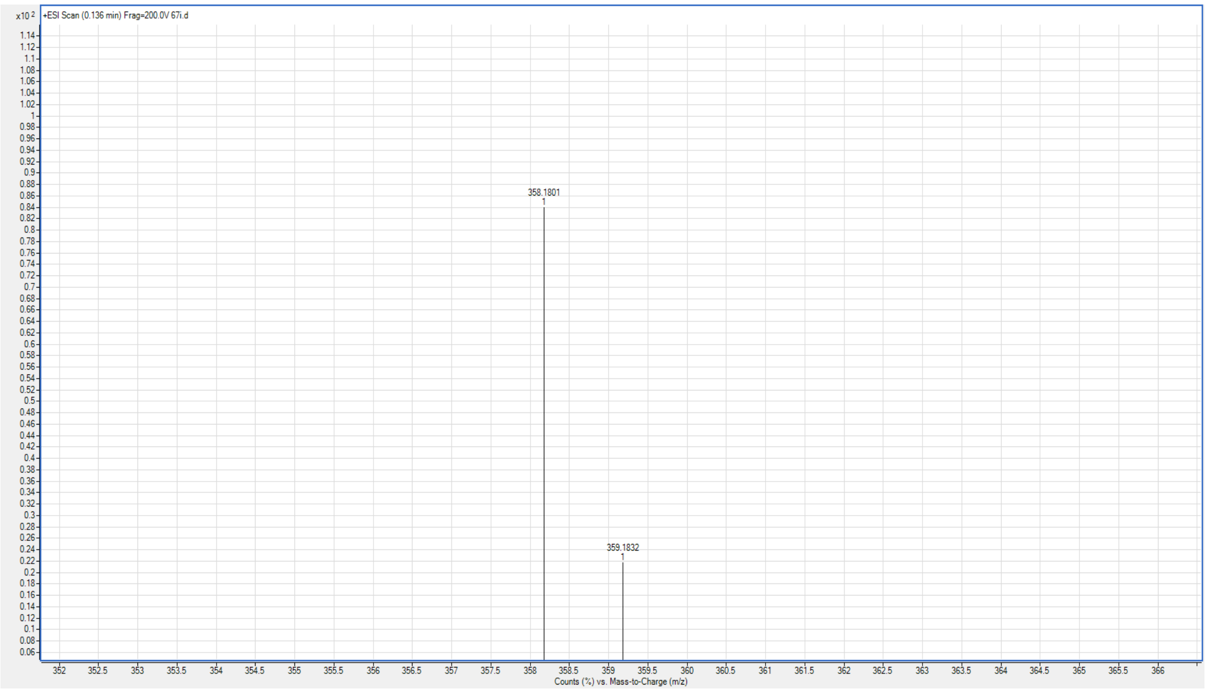

Figure S80: HR-MS spectrum of compound **6i,7i**.

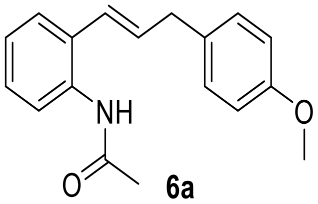

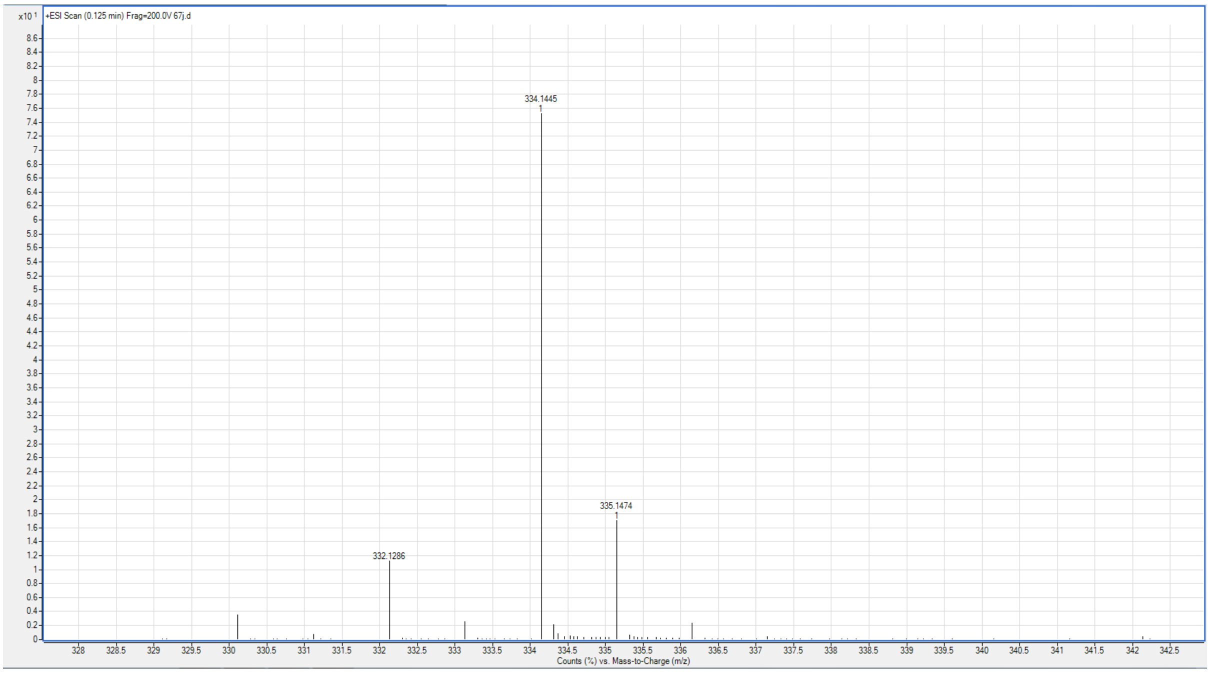

Figure S81: HR-MS spectrum of compound **6j,7j**.

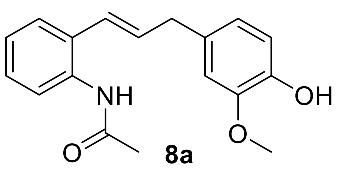

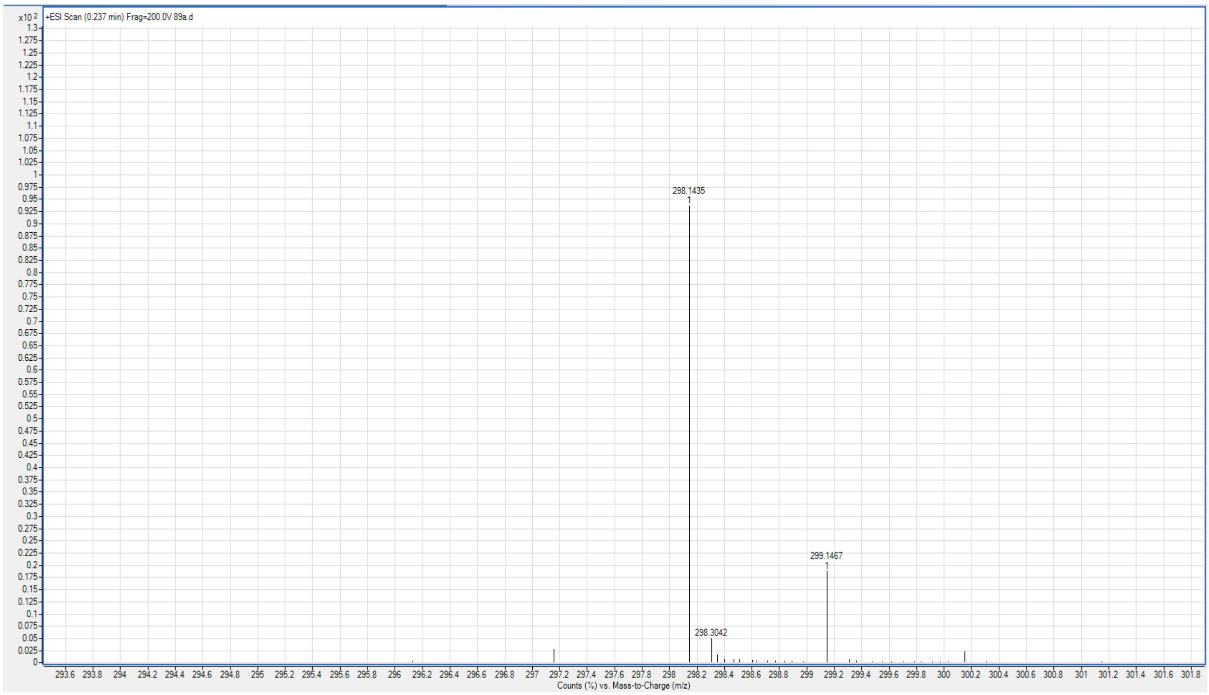

Figure S82: HR-MS spectrum of compound **8a,9a**.

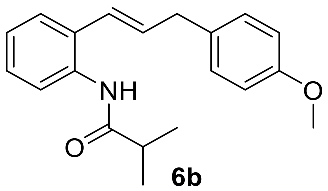

6b

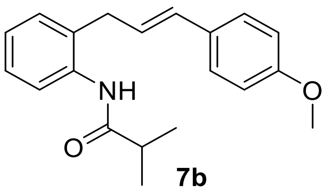

7b

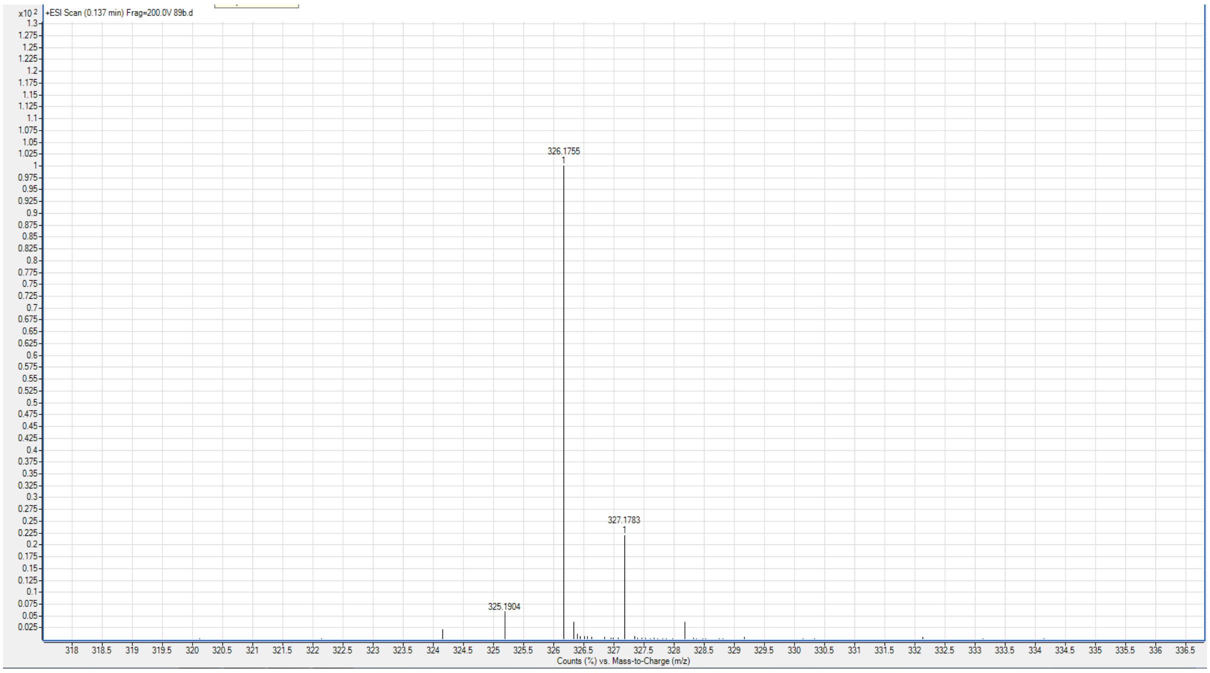

Figure S83: HR-MS spectrum of compound **8b,9b**.

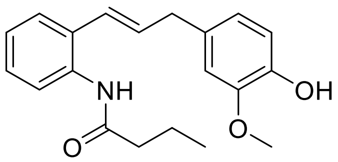

8c

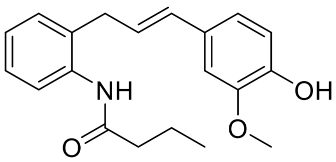

9c

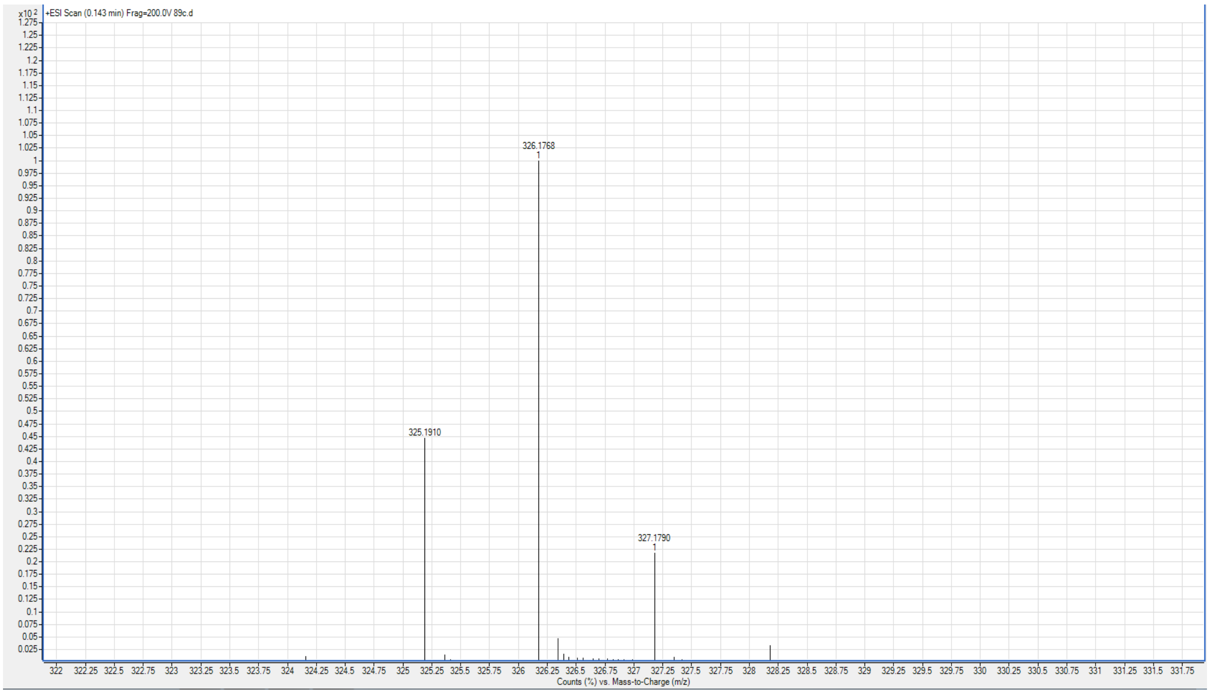

Figure S84: HR-MS spectrum of compound **8c,9c**.

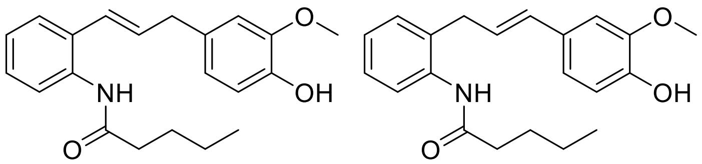

8d **9d**

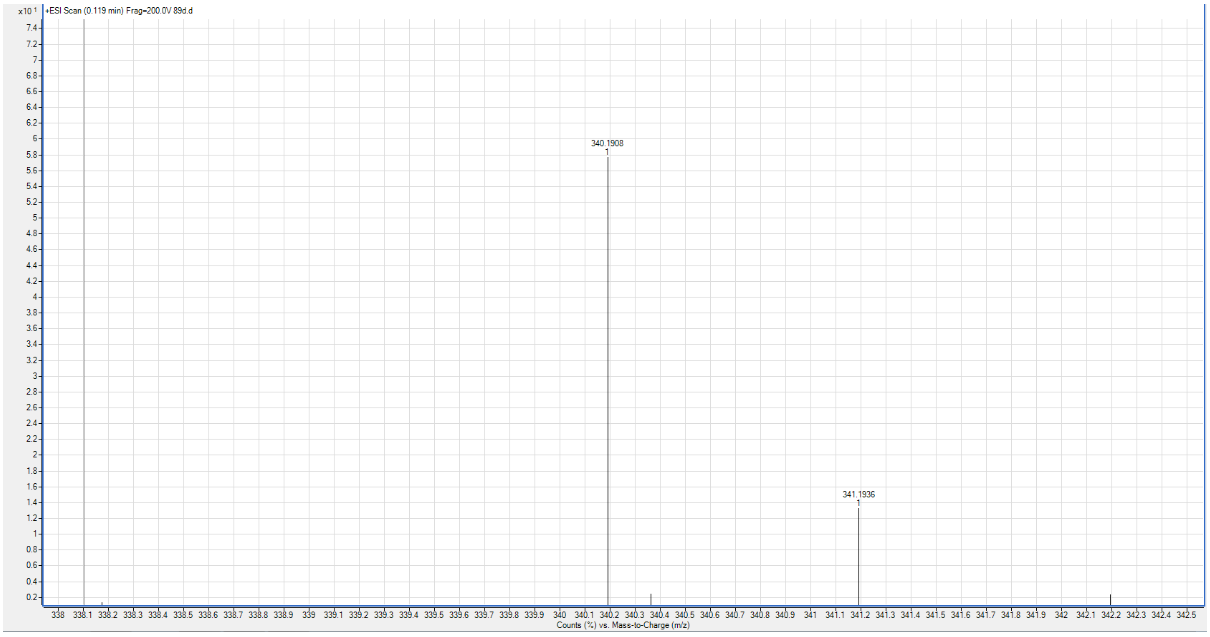

Figure S85: HR-MS spectrum of compound **8d,9d**.

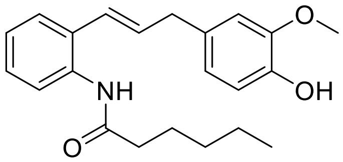

8e

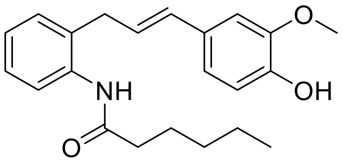

9e

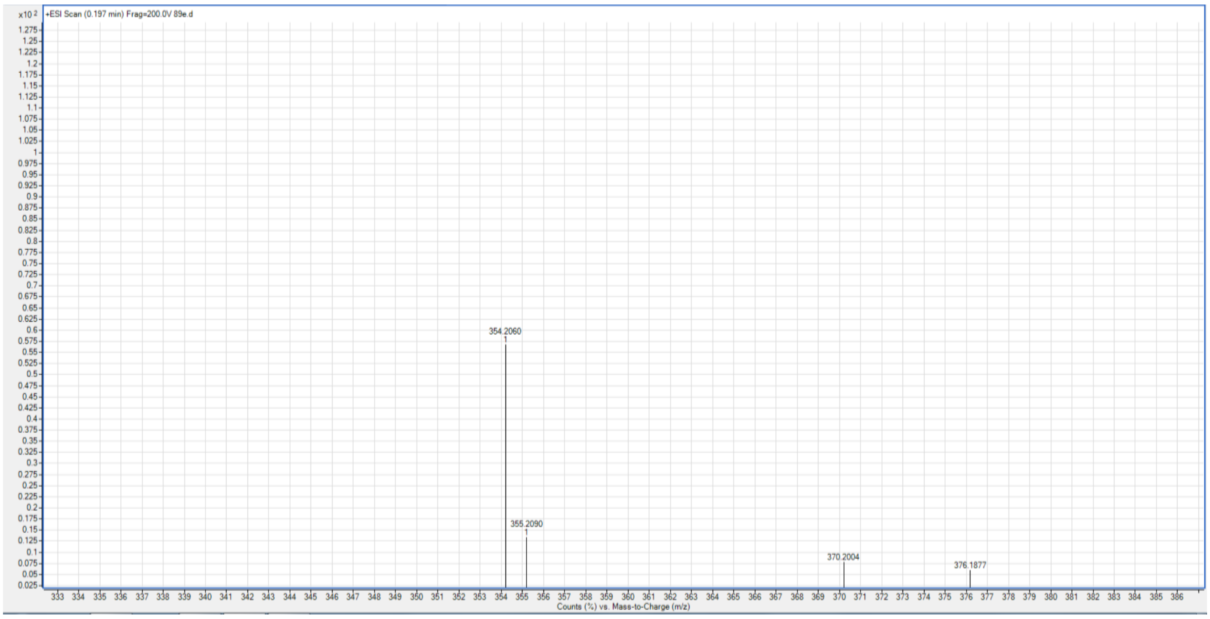

Figure S86: HR-MS spectrum of compound **8e,9e**.

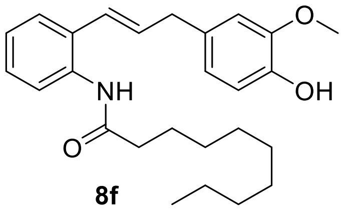

8f

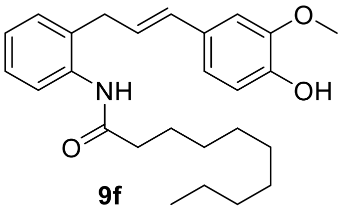

9f

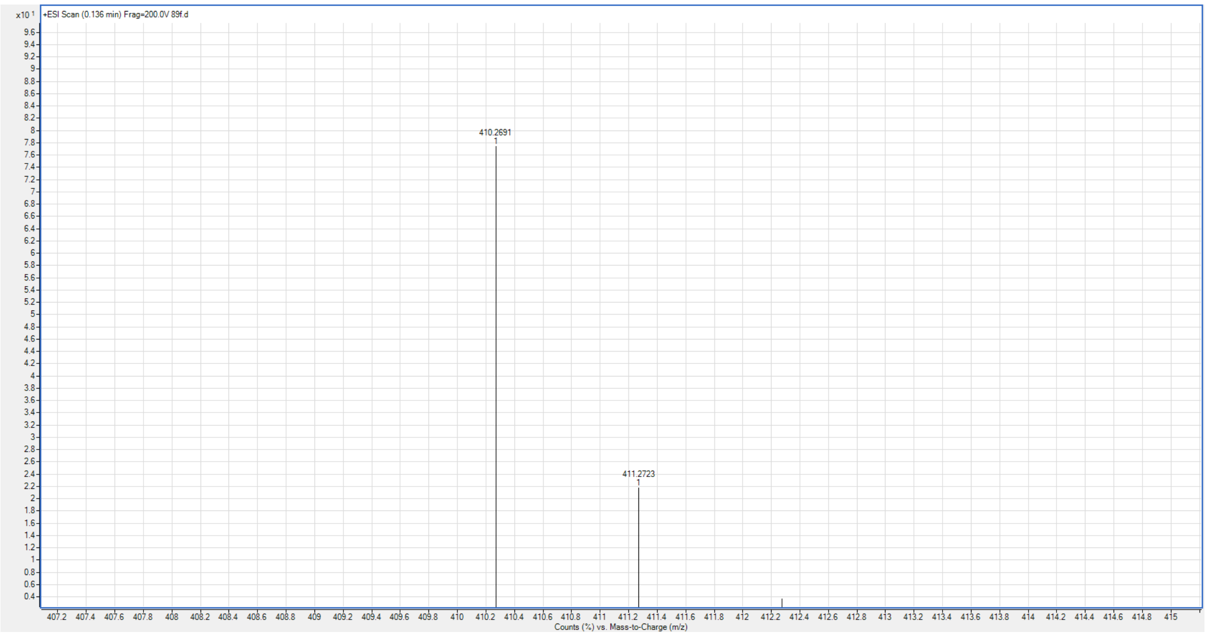

Figure S87: HR-MS spectrum of compound **8f,9f**.

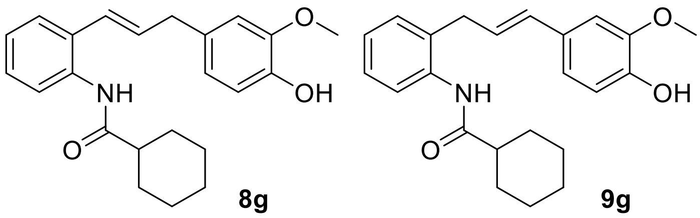

8g **9g**

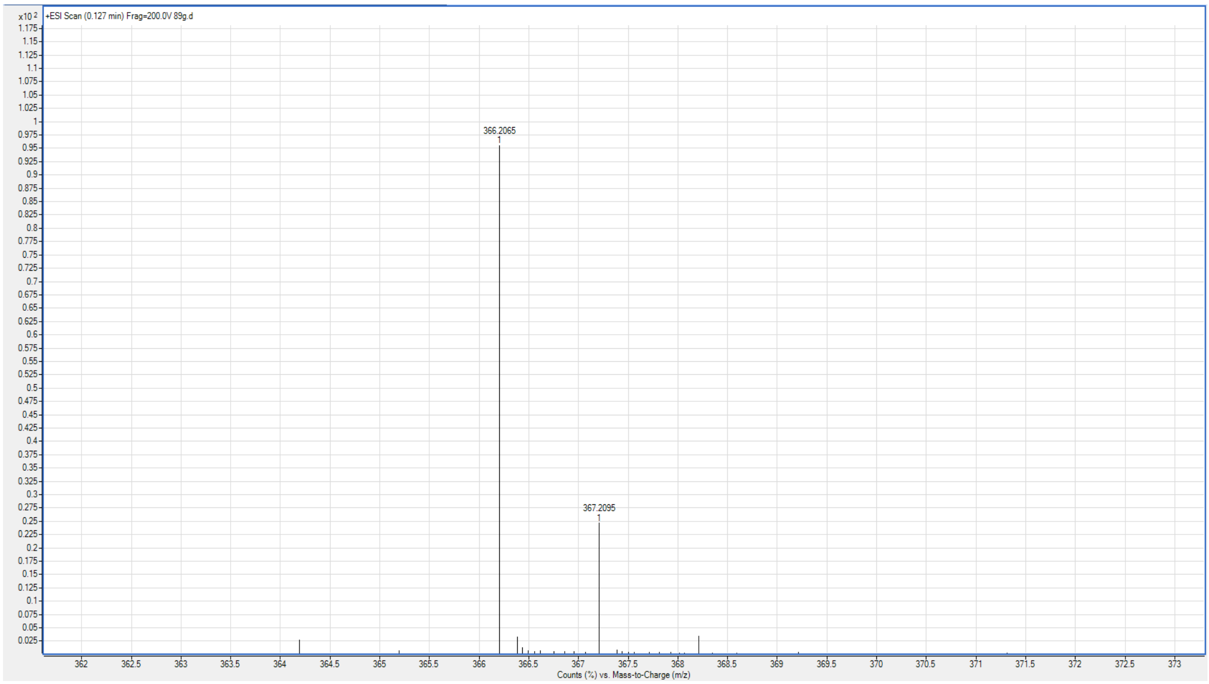

Figure S88: HR-MS spectrum of compound **8g,9g**.

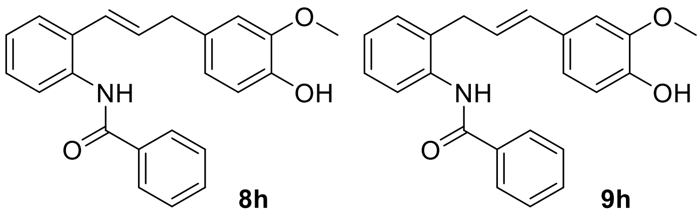

8h **9h**

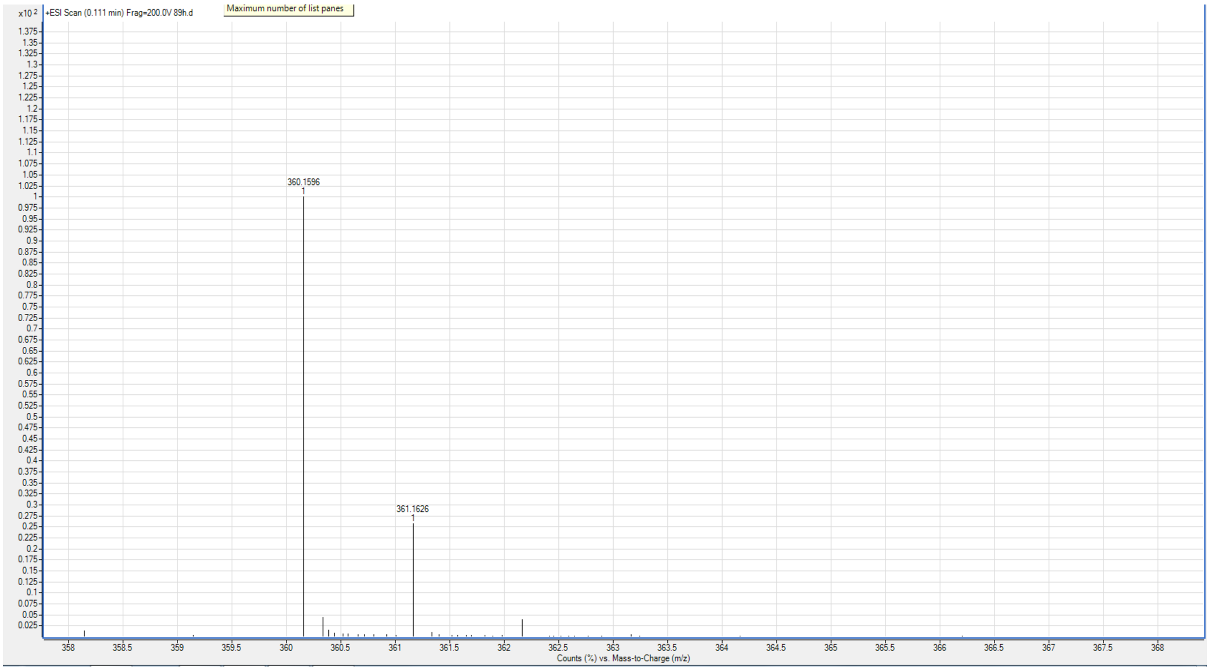

Figure S89: HR-MS spectrum of compound **8h,9h**.

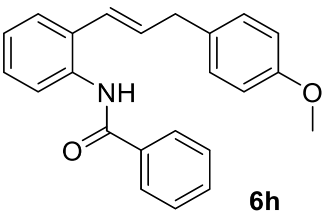

6h

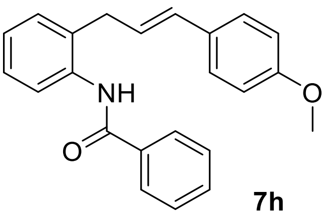

7h

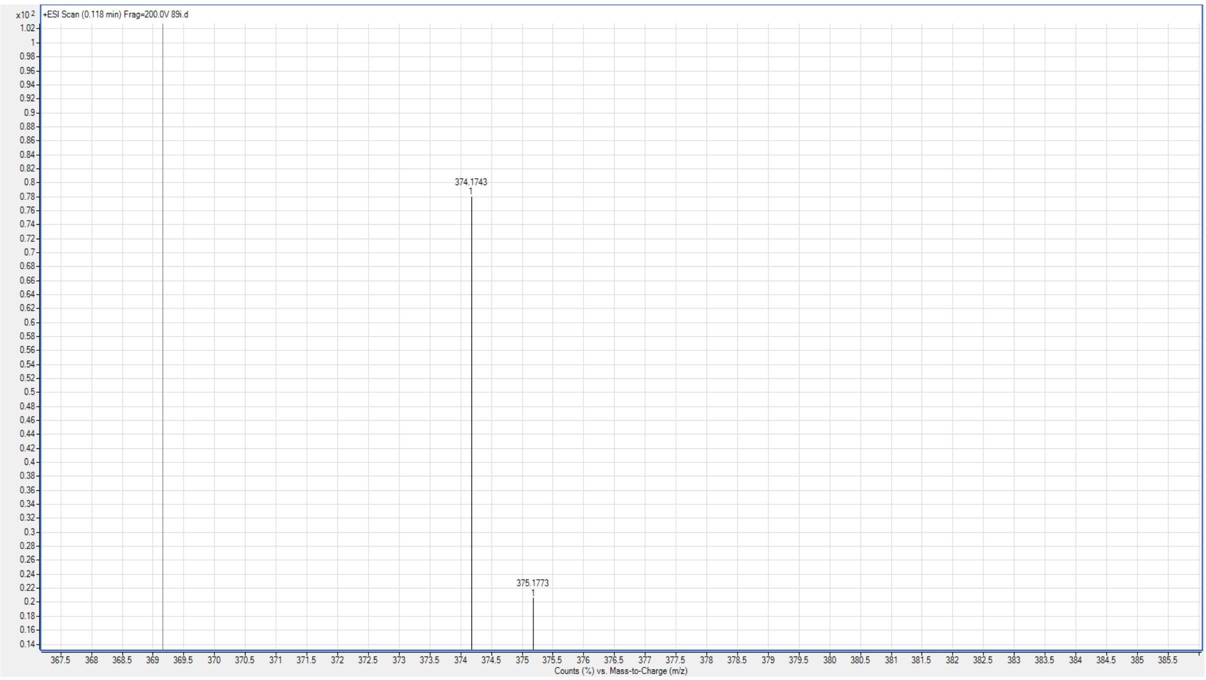

Figure S90: HR-MS spectrum of compound **8i,9i**.

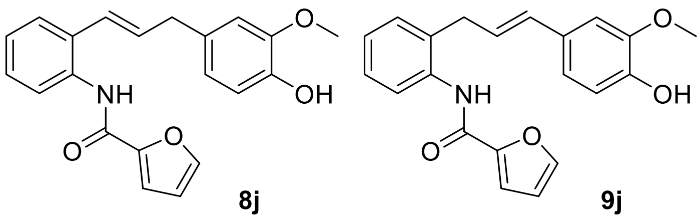

8j

9j

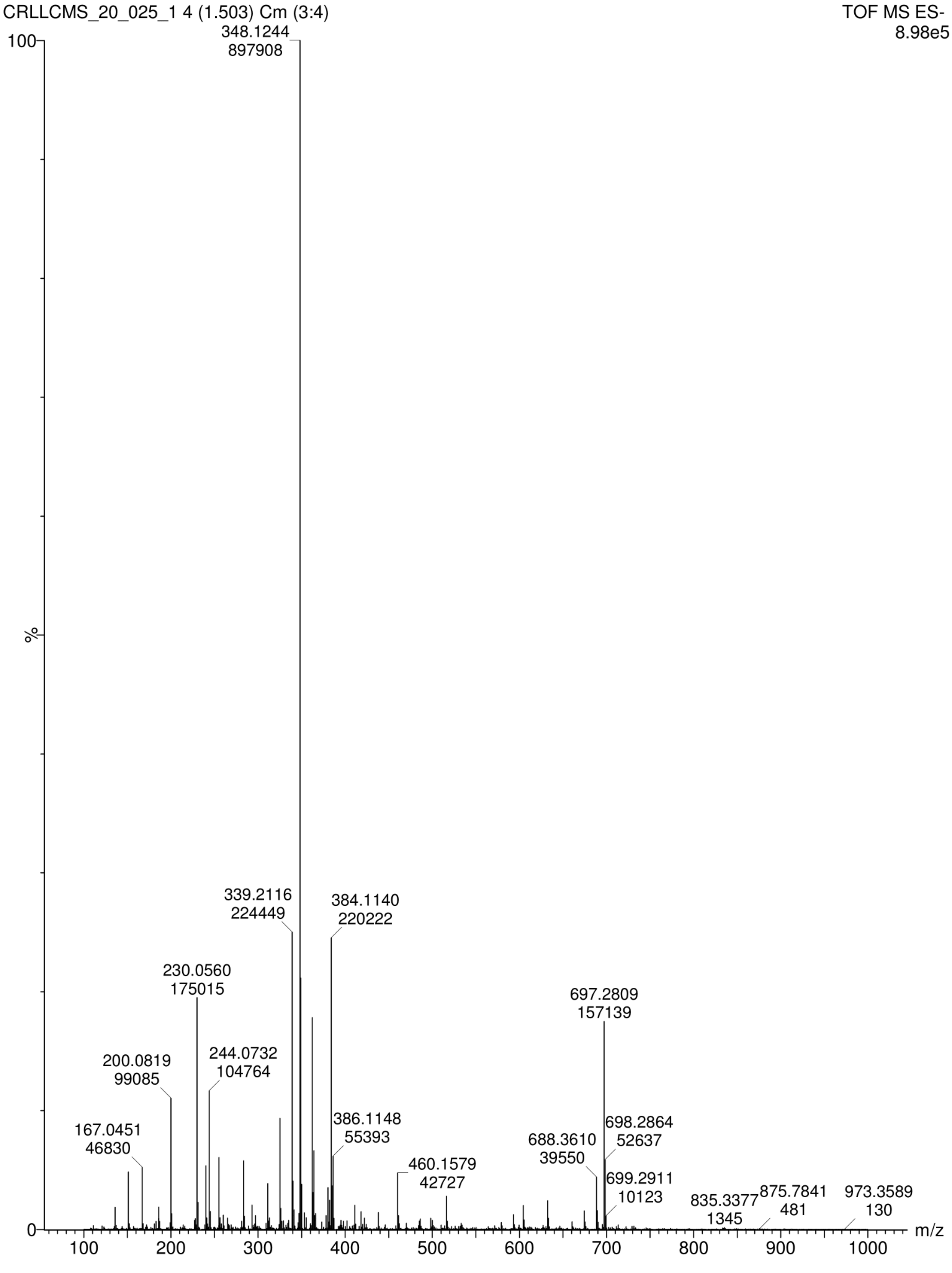

Figure S91: HR-MS spectrum of compound **8j,9j.**

7. Molecular modelling

General method.

For each compound, molecular mechanics calculations were first performed, with the Avogadro 1.2.0 software,[5,6] using the MMFF94 force field.[7] The most stable conformer was located through the Conformer Search function and its geometry was then re-optimised.

This optimised geometry was then used to prepare the input file for semi-empirical calculations performed using the PM7 method,[8] with the MOPAC2016 software.[9] The PRECISE keyword was used, corresponding to a convergence criterion of 10^{-6} kcal/mol for the self-consistent field (SCF) iterations.

7.1. Isomers 6a and 7a.

Energies after geometry optimisation (kcal/mol)

	MMFF94	**PM7**
E(**6a**)	42.17823	**−36.17743**
E(**7a**)	32.30116	**−37.57235**
ΔE	+9.87707	**+1.39492**

[5] Hanwell, M. D, Curtis, D. E., Lonie, D. C., Vandermeersch, T., Zurek, E. and Hutchison, G. R. Avogadro: An advanced semantic chemical editor, visualization, and analysis platform. *J. Cheminf.*, **2012**, 4, 17.
[6] Avogadro home page: https://avogadro.cc/ (accessed in September 2021).
[7] Halgren, T. A. Merck molecular force field. I. Basis, form, scope, parameterization, and performance of MMFF94. *J. Comput. Chem.*, **1996**, 17, 490-519.
[8] Stewart, J. J. P. Optimization of parameters for semiempirical methods VI: more modifications to the NDDO approximations and re-optimization of parameters. *J. Mol. Mod.*, **2013**, 19, 1-32.
[9] MOPAC2016, Stewart, J. J. P. Stewart Computational Chemistry, Colorado Springs, CO, USA. http://openmopac.net/ (accessed in September 2021).

Optimised geometries (PM7)

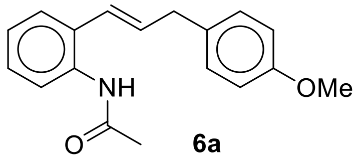

6a

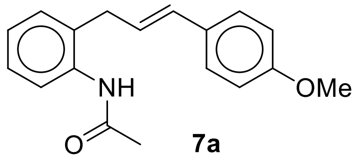

7a

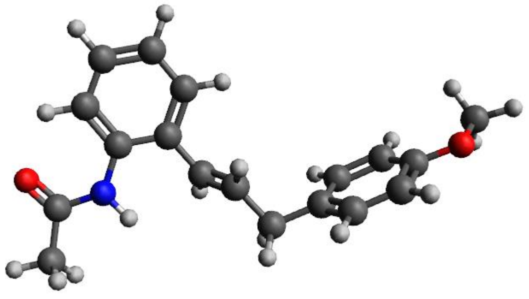

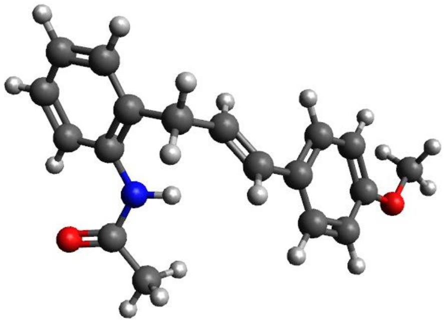

Cartesian coordinates of the optimised geometries (PM7)

6a:

C	-0.3136954900	-1.2306373200	-0.4689784100
C	-1.6089882400	-0.8372138500	-0.1410730900
C	0.7733442900	-0.5611777500	0.0870394100
C	0.5777480800	0.4958436900	0.9745270800
C	-0.7376824400	0.8967414700	1.2964637100
C	-1.8281236800	0.2237501100	0.7321839200
H	-0.1505085100	-2.0574065800	-1.1553178800
H	-2.4622960700	-1.3600310200	-0.5721314200
H	1.7893242000	-0.8651495300	-0.1656179600
H	-2.8512107500	0.5219541000	0.9709969500
C	1.7421371600	1.1520864600	1.5728754100
C	2.6050806400	1.8723201100	0.8499531000
H	1.8653750700	0.9841485000	2.6432209600
C	3.8236395500	2.5136127500	1.4356156600
H	2.4777511400	2.0208283900	-0.2234297700
C	5.0360203700	2.0088312000	0.7095568200
H	3.9136945900	2.3138050300	2.5243920000
H	3.7420127400	3.6198977000	1.3563446400
C	5.6024712800	2.7627109600	-0.3246872200
C	5.5865531600	0.7746018100	1.0519316500
C	6.7006007300	0.2806303200	0.3754850300
C	7.2506132200	1.0460300600	-0.6514811700
C	6.7107241600	2.2914592800	-1.0117047300
H	5.1692473900	3.7250365500	-0.5955750600
H	5.1398231700	0.1843511000	1.8520253700
H	7.1647345000	2.8620313800	-1.8176471700
O	8.3313016600	0.7053554900	-1.4039945500

N	-0.8977863100	2.0049688800	2.1611582900
C	-2.0664561000	2.3147085900	2.8581658700
O	-3.0744867800	1.6555311900	2.7629326800
C	-1.9573258200	3.5356545100	3.7323904100
H	-1.2362846900	3.3934953000	4.5477916100
H	-2.9340703600	3.7493826700	4.1989466200
H	-1.6680769500	4.4294017700	3.1651186500
H	-0.0704335400	2.5751438200	2.3117290500
C	8.9399197800	-0.5495351000	-1.1352330400
H	7.1186334900	-0.6801528300	0.6532187100
H	9.7534242500	-0.5746547000	-1.8735342200
H	8.2384587500	-1.3708376900	-1.3113462200
H	9.3447599100	-0.5779263400	-0.1190227600

7a:

C	-1.6758968100	2.6725828300	-0.4601319700
C	-1.9926141000	1.4786841600	-1.1033026300
C	-0.5902787100	2.7208987200	0.4106281300
C	0.1860220900	1.5894198200	0.6538572800
C	-0.1326640600	0.3848007000	-0.0109919100
C	-1.2256075400	0.3393183600	-0.8873957100
H	-2.2742464000	3.5629548000	-0.6340199900
H	-2.8432552600	1.4335077900	-1.7824514500
H	-0.3491517900	3.6577246900	0.9114868900
H	-1.4826737800	-0.5894191600	-1.4019701400
C	1.3170853500	1.6817428100	1.6366616000
C	2.6393612200	1.4943256400	0.9635623900
C	3.5982311000	0.7071065700	1.4693098100
C	4.8756608200	0.4653472900	0.8075690300
C	5.4912969400	-0.7885999600	0.9545566900
C	5.4947161200	1.4477690700	0.0322844300
C	6.7074375300	1.1955526100	-0.6060225000
C	7.2933203100	-0.0601451600	-0.4558219800
C	6.6932922200	-1.0633329600	0.3248533300
H	5.0173604300	-1.5541730000	1.5683262800
H	5.0299713600	2.4286965900	-0.0726088700
H	7.1801096700	-2.0301811600	0.4231517400
O	8.4670902500	-0.4593602300	-1.0115547500
N	0.6850251100	-0.7509653200	0.1957469400
C	0.2709709500	-2.0715796800	0.0238013500
O	-0.8515858400	-2.3610552600	-0.3176071400
C	1.3420747600	-3.0905208200	0.3087326400
H	2.2616944900	-2.9028216200	-0.2614942600
H	0.9854607900	-4.0962155900	0.0306322400
H	1.5973213000	-3.1228491800	1.3760417800
H	1.6361419900	-0.5728012300	0.5128806800
C	9.1463768400	0.4757861000	-1.8380875200
H	1.1585779700	0.9472458900	2.4577632100
H	1.3128373300	2.6708341300	2.1514666800
H	2.7553169100	2.0305700500	0.0219923300

H	3.4637275200	0.1858144700	2.4196643500
H	7.1744078800	1.9711715400	-1.2024982800
H	10.0272481200	-0.0972323300	-2.1597431400
H	9.4510659500	1.3557132100	-1.2631489800
H	8.5341653900	0.7521231400	-2.7020796300

7.2. Isomers 8a and 9a.

Energies after geometry optimisation (kcal/mol)

	MMFF94	**PM7**
E(**8a**)	40.30914	**−79.12742**
E(**9a**)	29.91479	**−79.99955**
ΔE	+10.39435	**+0.87213**

Optimised geometries (PM7)

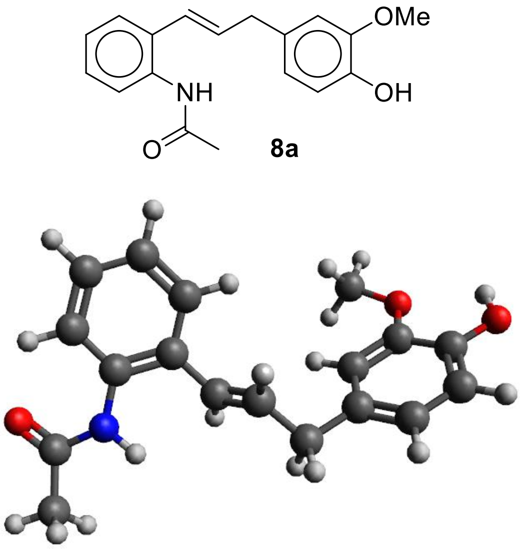

8a

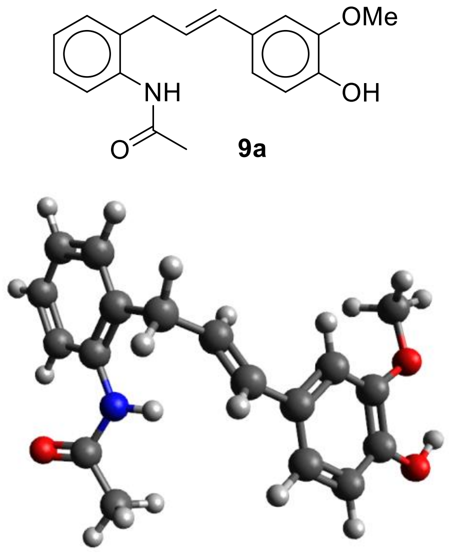

9a

Cartesian coordinates of the optimised geometries (PM7)

8a:

C	-0.0118857800	-1.3165428400	-0.3701373000
C	-1.3110076500	-1.0851827400	0.0764804900
C	1.0228313500	-0.4876844600	0.0545391300
C	0.7740275500	0.5700205600	0.9289868600
C	-0.5467900200	0.8062959500	1.3691869200
C	-1.5858225400	-0.0276430000	0.9375675100
H	0.1938177400	-2.1411943400	-1.0476169300
H	-2.1232386200	-1.7334139900	-0.2516003400

H	2.0403964800	-0.6654411700	-0.2923480500
H	-2.6115126800	0.1433419200	1.2718064800
C	1.8907496600	1.3932031000	1.3944200700
C	2.6679480700	2.0872260300	0.5565450000
H	2.0611034800	1.3743855600	2.4717795800
C	3.8554848000	2.8743765800	1.0131256300
H	2.4957190500	2.0962340900	-0.5204370200
C	5.1121331100	2.2119468500	0.5244576600
H	3.8934418400	2.9637346700	2.1210563800
H	3.7802223100	3.9199676400	0.6433162200
C	6.0598639200	2.9331792600	-0.1957158200
C	5.3190532500	0.8555090500	0.8110692500
C	6.4736001600	0.2355341000	0.3621813100
C	7.4330629700	0.9694591100	-0.3698176700
C	7.2289265300	2.3159823800	-0.6456628300
H	5.8974552300	3.9860930500	-0.4164600600
H	4.5703227500	0.3088033000	1.3795526800
H	7.9771983200	2.8696437900	-1.2100696700
O	8.5847154600	0.4183617200	-0.8345460300
O	6.8265873300	-1.0648706600	0.5800982000
C	5.7929616700	-1.9321142200	1.0275790400
H	5.4870782200	-1.6706293100	2.0459543700
H	6.2904815700	-2.9114117600	1.0143793100
H	4.9415718700	-1.9211960200	0.3395571900
N	-0.7771024000	1.9199009300	2.2105509400
C	-1.8835496000	2.0683000200	3.0492150100
O	-2.7645927200	1.2440261800	3.1102072900
C	-1.8767201700	3.3363578000	3.8596632400
H	-1.0258553000	3.3794808300	4.5516956300
H	-2.7936255900	3.3942686800	4.4709350600
H	-1.8543966900	4.2325101600	3.2259128600
H	-0.0451842500	2.6236242200	2.2303565000
H	8.6296794700	-0.5475945800	-0.6140995800

9a:

C	-1.5818517100	2.7063448000	-0.5059421700
C	-1.9004085200	1.5127699400	-1.1488039400
C	-0.5064033600	2.7491841300	0.3773978600
C	0.2589659600	1.6126705400	0.6324418500
C	-0.0598741500	0.4087468800	-0.0335908600
C	-1.1438074300	0.3687055000	-0.9215967300
H	-2.1714504200	3.6006130900	-0.6897801800
H	-2.7446946800	1.4712678600	-1.8362378900
H	-0.2651821100	3.6853988200	0.8792972000
H	-1.4022913300	-0.5597280300	-1.4361124200
C	1.3797074800	1.7002659200	1.6273162900
C	2.7060139600	1.5059325700	0.9647829900
C	3.6533339600	0.7064220500	1.4715623500
C	4.9254907500	0.4499398500	0.8011894300
C	5.4784042600	-0.8313714100	0.8622029600

C	5.5819141000	1.4691360200	0.0961042700
C	6.7760624200	1.1877771300	-0.5488356700
C	7.3223194600	-0.1133915700	-0.4967613200
C	6.6759979700	-1.1215228300	0.2105048500
H	4.9726364900	-1.6164174300	1.4207497000
H	5.1546554600	2.4679803700	0.0736049700
H	7.1120373200	-2.1184057800	0.2445192100
O	8.4845459200	-0.4495708700	-1.1119339200
O	7.5485017500	2.0754927600	-1.2404935100
C	6.9328148800	3.3052415900	-1.5983430300
H	6.0132541000	3.1371240000	-2.1677154200
H	7.7003120300	3.7725169500	-2.2307295500
H	6.7478051200	3.9138413400	-0.7071902400
N	0.7483443200	-0.7319042100	0.1822917900
C	0.3281308400	-2.0504310000	0.0042714600
O	-0.7934575900	-2.3324786600	-0.3457833200
C	1.3923549800	-3.0753448800	0.2923857400
H	2.2935841400	-2.9209204000	-0.3165522700
H	1.0123537400	-4.0846260400	0.0612710700
H	1.6852924800	-3.0749993400	1.3501962400
H	1.6982921800	-0.5600231100	0.5056925700
H	8.8621217300	0.3234374500	-1.6067361800
H	1.2098858100	0.9665200700	2.4469163700
H	1.3750784800	2.6889769800	2.1426164600
H	2.8336187600	2.0440555600	0.0252451800
H	3.5155150200	0.1827793300	2.4198886500

7.3. Isomers 6b and 7b.

Energies after geometry optimisation (kcal/mol)

	MMFF94	**PM7**
E(**6b**)	45.37892	**−46.03283**
E(**7b**)	35.22410	**−47.86614**
ΔE	+10.15482	**+1.83331**

Optimised geometries (PM7)

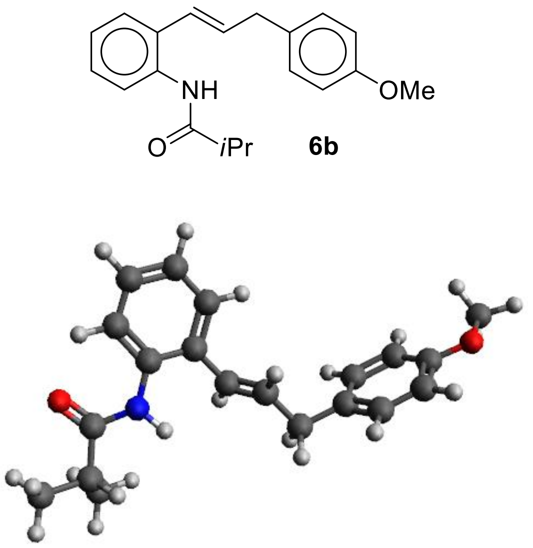

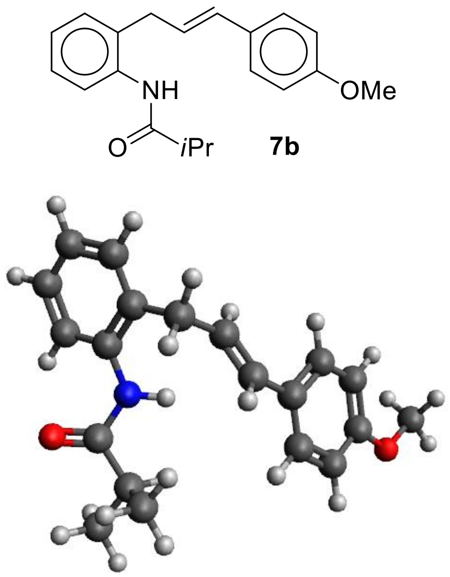

Cartesian coordinates of the optimised geometries (PM7)

6b:

C	-0.3066715400	-1.2771044900	-0.4585938000
C	-1.5993983500	-0.8532931900	-0.1587419300
C	0.7846017200	-0.6139080600	0.0966029000
C	0.5958857400	0.4680926100	0.9551030700
C	-0.7162549000	0.8995287700	1.2475582100
C	-1.8114450100	0.2325130600	0.6851008100
H	-0.1490630600	-2.1230026700	-1.1225473800
H	-2.4555188000	-1.3717887900	-0.5889479600
H	1.7979773100	-0.9428858800	-0.1331419300
H	-2.8308326400	0.5561414900	0.9044719200
C	1.7612928500	1.1197383200	1.5562573600
C	2.6494149800	1.8045173900	0.8294088600
H	1.8614533000	0.9814516500	2.6331892200
C	3.8667748700	2.4438325800	1.4193825900
H	2.5432775000	1.9253809600	-0.2496958800

C	5.0816369300	1.9563470900	0.6859421900
H	3.9606527900	2.2306942300	2.5054919100
H	3.7774238300	3.5506162900	1.3547200100
C	5.6622502900	2.7409663700	-0.3170547500
C	5.6213956000	0.7074901000	0.9907448800
C	6.7393666900	0.2301289000	0.3088773300
C	7.3047769800	1.0271838700	-0.6851044500
C	6.7747870400	2.2867637500	-1.0087805000
H	5.2371461800	3.7143034000	-0.5600064400
H	5.1627244900	0.0928842500	1.7654272100
H	7.2395225800	2.8813448500	-1.7908972500
O	8.3917195700	0.7065136700	-1.4375086300
N	-0.8729629700	2.0343195300	2.0779423600
C	-2.0225173500	2.3251807400	2.8154575300
O	-3.0095416000	1.6312152800	2.7643154100
C	-1.9125307900	3.5766126600	3.6846896700
C	-1.3477689800	3.1542824000	5.0420064000
C	-3.3046710800	4.1894463700	3.8278225700
H	-1.2335357900	4.3266942500	3.2120744900
H	-0.0466494600	2.6118762800	2.2020849300
C	9.0026825400	-0.5521573900	-1.1930840600
H	7.1481225800	-0.7427526100	0.5565532000
H	9.8277001900	-0.5530957200	-1.9189617000
H	8.3085183400	-1.3718053100	-1.4025322600
H	9.3918759900	-0.6065095900	-0.1718234800
H	-1.9852831300	2.3976674000	5.5214972200
H	-1.2885953400	4.0072507000	5.7281984700
H	-0.3423780300	2.7280591000	4.9554226000
H	-4.0206294400	3.4597300600	4.2357075700
H	-3.7039922100	4.5125346100	2.8585740000
H	-3.2957889100	5.0570121900	4.4951184700

7b:

C	-1.7276825400	2.6888668400	-0.4450507500
C	-2.0187824100	1.5153595700	-1.1365733200
C	-0.6448923900	2.7261221800	0.4296043100
C	0.1579613900	1.6036353300	0.6269152400
C	-0.1287532400	0.4250225800	-0.0950755200
C	-1.2234332000	0.3873919600	-0.9699989500
H	-2.3458345500	3.5719115600	-0.5834263100
H	-2.8710567900	1.4781655000	-1.8136742300
H	-0.4285603800	3.6459188600	0.9713350600
H	-1.4567491300	-0.5279438000	-1.5179033800
C	1.2712701700	1.6737828100	1.6317058300
C	2.6088988500	1.5102566100	0.9838162800
C	3.5404059500	0.6769512300	1.4660920700
C	4.8242943000	0.4421449000	0.8143048900
C	5.3790715300	-0.8476230800	0.8530353900
C	5.5039065500	1.4641991000	0.1495504100
C	6.7193890300	1.2175486200	-0.4860889300

C	7.2443630900	-0.0729066100	-0.4459921800
C	6.5821489300	-1.1168966500	0.2231484300
H	4.8552162300	-1.6452771200	1.3796734800
H	5.0847847700	2.4706087400	0.1296997500
H	7.0228513900	-2.1103616200	0.2368509500
O	8.4113027300	-0.4724188800	-1.0158224800
N	0.7208126700	-0.6982267000	0.0411036700
C	0.2995655900	-2.0228482400	-0.0787981900
O	-0.8472040900	-2.3086190700	-0.3286803500
C	1.4117016900	-3.0493330800	0.1319152500
H	2.3461348500	-2.7064069500	-0.3785301600
C	0.9712654700	-4.3769205100	-0.4816549100
C	1.6675654300	-3.1913944100	1.6332041100
H	1.6841086000	-0.5083541200	0.3094863600
C	9.1558522400	0.5056616300	-1.7284062200
H	1.0988168400	0.9124703800	2.4258002200
H	1.2512834700	2.6466585400	2.1756760600
H	2.7581153600	2.0996377100	0.0794907400
H	3.3747419000	0.1046646900	2.3817265600
H	7.2342998400	2.0234679800	-0.9966399400
H	10.0249468000	-0.0718751100	-2.0730059400
H	9.4740290000	1.3158305900	-1.0651618000
H	8.5859939900	0.8852412200	-2.5821362500
H	0.7986483400	-4.2819131600	-1.5612781800
H	0.0228789300	-4.7231627900	-0.0454420700
H	1.7188507100	-5.1608160000	-0.3264146300
H	2.0122488400	-2.2551139400	2.0848876900
H	2.4291178600	-3.9552648000	1.8316331400
H	0.7570670200	-3.4979763700	2.1662307700

7.4. Isomers 8b and 9b.

Energies after geometry optimisation (kcal/mol)

	MMFF94	**PM7**
E(**8b**)	43.51387	**−88.98630**
E(**9b**)	32.83785	**−90.35175**
ΔE	+10.67602	**+1.36545**

Optimised geometries (PM7)

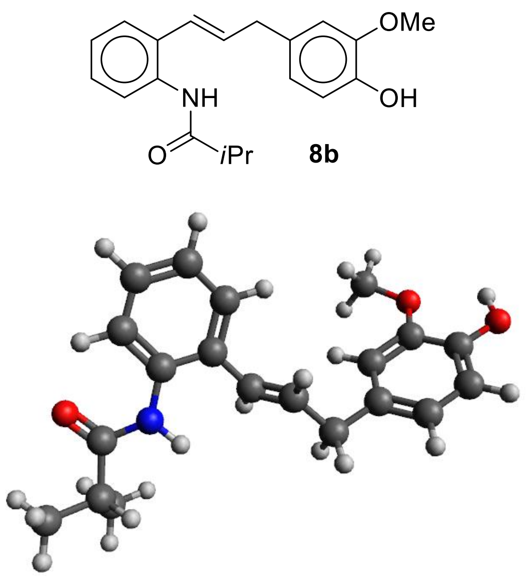

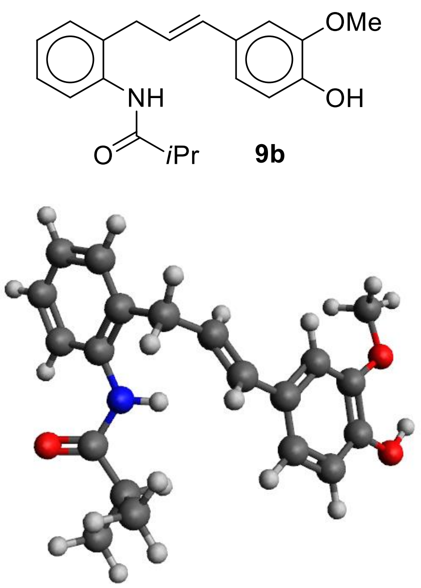

Cartesian coordinates of the optimised geometries (PM7)

8b:

C	0.0084369600	-1.3271940800	-0.3609081100
C	-1.2893622400	-1.0478315300	0.0617240000
C	1.0643246300	-0.5328927000	0.0776552700
C	0.8373172600	0.5375233400	0.9421028800
C	-0.4815358800	0.8226433200	1.3583411200
C	-1.5418642300	0.0235603300	0.9124427900
H	0.1967474800	-2.1618771700	-1.0308774500
H	-2.1177820300	-1.6690501000	-0.2776107000
H	2.0806540100	-0.7478125200	-0.2508819800
H	-2.5662074300	0.2333629600	1.2276527700
C	1.9737543000	1.3261461400	1.4208731500
C	2.7546671300	2.0297355300	0.5948803600
H	2.1515814200	1.2741921400	2.4957150300
C	3.9577610400	2.7878262100	1.0601796400

H	2.5728895100	2.0699606200	-0.4798853500
C	5.1956314100	2.1383762500	0.5106110200
H	4.0212854800	2.8271482700	2.1696098700
H	3.8821959800	3.8495562600	0.7397191300
C	6.0997668600	2.8674724000	-0.2560968100
C	5.4259909300	0.7827591700	0.7831148600
C	6.5593783500	0.1715064300	0.2725821000
C	7.4743054500	0.9132480600	-0.5070187300
C	7.2478921200	2.2591822100	-0.7677545000
H	5.9185713900	3.9193891100	-0.4666683700
H	4.7108665200	0.2298523500	1.3874439000
H	7.9623861500	2.8187377500	-1.3688729700
O	8.6028076100	0.3706157100	-1.0342806500
O	6.9312581700	-1.1273984000	0.4670407100
C	5.9273409000	-2.0026050300	0.9638516000
H	5.6840614200	-1.7545436300	2.0022420300
H	6.4242135900	-2.9807118900	0.9075400100
H	5.0354115300	-1.9844955600	0.3293032300
N	-0.6878251500	1.9488695800	2.1887375400
C	-1.7929820900	2.1283484600	3.0241425600
O	-2.6934663000	1.3254147600	3.0757842900
C	-1.7510281500	3.4055153400	3.8606627800
C	-3.1789188800	3.9284344000	4.0096450900
H	0.0723281600	2.6215932800	2.2230716100
H	8.6654774300	-0.5954862000	-0.8192654900
H	-3.8396157900	3.1628122500	4.4443594500
H	-3.6109115100	4.2038325700	3.0398027800
H	-3.2186608800	4.8094465200	4.6583413300
H	-1.1286558300	4.1883330100	3.3642671300
C	-1.1415887100	3.0487841100	5.2174021000
H	-1.7256907500	2.2664174400	5.7234172800
H	-1.1227942300	3.9189857500	5.8838914500
H	-0.1141745200	2.6802812600	5.1228114300

9b:

C	-1.6672688600	2.7161801100	-0.4863728300
C	-1.9508297900	1.5420019300	-1.1800818200
C	-0.5921389700	2.7549522000	0.3973923600
C	0.2115323500	1.6339890900	0.6009078500
C	-0.0669469900	0.4549174900	-0.1234706700
C	-1.1550123500	0.4155263000	-1.0068290900
H	-2.2854745100	3.5984299800	-0.6302134000
H	-2.7975065800	1.5033316800	-1.8642885600
H	-0.3825963200	3.6745804200	0.9420840700
H	-1.3825649300	-0.5006639600	-1.5558954400
C	1.3161023500	1.7074904800	1.6150830900
C	2.6579081900	1.5303252400	0.9800377600
C	3.5775360600	0.6915251600	1.4749796300
C	4.8575239800	0.4286690500	0.8232020600
C	5.3759832700	-0.8685111600	0.8514766100

C	5.5528398400	1.4529140900	0.1648366700
C	6.7518946200	1.1613898400	-0.4665931000
C	7.2640899400	-0.1542924000	-0.4465656400
C	6.5785934000	-1.1683804900	0.2142582400
H	4.8370812300	-1.6590096000	1.3710254100
H	5.1522078600	2.4629391100	0.1668900400
H	6.9884917100	-2.1768427000	0.2234246500
O	8.4297322800	-0.4991496500	-1.0501122700
O	7.5610129200	2.0520624300	-1.1114311900
C	6.9693229800	3.2903699500	-1.4805965400
H	6.0615823800	3.1365880600	-2.0723924200
H	7.7576161100	3.7504567600	-2.0923589100
H	6.7709116500	3.8978941600	-0.5916136700
N	0.7837970500	-0.6667900800	0.0160198000
C	0.3671957700	-1.9929887500	-0.1094238600
O	-0.7780218700	-2.2814241400	-0.3622199900
C	1.4847202800	-3.0143048900	0.0969636200
H	2.4156949200	-2.6659543800	-0.4167889900
C	1.0488366600	-4.3434197000	-0.5163837900
C	1.7477052800	-3.1562204300	1.5969498600
H	1.7457279100	-0.4767733600	0.2884464500
H	8.8352280500	0.2799723700	-1.5123218700
H	1.1320499200	0.9534592500	2.4137528300
H	1.2960923000	2.6844278200	2.1514557700
H	2.8181256800	2.1091301100	0.0706600200
H	3.4039079500	0.1314666300	2.3966939200
H	2.0970202200	-2.2206804100	2.0465779300
H	2.5087349800	-3.9216447800	1.7918323900
H	0.8394362000	-3.4610392800	2.1347712000
H	0.8674457100	-4.2469242100	-1.5945612100
H	0.1065818900	-4.6977871400	-0.0734262300
H	1.8034193100	-5.1222496900	-0.3690665800
